# Site-selective and metal-free C–H phosphonation of arenes *via* photoactivation of thianthrenium salts†

**DOI:** 10.1039/d3ra04512a

**Published:** 2023-08-08

**Authors:** Albert Gallego-Gamo, David Reyes-Mesa, Axel Guinart-Guillem, Roser Pleixats, Carolina Gimbert-Suriñach, Adelina Vallribera, Albert Granados

**Affiliations:** a Departament de Química and Centro de Innovación en Química Avanzada (ORFEO-CINQA), Universitat Autònoma de Barcelona Cerdanyola del Vallès 08193 Barcelona Spain carolina.gimbert@uab.es adelina.vallribera@uab.es albert.granados@uab.es

## Abstract

Aryl phosphonates are prevalent moieties in medicinal chemistry and agrochemicals. Their chemical synthesis normally relies on the use of precious metals, harsh conditions or aryl halides as substrates. Herein, we describe a sustainable light-promoted and site-selective C–H phosphonation of arenes *via* thianthrenation and the formation of an electron donor–acceptor complex (EDA) as key steps. The method tolerates a wide range of functional groups including biomolecules. The use of sunlight also promotes this transformation and our mechanistic investigations support a radical chain mechanism.

## Abstract 2

Aryl phosphonates are accessed by visible-light irradiation of new EDA complex between thianthrenium salts as electron-acceptors and phosphites as electron-donor molecules.

## Graphical abstract

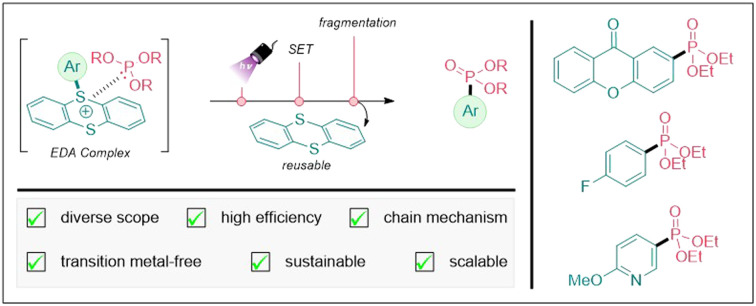

Organophosphorus compounds are ubiquitous compounds in pharmaceutical and agrochemical fields due to their interesting bioactivities. For example, aryl phosphonates can serve as suitable non-hydrolyzable phosphate mimics in biomedical chemistry.^1^ Thus, the preparation of such organic architectures has received great attention by synthetic organic chemists. Classically, the aryl phosphonate moiety can be accessed *via* Grignard or organolithium reagents and through transition-metal catalyzed processes (Scheme 1A).^2^ However, in recent years the implementation of electrocatalytic^3^ and visible light-mediated^4^ methods as a milder strategy for the preparation of phosphonates have been applied with success. In this field, photo-,^5^ metallaphotoredox^6^ and electron-donor–acceptor (EDA) methods^7^ (Scheme 1A) have been employed using different aryl radical precursors, such as aryl halides, aminated arenes, or aryl sulfonates through homolytic cleavage of C(sp^2^)–heteroatom bonds.^8^ In addition, two examples of light induced direct C(sp^2^)–H phosphonation have been reported in the same year. One of them, developed by König functionalizes electron-rich arenes by means of a ruthenium photocatalyst in combination with ammonium persulfate as sacrificial oxidant,^9^ while the second reported by Lei group uses a dual catalytic system composed of acridinium photosensitiser and a Co co-catalyst.^6*c*^ Indeed, the development of direct C(sp^2^)–H phosphonation methods are highly desirable, and although these latter synthetic methods are efficient, their environmental footprint is far from desirable. The use of transition metals, organic dyes, or excess sacrificial agents remain challenges towards a more sustainable green synthesis of these medicinally relevant backbones.

**Scheme 1:**
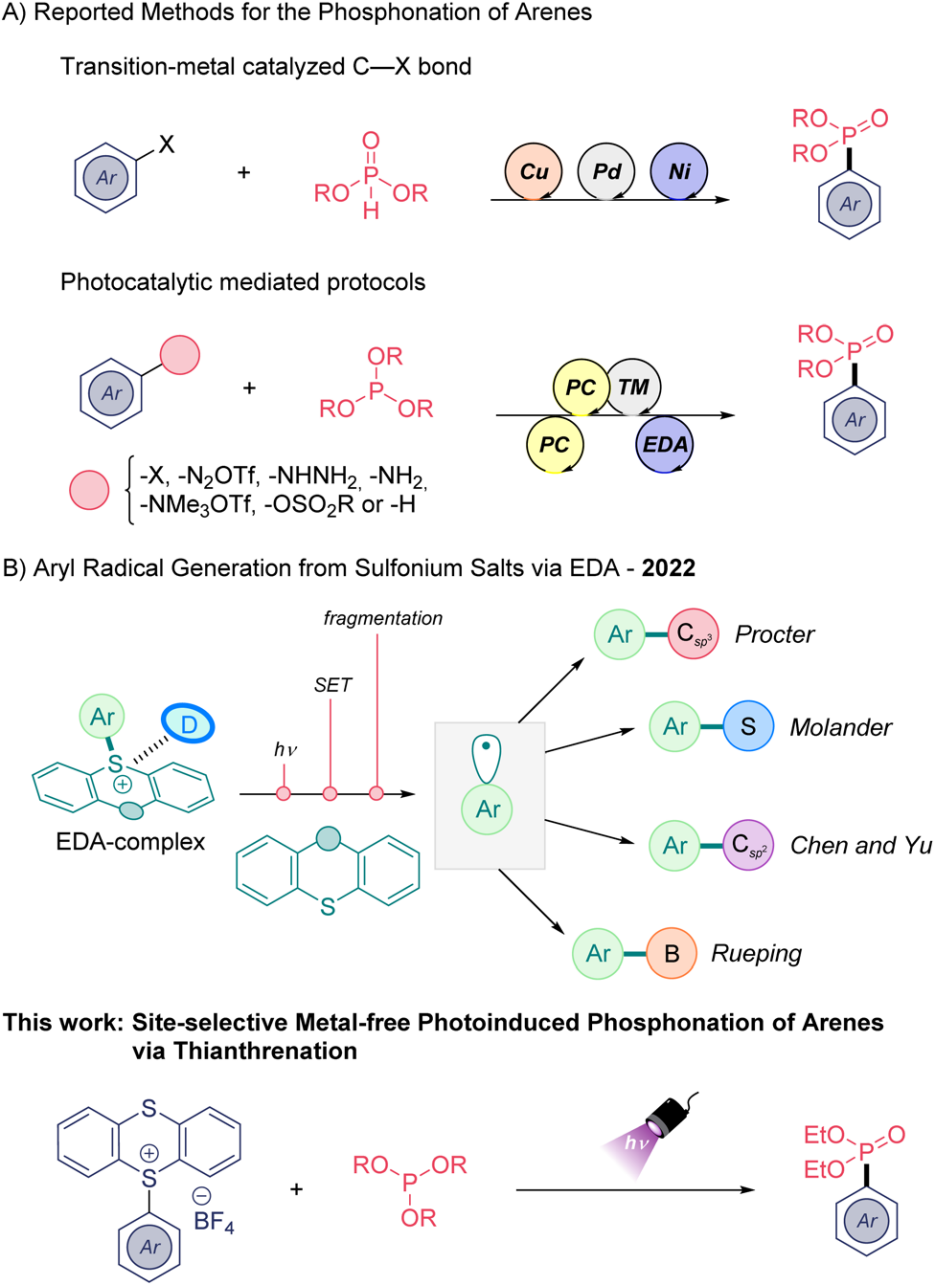
Top: (A) strategies for the preparation of aryl phosphonates. (B) Precedents using sulfonium salts as electron-acceptors in EDA complex strategies. Bottom: this work.

The photoactivation of electron donor–acceptor (EDA) complexes has emerged as a complementary tool for the generation of carbon- and heteroatom-centered radicals avoiding the use of exogenous photocatalysts.^10^ This mild mode of reactivity to generate open-shell species is very attractive from an environmental point of view.

The C(sp^2^)–H thianthrenation of arenes provides high site-selective functionalization avoiding the use of directing groups, yielding the so-called thianthrenium salts (TTS).^11^ Ritter and co-workers have exploited the aryl radical generation of TTS *via* metallaphotoredox catalysis.^12^ During the last year, different research groups have shown the potential of TTS to generate new molecular aggregates in the ground state in combination with suitable electron-donor molecules (Scheme 1B).^13^ Appropriate photoirradiation of these new molecular aggregates triggers a single electron transfer (SET) event yielding a radical ion pair. Subsequently, it undergoes an irreversible homolytic fragmentation to deliver thianthrene (TT) and the high-energy aryl radical species, which can be engaged in various transformations (Scheme 1B) including C(sp^2^)–B/C/S bond-forming reactions. The formation of C(sp^2^)–P bonds through this mode of reactivity is less known.^14^

Herein, we extend the range of electron-donor substrates in sulfonium salt EDA complex chemistry, using an organophosphorus reagent as electron-donor for the first time. A high site-selective C–H phosphonation reaction of arenes proceeding through the formation of TTS as key intermediates and using phosphites as electron-donor molecules is presented (Scheme 1, bottom). The generation of a reactive aryl radical intermediate in a regulated fashion collapses with the phosphite affording the desired aryl phosphonate and recovering the TT, which can be recycled. While preparing the submission of this manuscript, the Yang group presented the first C(sp^2^)–P bonds synthesis from TTS and a diamine external electron-donor.^15^ Interestingly, this work is highly complementary to our approach, relying on the formation of a different EDA complex and providing a distinct mechanistic pathway.

We began the optimization studies of this C(sp^2^)–P bond formation by selecting TTS 1a and triethylphosphite 2a as model substrates (Table 1). We were delighted to observe a 65% yield using dimethylacetamide (DMA) as solvent under 390 nm Kessil® overnight light irradiation (Table 1, entry 1). The use of an organic or inorganic salt provided better reactivity (Table 1, entries 2 and 3). We explored further the use of other more volatile solvents such as acetone, acetonitrile (MeCN) and dichloromethane (DCM), and they also proved to be competent solvents for this transformation (Table 1, entries 4–6). Then, we selected acetonitrile as solvent, which in combination with KHCO_3_ provided 3 in excellent yield (Table 1, entry 9). The necessity of light irradiation was confirmed because we detected only traces of product when conducting this transformation under dark conditions (Table 1, entry 10). Finally, we could decrease both the equivalents of 2a and the reaction time, observing same yields after only 30 minutes of illumination (Table 1, entry 13). The use of other wavelengths also proved to be suitable for this transformation, although it required longer reaction times. Finally, the use of non-dried solvent provided worst results. The presence of traces of water in the reaction mixture decreases the efficiency of this transformation due to the generation of highly reactive aryl radical intermediates as well as the presence of the moisture sensitive phosphite. Thus, anhydrous solvent is required to synthesize this aryl phosphonates in high yield.

**Table 1:** Optimization of the reaction conditions^a^

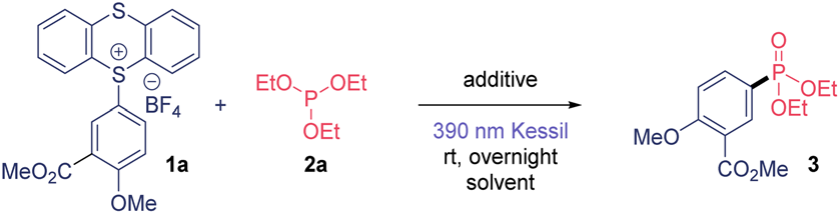					
Entry	Equiv. 2a	Solvent	Additive	Light	Yield^b^ (%)
1	10	DMA	None	390 nm	65
2	10	DMA	K_2_CO_3_	390 nm	82 (75)^c^
3	10	DMA	DABCO	390 nm	80
4	10	Acetone	K_2_CO_3_	390 nm	81
5	10	MeCN	K_2_CO_3_	390 nm	88
6	10	DCM	K_2_CO_3_	390 nm	85
7	10	MeCN	K_2_CO_3_	390 nm	87
8	10	MeCN	K_3_PO_4_	390 nm	84
9	10	MeCN	KHCO_3_	390 nm	90
10	10	MeCN	KHCO_3_	Dark	<10
11	5	MeCN	KHCO_3_	390 nm	89
12	2.5	MeCN	KHCO_3_	390 nm	75
**13**	**5**	**MeCN**	**KHCO**_**3**_	**390 nm**	**87** d
14	5	MeCN	KHCO_3_	456 nm	88
15	5	Wet MeCN	KHCO_3_	390 nm	53^d^

a Reaction conditions: 0.1 mmol of TT salt 1a, 0.1 mmol of the corresponding additive, indicated amounts of phosphite 2a and 0.1 M in the indicated dry solvent.

b Determined by ^1^H NMR using 1,3,5-trimethoxybenzene as internal standard.

c Isolated yield from 0.5 mmol of 1a.

d 30 minutes reaction time.

With suitable reaction conditions in hand, the scope of the presented photoinduced reaction was investigated (Table 2). First, we used triethylphosphite as organophosphorus coupling partner. A range of 2-substituted anisoles were amenable to this transformation, obtaining 2-nitrile (4), 2-halogenated (5 and 6) and 2-aldehyde (7) derivatives in high yields. Arenes containing alkylated substitutions were also competent substrates (8–10), yielding the desired aryl phosphonates from moderate to good yields. Biphenyls were used as coupling partners with success including biphenyl (11) and 4-bromophenyl (12), which the presence of the bromide allows subsequent post-functionalization. Notably, the phosphonate derivatives of the anti-inflammatories Fenbufen and Flurbiprofen were isolated in 74 and 88%, respectively. In general, the method also tolerates aryl amides, obtaining the bifunctional molecules 15 and 16 in good yields. The phosphonate derivative from the insecticide Xanthone was also isolated in a 65% yield (17). A wide range of ether-substituted arenes were tested under the reaction conditions (18–21) from good to excellent yields, including relevant bioactive molecules such Gemfibrozil (65%) or Clofibrate (76%). Of note, we could extend this method to electron-deficient monohalogenated arenes, in contrast to other photochemical C(sp^2^)–H phosphonations.^6*c*,9^ Thus, fluoro- (22) and chlorobenzene (23) derivatives were isolated in outstanding yields (95 and 90%, respectively). Additionally, 24 was prepared in excellent yield. Lastly, our method not only works for arenes, but also heteroaromatic 25 was synthesized in good yield.

**Table 2:** Substrate scope evaluation^a^

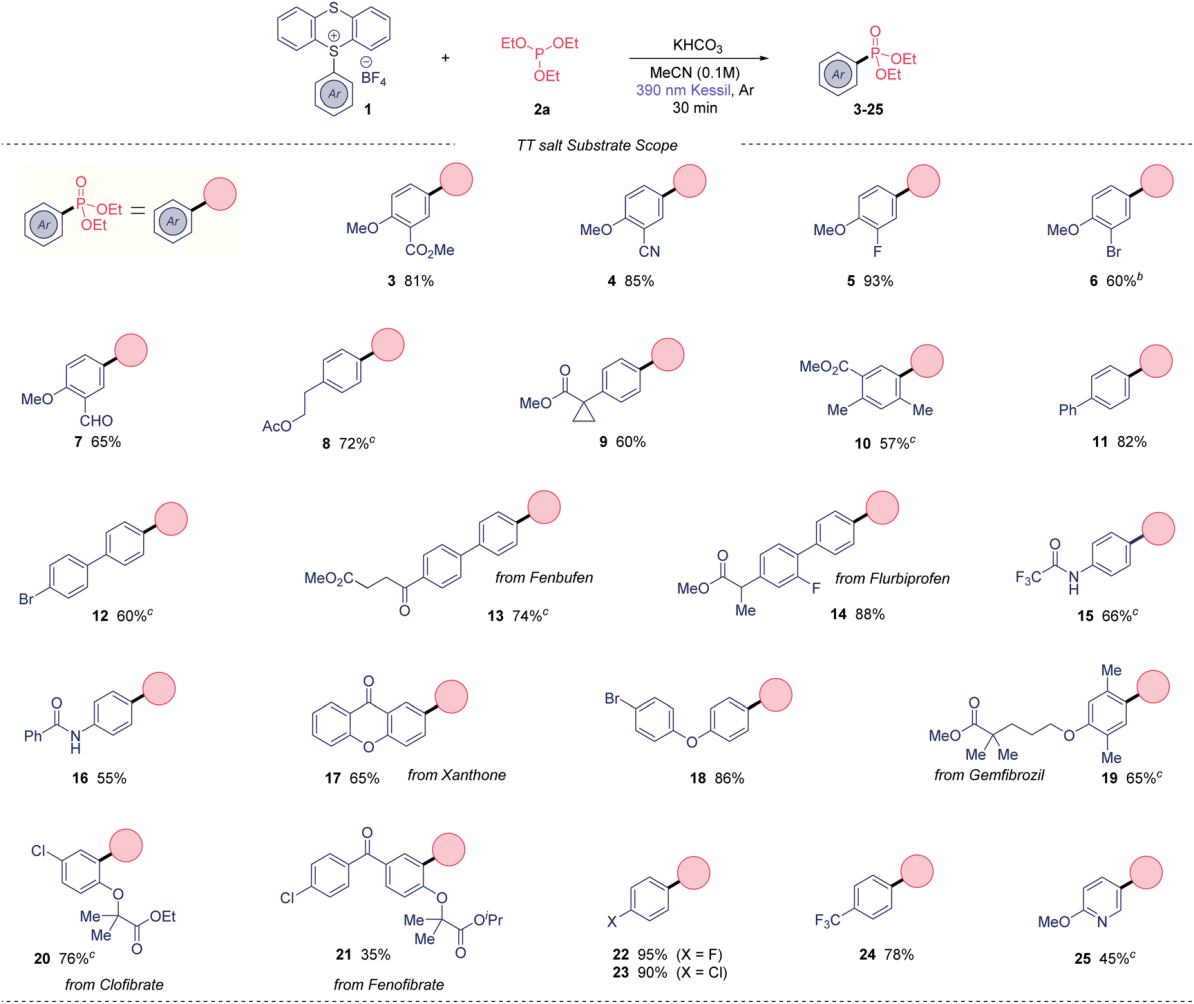

a Reaction conditions: 0.5 mmol of the corresponding TT salt, 2.5 mmol of 2a, 0.5 mmol of KHCO_3_ in MeCN (0.1 M) under 390 nm Kessil® irradiation for 30 minutes.

b 2.5 equiv. of 2a used.

c Light irradiation for 12 h.

Overall, this method presents high functional group tolerance, and it allows to carry halides through the photochemical process, in contrast to previously reported methods.^5–7^ Importantly, the thianthrene unit beyond acting as a leaving group, it is responsible to form the EDA complex with the phosphite as electron donor molecule.

Of note, we could also recover the TT leaving group in high purity in all cases in excellent yield (85–90%). Next, we moved our attention to the phosphites substrate scope (Table 3). Trimethyl phosphite also gave excellent yield (26) when using our model TT salt 1a, as well as the bulkier triisopropyl phosphite, which provide the desired phosphonate 27 in 75% yield. Notably, the use of triphenylphosphite also was amenable to this transformation, although the reaction required longer time to be completed.

**Table 3:** Substrate scope using TT salt 1a and different phosphites^a^

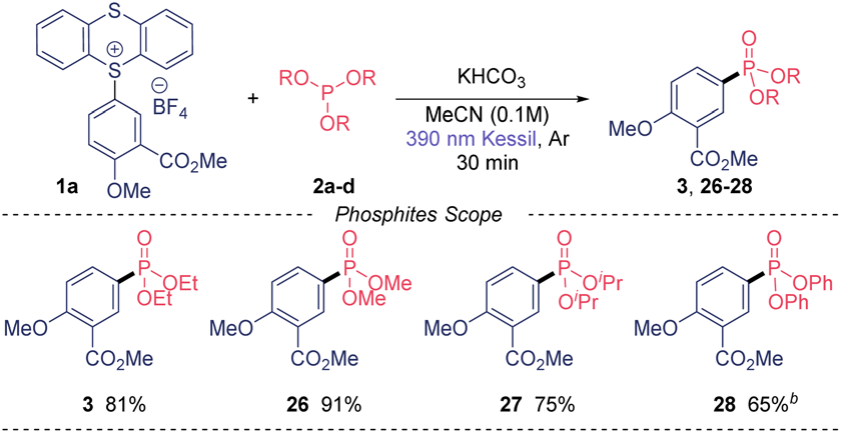

a Reaction conditions: 0.5 mmol of TT salt 1a, 2.5 mmol of the corresponding phosphite, 0.5 mmol of KHCO_3_ in MeCN (0.1 M) under 390 nm Kessil® irradiation for 30 minutes.

b Overnight irradiation.

Because of the efficiency of the developed photochemical process, we further examined the adaptability of this transformation when natural sunlight was used as illumination source (Scheme 2A). To our delight, we isolated compound 4 in 82% yield during only 2 hours of sunlight exposure (see ESI† for details). This experiment highlights the practicality of this transformation. Additionally, we prepared fluorinated phosphonate 22 in an excellent 79% yield when using a solar simulator as light source (see Scheme 2B and ESI†). Finally, to highlight the usefulness of this synthetic approach we studied the direct C–H phosphonation sequence for the provision of bifunctional aryl phosphonate 18, directly from unfunctionalized 4-bromophenoxybenzene (Scheme 2C). This telescoped synthesis was also accomplished in large scale (from 5 mmol), and we were delighted to isolate 18 in an excellent 84% yield. Importantly, we did not detect any side-product when scaling-up the reaction. The TT was recovered as well in an 88%, which makes this protocol sustainable with minimum waste.

**Scheme 2:**
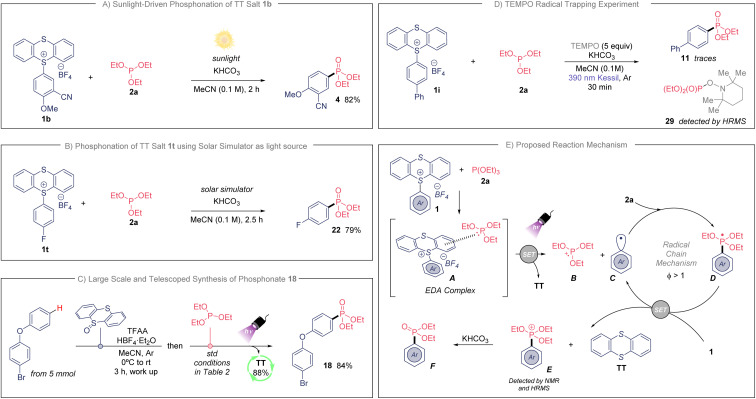
(A) Light-promoted synthesis of 4 under standard conditions from natural sunlight. (B) Photoinduced preparation of 22 using the standard conditions and a solar light simulator. (C) Large and telescoped synthesis of 18, see ESI† for additional details. (D) TEMPO radical trapping experiment. (E) Proposed reaction mechanism.

Next, we sought to study the operative mechanism of this transformation. First, the analysis by UV/vis absorption of the individuals and mixtures of the reaction components in MeCN showed that TTS 1a meets 2a in a new molecular aggregate (EDA complex, Scheme 2E). TTS 1a and reagent 2a present an absorption band near ultraviolet range, while the mixture of both exhibits a bathochromic shift within the visible-light absorption (see ESI†). These changes are apparent to eye since the independent solutions of 1a and 2a stay colorless, while the mixture of both changed to a slightly pale-yellow color (see ESI†). Secondly, the addition of TEMPO (2,2,6,6-tetramethyl-1-piperidinyloxy) under the standard reaction conditions totally inhibited the preparation of the phosphonated arene (Scheme 2D), detecting unreacted 1i. Additionally, the detection of the TEMPO adduct 29 indicates the involvement of the phosphite as electron-donor molecule. Moreover, the photochemical quantum yield (*Φ*) of this reaction was measured (*Φ* = 118, see ESI†). This high value indicates that this mechanism operates through an efficient radical chain pathway.^16^

With the experimental evidences described above, a plausible mechanism for the phosphonation of arenes is shown in Scheme 2E. The association of TTS with the phosphite involves the formation of a new EDA complex intermediate that induces a radical chain. The newly generated EDA complex A undergoes single electron transfer (SET) upon irradiation, providing the cationic radical species B, thianthrene (TT) and the highly transient aryl radical C. The generated radical C reacts with the corresponding phosphite 2a*via* radical addition affording the radical intermediate D. Next, we propose a SET event between radical D (*E*^0^_ox_ ≃ −1.80 V *vs.* SCE)^5*g*^ and TT salt 1 (*E*^0^_red_ ≃ −1.05 V *vs.* SCE for 1b),^17^ producing the aryl radical C, which enters in the chain cycle, as well as TT and the phosphonium intermediate E. Of note, the proposed intermediate E was confirmed by bidimensional ^1^H/^31^P HMBC NMR, as well as by HRMS when the reaction was done in the absence of KHCO_3_ (see ESI† for details). Further, the phosphonium E follows an ionic Arbuzov-like pathway providing the desired aryl phosphonate.

## Conclusions

We have reported a practical, sustainable, and scalable C(sp^2^)–H phosphonation of arenes avoiding the use of directing groups, stoichiometric oxidants, or harsh conditions. This transformation is enabled by the generation of thianthrenium salts as key intermediates, and it is completed within minutes using phosphites as electron-donors. The thianthrene unit beyond simply acting as a leaving group, it serves as the electron-accepting component regardless of the electronic nature of the rest of the electron-acceptor substrate. Moreover, the TT unit could be recovered in all the reactions in both high purity and yield, which can be reused for more TTS production.^11,18^ Simple electron-rich and electron-deficient arenes were phosphonated from moderate to excellent yields, as well as complex biomolecules. The presented method could serve as an efficient tool for late-stage phosphonation. Mechanistic investigations are in agreement with the formation of an intermediate EDA complex that induces an efficient radical chain mechanism.

## Author contributions

Albert Gallego-Gamo and Albert Granados optimized the reaction conditions. Albert Gallego-Gamo, David Reyes-Mesa, Axel Guinart-Guillem and Albert Granados explored the scope, and the mechanism of the reaction. Albert Granados, Carolina Gimbert-Suriñach and Adelina Vallribera supervised the project. Roser Pleixats, Carolina Gimbert-Suriñach and Adelina Vallribera acquired financial support for the development of this project. The manuscript was written by Albert Gallego, Roser Pleixats, Carolina Gimbert-Suriñach, Adelina Vallribera and Albert Granados.

## Conflicts of interest

There are no conflicts to declare.

## Supplementary Material

RA-013-D3RA04512A-s001
