# Supplementary material for: Site-selective and metal-free C–H phosphonation of arenes via photoactivation of thianthrenium salts
Source: RSC Adv. 2023 Aug 8;13(33):23359–64. doi: 10.1039/d3ra04512a (PMC10407877; doi:10.1039/d3ra04512a)
Supplement: RA-013-D3RA04512A-s001 [file RA-013-D3RA04512A-s001.pdf]

Electronic Supplementary Material (ESI) for RSC Advances.
This journal is © The Royal Society of Chemistry 2023

Supporting Information

Site-Selective and Metal-free C–H Phosphonation of Arenes via Photoactivation of Thianthrenium Salts

Albert Gallego-Gamo, David Reyes-Mesa,‡ Axel Guinart-Guillem,‡ Roser Pleixats, Carolina Gimbert-Suriñach,*Adelina Vallribera,* and Albert Granados*

Departament de Química and Centro de Innovación en Química Avanzada (ORFEO-CINQA), Universitat Autònoma de Barcelona, Cerdanyola del Vallès, 08193 Barcelona, Spain

*To whom correspondence should be addressed.

carolina.gimbert@uab.cat adelina.vallribera@uab.es albert.granados@uab.es

TABLE OF CONTENT

1. General Considerations 2
2. List of Used Thianthrenium Salts 3
3. Synthesis of Aryl Phosphonates: Reaction Workflow, Optimization and Compound Characterization... 4
4. Sunlight-driven synthesis of **4** 19
5. Photochemical Preparation of **22** Using a Solar Simulator 20
6. Large scale and Telescoped Synthesis of **18** 20
7. Mechanistic Investigation 22
8. Unsuccessful Substrates 31
9. NMR Spectra 32

1. General Considerations

1.1 General

All chemical transformations requiring inert atmosphere were done using Schlenk line techniques with a 4- or 5-port dual-bank manifold. For purple or blue light irradiation, a Kessil PR160-purple LED lamp (30 W High Luminous DEX 2100 LED, λ_{max} = 390 nm) or a Kessil A160WE Blue LED lamps (40 W, λ_{max} = 456 nm) were placed 4 cm away from the reaction vials. Photoredox-catalyzed reactions were performed using 4 or 8 mL Chemglass vials (15-425 Green Open Top Cap, TFE Septa). Reactions were monitored by TLC or NMR. TLC analysis was performed using hexanes/EtOAc as the eluent and visualized using phosphomolybdic acid, ninhydrin, *p*-anisaldehyde stain, and/or UV light. The UV-vis spectra were recorded in a UV-Vis spectrophotometer HP 8453 (*Servei d'Anàlisi Química*, UAB), at room temperature, with the appropriate solvent. The cyclic voltammetry experiments were performed in a BioLogic® SP-50 Single Channel Potentiostat, in one-compartment three-electrode set-up using glassy carbon disk as working electrode (ø = 3 mm), platinum wire as auxiliary electrode and SCE. Experiments were performed at room temperature, using the appropriate solvent, degassing with Ar, using $TBAPF_6$ as supporting electrolyte (0.1 M). All the experiments were referred to ferrocene as internal standard. The NMR experiments (^{1}H, ^{13}C, ^{19}F {^{1}H decoupled}, ^{31}P {^{1}H decoupled}, ^{1}H/^{31}P HMBC) were performed in the *Servei de Ressonància Magnètica Nuclear*, UAB, using NEO 300, NEO 400 or III 400SB or NEO 500. Chemical shifts are referenced to residual, nondeuterated $CHCl_3$ (δ 7.26 in ^{1}H NMR and 77.3 in ^{13}C NMR). The HRMS (ESI+) and elemental analyses were done by the *Servei d'Anàlisi Química,* UAB. HRMS was done using a Bruker micrOTOF-QII mass spectrometer (fly time analyzer) through positive electrospray ionization. IR spectra were recorded on an FT-IR using either neat oil or solid products. Melting points (°C) are uncorrected.

1.2 Chemicals

Deuterated NMR solvents were purchased from Euroisotop. Dry acetone, DMA, MeCN were obtained from Aldrich and used as received. DCM were purchased and dried *via* a solvent delivery system. Bulk solvents were purchased from VWR. Thianthrene-*S*-oxide was prepared as reported by the Ritter group and arenes and phosphites were purchased from commercial suppliers and used as received.

2. List of Used Thianthrenium Salts

Thianthrenium salts (TT salts) **1a-s** and **1w** were prepared as previously reported by the Ritter group.[1] **1t** and **1u** were prepared following reported procedure from Stuart and co-workers.[2] **1v** was prepared from the corresponding arylboronic acid as described by Guo and Wang.[3]

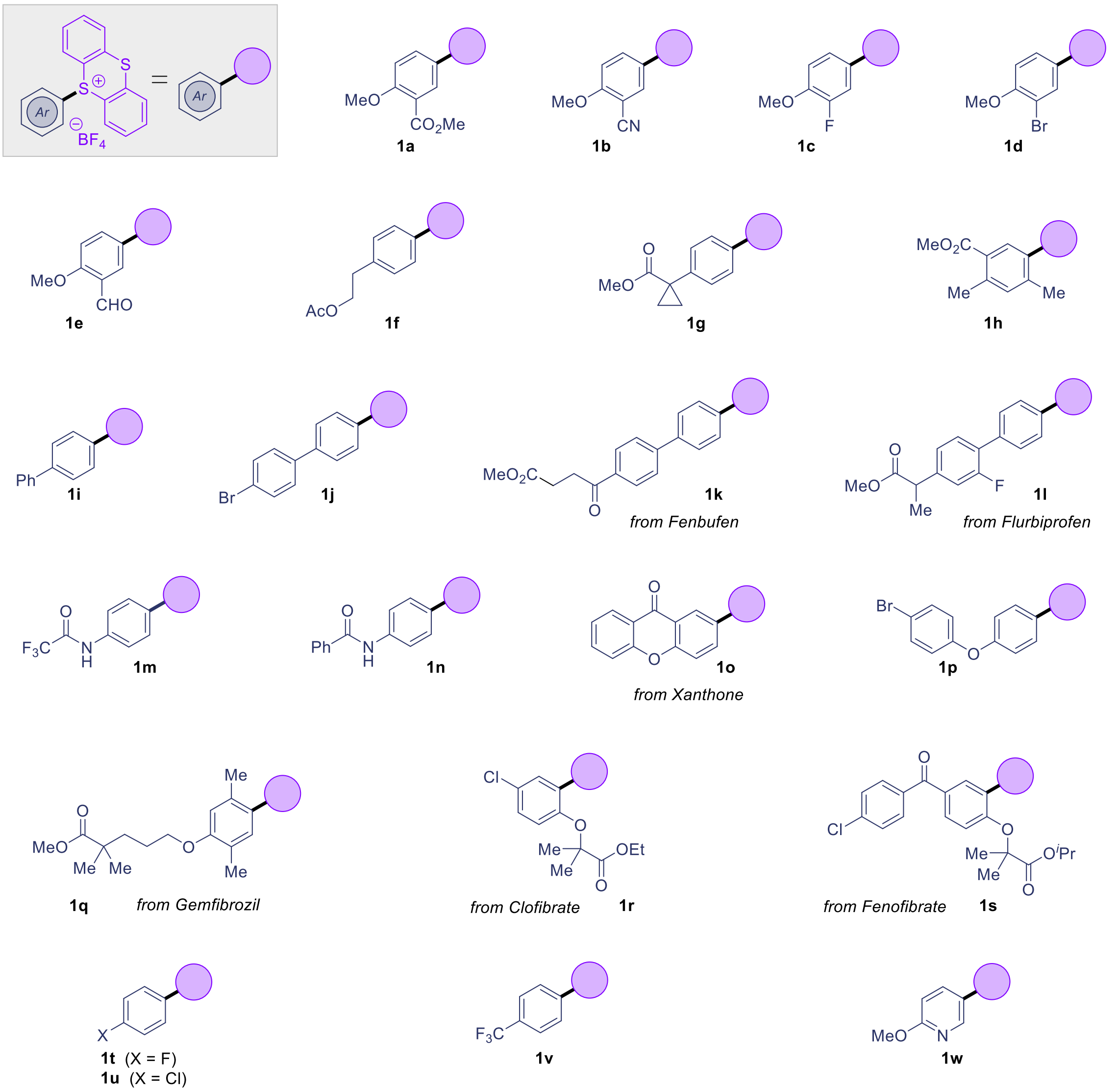

[1] a) F. Berger, M. B. Plutschack, J. Riegger, W. Yu, S. Speicher, M. Ho, N. Frank, T. Ritter, *Nature* 2019, **567**, 223–228. b) R. Sang, S. E. Korkis, W. Su, F. Ye, P. S. Engl, F. Berger, T. Ritter, *Angew. Chem. Int. Ed.* 2019, **58**, 16161–16166. c) J. Li, J. Chen, R. Sang, W. S. Ham, M. B. Plutschack, F. Berger, S. Chabbra, A. Schnegg, C. Genicot, T. Ritter, *Nat. Chem.* 2020, **12**, 56–62.

[2] R. A. Roberts, B. E. Metze, A. Nilova, D. R. Stuart, *J. Am. Chem. Soc.* 2023, **145**, 3306-3311.

[3] X.-Y. Chen, Y.-N. Li, Y. Wu, J. Bai, Y. Guo, P. Wang, *J. Am. Chem. Soc.* 2023, **145**, 10431-10440.

3. Synthesis of Aryl Phosphonates: Reaction Workflow, Optimization and Compound Characterization

3.1. Reaction Workflow

All photoinduced reactions were done using a Kessil PR160-purple LED lamp (30 W High Luminous DEX 2100 LED, λ_{max} = 390 nm). The LED was placed 4 cm away from the reaction vial within a ventilated fume hood and using a fan to maintain the temperature approximately at 25ºC.

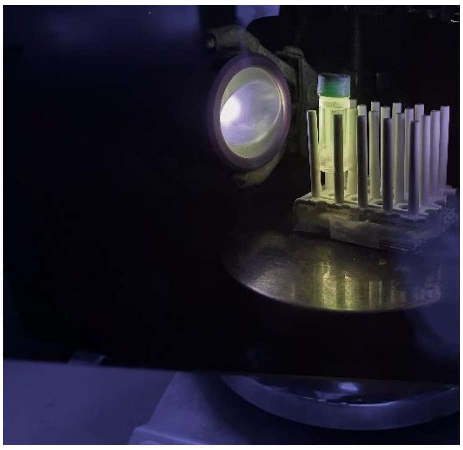

Figure S1. Reaction setup for the photoinduced phosphonation of arenes

3.3. General Procedure for the Photochemical Synthesis of Aryl Phosphonates

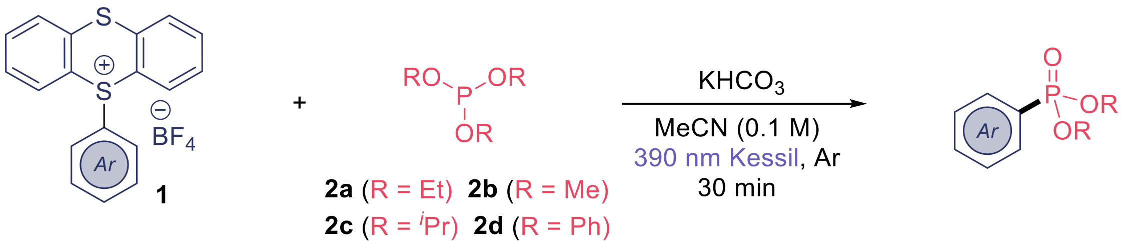

To a flame-dried 8 mL vial equipped with a magnetic stir bar, TT salt **1** (0.5 mmol, 1.0 equiv) and $KHCO_3$ (50 mg, 0.5 mmol, 1.0 equiv) were added, and the vial was subjected to 3 cycles of vacuum/argon degassing. Subsequently, 5 mL of dry MeCN were added under inert atmosphere. Subsequently, the corresponding phosphite was added via syringe (2.5 mmol, 5 equiv), and the solution was degassed with Argon for 30 seconds. The reaction mixture was irradiated for 30 minutes (unless otherwise noted) with a 390 nm Kessil PR160-purple LED as described in the "*Workflow*" section. The temperature of the reaction was maintained at approximately 25 °C via a fan. Upon completion, the solvent was removed under reduced pressure. The crude mixture was subjected to flash column purification using hexanes/EtOAc mixtures to yield the desired aryl phosphonate.

3.3. Compound Characterization Data

Methyl 5-(Diethoxyphosphoryl)-2-methoxybenzoate (3)

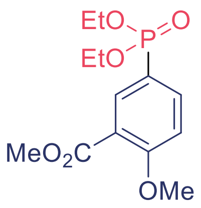

Prepared according to the *General Procedure* from the corresponding thianthrenium salt **1a** (234 mg, 0.50 mmol, 1.0 equiv) and **2a** (430 µL, 415 mg, 2.5 mmol, 5.0 equiv). After purification by flash column chromatography (hexane:EtOAc 1:1), the title compound **3** was obtained as a colorless oil (109 mg, 0.41 mmol, 81%). $\mathbf{R_f}$ = 0.32 (silica gel, *n*-hexane / EtOAc, 1:2 (v/v)). **^{1}H NMR** (300 MHz, $CDCl_3$), δ (ppm) = 8.21 (dd, *J* = 13.3, 2.0 Hz, 1H), 7.91 (ddd, *J* = 12.6, 8.6, 2.1 Hz, 1H), 7.05 (dd, *J* = 8.7, 3.3 Hz, 1H), 4.23 – 4.02 (m, 4H), 3.95 (s, 3H), 3.89 (s, 3H), 1.31 (t, *J* = 7.1 Hz, 6H). **^{13}C{^{1}H} NMR** (101 MHz, $CDCl_3$), δ (ppm) = 165.7, 162.0, 137.3 (d, *J* = 11.2 Hz), 135.6 (d, *J* = 12.4 Hz), 120.3, 119.7 (d, *J* = 196.9 Hz), 111.9 (d, *J* = 15.8 Hz), 62.1 (d, *J* = 5.4 Hz, 2C), 56.2, 52.2, 16.4, 16.3. **^{31}P{^{1}H} NMR** (162 MHz, $CDCl_3$), δ (ppm) = 18.0. **FT-IR** (cm^{-1}, neat, ATR), ṽ = 2983, 2953, 1733, 1573, 1496, 1438, 1241, 1139, 1016. **HRMS (ESI)** calcd for $C_{13}H_{19}O_6PNa$ $[M+Na]^+$: 325.0811, found 325.0809.

Diethyl (3-Cyano-4-methoxyphenyl)phosphonate (4)

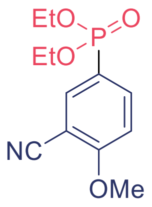

Prepared according to the *General Procedure* from the corresponding thianthrenium salt **1b** (218 mg, 0.50 mmol, 1.0 equiv) and **2a** (430 µL, 415 mg, 2.5 mmol, 5.0 equiv). After purification by flash column chromatography (hexane:EtOAc 1:1), the title compound **4** was obtained as a yellowish oil (114 mg, 0.42 mmol, 85%). $\mathbf{R_f}$ = 0.23 (silica gel, *n*-hexane / EtOAc, 1:2 (v/v)). **^{1}H NMR** (300 MHz, $CDCl_3$), δ (ppm) = 8.22 – 7.86 (m, 2H), 7.07 (dd, *J* = 9.3, 3.0 Hz, 1H), 4.23 – 4.05 (m, 4H), 4.01 (s, 3H), 1.34 (t, *J* = 7.1 Hz, 6H). **^{13}C{^{1}H} NMR** (75 MHz, $CDCl_3$), δ (ppm) = 163.8 (d, *J* = 3.3 Hz), 138.1 (d, *J* = 10.8 Hz), 137.6 (d, *J* = 11.8 Hz), 121.3 (d, *J* = 197.3 Hz), 115.3, 111.4 (d, *J* = 15.8 Hz), 102.6 (d, *J* = 18.0 Hz), 62.5 (d, *J* = 5.5 Hz, 2C), 56.4, 16.4, 16.3. **^{31}P{^{1}H} NMR** (122 MHz, $CDCl_3$), δ (ppm) = 16.0. **FT-IR** (cm^{-1}, neat, ATR), ṽ = 2982, 2907, 2230, 1599, 1496, 1245, 1047, 1016, 964. **HRMS (ESI)** calcd for $C_{12}H_{17}O_4P$ $[M+H]^+$: 270.0889, found 270.0880.

Diethyl (3-Fluoro-4-methoxyphenyl)phosphonate (5)

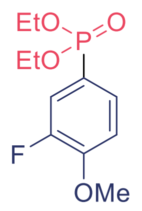

Prepared according to the *General Procedure* from the corresponding thianthrenium salt **1c** (214 mg, 0.50 mmol, 1.0 equiv) and **2a** (430 µL, 415 mg, 2.5 mmol, 5.0 equiv). After purification by flash column chromatography (hexane:EtOAc 1:1), the title compound **5** was obtained as a yellowish oil (107 mg, 0.46 mmol, 93%). R_f = 0.15 (silica gel, *n*-hexane / EtOAc, 1:1 (v/v)). **^{1}H NMR** (300 MHz, $CDCl_3$), δ (ppm) = 7.52 (dddd, *J* = 13.2, 8.3, 1.8, 1.1 Hz, 1H), 7.42 (ddd, *J* = 13.6, 10.9, 1.8 Hz, 1H), 6.98 (td, *J* = 8.2, 4.4 Hz, 1H), 4.19 – 3.95 (m, 4H), 3.88 (s, 3H), 1.26 (t, *J* = 7.1 Hz, 6H). **^{13}C{^{1}H} NMR** (75 MHz, $CDCl_3$), δ (ppm) = 151.9 (dd, *J* = 249.1, 21.8 Hz), 151.2 (dd, *J* = 10.4, 3.3 Hz), 129.0 (dd, *J* = 10.1, 3.9 Hz), 121.7 (d, *J* = 5.2 Hz), 119.1 (dd, *J* = 19.1, 11.6 Hz), 113.0 (dd, *J* = 18.3, 1.9 Hz), 62.1 (d, *J* = 5.4 Hz, 2C), 56.1, 16.4, 16.3. **^{19}F{^{1}H} NMR** (282 MHz, $CDCl_3$), δ (ppm) = -134.2. **^{31}P{^{1}H} NMR** (162 MHz, $CDCl_3$), δ (ppm) = 17.5. **FT-IR** (cm^{-1}, neat, ATR), ṽ = 2917, 1849, 1610, 1513, 1443, 1259, 1243, 1015. **HRMS (ESI)** calcd for $C_{11}H_{16}FO_4PNa$ $[M+Na]^+$: 285.0662, found 285.0664.

Diethyl (3-Bromo-4-methoxyphenyl)phosphonate (6)

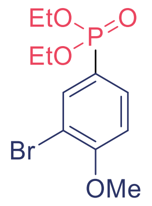

Prepared according to the *General Procedure* from the corresponding thianthrenium salt **1d** (245 mg, 0.50 mmol, 1.0 equiv) and **2a** (215 µL, 207 mg, 1.25 mmol, 2.5 equiv). After purification by flash column chromatography (hexane:EtOAc 1:1), the title compound **6** was obtained as a tan oil (96 mg, 0.30 mmol, 60%). R_f = 0.20 (silica gel, *n*-hexane / EtOAc, 1:1 (v/v)). **^{1}H NMR** (400 MHz, $CDCl_3$), δ (ppm) = 7.94 (dd, *J* = 13.0, 1.9 Hz, 1H), 7.73 (ddd, *J* = 12.9, 8.4, 1.9 Hz, 1H), 6.94 (dd, *J* = 8.4, 3.9 Hz, 1H), 4.20 – 3.98 (m, 4H), 3.93 (s, 3H), 1.31 (t, *J* = 7.1 Hz, 6H). **^{13}C{^{1}H} NMR** (101 MHz, $CDCl_3$), δ (ppm) = 159.1 (d, *J* = 3.2 Hz), 136.8 (d, *J* = 11.8 Hz), 133.0 (d, *J* = 10.6 Hz), 121.6 (d, *J* = 194.4 Hz), 112.2 (d, *J* = 20.3 Hz), 111.7 (d, *J* = 17.0 Hz), 62.3 (d, *J* = 5.4 Hz, 2C), 56.5, 16.4, 16.3. **^{31}P{^{1}H} NMR** (162 MHz, $CDCl_3$), δ (ppm) = 17.3. **FT-IR** (cm^{-1}, neat, ATR), ṽ = 2980, 2904, 1589, 1493, 1237, 1131, 1046, 1013, 960. **HRMS (ESI)** calcd for $C_{11}H_{16}BrO_4PNa$ $[M+Na]^+$: 344.9861, found 344.9861.

Diethyl (3-Formyl-4-methoxyphenyl)phosphonate (7)

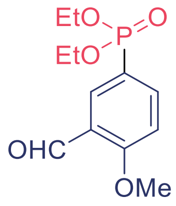

Prepared according to the *General Procedure* from the corresponding thianthrenium salt **1e** (219 mg, 0.50 mmol, 1.0 equiv) and **2a** (430 µL, 415 mg, 2.5 mmol, 5.0 equiv). After purification by flash column chromatography (hexane:EtOAc 1:1), the title compound **7** was obtained as a tan oil (88 mg, 0.33 mmol, 65%). R_f = 0.38 (silica gel, *n*-hexane / EtOAc, 1:1 (v/v)). $\mathbf{R_f}$ = 0.38 (silica gel, *n*-hexane / EtOAc, 1:1 (v/v)). **^{1}H NMR** (400 MHz, $CDCl_3$), δ (ppm) = 10.40 (s, 1H), 8.17 (dd, *J* = 13.2, 2.1 Hz, 1H), 8.04 – 7.91 (m, 1H), 7.05 (dd, *J* = 8.6, 3.0 Hz, 1H), 4.19 – 3.99 (m, 4H), 3.94 (s, 3H), 1.26 (t, *J* = 7.0 Hz, 6H). **^{13}C{^{1}H} NMR** (101 MHz, $CDCl_3$), δ (ppm) = 188.8, 164.3 (d, *J* = 3.4 Hz), 139.5 (d, *J* = 11.9 Hz), 132.6 (d, *J* = 11.4 Hz), 124.6 (d, *J* = 14.4 Hz), 120.6 (d, *J* = 196.5 Hz), 111.9 (d, *J* = 15.3 Hz), 62.3 (d, *J* = 5.6 Hz, 2C), 56.0, 16.4, 16.3. **^{31}P{^{1}H} NMR** (162 MHz, $CDCl_3$), δ (ppm) = 17.6. **FT-IR** (cm^{-1}, neat, ATR), ṽ = 2984, 2943, 1685, 1600, 1573, 1462, 1283, 1251, 1015, 960. **HRMS (ESI)** calcd for $C_{12}H_{18}O_5P$ $[M+H]^+$: 273.0886, found 273.0889.

4-(Diethoxyphosphoryl)phenethyl Acetate (8)

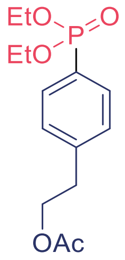

Prepared according to the *General Procedure* from the corresponding thianthrenium salt **1f** (233 mg, 0.50 mmol, 1.0 equiv), **2a** (430 µL, 415 mg, 2.5 mmol, 5.0 equiv) and overnight irradiation. After purification by flash column chromatography (hexane:EtOAc:EtOH 1:1:0.005) in alumina, the title compound **8** was obtained as a colorless oil (108 mg, 0.36 mmol, 72%). $\mathbf{R_f}$ = 0.35 (alumina, *n*-hexane / EtOAc / EtOH, 1:1:0.005 (v/v/v)). **^{1}H NMR** (400 MHz, $CDCl_3$), δ (ppm) = 7.69 (dd, *J* = 13.2, 8.2 Hz, 2H), 7.27 (dd, *J* = 8.0, 3.7 Hz, 2H), 4.23 (t, *J* = 6.9 Hz, 2H), 4.14 – 3.97 (m, 4H), 2.93 (t, *J* = 6.9 Hz, 2H), 1.97 (s, 3H), 1.26 (t, *J* = 7.1 Hz, 6H). **^{13}C{^{1}H} NMR** (101 MHz, $CDCl_3$), δ (ppm) = 170.8, 142.7 (d, *J* = 3.3 Hz), 131.9 (d, *J* = 10.3 Hz, 2C), 129.0 (d, *J* = 15.3 Hz, 2C), 126.4 (d, *J* = 189.8 Hz), 64.2, 62.0 (d, *J* = 5.4 Hz, 2C), 35.0, 20.8, 16.3, 16.2. **^{31}P{^{1}H} NMR** (162 MHz, $CDCl_3$), δ (ppm) = 18.9. **FT-IR** (cm^{-1}, neat, ATR), ṽ = 2982, 2932, 1738, 1607, 1367, 1235, 1019, 958. **HRMS (ESI)** calcd for $C_{14}H_{21}O_5PNa$ $[M+Na]^+$: 323.1019, found 323.1029.

Methyl 1-(4-(Diethoxyphosphoryl)phenyl)cyclopropane-1-carboxylate (9)

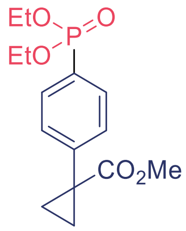

Prepared according to the *General Procedure* from the corresponding thianthrenium salt **1g** (240 mg, 0.50 mmol, 1.0 equiv) and **2a** (430 µL, 415 mg, 2.5 mmol, 5.0 equiv). After purification by flash column chromatography (hexane:EtOAc 1:1), the title compound **9** was obtained as a yellowish oil (93 mg, 0.30 mmol, 60%). $\mathbf{R_f}$ = 0.35 (silica gel, *n*-hexane / EtOAc, 1:1 (v/v)). **^{1}H NMR** (300 MHz, $CDCl_3$), δ (ppm) = 7.76 – 7.67 (m, 2H), 7.45 – 7.36 (m, 2H), 4.26 – 3.95 (m, 4H), 3.61 (s, 3H), 1.62 (dd, *J* = 6.2, 4.0 Hz, 2H), 1.31 (t, *J* = 7.1 Hz, 6H), 1.23 – 1.11 (m, 2H). **^{13}C{^{1}H} NMR** (75 MHz, $CDCl_3$), δ (ppm) = 174.3, 144.0 (d, *J* = 3.3 Hz), 131.7 (d, *J* = 10.2 Hz, 2C), 130.6 (d, *J* = 15.4 Hz, 2C), 127.1 (d, *J* = 189.4 Hz), 62.1 (d, *J* = 5.5 Hz, 2C), 52.4, 29.0, 16.6, 16.4, 16.3. **^{31}P{^{1}H} NMR** (122 MHz, $CDCl_3$), δ (ppm) = 18.7. **FT-IR** (cm^{-1}, neat, ATR), ṽ = 2919, 1725, 1299, 1169, 1021. **HRMS (ESI)** calcd for $C_{15}H_{21}O_5PNa$ $[M+Na]^+$: 335.1019, found 335.1016.

Methyl 5-(Diethoxyphosphoryl)-2,4-dimethylbenzoate (10)

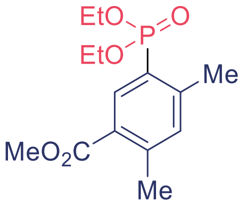

Prepared according to the *General Procedure* from the corresponding thianthrenium salt **1h** (233 mg, 0.50 mmol, 1.0 equiv), **2a** (430 µL, 415 mg, 2.5 mmol, 5.0 equiv) and overnight irradiation. After purification by flash column chromatography (hexane:EtOAc 1:1), the title compound **10** was obtained as a white solid (85 mg, 0.28 mmol, 57%). $\mathbf{R_f}$ = 0.38 (silica gel, *n*-hexane / EtOAc, 1:1 (v/v)). **M.P**: 57-61ºC. **^{1}H NMR** (300 MHz, $CDCl_3$), δ (ppm) = 8.46 (d, *J* = 15.0 Hz, 1H), 7.15 (d, *J* = 5.2 Hz, 1H), 4.20 – 4.00 (m, 4H), 3.87 (s, 3H), 2.61 (s, 3H), 2.56 (d, *J* = 1.6 Hz, 3H), 1.32 (t, *J* = 7.1 Hz, 6H). **^{13}C{^{1}H} NMR** (75 MHz, $CDCl_3$), δ (ppm) = 167.1, 145.7 (d, *J* = 10.2 Hz), 145.1, 136.6 (d, *J* = 12.3 Hz), 134.8 (d, *J* = 14.5 Hz), 126.8 (d, *J* = 15.3 Hz), 124.6 (d, *J* = 187.8 Hz), 62.0 (d, *J* = 5.6 Hz, 2C), 51.8, 21.6, 21.1 (d, *J* = 3.2 Hz), 16.3, 16.2. **^{31}P{^{1}H} NMR** (122 MHz, $CDCl_3$), δ (ppm) = 18.4. **FT-IR** (cm^{-1}, neat, ATR), ṽ = 2983, 2928, 1718, 1600, 1259, 1240, 1157, 1018, 956. **HRMS (ESI)** calcd for $C_{14}H_{21}O_5PNa$ $[M+Na]^+$: 323.1019, found 323.1015.

Diethyl [1,1'-Biphenyl]-4-ylphosphonate (11)

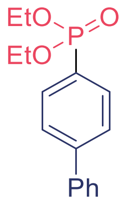

Prepared according to the *General Procedure* from the corresponding thianthrenium salt **1i** (228 mg, 0.50 mmol, 1.0 equiv) and **2a** (430 µL, 415 mg, 2.5 mmol, 5.0 equiv). After purification by flash column chromatography (hexane:EtOAc 2:3), the title compound **11** was obtained as a yellowish oil (119 mg, 0.41 mmol, 82%). $\mathbf{R_f}$ = 0.32 (silica gel, *n*-hexane / EtOAc, 2:3 (v/v)). The spectral data matches those previously reported.[4] **^{1}H NMR** (300 MHz, $CDCl_3$), δ (ppm) = 7.99 – 7.75 (m, 2H), 7.72 – 7.61 (m, 2H), 7.60 – 7.53 (m, 2H), 7.48 – 7.30 (m, 3H), 4.24 – 3.96 (m, 4H), 1.32 (t, *J* = 7.1 Hz, 6H). **^{13}C{^{1}H} NMR** (75 MHz, $CDCl_3$), δ (ppm) = 145.2 (d, *J* = 3.2 Hz), 140.0, 132.3 (d, *J* = 10.2 Hz, 2C), 128.9 (2C), 128.2, 127.3 (2C), 127.2 (d, *J* = 15.4 Hz, 2C), 126.0, 62.1 (d, *J* = 5.6 Hz, 2C), 16.4, 16.3. **^{31}P{^{1}H} NMR** (122 MHz, $CDCl_3$), δ (ppm) = 18.9.

Diethyl (4'-Bromo-[1,1'-biphenyl]-4-yl)phosphonate (12)

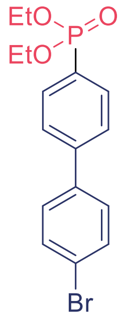

Prepared according to the *General Procedure* from the corresponding thianthrenium salt **1j** (267 mg, 0.50 mmol, 1.0 equiv), **2a** (430 µL, 415 mg, 2.5 mmol, 5.0 equiv) and overnight irradiation. After purification by flash column chromatography (hexane:EtOAc 1:1), the title compound **12** was obtained as a yellowish oil (110 mg, 0.30 mmol, 60%). $\mathbf{R_f}$ = 0.28 (silica gel, *n*-hexane / EtOAc, 1:1 (v/v)). **^{1}H NMR** (300 MHz, $CDCl_3$), δ (ppm) = 7.84 (dd, *J* = 13.0, 8.3 Hz, 2H), 7.65 – 7.58 (m, 2H), 7.54 (d, *J* = 8.5 Hz, 2H), 7.42 (d, *J* = 8.6 Hz, 2H), 4.17 – 4.02 (m, 4H), 1.30 (t, *J* = 7.1 Hz, 6H). **^{13}C{^{1}H} NMR** (75 MHz, $CDCl_3$), δ (ppm) = 143.9 (d, *J* = 3.2 Hz), 138.9, 132.4 (d, *J* = 10.3 Hz, 2C), 132.1 (2C), 128.8 (2C), 127.5 (d, *J* = 189.7 Hz), 126.9 (d, *J* = 15.3 Hz, 2C), 122.6, 62.2 (d, *J* = 5.4 Hz, 2C), 16.4, 16.3. **^{31}P{^{1}H} NMR** (122 MHz, $CDCl_3$), δ (ppm) = 18.6. **FT-IR** (cm^{-1}, neat, ATR), ṽ = 2981, 1603, 1244, 1133, 1050, 1021. **HRMS (ESI)** calcd for $C_{16}H_{19}BrO_3P$ $[M+H]^+$: 369.0250, found 369.0247.

---

[4] N. Shen, R. Li, C. Liu, X. Shen, W. Guan, R. Shang, *ACS Catal.* 2022, **12**, 2788-2795.

Methyl 4-(4'-(Diethoxyphosphoryl)-[1,1'-biphenyl]-4-yl)-4-oxobutanoate (13)

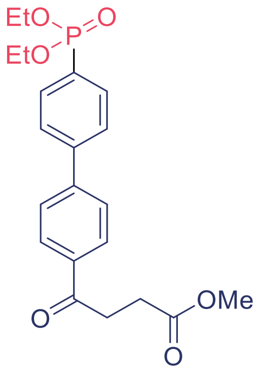

Prepared according to the *General Procedure* from the corresponding thianthrenium salt **1k** (285 mg, 0.50 mmol, 1.0 equiv), **2a** (430 µL, 415 mg, 2.5 mmol, 5.0 equiv) and overnight irradiation. After purification by flash column chromatography (hexane:EtOAc 1:1), the title compound **13** was obtained as a tan oil (149 mg, 0.37 mmol, 74%). $\mathbf{R_f}$ = 0.26 (silica gel, *n*-hexane / EtOAc, 1:1 (v/v)). **^{1}H NMR** (400 MHz, $CDCl_3$), δ (ppm) = 8.10 (d, *J* = 8.1 Hz, 2H), 7.93 (dd, *J* = 13.0, 7.9 Hz, 2H), 7.77 – 7.69 (m, 4H), 4.24 – 4.06 (m, 4H), 3.74 (s, 3H), 3.38 (t, *J* = 6.6 Hz, 2H), 2.82 (t, *J* = 6.5 Hz, 2H), 1.37 (t, *J* = 7.1 Hz, 6H). **^{13}C{^{1}H} NMR** (101 MHz, $CDCl_3$), δ (ppm) = 197.6, 173.3, 144.6, 143.7 (d, *J* = 3.3 Hz), 135.9, 132.5 (d, *J* = 10.2 Hz, 2C), 129.1, 128.8 (2C), 127.5 (2C), 127.3 (d, *J* = 15.3 Hz, 2C), 62.2 (d, *J* = 5.4 Hz, 2C), 51.9, 33.5, 28.0, 16.4, 16.3. **^{31}P{^{1}H} NMR** (162 MHz, $CDCl_3$), δ (ppm) = 18.4. **FT-IR** (cm^{-1}, neat, ATR), ṽ = 2986, 1733, 1680, 1393, 1248, 1136, 1053, 1021. **HRMS (ESI)** calcd for $C_{21}H_{26}O_6P$ $[M+Na]^+$: 405.1462, found 405.1448.

Methyl 2-(4'-(Diethoxyphosphoryl)-2-fluoro-[1,1'-biphenyl]-4-yl)propanoate (14)

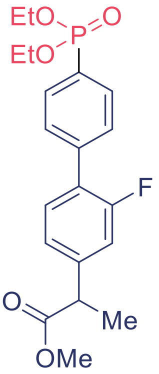

Prepared according to the *General Procedure* from the corresponding thianthrenium salt **1l** (280 mg, 0.50 mmol, 1.0 equiv) and **2a** (430 µL, 415 mg, 2.5 mmol, 5.0 equiv). After purification by flash column chromatography (hexane:EtOAc 1:2), the title compound **14** was obtained as a tan oil (173 mg, 0.44 mmol, 88%). $\mathbf{R_f}$ = 0.55 (silica gel, *n*-hexane / EtOAc, 1:2 (v/v)). **^{1}H NMR** (300 MHz, $CDCl_3$), δ (ppm) = 7.89 (dd, *J* = 13.1, 8.5 Hz, 2H), 7.65 (ddd, *J* = 8.4, 3.8, 1.6 Hz, 2H), 7.42 (t, *J* = 8.1 Hz, 1H), 7.22 – 7.12 (m, 2H), 4.29 –

4.06 (m, 4H), 3.79 (q, J = 7.1 Hz, 1H), 3.72 (s, 3H), 1.56 (d, J = 7.2 Hz, 3H), 1.37 (t, J = 7.0, 6H). **^{13}C{^{1}H} NMR** (75 MHz, $CDCl_3$), δ (ppm) = 174.2, 159.6 (d, J = 249.4 Hz), 142.8 (d, J = 7.7 Hz), 139.5 (dd, J = 3.3, 1.3 Hz), 131.9 (d, J = 10.2 Hz, 2C), 130.7 (d, J = 3.7 Hz), 128.9 (dd, J = 15.2, 3.2 Hz), 127.5 (d, J = 189.2 Hz), 126.6 (d, J = 13.6 Hz), 123.8 (d, J = 3.3 Hz), 115.4 (d, J = 23.5 Hz, 2C), 62.1 (d, J = 5.4 Hz, 2C), 52.2, 44.9 (d, J = 1.6 Hz), 18.4, 16.4, 16.3. **^{19}F{^{1}H} NMR** (282 MHz, $CDCl_3$), δ (ppm) = -117.2. **^{31}P{^{1}H} NMR** (122 MHz, $CDCl_3$), δ (ppm) = 18.6. **FT-IR** (cm^{-1}, neat, ATR), ṽ = 2953, 2922, 1735, 1457, 1247, 1166, 1127, 1021. **HRMS (ESI)** calcd for $C_{20}H_{25}FO_5P$ [M+H]$^+$: 395.1418, found 395.1407.

Diethyl (4-(2,2,2-Trifluoroacetamido)phenyl)phosphonate (15)

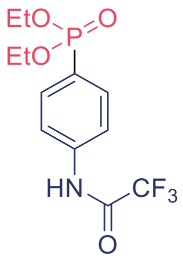

Prepared according to the *General Procedure* from the corresponding thianthrenium salt **1m** (246 mg, 0.50 mmol, 1.0 equiv), **2a** (430 µL, 415 mg, 2.5 mmol, 5.0 equiv) and overnight irradiation. After purification by flash column chromatography (hexane:EtOAc 1:1), the title compound **15** was obtained as a yellowish oil (107 mg, 0.33 mmol, 66%). **R_f** = 0.30 (silica gel, *n*-hexane / EtOAc, 1:1 (v/v)). **^{1}H NMR** (400 MHz, $CDCl_3$), δ (ppm) = 9.73 (s, 1H), 7.92 – 7.78 (m, 4H), 4.20 – 4.05 (m, 4H), 1.34 (t, J = 7.1 Hz, 6H). **^{13}C{^{1}H} NMR** (101 MHz, $CDCl_3$), δ (ppm) = 155.5 (q, J = 37.9 Hz), 139.9 (d, J = 3.7 Hz), 132.9 (d, J = 10.7 Hz, 2C), 125.1 (d, J = 192.7 Hz), 120.4 (d, J = 15.4 Hz, 2C), 115.7 (q, J = 288.7 Hz), 62.4 (d, J = 5.7 Hz, 2C), 16.4, 16.3. **^{19}F{^{1}H} NMR** (376 MHz, $CDCl_3$), δ (ppm) = -75.4. **^{31}P{^{1}H} NMR** (162 MHz, $CDCl_3$), δ (ppm) = 17.4. **FT-IR** (cm^{-1}, neat, ATR), ṽ = 2930, 1621, 1590, 1527, 1480, 1321, 1220, 1019, 958. **HRMS (ESI)** calcd for $C_{12}H_{15}F_3O_4NPNa$ [M+Na]$^+$: 348.0584, found 348.0588.

Diethyl (4-Benzamidophenyl)phosphonate (16)

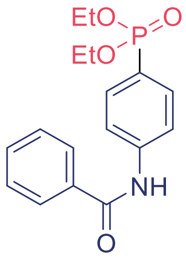

Prepared according to the *General Procedure* from the corresponding thianthrenium salt **1n** (250 mg, 0.50 mmol, 1.0 equiv) and **2a** (430 µL, 415 mg, 2.5 mmol, 5.0 equiv). After purification by flash column

chromatography (hexane:EtOAc 1:4), the title compound **16** was obtained as a white powder (91 mg, 0.28 mmol, 55%). **R$_f$** = 0.25 (silica gel, *n*-hexane / EtOAc, 1:4 (v/v)). **M.P.:** 154-157 ºC. **^{1}H NMR** (400 MHz, $CDCl_3$), δ (ppm) = 9.14 (s, 1H), 8.00 – 7.93 (m, 2H), 7.88 (dd, *J* = 8.6, 3.6 Hz, 2H), 7.73 (dd, *J* = 12.9, 8.6 Hz, 2H), 7.58 – 7.49 (m, 1H), 7.44 (dd, *J* = 8.3, 6.8 Hz, 2H), 4.14 – 3.94 (m, 4H), 1.29 (t, *J* = 7.1 Hz, 6H). **^{13}C{^{1}H} NMR** (101 MHz, $CDCl_3$), δ (ppm) = 166.4, 142.5 (d, *J* = 3.5 Hz), 134.6, 132.8 (d, *J* = 10.9 Hz, 2C), 132.0, 128.6 (2C), 127.5 (2C), 122.8 (d, *J* = 192.4 Hz), 119.9 (d, *J* = 15.2 Hz, 2C), 62.1 (d, *J* = 5.4 Hz, 2C), 16.3, 16.2. **^{31}P{^{1}H} NMR** (162 MHz, $CDCl_3$), δ (ppm) = 18.7. **FT-IR** (cm^{-1}, neat, ATR), ṽ = 2928, 1669, 1591, 1527, 1480, 1321, 1219, 1140, 1019, 962. **Elemental Analysis** calcd for $C_{17}H_{20}NO_4P$: C: 61.26%, H: 6.05%, N: 4.20%; found C: 61.64%, H: 6.24%, N: 3.90%. **HRMS (ESI)** calcd for $C_{17}H_{20}NO_4PNa$ $[M+Na]^+$: 356.1022, found 356.1025.

Diethyl (9-Oxo-9*H*-xanthen-2-yl)phosphonate (17)

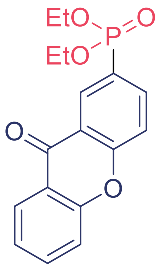

Prepared according to the *General Procedure* from the corresponding thianthrenium salt **1o** (249 mg, 0.50 mmol, 1.0 equiv) and **2a** (430 µL, 415 mg, 2.5 mmol, 5.0 equiv). After purification by flash column chromatography (hexane:EtOAc 1:1), the title compound **17** was obtained as a white solid (108 mg, 0.33 mmol, 65%). **R$_f$** = 0.40 (silica gel, *n*-hexane / EtOAc, 1:1 (v/v)). **M.P.:** 71-73 ºC. **^{1}H NMR** (400 MHz, $CDCl_3$), δ (ppm) = 8.74 (d, *J* = 14.0 Hz, 1H), 8.28 (d, *J* = 7.8 Hz, 1H), 8.17 – 8.06 (m, 1H), 7.71 (t, *J* = 7.7 Hz, 1H), 7.53 (dd, *J* = 8.5, 3.2 Hz, 1H), 7.46 (d, *J* = 8.4 Hz, 1H), 7.36 (t, *J* = 7.5 Hz, 1H), 4.30 – 3.96 (m, 4H), 1.32 (t, *J* = 7.1 Hz, 6H). **^{13}C{^{1}H} NMR** (101 MHz, $CDCl_3$), δ (ppm) = 176.3, 158.3 (d, *J* = 3.4 Hz), 155.9, 137.4 (d, *J* = 11.7 Hz), 135.3, 131.6 (d, *J* = 11.4 Hz), 126.8, 124.6, 124.5 (d, *J* = 193.8 Hz), 121.9, 121.6 (d, *J* = 15.3 Hz), 118.6 (d, *J* = 14.7 Hz), 118.1, 62.4 (d, *J* = 5.6 Hz, 2C), 16.4, 16.3. **^{31}P{^{1}H} NMR** (162 MHz, $CDCl_3$), δ (ppm) = 16.7. **FT-IR** (cm^{-1}, neat, ATR), ṽ = 2921, 1733, 1661, 1612, 1460, 1308, 1232, 1018, 962. **HRMS (ESI)** calcd for $C_{17}H_{18}O_5P$ $[M+H]^+$: 333.0886, found 333.0880. **Elemental analysis**: calcd for $C_{17}H_{17}O_5P$: C: 61.45 %, H: 5.16 %, found: C: 61.43 %, H: 5.17 %.

Diethyl (4-(4-Bromophenoxy)phenyl)phosphonate (18)

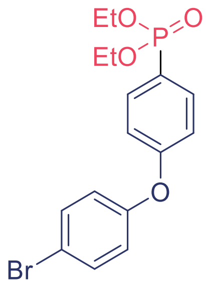

Prepared according to the *General Procedure* from the corresponding thianthrenium salt **1p** (276 mg, 0.50 mmol, 1.0 equiv) and **2a** (430 µL, 415 mg, 2.5 mmol, 5.0 equiv). After purification by flash column chromatography (hexane:EtOAc 1:1), the title compound **18** was obtained as a yellowish oil (165 mg, 0.43 mmol, 86%). $\mathbf{R_f}$ = 0.48 (silica gel, *n*-hexane / EtOAc, 1:1 (v/v)). **^{1}H NMR** (400 MHz, $CDCl_3$), δ (ppm) = 7.75 (dd, *J* = 12.8, 8.7 Hz, 2H), 7.45 (d, *J* = 8.8 Hz, 2H), 6.99 (dd, *J* = 8.7, 3.3 Hz, 2H), 6.91 (d, *J* = 8.9 Hz, 2H), 4.18 – 3.98 (m, 4H), 1.30 (t, *J* = 7.1 Hz, 6H). **^{13}C{^{1}H} NMR** (101 MHz, $CDCl_3$), δ (ppm) = 160.7 (d, *J* = 3.6 Hz), 154.7, 133.9 (d, *J* = 11.2 Hz, 2C), 133.0 (2C), 122.6 (d, *J* = 193.9 Hz), 121.7 (2C), 117.7 (d, *J* = 16.0 Hz, 2C), 117.1, 62.1 (d, *J* = 5.5 Hz, 2C), 16.4, 16.3. **^{31}P{^{1}H} NMR** (162 MHz, $CDCl_3$), δ (ppm) = 18.5. **FT-IR** (cm^{-1}, neat, ATR), ṽ = 2981, 1601, 1578, 1481, 1237, 1130, 1022, 962. **HRMS (ESI)** calcd for $C_{16}H_{18}BrO_4PNa$ $[M+Na]^+$: 407.0018, found 407.0014.

Methyl 5-(4-(Diethoxyphosphoryl)-2,5-dimethylphenoxy)-2,2-dimethylpentanoate (19)

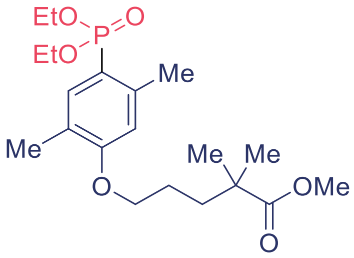

Prepared according to the *General Procedure* from the corresponding thianthrenium salt **1q** (283 mg, 0.50 mmol, 1.0 equiv), **2a** (430 µL, 415 mg, 2.5 mmol, 5.0 equiv) and overnight irradiation. After purification by flash column chromatography (hexane:EtOAc 1:2), the title compound **19** was obtained as a yellowish oil (130 mg, 0.33 mmol, 65%). $\mathbf{R_f}$ = 0.55 (silica gel, *n*-hexane / EtOAc, 1:2 (v/v)). **^{1}H NMR** (300 MHz, $CDCl_3$), δ (ppm) = 7.65 (d, *J* = 13.8 Hz, 1H), 6.62 (d, *J* = 4.8 Hz, 1H), 4.14 – 4.04 (m, 4H), 3.94 (t, *J* = 5.5 Hz, 2H), 3.64 (s, 3H), 2.49 (s, 3H), 2.16 (s, 3H), 1.76 – 1.67 (m, 4H), 1.29 (t, *J* = 7.1 Hz, 6H), 1.20 (s, 6H). **^{13}C{^{1}H} NMR** (75 MHz, $CDCl_3$), δ (ppm) = 178.1, 160.1 (d, *J* = 3.5 Hz), 141.4 (d, *J* = 11.2 Hz), 136.4 (d, *J* = 11.7 Hz), 123.6 (d, *J* = 15.4 Hz), 117.0 (d, *J* = 189.5 Hz), 113.4 (d, *J* = 16.7 Hz), 67.9, 63.6 (d, *J* = 5.9 Hz, 2C), 61.5 (d, *J* = 5.3 Hz), 51.7, 42.0, 36.9, 25.1 (2C), 21.2 (d, *J* = 3.5 Hz), 16.3, 16.2, 15.5. **^{31}P{^{1}H} NMR** (122 MHz, $CDCl_3$),

δ (ppm) = 20.9. **FT-IR** (cm^{-1}, neat, ATR), ṽ = 2986, 1680, 1375, 1248, 1130, 1045, 1019. **HRMS (ESI)** calcd for $C_{20}H_{34}O_6P$ $[M+H]^+$: 401.2088, found 401.2087.

Ethyl 2-(4-Chloro-2-(diethoxyphosphoryl)phenoxy)-2-methylpropanoate (20)

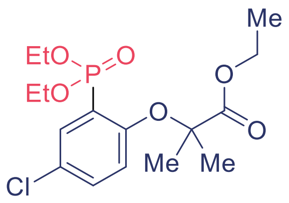

Prepared according to the *General Procedure* from the corresponding thianthrenium salt **1r** (272 mg, 0.50 mmol, 1.0 equiv), **2a** (430 µL, 415 mg, 2.5 mmol, 5.0 equiv) and overnight irradiation. After purification by flash column chromatography (hexane:EtOAc 1:1), the title compound **20** was obtained as a colorless oil (131 mg, 0.38 mmol, 76%). **R$_f$** = 0.45 (silica gel, *n*-hexane / EtOAc, 1:2 (v/v)). **^{1}H NMR** (400 MHz, $CDCl_3$), δ (ppm) = 7.84 (dd, *J* = 15.0, 2.7 Hz, 1H), 7.35 (dd, *J* = 8.8, 2.7 Hz, 1H), 6.69 (dd, *J* = 8.8, 7.1 Hz, 1H), 4.25 (q, *J* = 7.1 Hz, 2H), 4.21 – 4.11 (m, 4H), 1.67 (s, 6H), 1.37 (t, *J* = 7.1 Hz, 6H), 1.26 (t, *J* = 7.1 Hz, 3H). **^{13}C{^{1}H} NMR** (101 MHz, $CDCl_3$), δ (ppm) = 173.9, 156.3 (d, *J* = 1.8 Hz), 135.0 (d, *J* = 8.0 Hz), 133.1 (d, *J* = 2.4 Hz), 126.4 (d, *J* = 19.2 Hz), 121.2 (d, *J* = 185.9 Hz), 117.6 (d, *J* = 10.4 Hz), 80.2, 62.2 (d, *J* = 5.4 Hz, 2C), 61.6, 25.1 (2C), 16.4, 16.3, 14.1. **^{31}P{^{1}H} NMR** (162 MHz, $CDCl_3$), δ (ppm) = 14.5. **FT-IR** (cm^{-1}, neat, ATR), ṽ = 2982, 2934, 1735, 1586, 1467, 1384, 1247, 1137, 1079, 1019. **HRMS (ESI)** calcd for $C_{16}H_{25}ClO_6P$ $[M+H]^+$: 379.1072, found 379.1074.

Isopropyl 2-(4-(4-Chlorobenzoyl)-2-(diethoxyphosphoryl)phenoxy)-2-methylpropanoate (21)

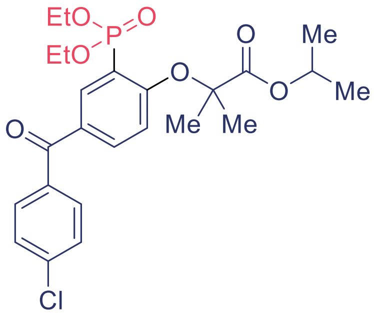

Prepared according to the *General Procedure* from the corresponding thianthrenium salt **1s** (331 mg, 0.50 mmol, 1.0 equiv) and **2a** (430 µL, 415 mg, 2.5 mmol, 5.0 equiv). After purification by flash column chromatography (hexane:EtOAc 1:1), the title compound **21** was obtained as a yellowish oil (87 mg, 0.18 mmol, 35%). **R$_f$** = 0.36 (silica gel, *n*-hexane / EtOAc, 1:1 (v/v)). **^{1}H NMR** (400 MHz, $CDCl_3$), δ (ppm) = 8.31 (dd, *J* = 15.1, 2.3 Hz, 1H), 7.93 (ddd, *J* = 8.7, 2.4, 0.7 Hz, 1H), 7.70 (d, *J* = 8.6 Hz, 2H), 7.47 (d, *J* = 8.6 Hz, 2H), 6.78 (dd, *J* = 8.7, 6.2 Hz, 1H), 5.10 (p, *J* = 6.3 Hz, 1H), 4.22 – 4.00 (m, 4H), 1.73 (s, 6H), 1.35 (t, *J* = 7.1

Hz, 6H), 1.21 (d, J = 6.3 Hz, 6H). **^{13}C{^{1}H} NMR** (101 MHz, $CDCl_3$), δ (ppm) = 193.5, 172.7, 161.3 (d, J = 2.4 Hz), 138.8, 138.2 (d, J = 8.1 Hz), 135.8, 135.5 (d, J = 1.9 Hz), 131.1 (2C), 129.7 (d, J = 13.5 Hz), 128.8 (2C), 118.7 (d, J = 186.9 Hz), 115.1 (d, J = 9.1 Hz), 80.5, 69.5, 62.2 (d, J = 5.5 Hz, 2C), 25.2 (2C), 21.5 (2C), 16.4, 16.3. **^{31}P{^{1}H} NMR** (162 MHz, $CDCl_3$), δ (ppm) = 14.8. **FT-IR** (cm^{-1}, neat, ATR), ṽ = 2981, 2925, 1728, 1590, 1266, 1089, 1022, 968. **HRMS (ESI)** calcd for $C_{24}H_{31}ClO_7P$ [M+H]$^+$: 497.1490, found 497.1498.

Diethyl (4-Fluorophenyl)phosphonate (22)

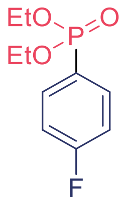

Prepared according to the *General Procedure* from the corresponding thianthrenium salt **1t** (199 mg, 0.50 mmol, 1.0 equiv) and **2a** (430 µL, 415 mg, 2.5 mmol, 5.0 equiv). After purification by flash column chromatography (hexane:EtOAc 1:2), the title compound **22** was obtained as a tan oil (110 mg, 0.48 mmol, 95%). **R_f** = 0.50 (silica gel, *n*-hexane / EtOAc, 1:2 (v/v)). The spectral data matches those previously reported.[5] **^{1}H NMR** (400 MHz, $CDCl_3$), δ (ppm) = 7.79 (ddd, J = 12.7, 8.7, 4.7 Hz, 2H), 7.14 – 7.09 (m, 2H), 4.17 – 3.99 (m, 4H), 1.28 (t, J = 7.0 Hz, 6H). **^{13}C{^{1}H} NMR** (101 MHz, $CDCl_3$), δ (ppm) = 165.3 (dd, J = 253.2, 3.9 Hz), 134.3 (dd, J = 11.2, 8.9 Hz, 2C), 124.5 (dd, J = 193.0, 3.4 Hz), 115.8 (dd, J = 21.4, 16.3 Hz, 2C), 62.2 (d, J = 5.4 Hz, 2C), 16.3, 16.2. **^{19}F{^{1}H} NMR** (376 MHz, $CDCl_3$), δ (ppm) = -106.1. **^{31}P{^{1}H} NMR** (162 MHz, $CDCl_3$), δ (ppm) = 17.7.

Diethyl (4-Chlorophenyl)phosphonate (23)

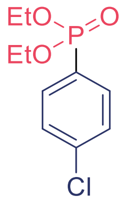

Prepared according to the *General Procedure* from the corresponding thianthrenium salt **1u** (212 mg, 0.50 mmol, 1.0 equiv) and **2a** (430 µL, 415 mg, 2.5 mmol, 5.0 equiv). After purification by flash column chromatography (hexane:EtOAc 1:2), the title compound **23** was obtained as a yellowish oil (112 mg, 0.45 mmol, 90%). **R_f** = 0.45 (silica gel, *n*-hexane / EtOAc, 1:2 (v/v)). The spectral data matches those previously reported.[5] **^{1}H NMR** (400 MHz, $CDCl_3$), δ (ppm) = 7.74 (dd, J = 12.9, 8.4 Hz, 2H), 7.44 (dd, J = 8.4, 3.5 Hz, 2H), 4.19 – 4.02 (m, 4H), 1.31 (t, J = 7.1 Hz, 6H). **^{13}C{^{1}H} NMR** (101 MHz, $CDCl_3$), δ (ppm) = 138.9, 133.2

[5] L. Pan, A. S. Kelley, M. V. Cooke, M. M. Deckert and S. Laulhé, *ACS Sustainable Chem. Eng.* 2022, **10**, 691–695.

(d, J = 10.7 Hz, 2C), 128.9 (d, J = 15.6 Hz, 2C), 127.0 (d, J = 191.0 Hz), 62.3 (d, J = 5.5 Hz, 2C), 16.4, 16.3. **^{31}P{^{1}H} NMR** (162 MHz, $CDCl_3$), δ (ppm) = 17.6.

Diethyl (4-(Trifluoromethyl)phenyl)phosphonate (24)

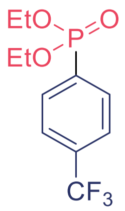

Prepared according to the *General Procedure* from the corresponding thianthrenium salt **1v** (180 mg, 0.40 mmol, 1.0 equiv) and **2a** (430 µL, 415 mg, 2.5 mmol, 5.0 equiv). After purification by flash column chromatography (hexane:EtOAc 1:1), the title compound **24** was obtained as a colorless oil (88 mg, 0.31 mmol, 78%). **R_f** = 0.45 (silica gel, *n*-hexane / EtOAc, 1:1 (v/v)). The spectral data matches those previously reported.[5] **^{1}H NMR** (400 MHz, $CDCl_3$), δ (ppm) = 7.94 (dd, J = 13.0, 7.9 Hz, 2H), 7.71 (dd, J = 8.3, 3.6 Hz, 2H), 4.21 – 4.05 (m, 4H), 1.32 (t, J = 7.1 Hz, 6H). **^{13}C{^{1}H} NMR** (75 MHz, $CDCl_3$), δ (ppm) = 134.4 – 133.6 (m), 132.9 (d, J = 187 Hz), 132.2 (d, J = 10.1 Hz, 2C), 125.3 (dq, J = 15.2, 3.7 Hz, 2C), 62.5 (d, J = 5.6 Hz, 2C), 16.3, 16.2. **^{19}F{^{1}H} NMR** (376 MHz, $CDCl_3$), δ (ppm) = -63.4. **^{31}P{^{1}H} NMR** (162 MHz, $CDCl_3$), δ (ppm) = 16.2.

Diethyl (6-Methoxypyridin-3-yl)phosphonate (25)

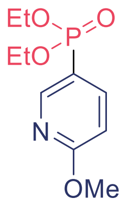

Prepared according to the *General Procedure* from the corresponding thianthrenium salt **1w** (170 mg, 0.40 mmol, 1.0 equiv), **2a** (430 µL, 415 mg, 2.5 mmol, 5.0 equiv) and overnight irradiation. After purification by flash column chromatography (hexane:EtOAc:Et_3N 1:2:0.02), the title compound **25** was obtained as a yellow oil (45 mg, 0.18 mmol, 45%). **R_f** = 0.50 (silica gel, *n*-hexane / EtOAc / Et_3N, 1:2:0.02 (v/v/v)). **^{1}H NMR** (400 MHz, $CDCl_3$), δ (ppm) = 8.57 (ddd, J = 6.9, 2.3, 0.8 Hz, 1H), 7.90 (ddd, J = 11.7, 8.5, 2.3 Hz, 1H), 6.80 (ddd, J = 8.5, 2.7, 0.8 Hz, 1H), 4.19 – 4.06 (m, 4H), 3.98 (s, 3H), 1.32 (t, J = 7.1 Hz, 6H). **^{13}C{^{1}H} NMR** (101 MHz, $CDCl_3$), δ (ppm) = 166.5, 151.5 (d, J = 14.7 Hz), 141.4 (d, J = 10.1 Hz), 116.8 (d, J = 196.5 Hz), 111.1 (d, J = 12.7 Hz), 62.2 (d, J = 5.5 Hz), 53.8, 16.3, 16.2. **^{31}P{^{1}H} NMR** (162 MHz, $CDCl_3$), δ (ppm) = 17.5. **FT-IR** (cm^{-1}, neat, ATR), ṽ = 3479, 2982, 2946, 1592, 1486, 1289, 1239, 1129, 1013. **HRMS (ESI)** calcd for $C_{10}H_{16}NO_4P$ $[M+Na]^+$: 268.0709, found 268.0701.

Methyl 5-(Dimethoxyphosphoryl)-2-methoxybenzoate (26)

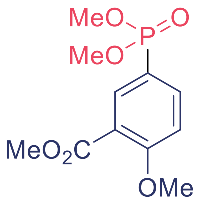

Prepared according to the *General Procedure* from the corresponding thianthrenium salt **1a** (234 mg, 0.50 mmol, 1.0 equiv) and trimethyl phosphite (**2b**) (295 µL, 310 mg, 2.5 mmol, 5.0 equiv). After purification by flash column chromatography (hexane:EtOAc 1:2), the title compound **26** was obtained as a yellow oil (125 mg, 0.46 mmol, 91%). $\mathbf{R_f}$ = 0.28 (silica gel, *n*-hexane / EtOAc, 1:2 (v/v)). ^{1}**H NMR** (400 MHz, $CDCl_3$), δ (ppm) = 8.13 (dd, *J* = 13.3, 2.1 Hz, 1H), 7.84 (ddd, *J* = 12.6, 8.6, 2.1 Hz, 1H), 7.02 (dd, *J* = 8.6, 3.3 Hz, 1H), 3.89 (s, 3H), 3.83 (s, 3H), 3.70 (s, 3H), 3.67 (s, 3H). ^{13}**C{^{1}H} NMR** (101 MHz, $CDCl_3$), δ (ppm) = 165.5 (d, *J* = 1.9 Hz), 162.2 (d, *J* = 3.4 Hz), 137.4 (d, *J* = 11.2 Hz), 135.6 (d, *J* = 12.1 Hz), 120.4 (d, *J* = 15.6 Hz), 118.0 (d, *J* = 197.6 Hz), 112.0 (d, *J* = 15.8 Hz), 56.2, 52.7, 52.6, 52.2. ^{31}**P{^{1}H} NMR** (162 MHz, $CDCl_3$), δ (ppm) = 20.8. **FT-IR** (cm^{-1}, neat, ATR), ṽ = 2953, 2922, 1730, 1600, 1437, 1254, 1083, 1014. **HRMS (ESI)** calcd for $C_{11}H_{16}O_6P$ $[M+H]^+$: 275.0679, found 275.0677.

Methyl 5-(Diisopropoxyphosphoryl)-2-methoxybenzoate (27)

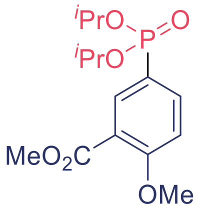

Prepared according to the *General Procedure* from the corresponding thianthrenium salt **1a** (234 mg, 0.50 mmol, 1.0 equiv) and triisopropyl phosphite (**2c**) (620 µL, 520 mg, 2.5 mmol, 5.0 equiv). After purification by flash column chromatography (hexane:EtOAc 1:2), the title compound **27** was obtained as a colorless oil (124 mg, 0.38 mmol, 75%). $\mathbf{R_f}$ = 0.33 (silica gel, *n*-hexane / EtOAc, 1:2 (v/v)). ^{1}**H NMR** (300 MHz, $CDCl_3$), δ (ppm) = 8.20 (dd, *J* = 13.3, 2.0 Hz, 1H), 7.89 (ddd, *J* = 12.5, 8.6, 2.0 Hz, 1H), 7.02 (dd, *J* = 8.6, 3.2 Hz, 1H), 4.64 (dp, *J* = 8.0, 6.2 Hz, 2H), 3.93 (s, 3H), 3.87 (s, 3H), 1.34 (d, *J* = 6.2 Hz, 6H), 1.20 (d, *J* = 6.2 Hz, 6H). ^{13}**C{^{1}H} NMR** (101 MHz, $CDCl_3$), δ (ppm) = 165.7 (d, *J* = 1.6 Hz), 161.8 (d, *J* = 3.3 Hz), 137.1 (d, *J* = 11.0 Hz), 135.5 (d, *J* = 12.2 Hz), 121.27 (d, *J* = 197.0 Hz), 120.1 (d, *J* = 15.5 Hz), 111.8 (d, *J* = 15.7 Hz), 70.8 (d, *J* = 5.4 Hz, 2C), 56.1, 52.1, 24.0 (d, *J* = 4.0 Hz, 2C), 23.8 (d, *J* = 4.9 Hz, 2C). ^{31}**P{^{1}H} NMR** (162 MHz, $CDCl_3$), δ (ppm) = 15.8. **FT-IR** (cm^{-1}, neat, ATR), ṽ = 2921, 2851, 1732, 1600, 1571, 1460, 1375, 1241, 1082, 1003, 973. **HRMS (ESI)** calcd for $C_{15}H_{24}O_6P$ $[M+H]^+$: 331.1305, found 331.1308.

Methyl 5-(Diphenoxyphosphoryl)-2-methoxybenzoate (28)

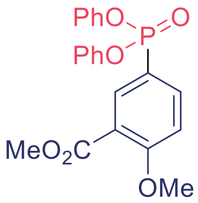

Prepared according to the *General Procedure* from the corresponding thianthrenium salt **1a** (234 mg, 0.50 mmol, 1.0 equiv) and triphenyl phosphite (**2d**) (658 µL, 775 mg, 2.5 mmol, 5.0 equiv). After purification by flash column chromatography (from hexane:EtOAc 7:3 to 1:1), the title compound **28** was obtained as a colorless oil (129 mg, 0.33 mmol, 65%). $\mathbf{R_f}$ = 0.23 (silica gel, *n*-hexane / EtOAc, 7:3 (v/v)). ^{1}**H NMR** (400 MHz, $CDCl_3$), δ (ppm) = 8.39 (dd, *J* = 13.9, 2.1 Hz, 1H), 8.03 (ddd, *J* = 13.1, 8.6, 2.1 Hz, 1H), 7.35 – 7.28 (m, 4H), 7.22 – 7.07 (m, 6H), 7.06 (dd, *J* = 8.7, 3.7 Hz, 1H), 3.94 (s, 3H), 3.89 (s, 3H). ^{13}**C{^{1}H} NMR** (101 MHz, $CDCl_3$), δ (ppm) = 165.4 (d, *J* = 1.8 Hz), 162.6 (d, *J* = 3.4 Hz), 150.3 (d, *J* = 7.4 Hz, 2C), 137.8 (d, *J* = 11.8 Hz), 136.2 (d, *J* = 12.7 Hz), 129.8 (4C), 125.2 (2C), 120.6 (d, *J* = 4.5 Hz, 4C), 120.5, 117.9 (d, *J* = 202.2 Hz), 112.1 (d, *J* = 16.6 Hz), 56.3, 52.3. ^{31}**P{^{1}H} NMR** (162 MHz, $CDCl_3$), δ (ppm) = 11.0. **FT-IR** (cm^{-1}, neat, ATR), ṽ = 2949, 1632, 1596, 1489, 1267, 1186, 1138, 928. **HRMS (ESI)** calcd for $C_{21}H_{20}O_6P$ $[M+H]^+$: 399.0992, found 399.0993.

4. Sunlight-driven synthesis of 4

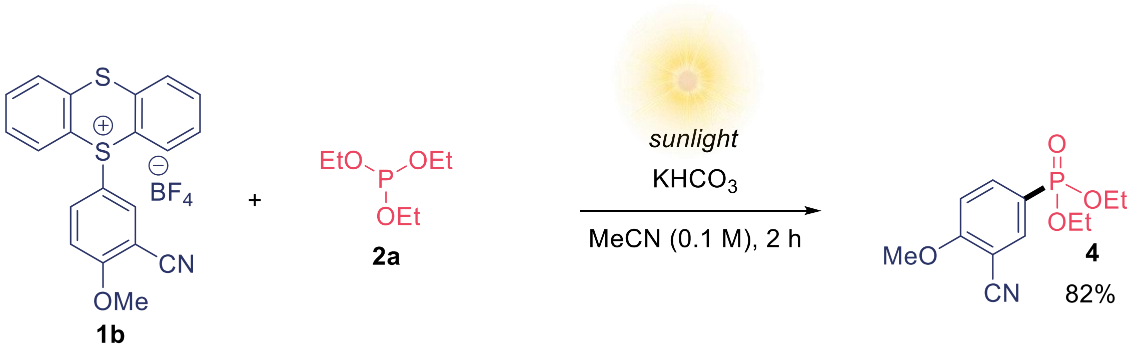

To an 8 mL Chemglass vial (2-dram, 17 x 60 mm, 15-425 Green Open Top Cap, TFE Septa) equipped with a magnetic stir bar was added thianthrenium salt **1b** (218 mg, 0.50 mmol, 1.0 equiv) and $KHCO_3$ (50 mg, 0.50 mmol, 2.0 equiv). The vial was then charged with dry MeCN (5 mL, c = 0.1 M) and closed with a septum. Then, the vial was purged with argon for 30 seconds and **2a** was added in one portion via syringe (429 µL, 415 mg, 2.5 mmol, 5.0 equiv). The cap was covered with *Parafilm* and the reaction mixture was irradiated for 2.5 h with natural sunlight on April 20th 2023 (from 1:40 pm to 3:40 pm in Bellaterra, Barcelona, Spain) as shown in *Figure S2*. The temperature in the room was 23ºC. Upon completion, the solvent was removed under high vacuum and the crude was subjected to purification by flash column chromatography (10 – 60% EtOAc in hexanes). The title compound **4** was obtained as a tan oil (96 mg, 0.41 mmol, 82%).

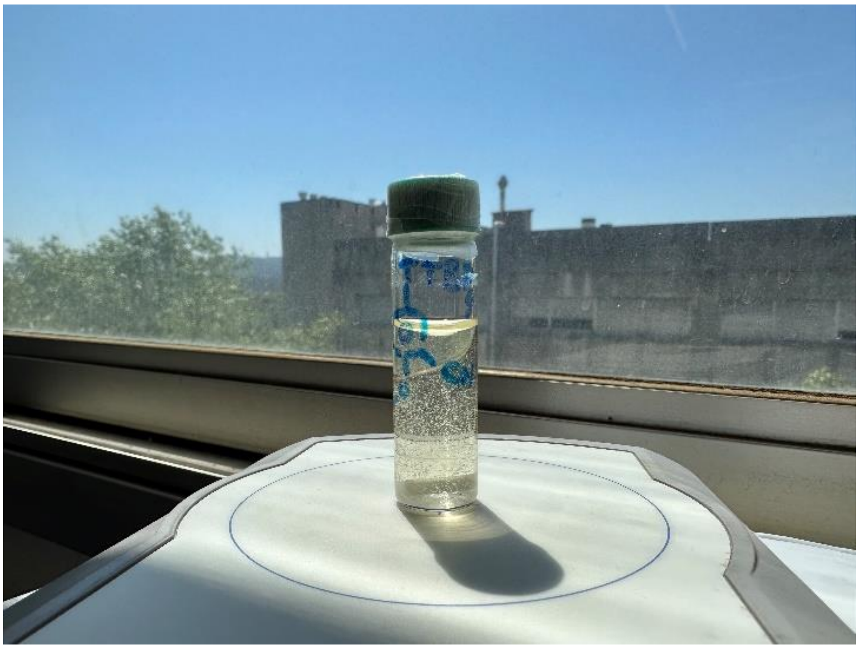

Figure S2. *Setup for the sun-light driven synthesis of* ***4****.*

5. Photochemical Preparation of 22 Using a Solar Simulator

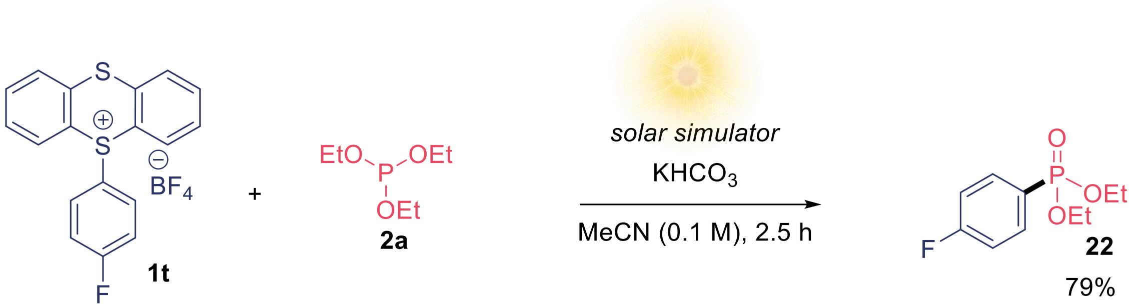

To an 8 mL Chemglass vial (2-dram, 17 x 60 mm, 15-425 Green Open Top Cap, TFE Septa) equipped with a magnetic stir bar was added thianthrenium salt **1t** (199 mg, 0.50 mmol, 1.0 equiv) and $KHCO_3$ (50 mg, 0.5 mmol, 2.0 equiv). The vial was then charged with dry MeCN (5 mL, c = 0.1 M) and closed with a septum. Then, the vial was purged with argon for 30 seconds and **2a** was added in one portion via syringe (429 µL, 415 mg, 2.5 mmol, 5.0 equiv). The cap was covered with *Parafilm* and the reaction mixture was irradiated for 2.5 h with Abel LS-50 Light source as shown in *Figure S3*. The reaction vial was placed at 1 sun distance (intensity of simulator 100 mW/cm^2). The temperature in the room was 21ºC. Upon completion, the solvent was removed under high vacuum and the crude was subjected to purification by flash column chromatography (10 – 60% EtOAc in hexanes). The title compound **22** was obtained as a tan oil (92 mg, 0.40 mmol, 79%).

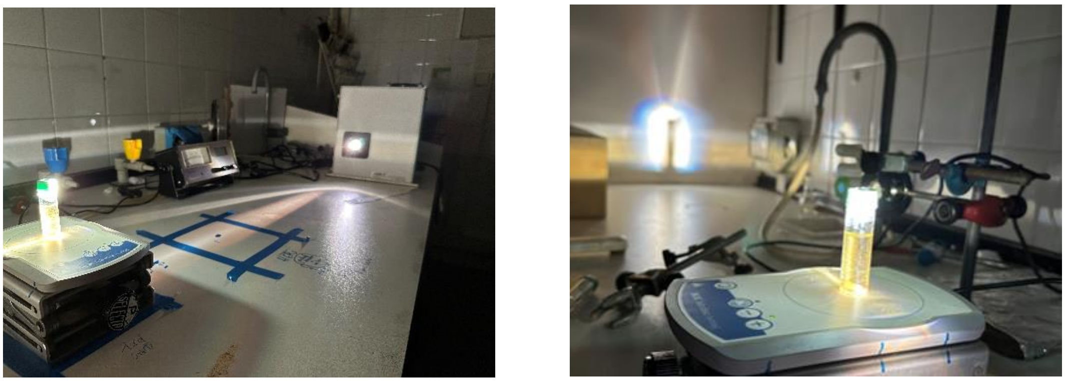

Figure S3. *Setup for the synthesis of* ***22*** *using a solar simulator.*

6. Large scale and Telescoped Synthesis of 18

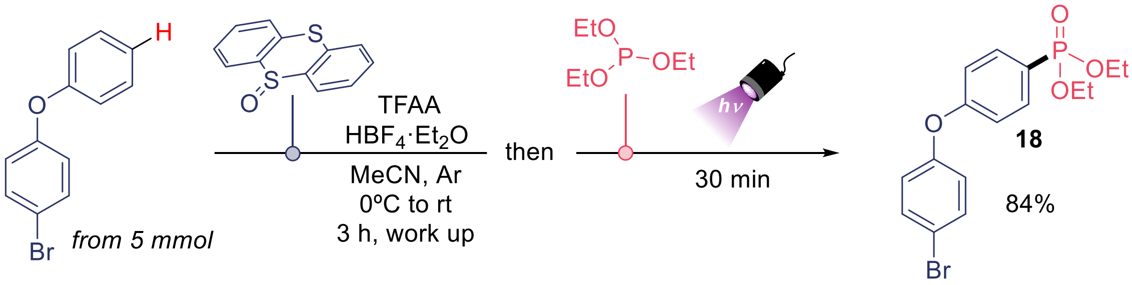

4-Bromophenoxybenzene (1.25 g, 5.0 mmol, 1 equiv) was placed into a 50 mL round-bottomed flask equipped with a magnetic stir bar and dissolved with 8 mL of dry MeCN at 0ºC. Then, while stirring, thianthrene *S*-oxide (1.16 g, 5.0 mmol, 1.0 equiv) was added, followed by $HBF_4·Et_2O$ (0.75 mL, 0.9 g, 5.5 mmol, 1.1 equiv)

and trifluoroacetic anhydride (TFAA) (1.1 mL, 1.6 g, 7.5 mmol, 1.5 equiv). The ice bath was removed, and the reaction mixture was stirred for 3h. Then, the solution was diluted with 20 mL of CH_2Cl_2 and poured into a separatory funnel. The organic phase was washed with a satured solution of $NaHCO_3$ (25x3 mL), and 10% $NaBF_4$ aqueous solution (2 × 25 ml). The organic layer was dried over Na_2SO_4, filtered, and the solvent was removed under reduced pressure. The obtained crude was dissolved in 50 mL of dry MeCN and transferred into a 250 mL regular Schlenk flask. Then, $KHCO_3$ (500 mg, 5.0 mmol, 1.0 equiv) was added, and the solvent purged with argon for 2 minutes. Afterwards, **2a** was added in one portion via syringe (429 µL, 415 mg, 2.5 mmol, 5.0 equiv). The cap was covered with *Parafilm* and the reaction mixture was irradiated for 30 minutes with two Kessil PR160-blue LED lamps (30 W High Luminous DEX 2100 LED, λ_{max} = 390 nm) as shown in *Figure S4*. The temperature of the reaction was maintained at approximately 25 °C via a fan. Upon completion, the solvent was removed under high vacuum and the crude was subjected to purification by flash column chromatography (10 – 60% EtOAc in hexanes). The title compound **18** was obtained as a tan oil (1.61 g, 4.2 mmol, 84%).

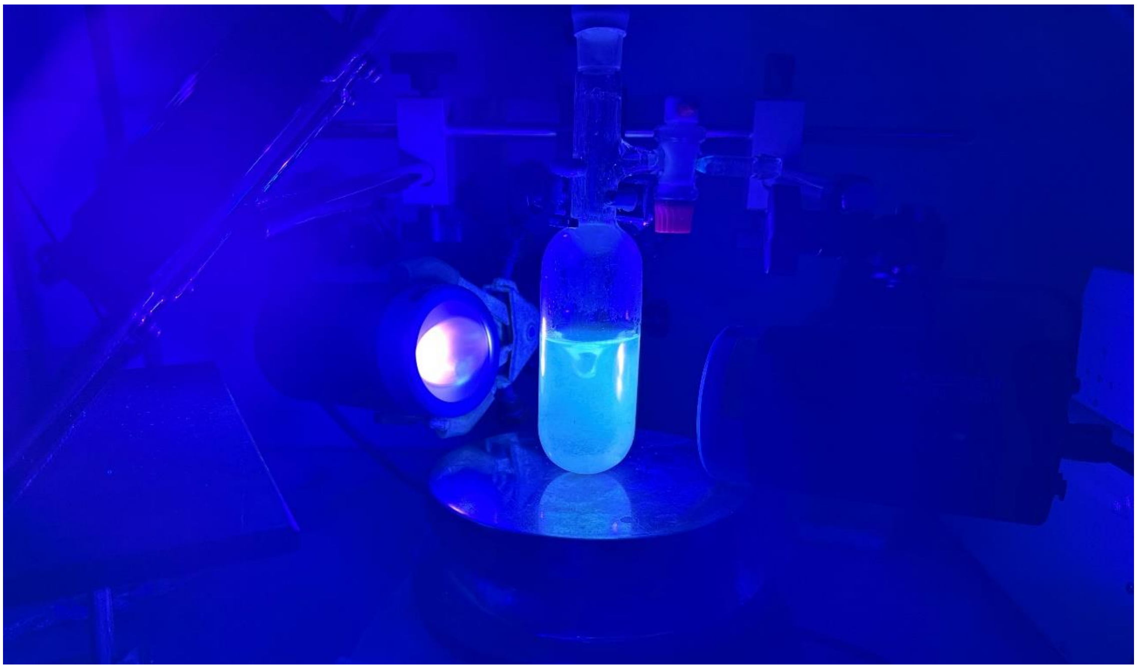

Figure S4. *Setup for the Large Scale and Telescoped Synthesis of* ***18****.*

7. Mechanistic Investigation

7.1. UV/Vis Studies

UV/vis absorption spectra were measured in a 1 cm quartz cuvette using a Genesys 150 UV/vis spectrophotometer from Thermo Scientific. Absorption spectra of individual reaction components and mixtures thereof were recorded. A bathochromic shift was observed for a mixture of thianthrenium tetrafluoroborate salt **1a**, and phosphite **2a**, in MeCN (0.1 M), which was a visibly slight pale yellow in color. This indicates the formation of an electron donor-acceptor (EDA) complex (*Figure S5*, green band).

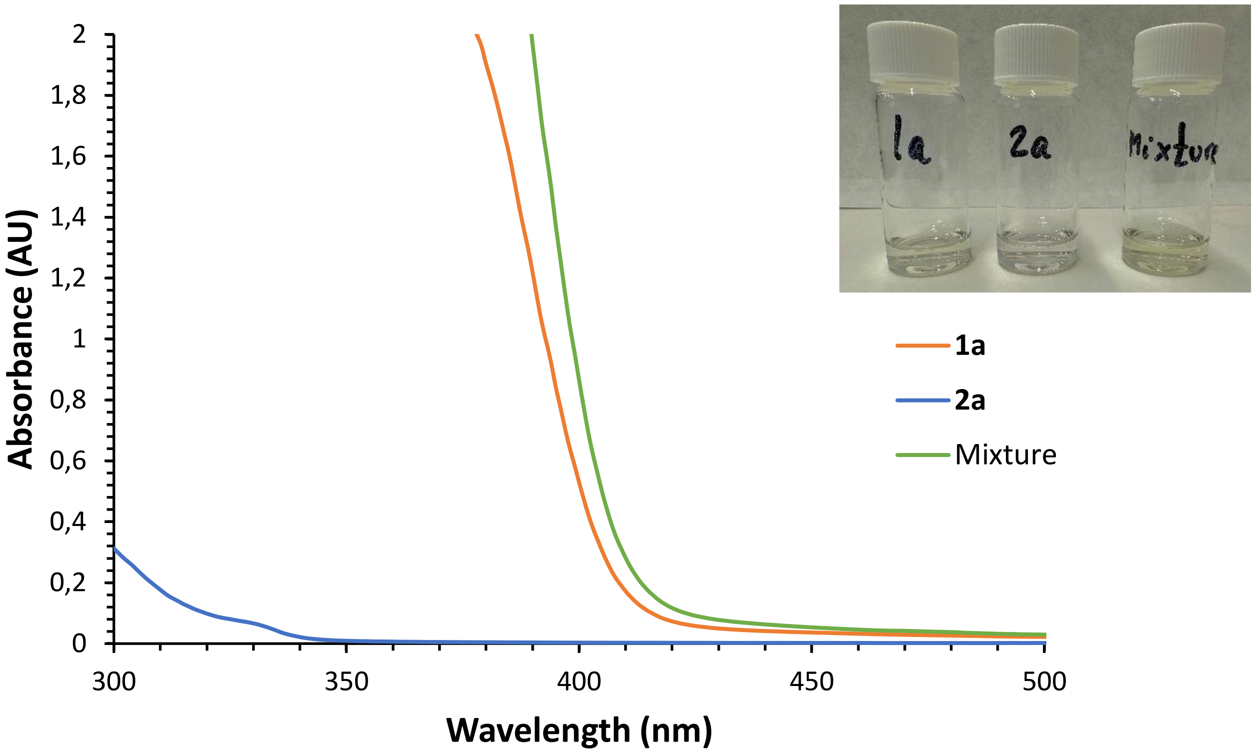

Figure S5. *UV/vis absorption spectra of individual reaction components and a combination thereof. All spectra were measured in MeCN and with a concentration of 0.1 M TT salt* ***1a****, 0.5 M of* ***2a****. The stoichiometry and concentration of samples reflects the used reaction conditions. Visual appearance of reaction components and mixtures thereof.*

7.2. TEMPO Radical Trapping Experiment

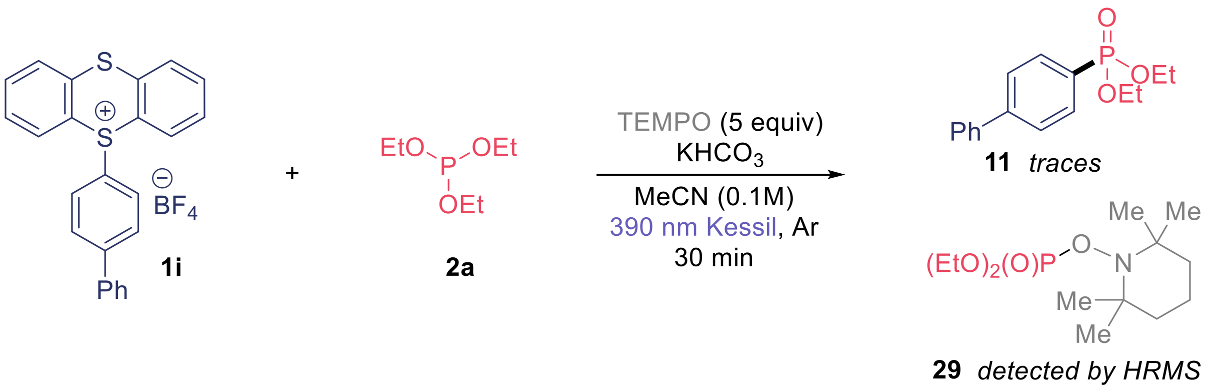

To a flame-dried 4 mL vial equipped with a magnetic stir bar, TT salt **1i** (45 mg, 0.1 mmol, 1.0 equiv), TEMPO (80 mg, 0.5 mmol, 5.0 equiv) and $KHCO_3$ (10 mg, 0.1 mmol, 1.0 equiv) were added, and the vial was subjected to 3 cycles of vacuum/argon degassing. Subsequently, 1 mL of dry MeCN were added under inert atmosphere. Subsequently, phosphite **2a** was added via syringe (88 μL, 2.5 mmol, 5 equiv), and the solution was degassed with Argon for 30 seconds. The reaction mixture was irradiated for 30 minutes with a 390 nm Kessil PR160-purple LED as described in the "*Workflow*" section. The temperature of the reaction was maintained at approximately 25 °C via a fan. After the reaction time, the solvent was removed under reduced pressure. The crude mixture was analyzed by HRMS, and we detected the formation of **29** and unreacted. **HRMS (ESI)** calcd for $C_{13}H_{29}NO_4P$ $[M+H]^+$: 294.1829, found 294.1828.

7.3. Photochemical Quantum Yield (ϕ)

The quantum yield of for our model photochemical reaction was determined using previously reported methods using equation 1:[6]

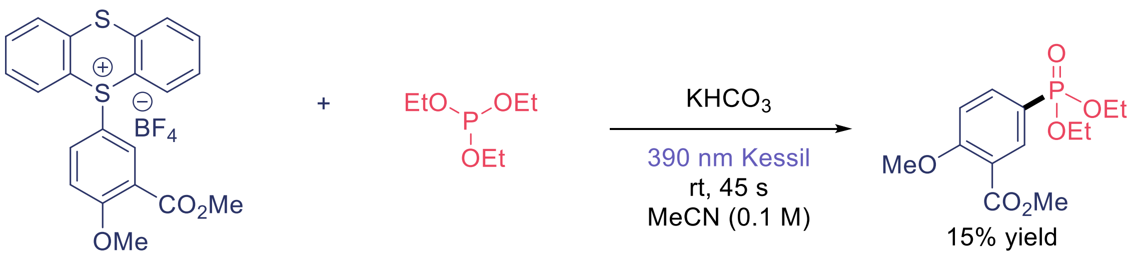

$$\Phi(\text{reaction at 390 nm}) = \frac{\text{mol of formed product}}{\text{mol of photon flux} \cdot \text{t} \cdot \text{f}} \qquad (1)$$

[6] a) M. El Khatib, R. A. M. Serafim, G. A. Molander, *Angew. Chem. Int. Ed.*, 2016, **55**, 254.
b) M. Cismesia; T. P. Yoon, *Chem. Sci.*, 2015, **6**, 5426.

where Φ is the quantum yield of the reaction, t is the time of the reaction (s), f is the incident light absorbed by the EDA Complex at 390 nm and the photon flux is calculated by standard ferrioxalate actinometry.

The fraction of light, f, absorbed was determined according to equation 2:

$$f = 1 - 10^{-A} \qquad (2)$$

where A is the absorbance of the EDA Complex in MeCN at 390 nm. The wavelength of 390 nm was chosen based on the known absolute $\Phi(Fe^{+2})$ value[7] and is the wavelength we are using in our reaction. The absorbance of the EDA Complex was measured (0.1 M) in MeCN (2 mL). The absorbance (A) at 390 nm was determined to be >2, thus indicating the fraction of light absorbed is 0.99 according to equation 2.

Photon flux sample calculation

Standard ferrioxalate actinometry was used to determine the photon flux of the spectrophotometer using equations 3 and 4. For the ferrioxalate actinometer the production of iron(II) ions proceeds by the following reactions:

$$[Fe(C_2O_4)n]^{+(3-2n)} \xrightarrow{\text{light}} Fe^{+2} + (n-1)(C_2O_4)^{-2} + C_2O_4^{-}$$

$$[Fe(C_2O_4)n]^{+(3-2n)} + C_2O_4^{-} \longrightarrow Fe^{+2} + n(C_2O_4)^{-2} + 2\,CO_2$$

The moles of Fe^{+2} formed are determined spectrophotometrically by development with 1,10-phenanthroline (phen) to form the red $[Fe(phen)_3]^{+2}$ moiety (λ = 510 nm).[8] The photon flux is defined as shown in equation 3:

$$\text{Photon Flux} = \frac{\text{mol Fe}^{+2}}{\Phi(\text{Fe}^{+2}) \cdot t \cdot f} \qquad (3)$$

Where Φ is the quantum yield for the ferrioxalate actinometer (1.01 at λ = 438 nm), t is the time (s), and f ~1, and the mol of Fe^{+2} are calculated according to equation 4.

$$\text{mol (Fe}^{+2}) = \frac{V \cdot \Delta A}{l \cdot \epsilon} \qquad (4)$$

Where V is the total volume of the solution, ΔA is the difference in absorbance between irradiated and nonirradiated solutions, l is the path length (1.0 cm), ε is the molar absorptivity at 510 nm (11110 L $mol^{-1}cm^{-1}$).

[7] G.-C. He, S.-Y. Guo, H. Zheng, C.-H. Liu, Y. Li, X.-T. Min, D.-W. Ji, Q.-A. Chen, *Cell Rep. Phys. Sci.* 2022, **3**, 100768.
[8] J. N. Demas, W. D. Bowman, E. F. Zalewski, R. Velapoidl, *J. Phys. Chem.* 1981, **85**, 2766.

The following solutions were prepared in the dark (flasks were wrapped in aluminum foil) and stored in the dark at room temperature:

– Ferrioxalate solution (0.15 M): Potassium ferrioxalate hydrate (0.656 g) was added to a flask wrapped in aluminum foil containing H_2SO_4 (10 mL, 0.05 M). The flask was stirred for complete dissolution of the green solid in complete darkness. It is noteworthy that the solution should not be exposed to any incident light.

– Developer solution: 1,10-Phenanthroline (50 mg) and sodium acetate (11.26 g) were added to a flask containing H_2SO_4 (50 mL, 0.5 M) and sonicated until completely dissolved.

The absorbance of the non-irradiated sample. The buffered solution of phen (350 µL) was added to a ferrioxalate solution (2.0 mL) in a vial that had been covered with aluminum foil and with the lights of the laboratory switched off. The vial was capped and allowed to rest for 1 h and then transferred to a cuvette. The absorbance of the non-irradiated sample was measured at 510 nm to be 1.45 (average of two determinations, light 1 and light 2, see *Figure S6*).

The absorbance of the irradiated sample. In a cuvette equipped with a stir bar was added the ferrioxalate solution (2.0 mL), and the stirred solution was irradiated for 30 s at λ = 390 nm with an excitation slit width = 10.0 nm. After irradiation, the buffered phen solution (350 µL) was added to the cuvette and allowed to rest for 1 h in the dark to allow the ferrous ions to coordinate completely to phen. The absorbance of the sample was measured at 510 nm to be 0.01.

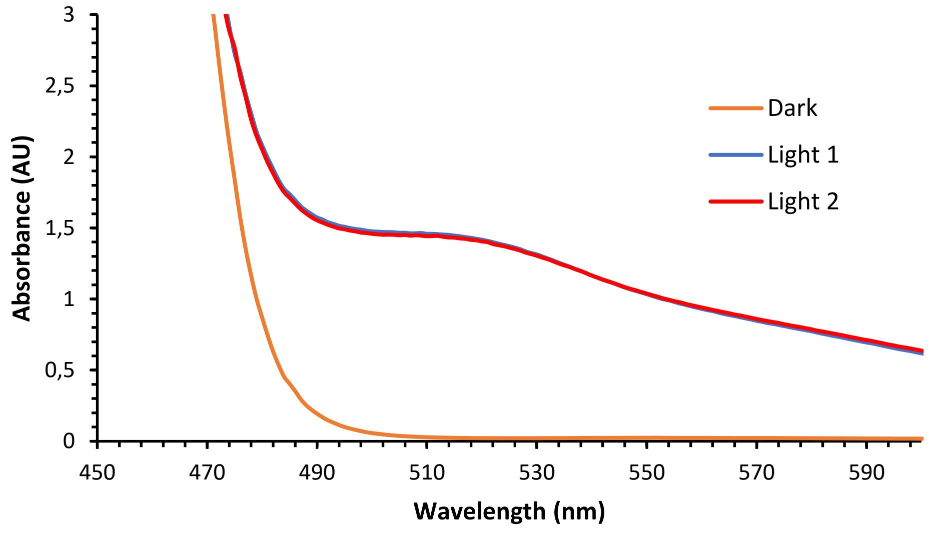

Figure S6. *Absorption spectra for irradiated and non-irradiated samples of red [Fe(phen)$_3$]$^{+2}$*

$$\text{mol}\ (\text{Fe}^{+2}) = \frac{\text{V} \cdot \Delta\text{A}}{\text{l} \cdot \epsilon} \qquad (4)$$

$$\text{mol (Fe}^{+2}) = \frac{0.00235\ \text{L} \cdot 1.44}{1.0\ \text{cm} \cdot 11100\ \text{L} \cdot \text{mol}^{-1}\text{cm}^{-1}} = 3.05\text{x}10^{-7}\text{mol}$$

$$\text{Photon Flux} = \frac{\text{mol Fe}^{+2}}{\Phi(\text{Fe}^{+2}) \cdot \text{t} \cdot \text{f}} \qquad (3)$$

$$\text{Photon Flux} = \frac{3.05\text{x}10^{-7}\text{mol}}{1.19 \cdot 90\ \text{s} \cdot 1} = 2.85x10^{-9}\ einstein\ s^{-1}$$

Quantum yield determination

Therefore, the quantum yield of the reaction is determined to be:

$$\Phi(\text{reaction at 390 nm}) = \frac{\text{mol of formed product}}{\text{mol of photon flux} \cdot \text{t} \cdot \text{f}} \qquad (1)$$

$$\boldsymbol{\Phi}(\textbf{reaction at 390 nm}) = \frac{1.5\text{x}10^{-5}\ \text{mol}}{2.85\text{x}10^{-9}\ \text{einstein s}^{-1} \cdot 45\ \text{s} \cdot 0.99} = \mathbf{118}$$

7.4. Cyclic Voltammetry of TT salt **1a**

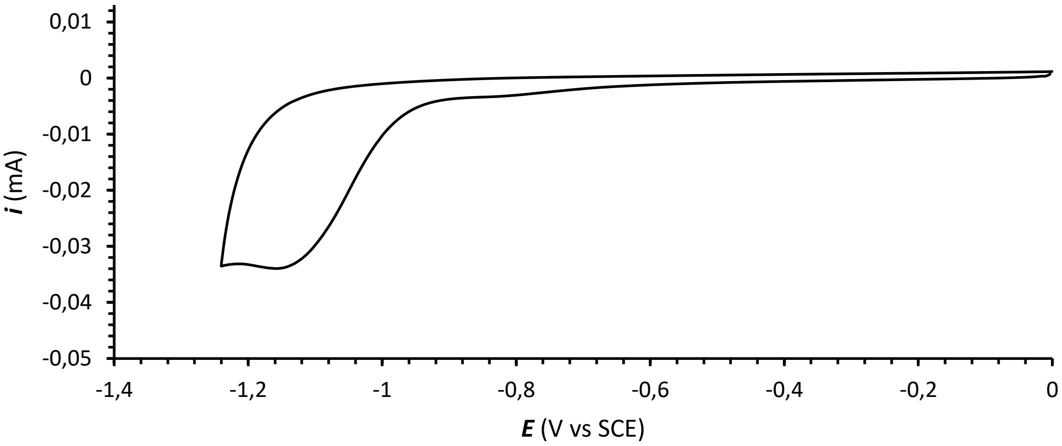

Figure S7. *Cyclic voltammetry experiments of TT salt **1b**. Conditions: 2.5 mM of **1b** in MeCN at scan rate of 100 mV/s, TBAPF$_6$ 0.1 M at r.t. Scan direction: From 0.0 V to negative values. IUPAC plotting.*

7.5. $^{1}H/^{31}P$ HMBC NMR Characterization of Intermediate ***E***

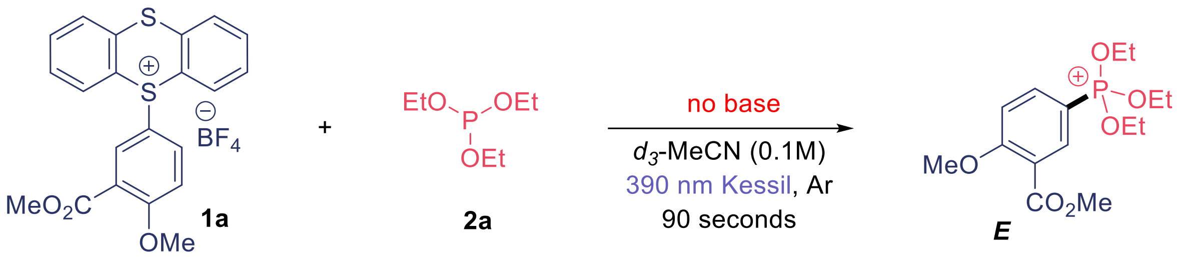

To a flame-dried 4 mL vial equipped with a magnetic stir bar, TT salt **1a** (47 mg, 0.1 mmol, 1.0 equiv) was added, and the vial was subjected to 3 cycles of vacuum/argon degassing. Subsequently, 1 mL of dry deuterated MeCN was added under inert atmosphere. Subsequently, **2a** was added via syringe (88 µL, 2.5 mmol, 5 equiv), and the solution was degassed with Argon for 30 seconds. The reaction mixture was irradiated for 90 seconds with a 390 nm Kessil PR160-purple LED as described in the "*Workflow*" section. The temperature of the reaction was maintained at approximately 25 °C via a fan. Then, a sample of 0.5 mL of the irradiated solution was added in an NMR tube and analyzed in a NEO 300 MHz spectrometer.

We intentionally did not include the base in this experiment to detect the phosphonium intermediate **E** and thianthrene, as well as starting **1a**, **2a**, and maybe product. The ^{1}H NMR spectra confirmed our hypothesis and showed the formation of a new species which bears three OEt groups and thianthrene as main products (*Figure S7*). Additionally, a signal in ^{31}P NMR spectra appeared at a chemical shift of 31 ppm, suggesting a cationic phosphonium intermediate (*Figure S8*).[9]In addition, both experiments showed that there was still unreacted **1a** and traces of desired product. The formation of product was kind of expected because the reaction also works without the addition of any external base (see Table 1, entry 1 in the manuscript). Then, motivated by these observations a bidimensional $^{1}H/^{31}P$ HMBC experiment was done (*Figure S9*). We confirmed that the new species containing three OEt groups attached to an aromatic ring belong to the phosphonium species **E**.

[9] a) D. G. Gilheany, J. S. Kudavalli, A. D. Molloy, K. Nikitin, K. V. Rajendran, WO2012113889A1, Processes for the stereoselective preparation of p-chiral four -coordinated phosphorus borane compounds and p-chiral three-coordinated phosphorus compounds, **2012**.
b) C. Li, L.-B. Han, *Organometallics* 2020, **39**, 3613.

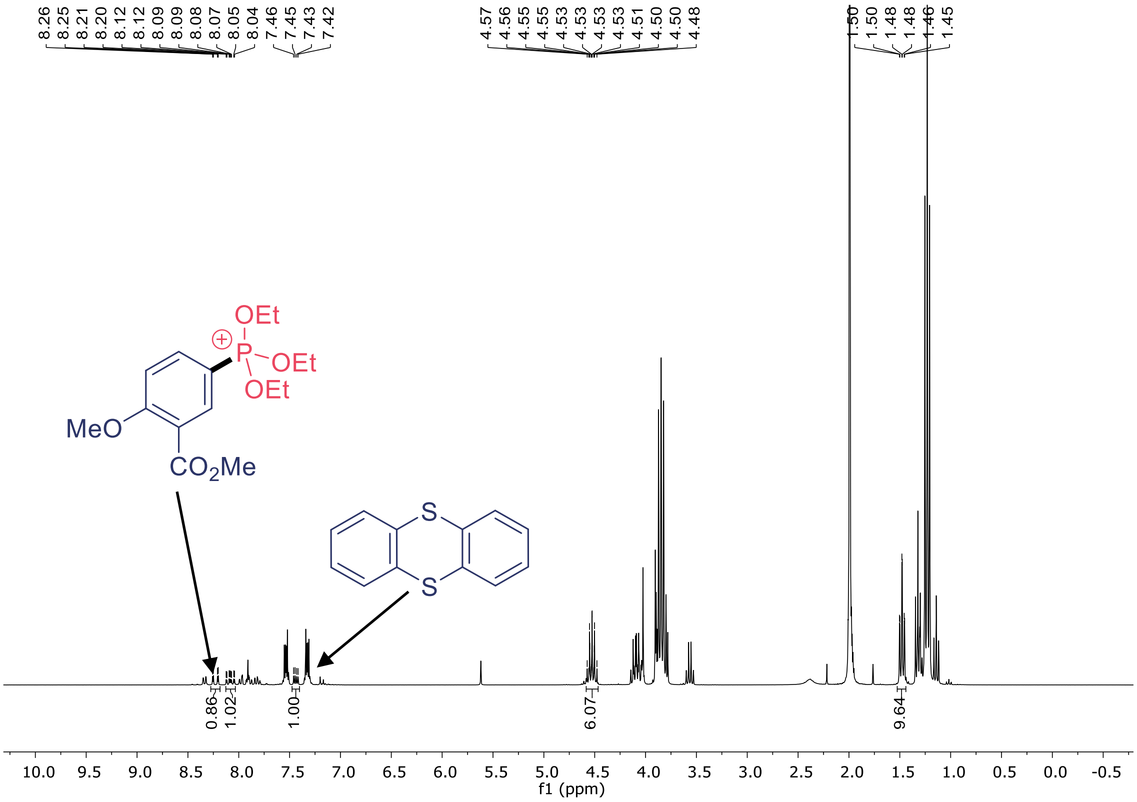

Figure S8. ^{1}H NMR (400 MHz, CD_3CN) of the reaction mixture.

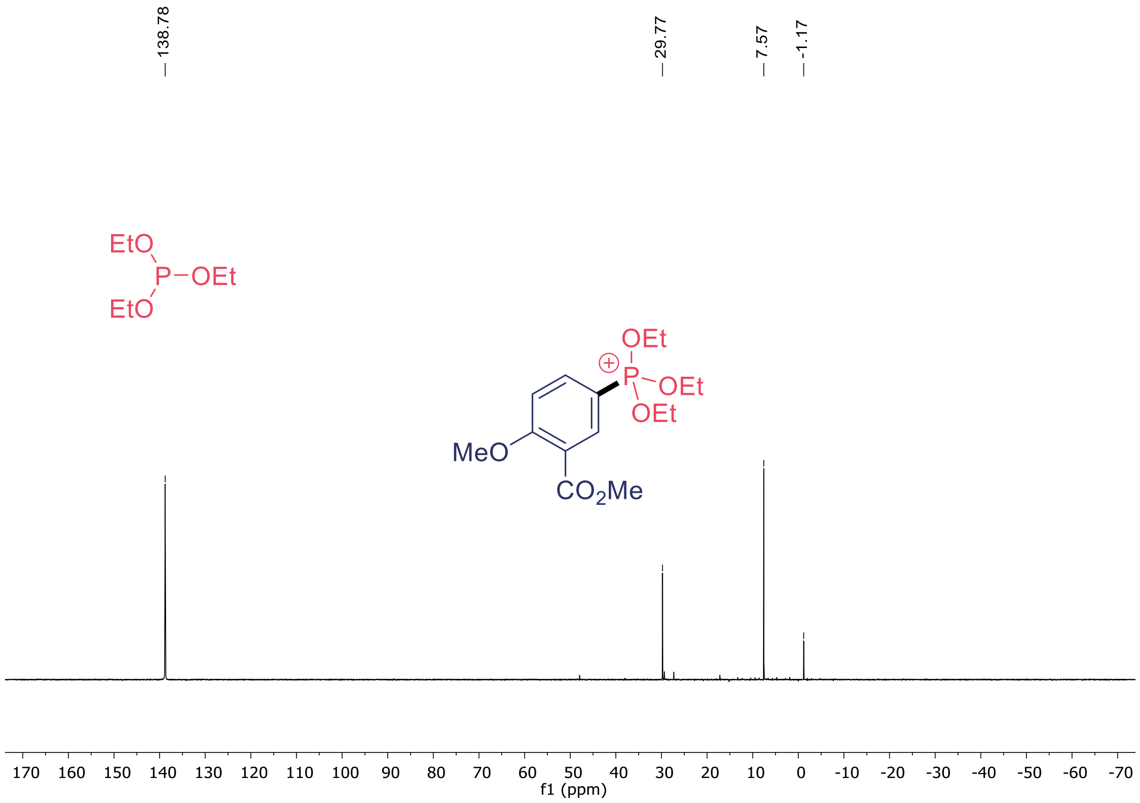

Figure S9. ^{31}P NMR (162 MHz, CD_3CN) of the reaction mixture.

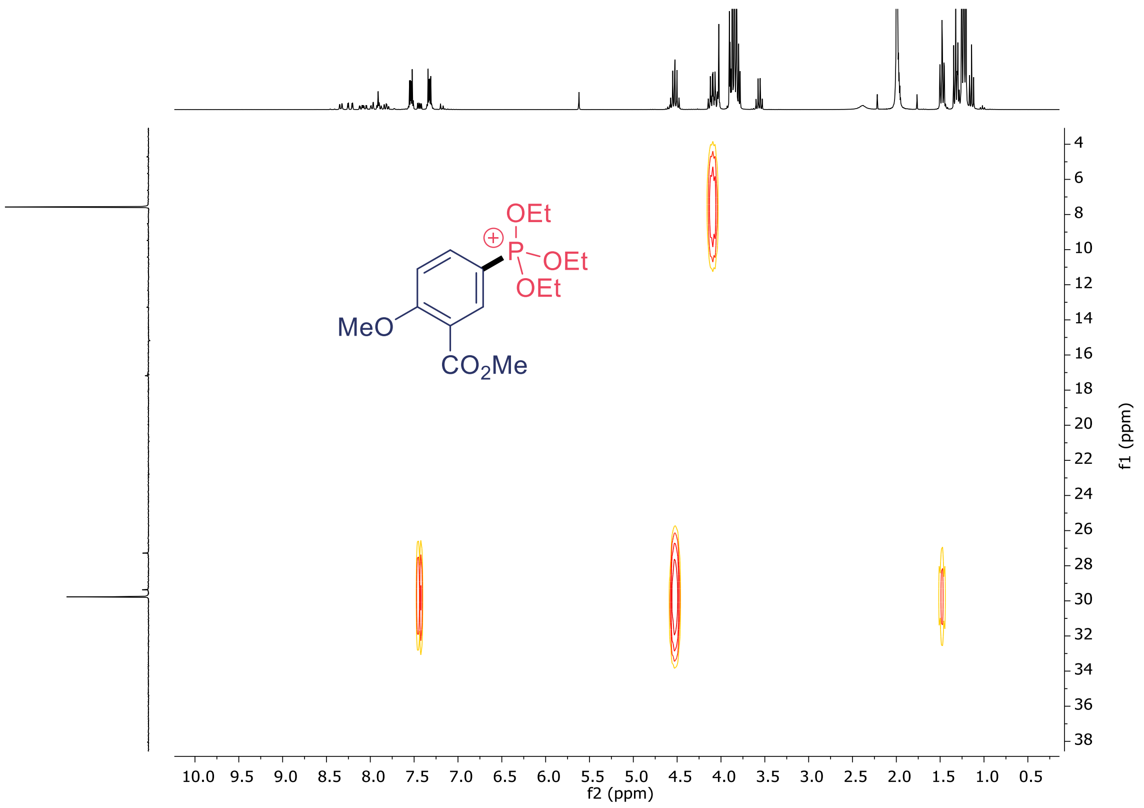

Figure S10. $^{1}H/^{31}P$ HMBC (400 MHz, CD_3CN) of the reaction mixture.

The reported HMBC experiment was done using the following pulse program:

Avance-version (12/01/11); HMBC; 2D H-1/X correlation via heteronuclear zero and double quantum; coherence; optimized on long range couplings; no decoupling range acquisition; using gradient pulses for selection.

128 scans and TD = 1.

7.6. ESI-HRMS Characterization of Intermediate ***E***

To confirm the intermediate ***E*** further, we repeated the previous experiment in *Section 7.5* but using regular MeCN as solvent. The reaction mixture was analyzed by HRMS in *Servei d'Anàlisi Química*. The chromatogram revealed the formation of the following chemical species.

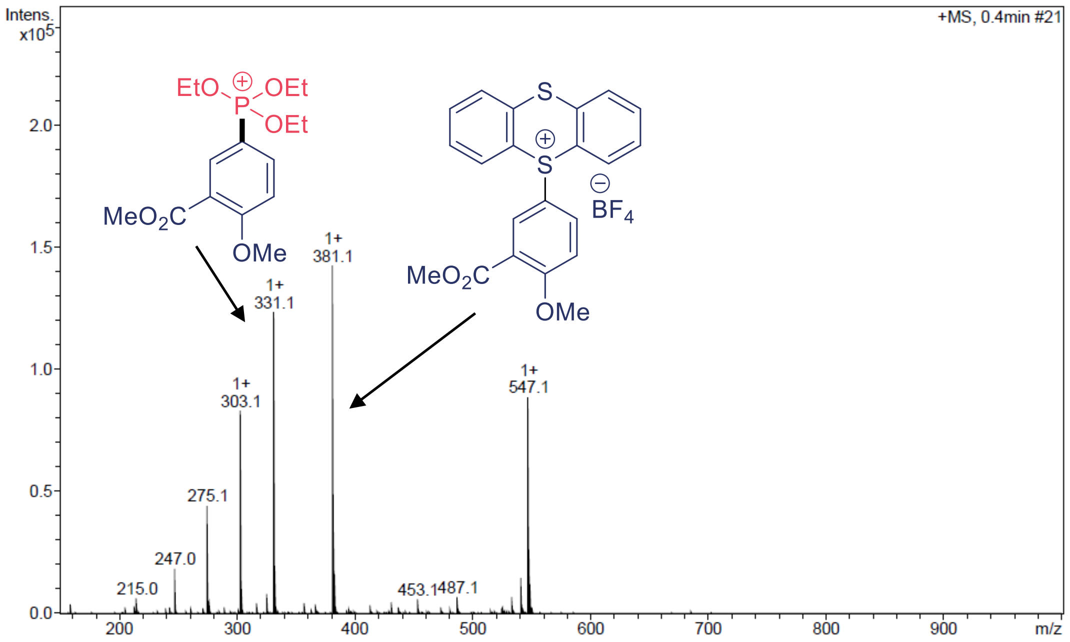

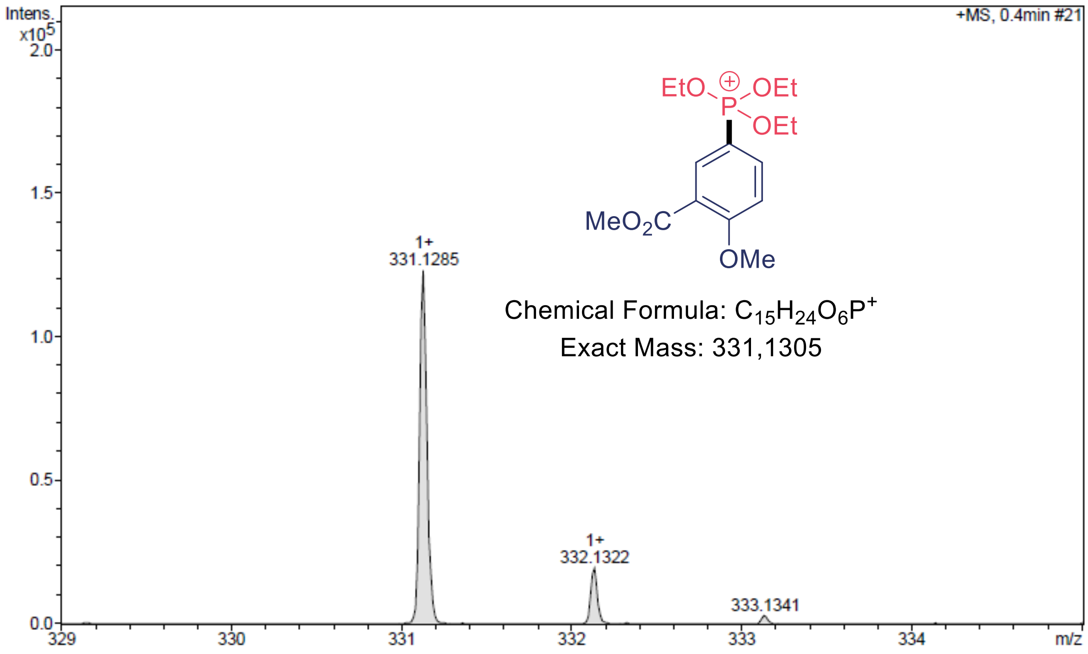

#	m/z	I	I %
1	331.1285	123104	100.0
2	332.1322	19633	15.9
3	333.1341	2891	2.3

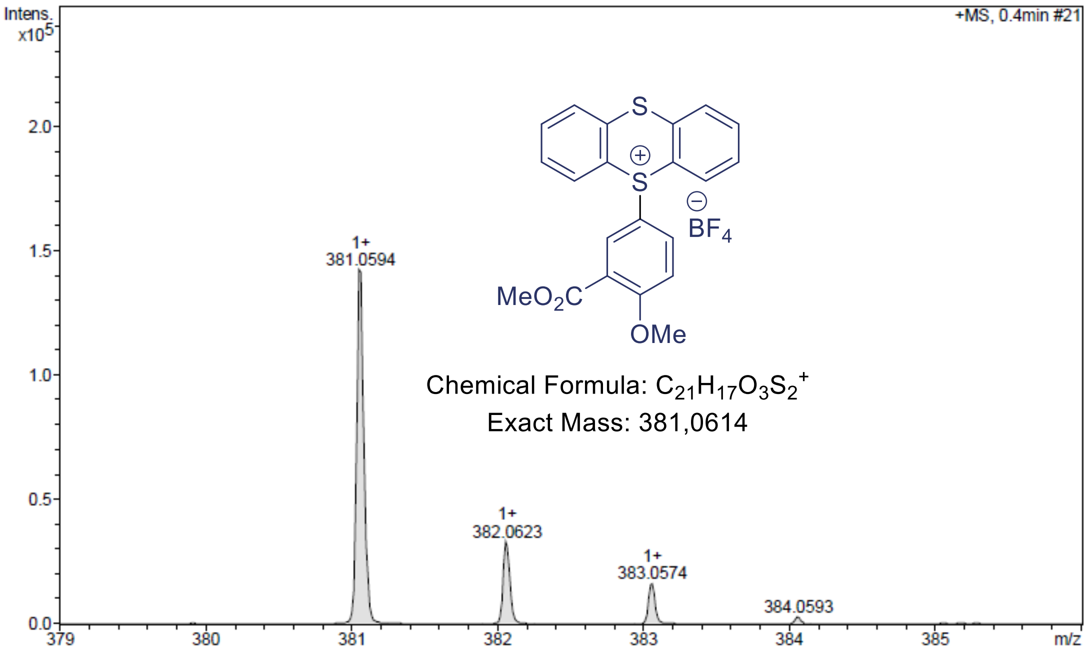

#	m/z	I	I %
1	381.0594	142257	100.0
2	382.0623	33246	23.4
3	383.0574	16383	11.5
4	384.0593	2977	2.1

8. Unsuccessful Substrates

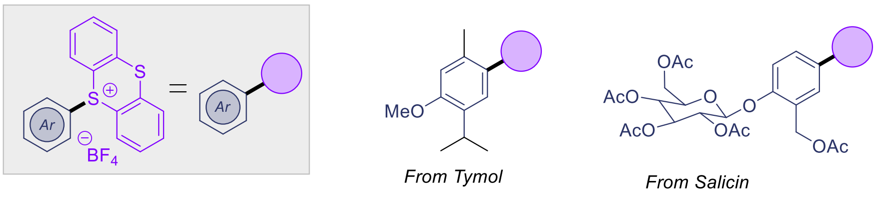

9. NMR Spectra

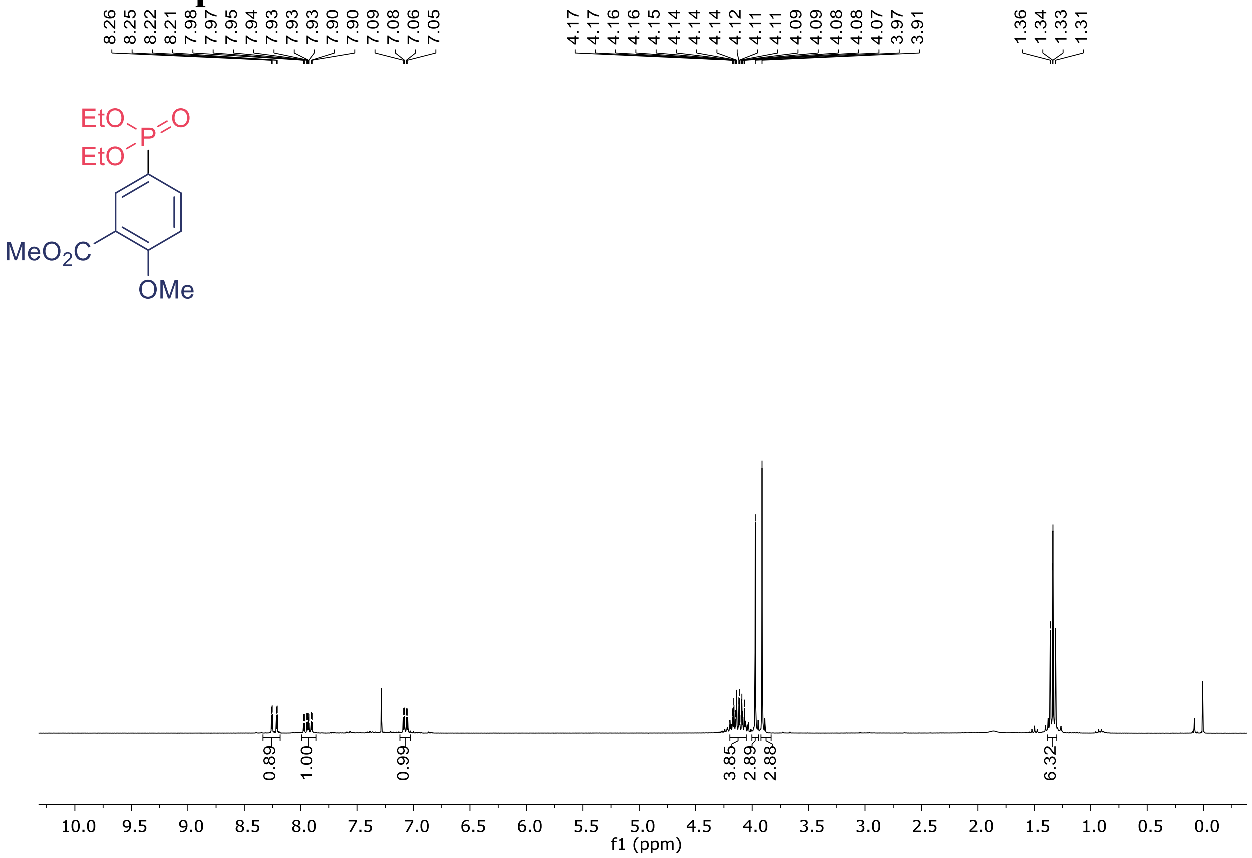

^{1}H NMR (400 MHz, $CDCl_3$) of compound **3**.

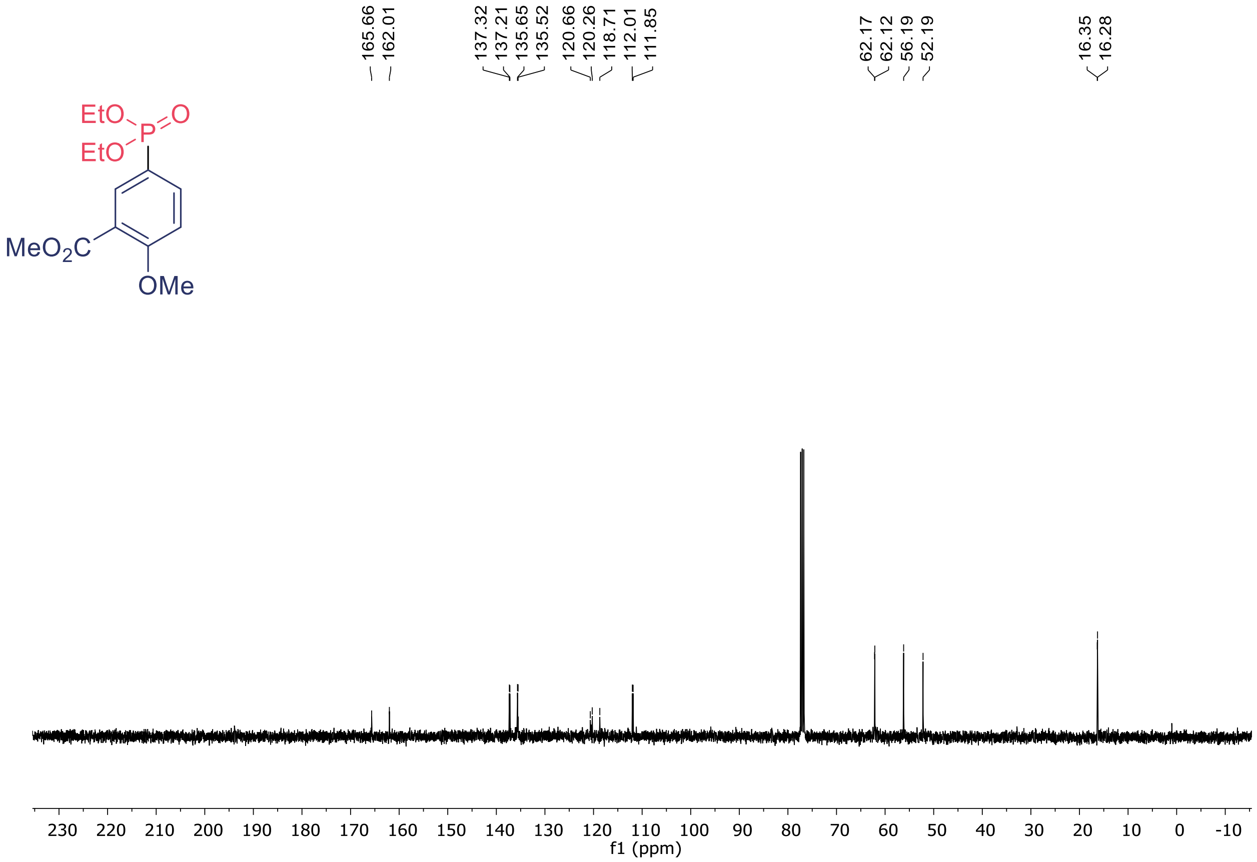

^{13}C NMR (101 MHz, $CDCl_3$) of compound **3**.

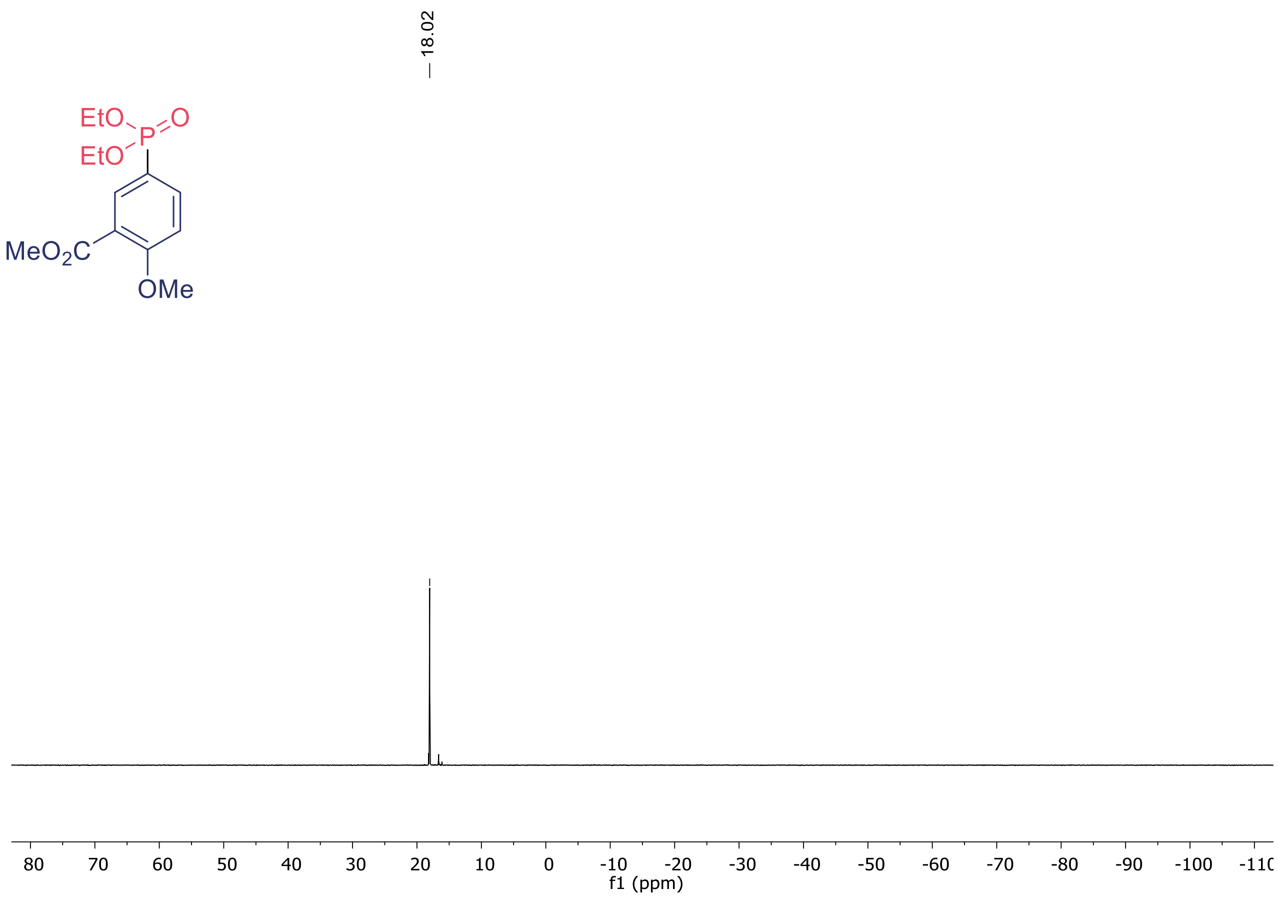

^{31}P NMR (122 MHz, $CDCl_3$) of compound **3**.

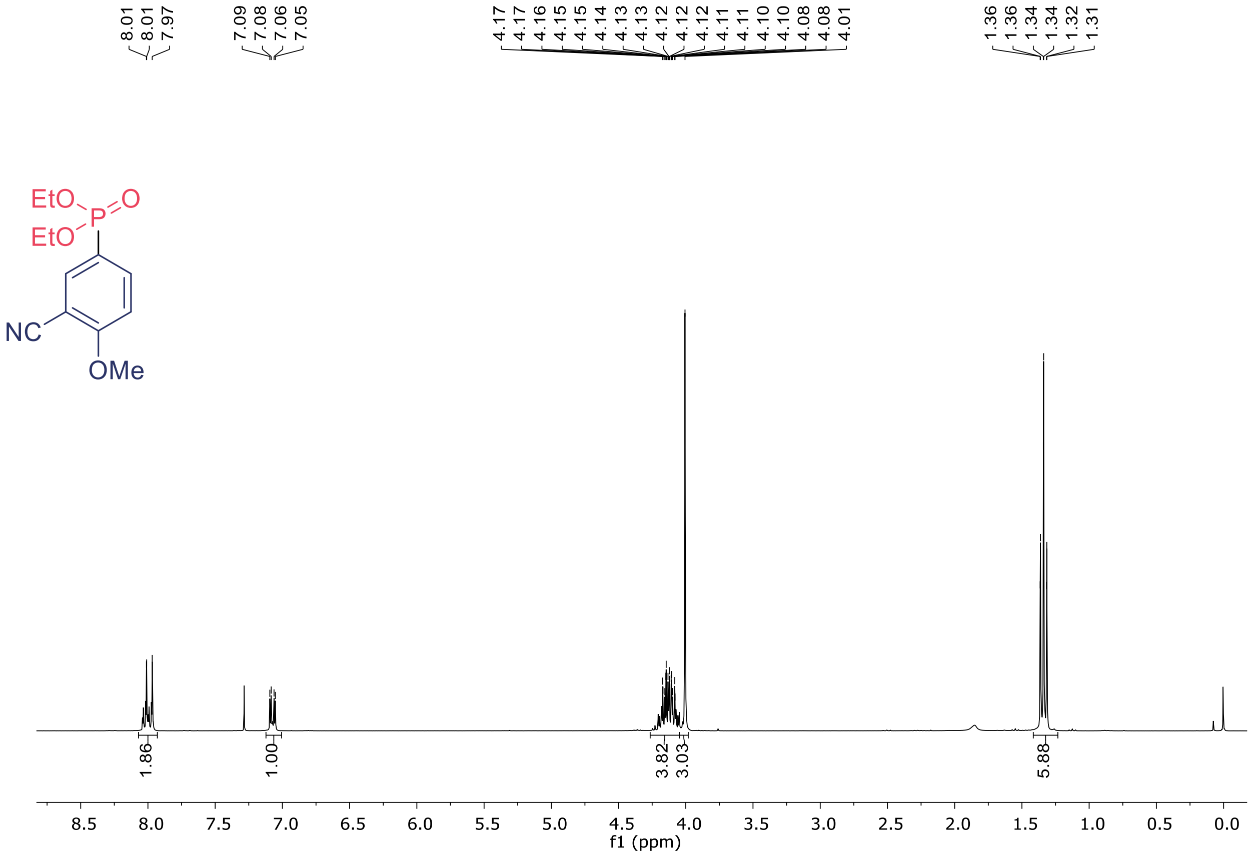

^{1}H NMR (300 MHz, $CDCl_3$) of compound **4**.

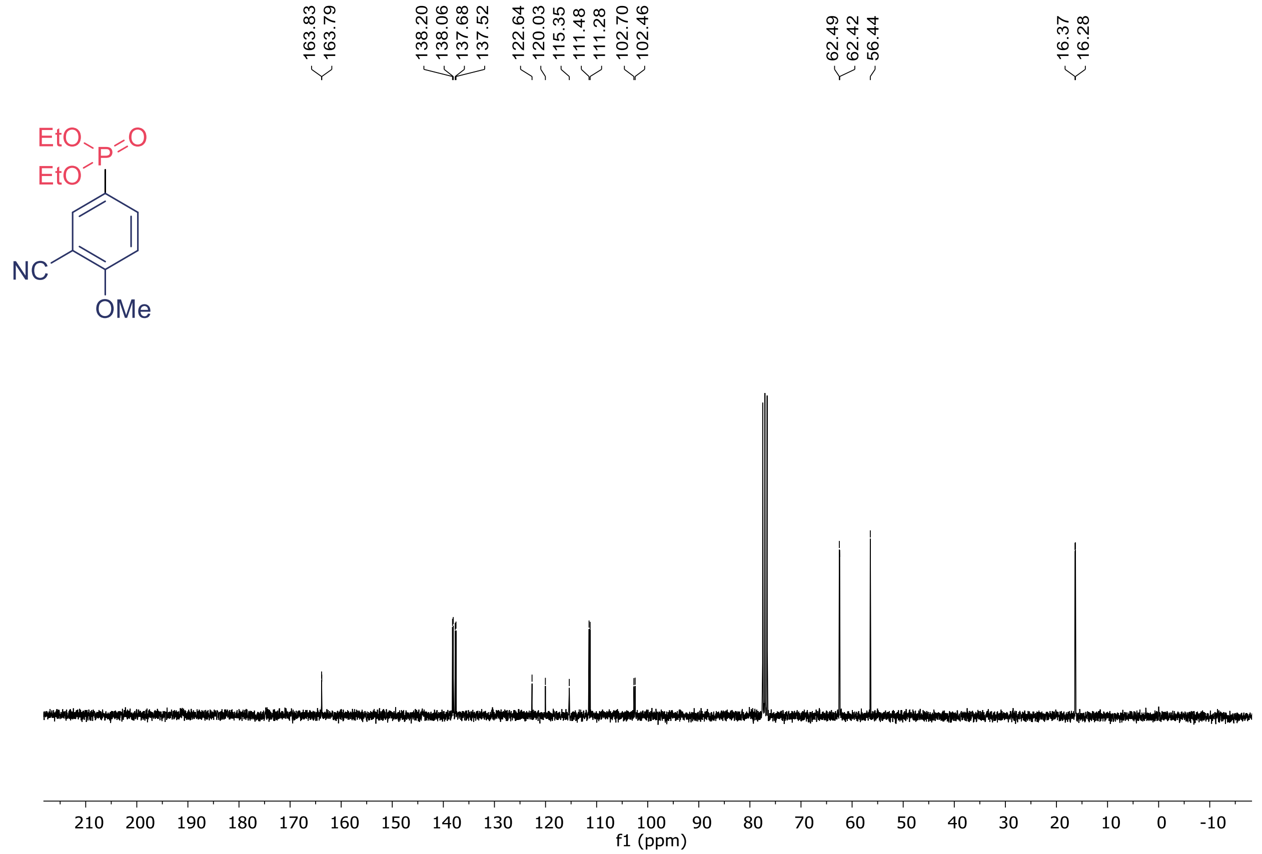

^{13}C NMR (75 MHz, $CDCl_3$) of compound **4**.

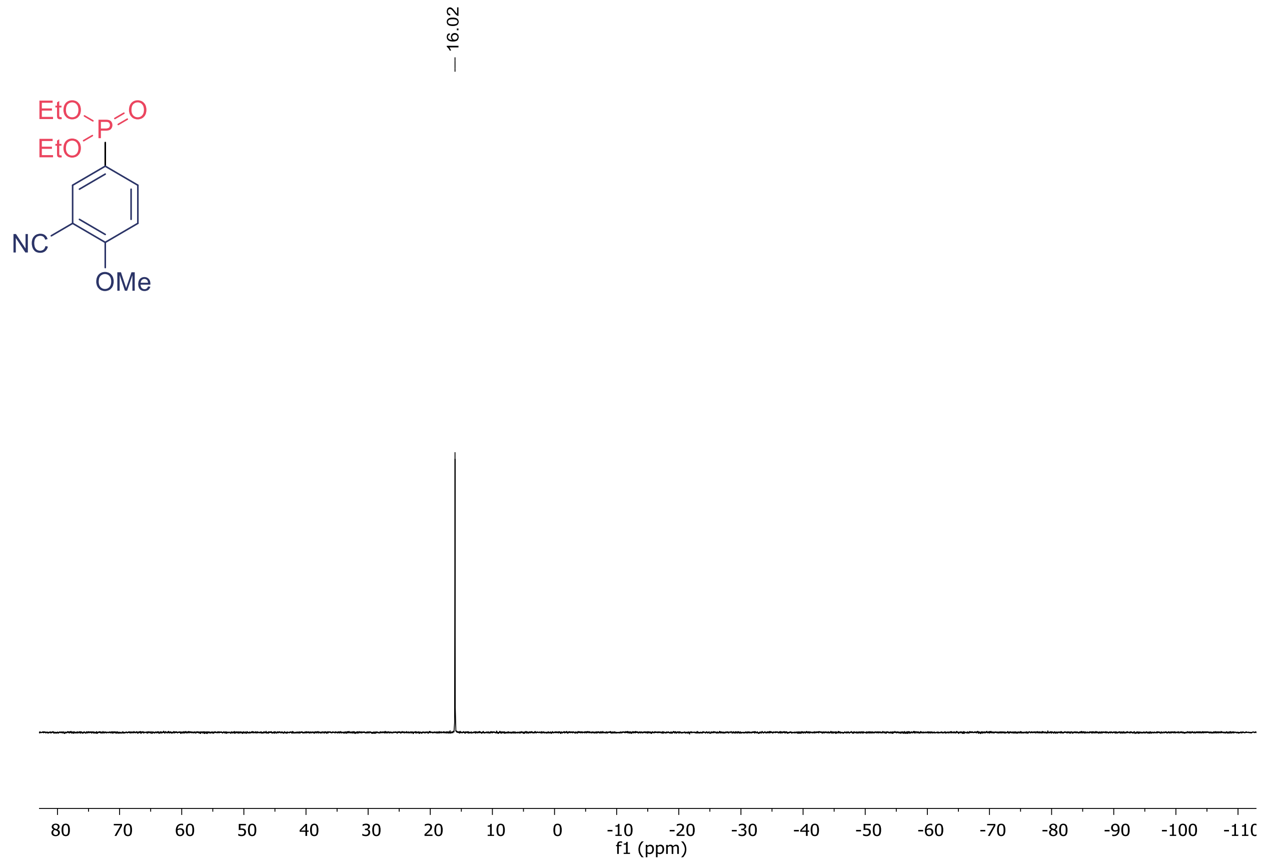

^{31}P NMR (122 MHz, $CDCl_3$) of compound **4**.

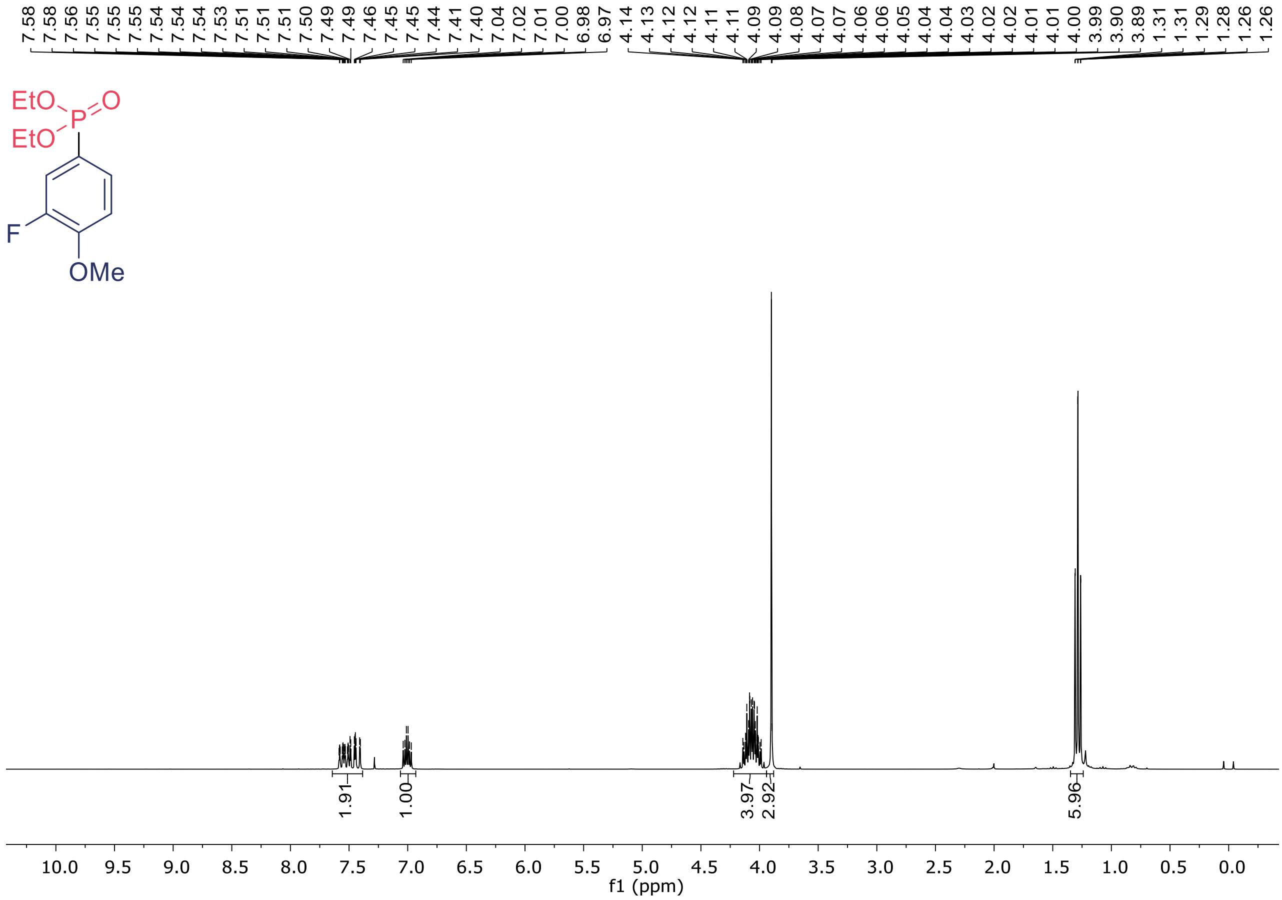

^{1}H NMR (300 MHz, $CDCl_3$) of compound **5**.

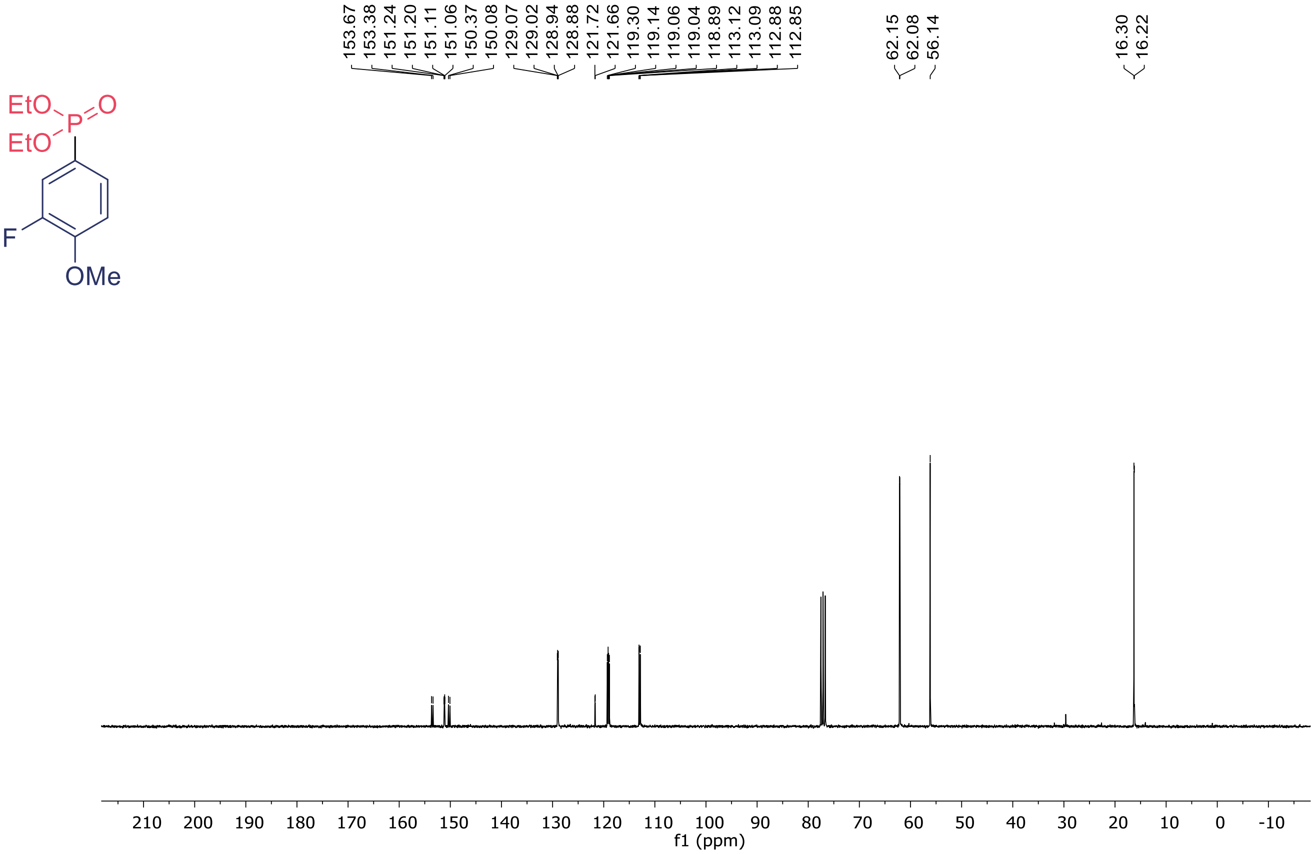

^{13}C NMR (75 MHz, $CDCl_3$) of compound **5**.

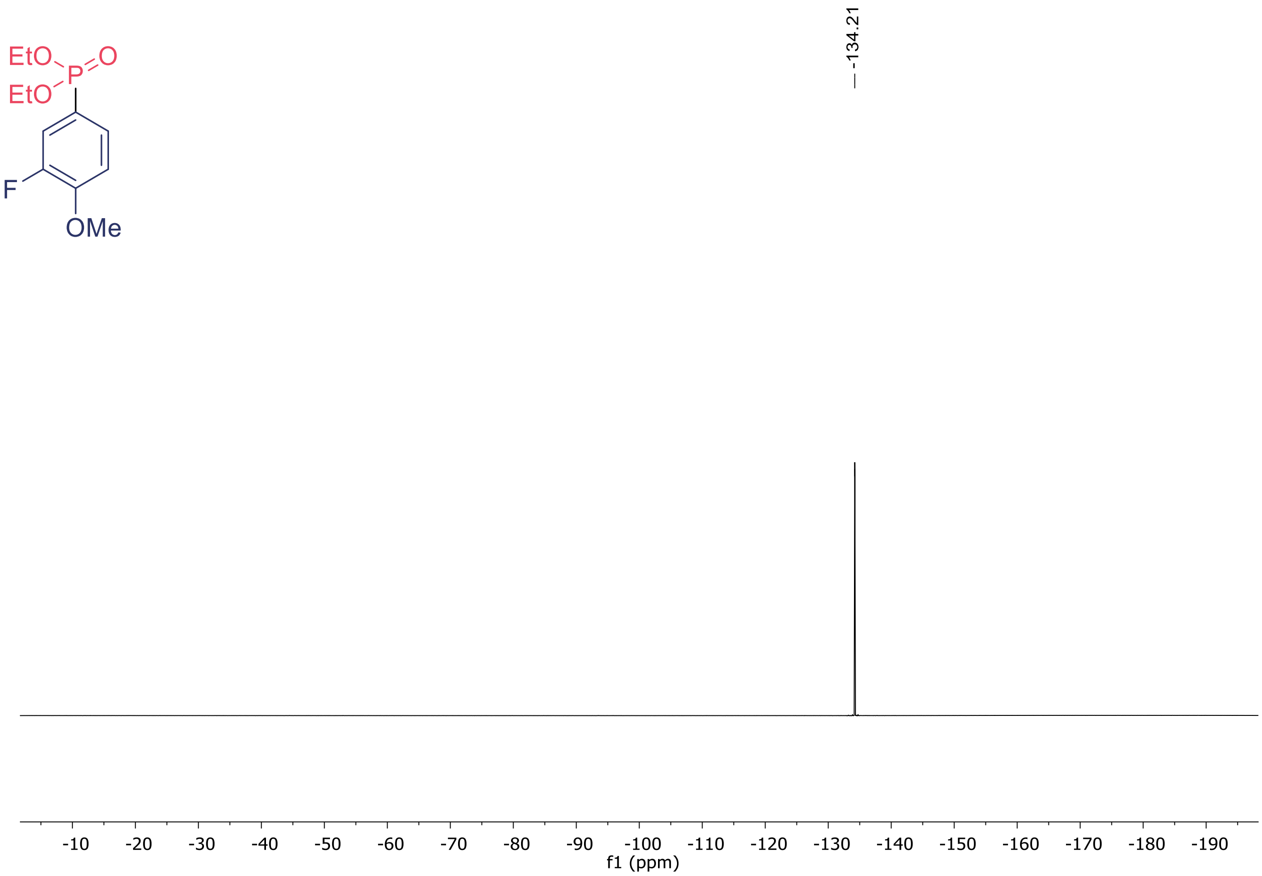

^{19}F NMR (282 MHz, $CDCl_3$) of compound **5**.

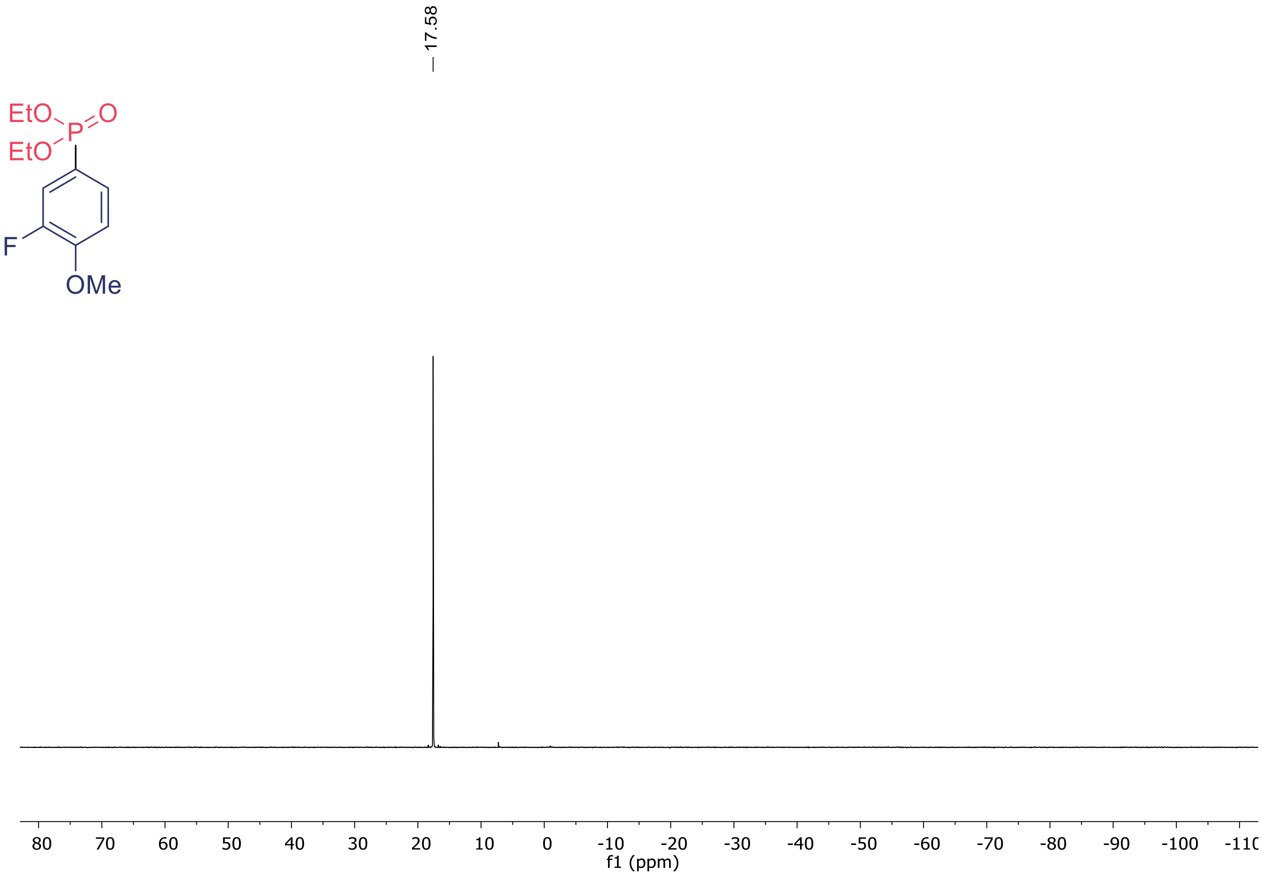

^{31}P NMR (122 MHz, $CDCl_3$) of compound **5**.

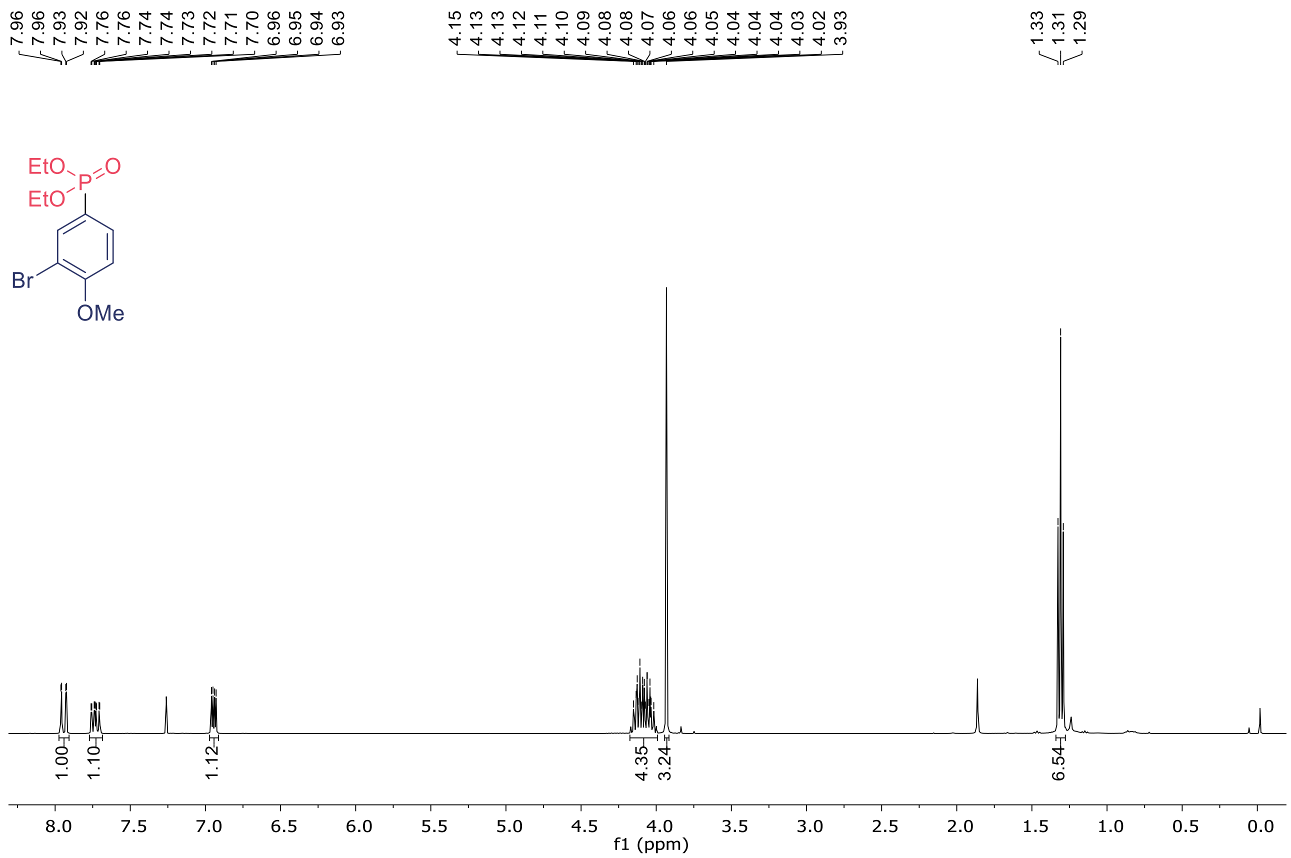

^{1}H NMR (400 MHz, $CDCl_3$) of compound **6**.

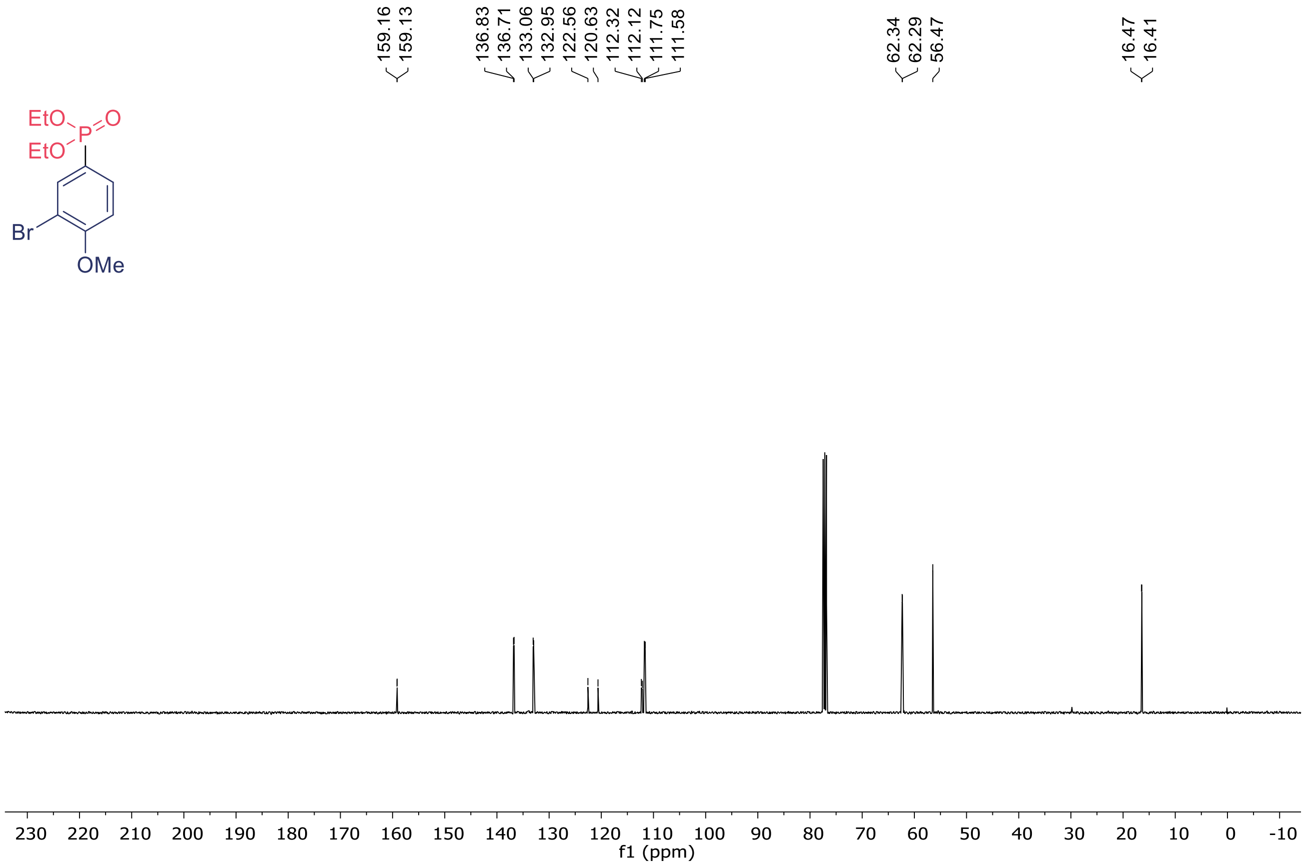

^{13}C NMR (101 MHz, $CDCl_3$) of compound **6**.

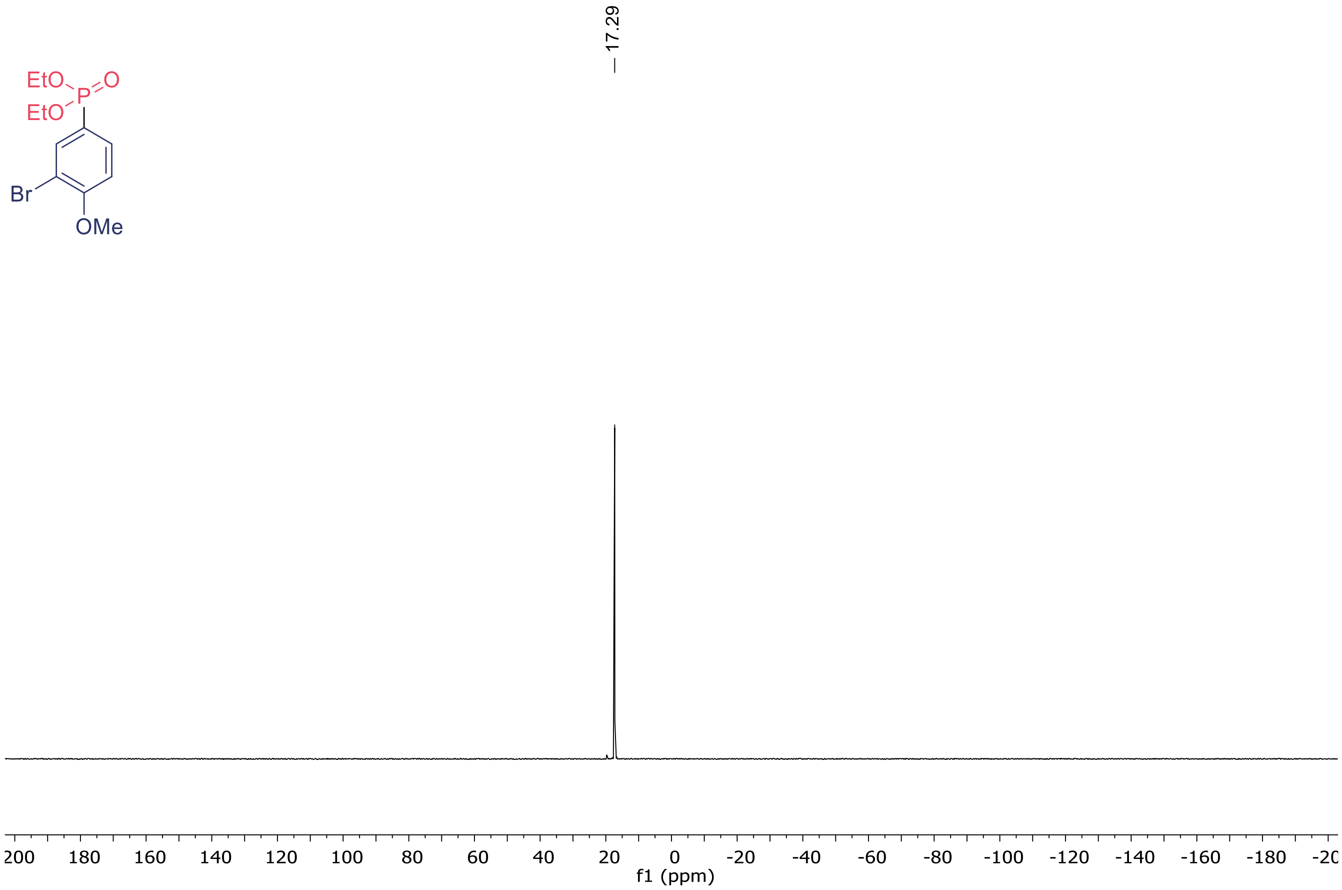

^{31}P NMR (162 MHz, $CDCl_3$) of compound **6**.

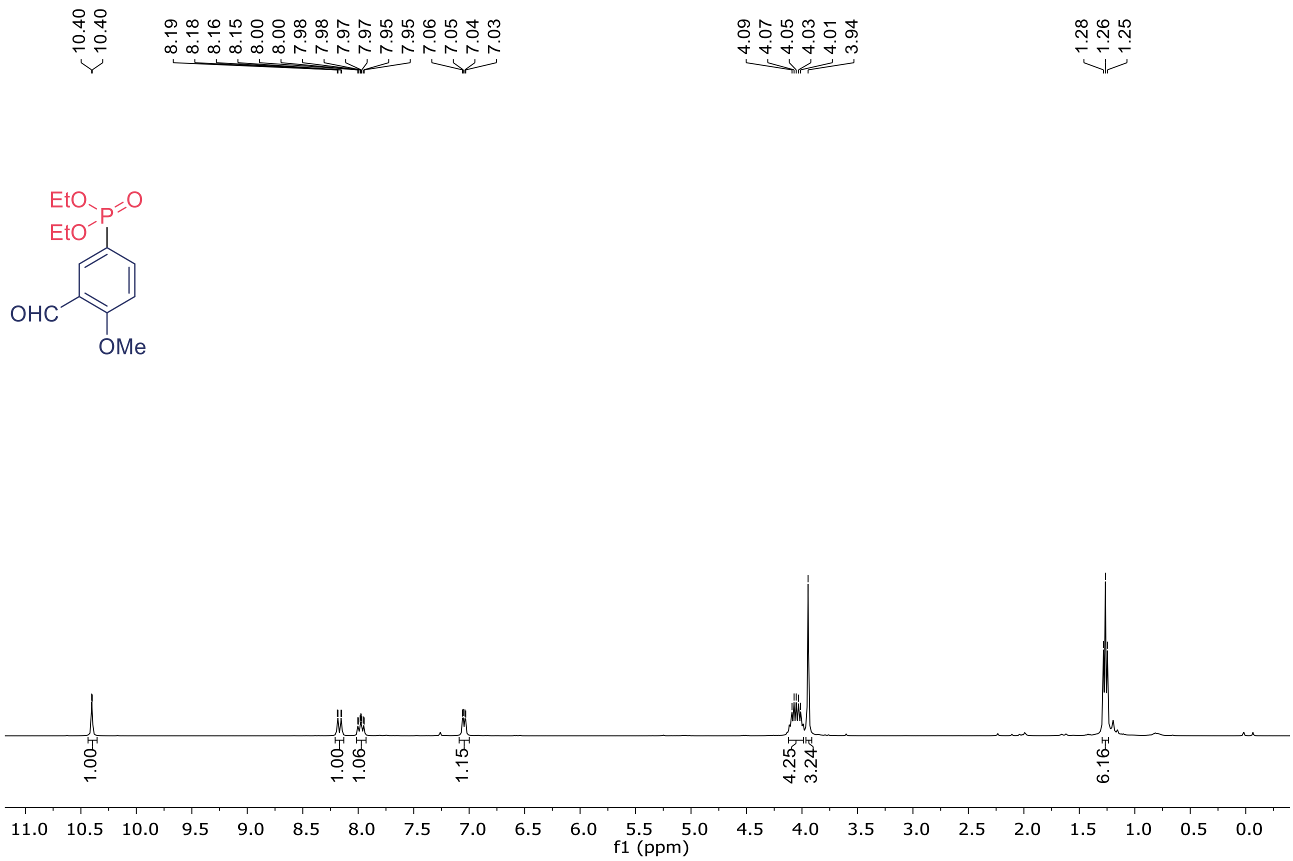

^{1}H NMR (400 MHz, $CDCl_3$) of compound **7**.

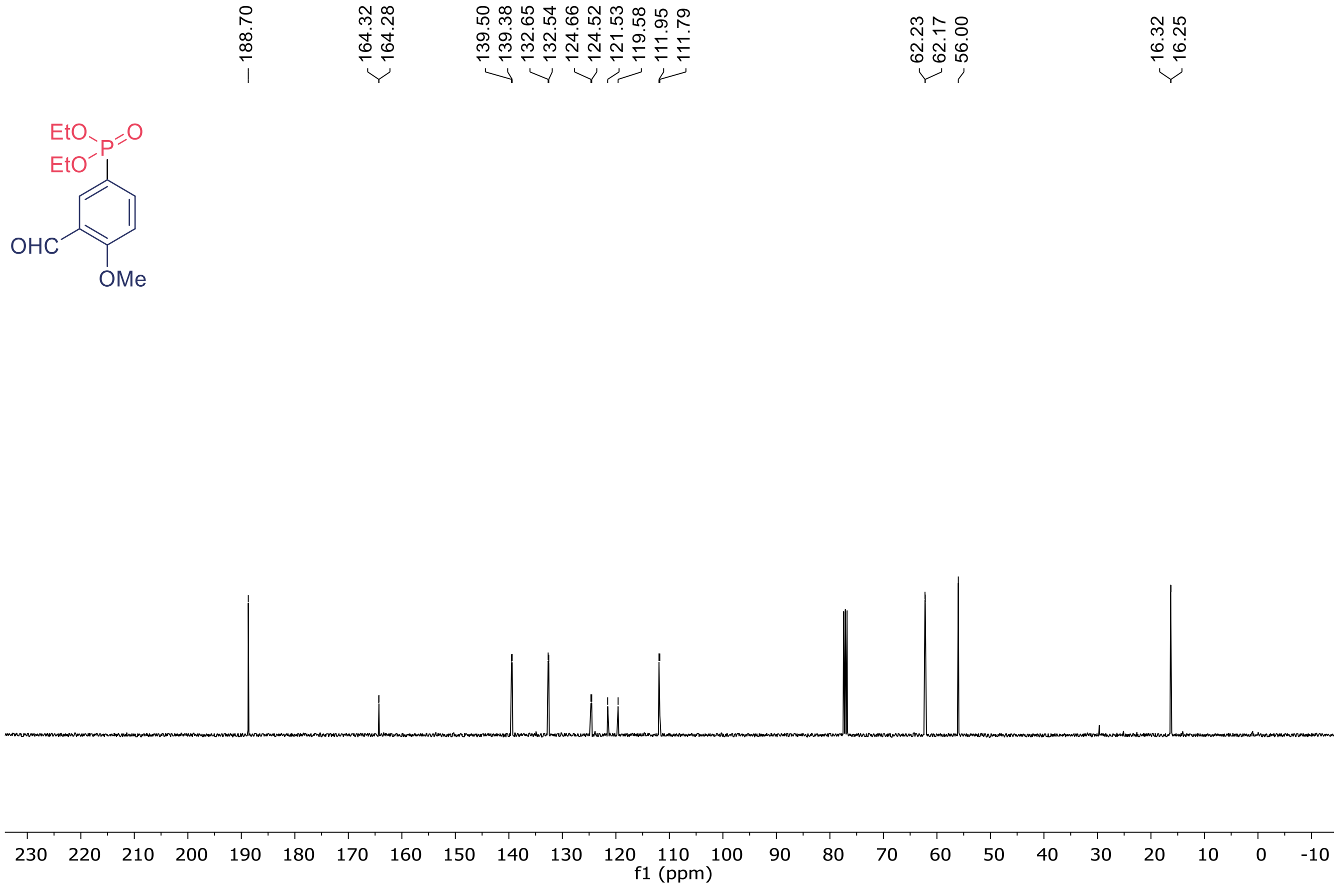

^{13}C NMR (101 MHz, $CDCl_3$) of compound **7**.

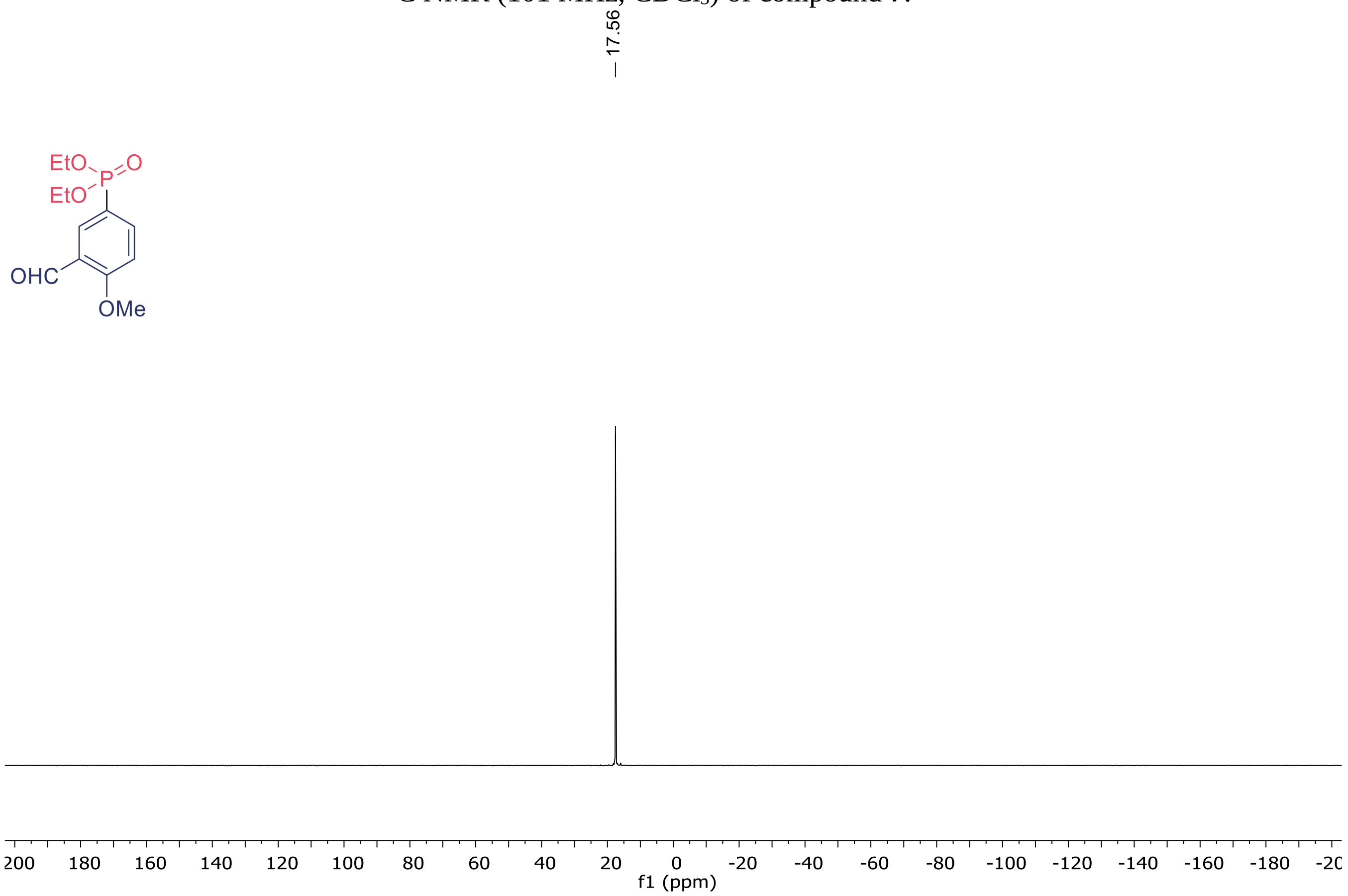

^{31}P NMR (162 MHz, $CDCl_3$) of compound **7**.

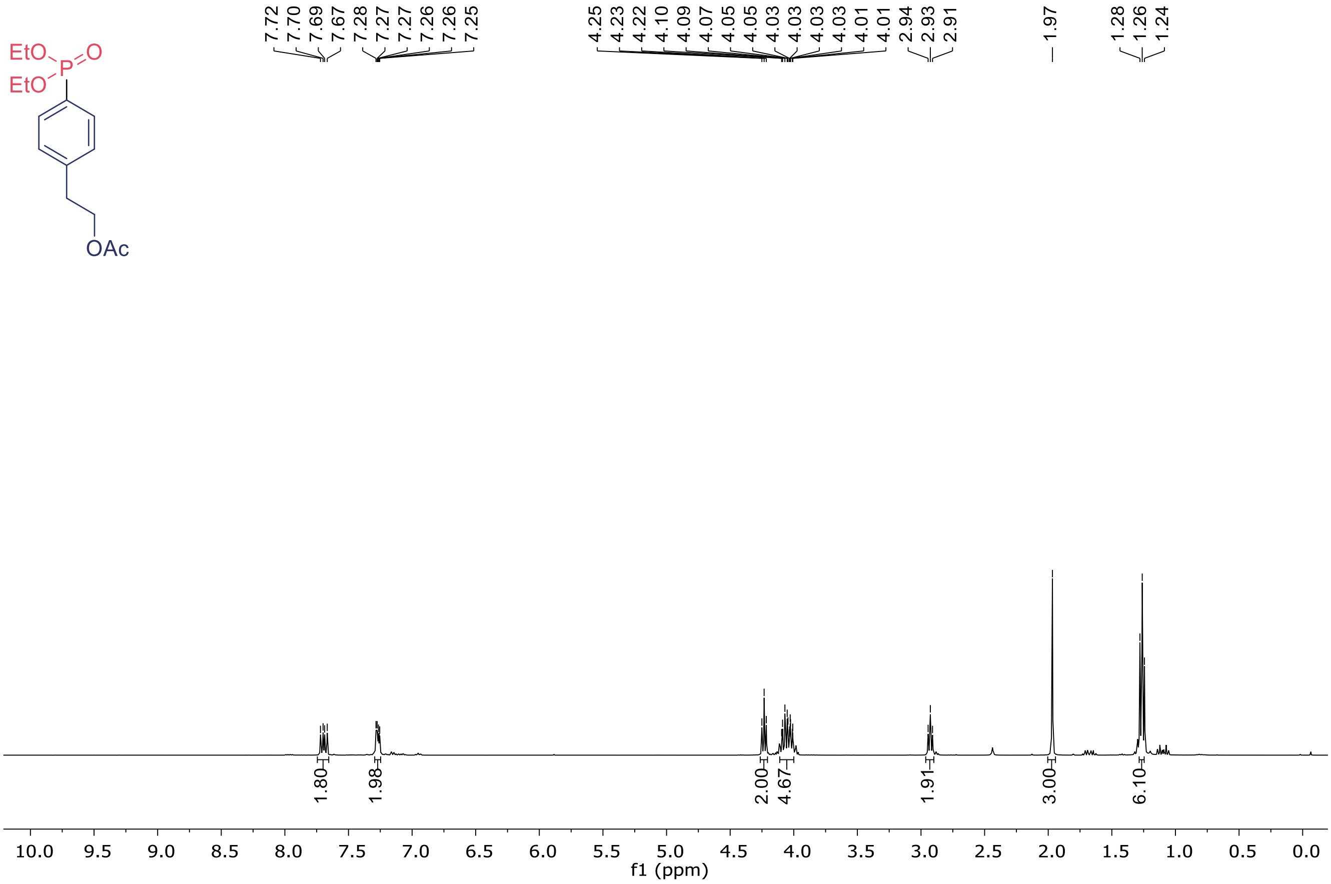

^{1}H NMR (400 MHz, $CDCl_3$) of compound **8**.

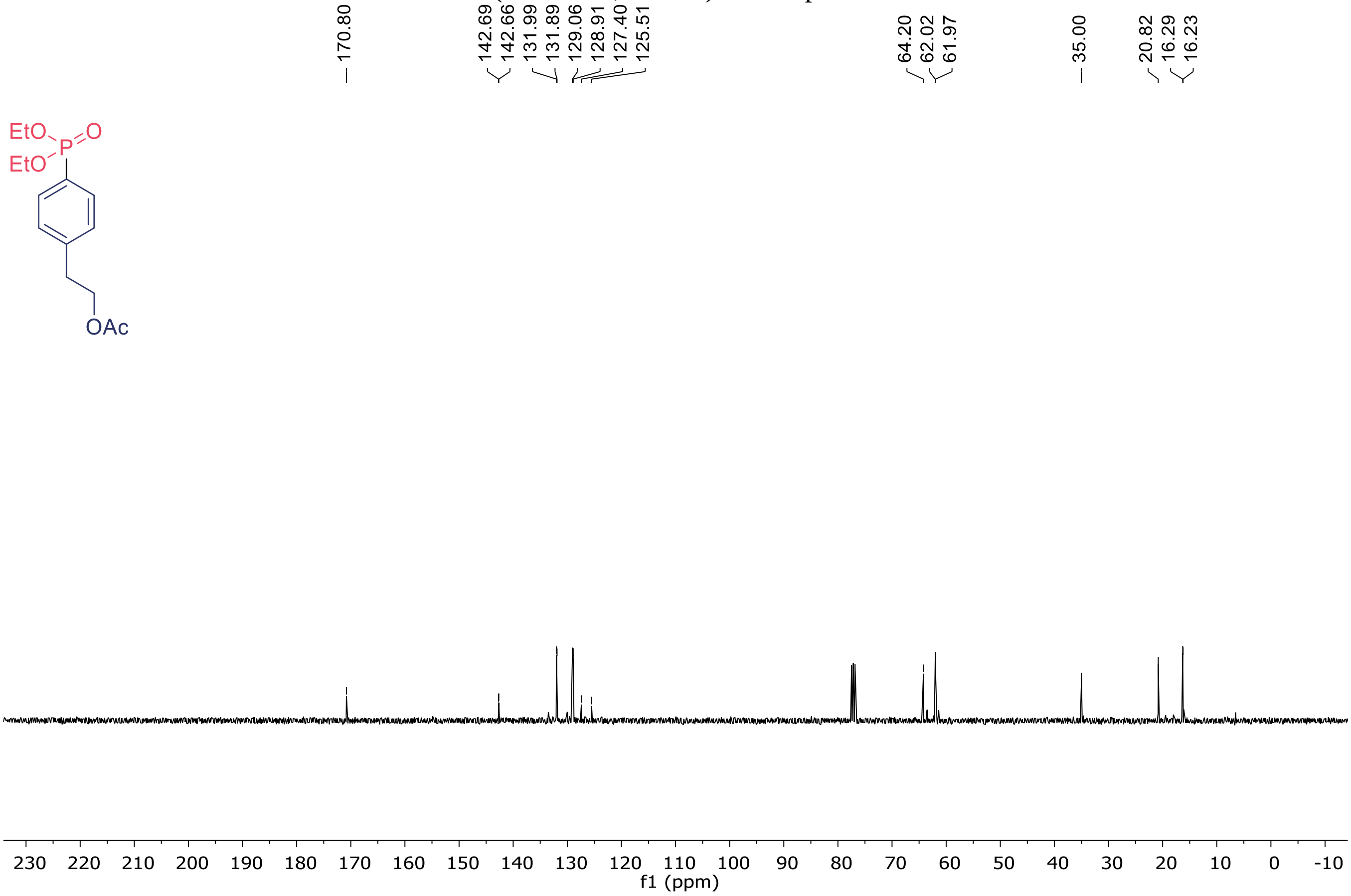

^{13}C NMR (101 MHz, $CDCl_3$) of compound **8**.

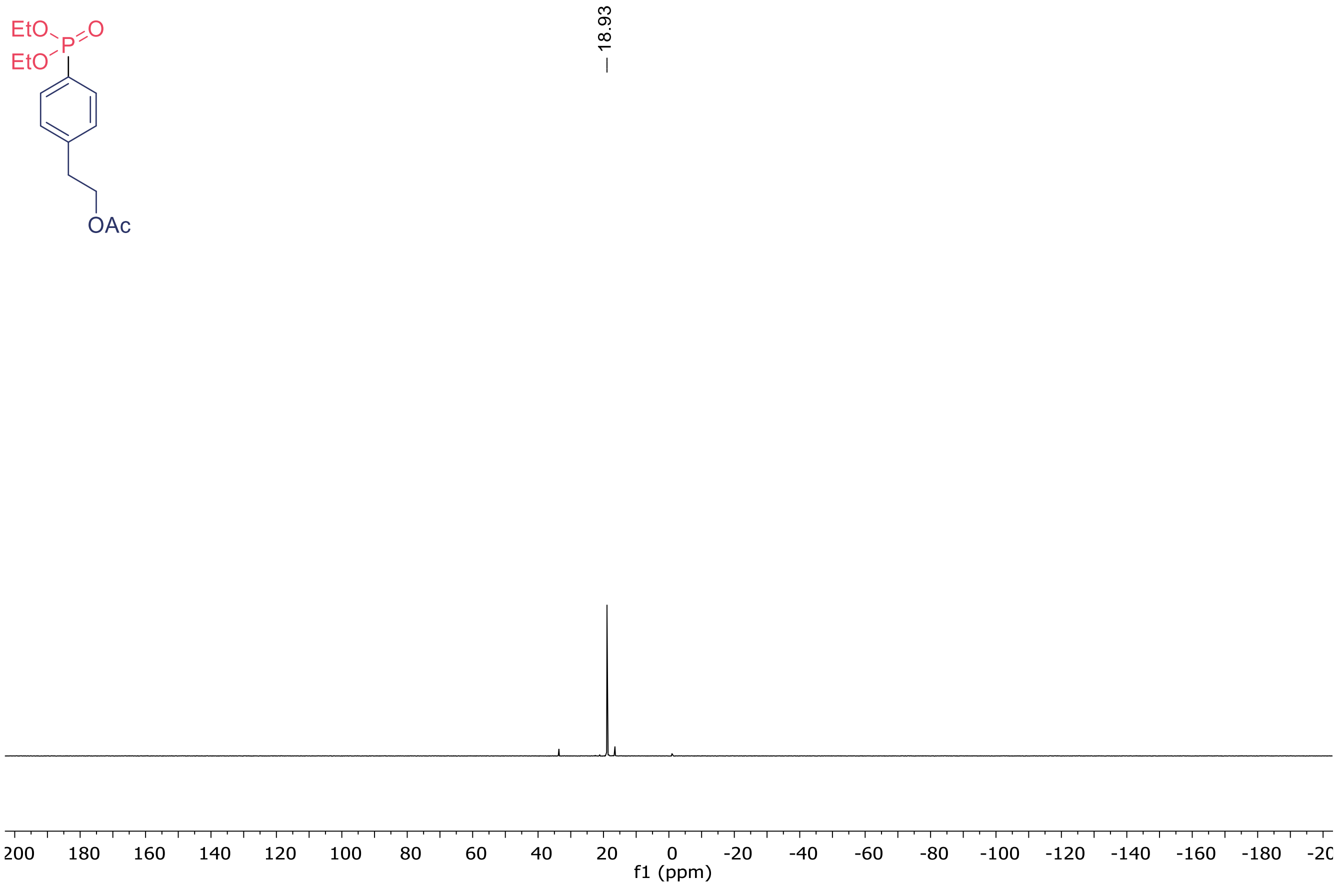

^{31}P NMR (162 MHz, $CDCl_3$) of compound **8**.

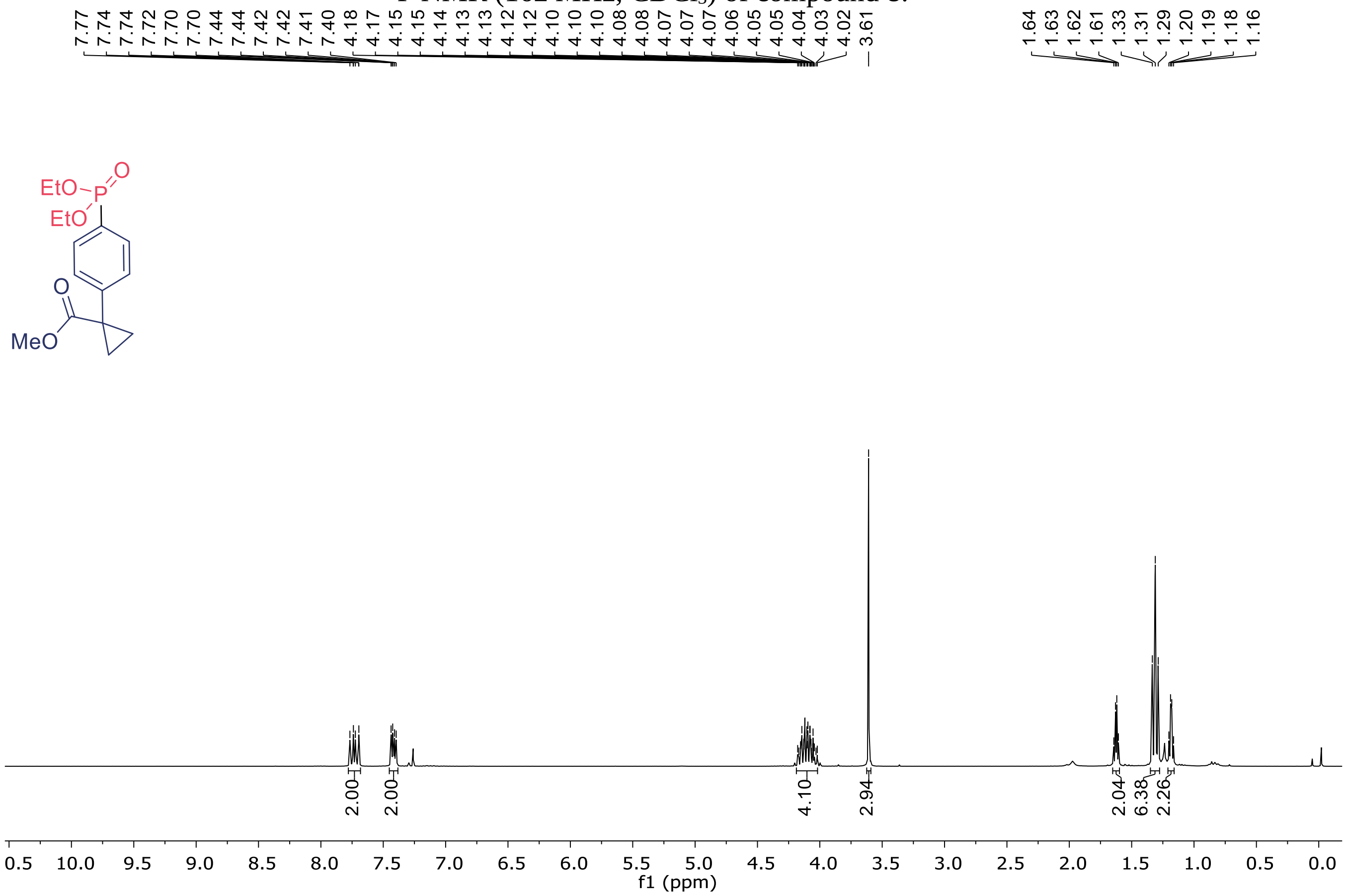

^{1}H NMR (300 MHz, $CDCl_3$) of compound **9**.

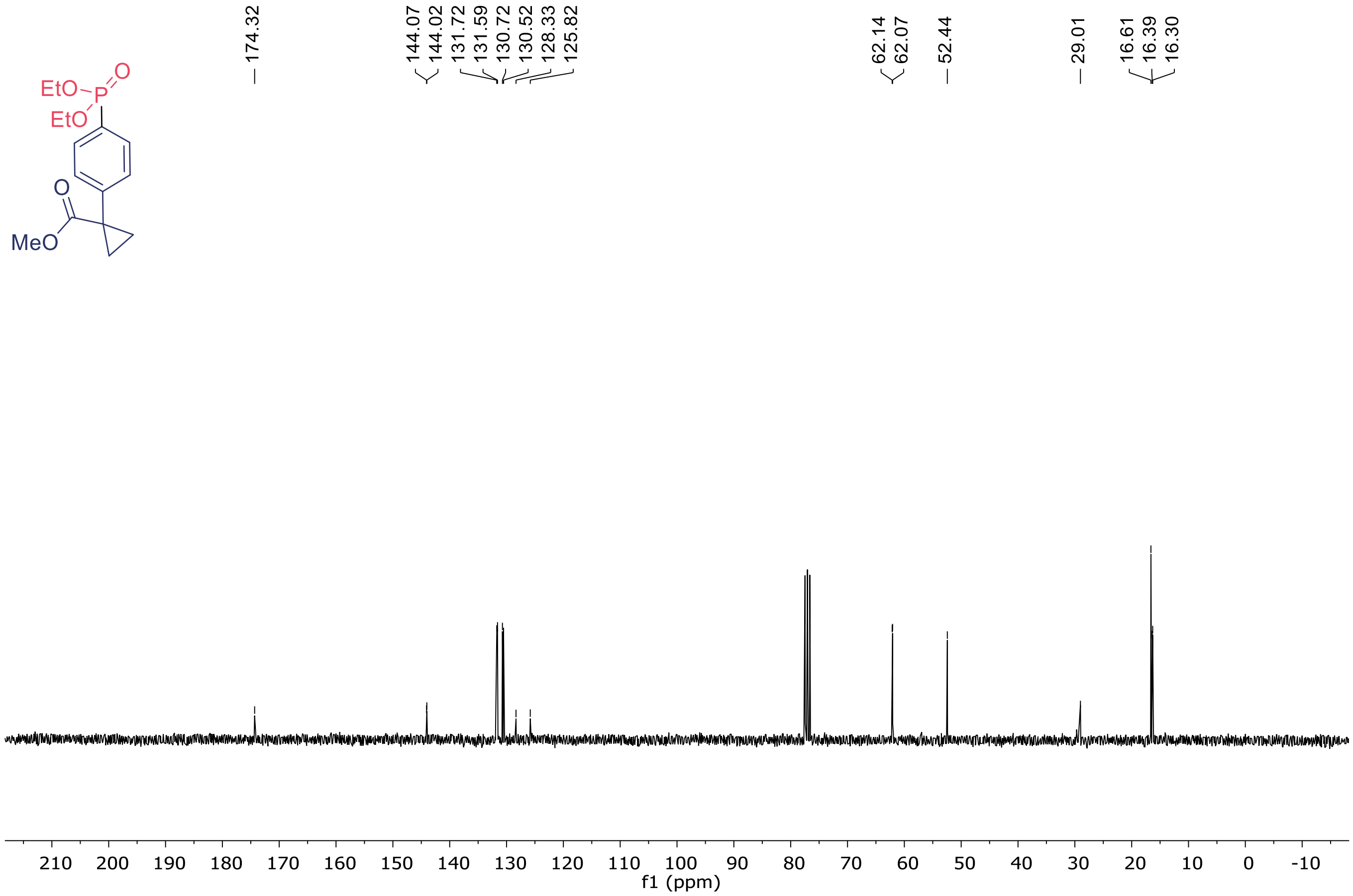

^{13}C NMR (75 MHz, $CDCl_3$) of compound **9**.

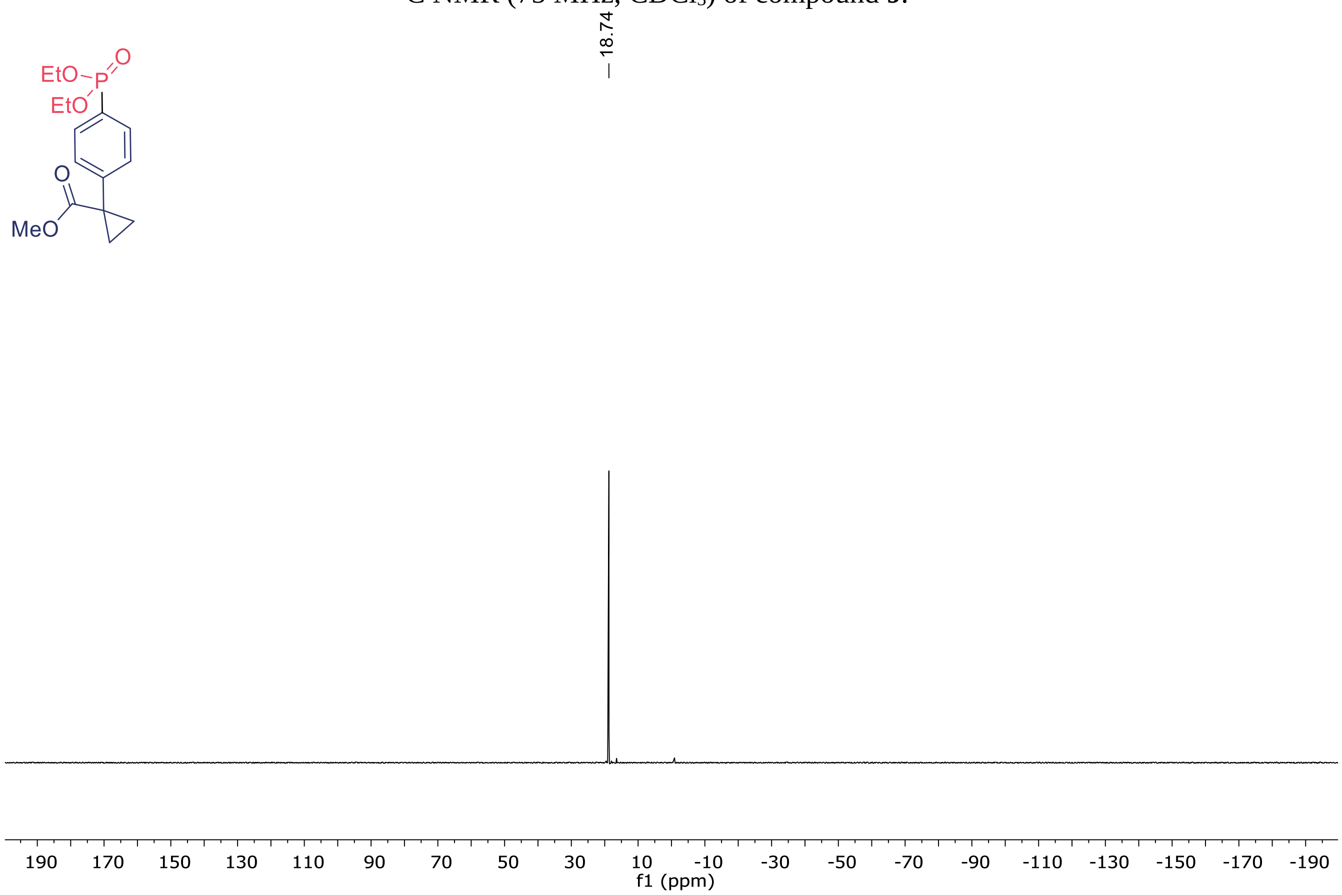

^{31}P NMR (122 MHz, $CDCl_3$) of compound **9**.

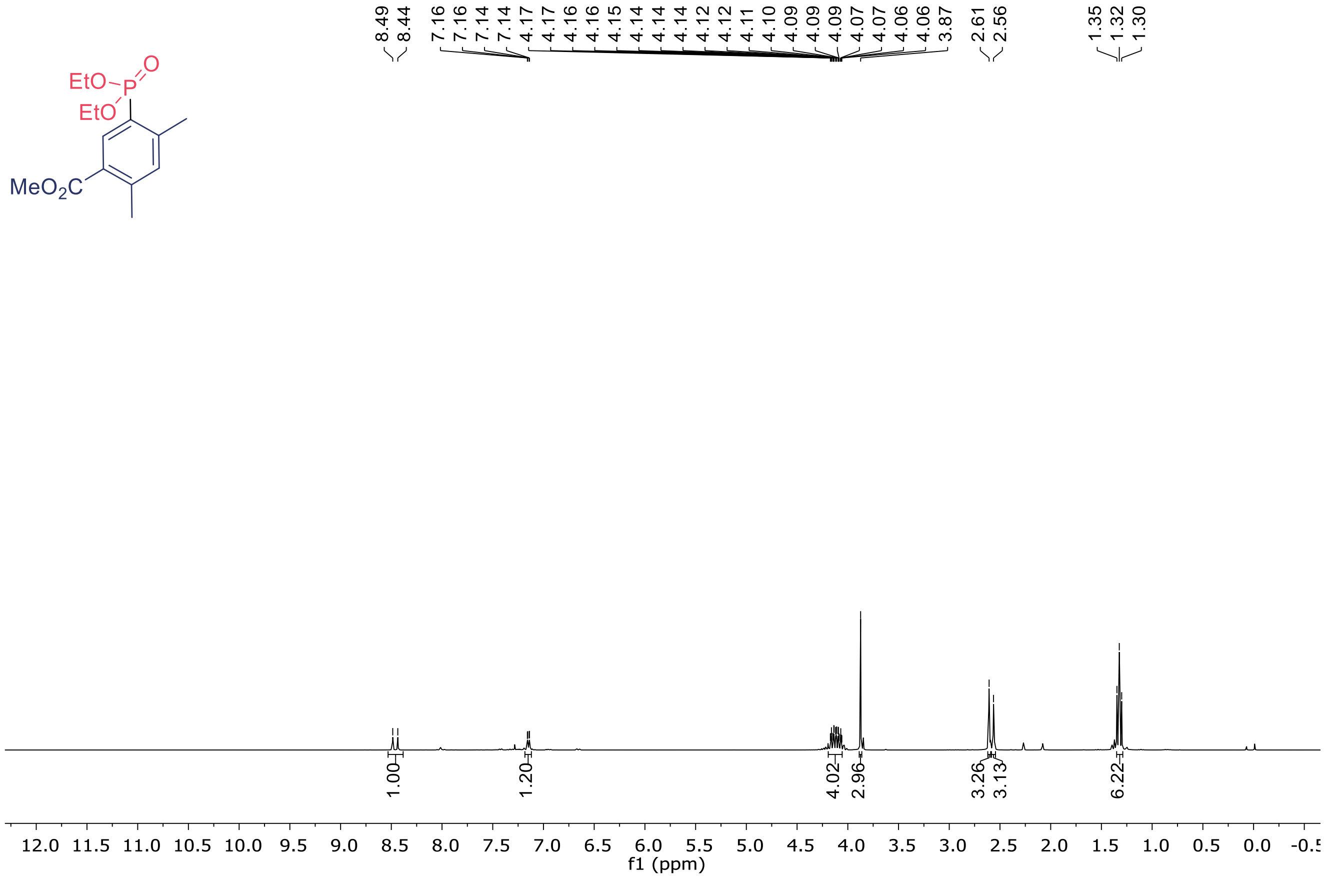

^{1}H NMR (300 MHz, $CDCl_3$) of compound **10**.

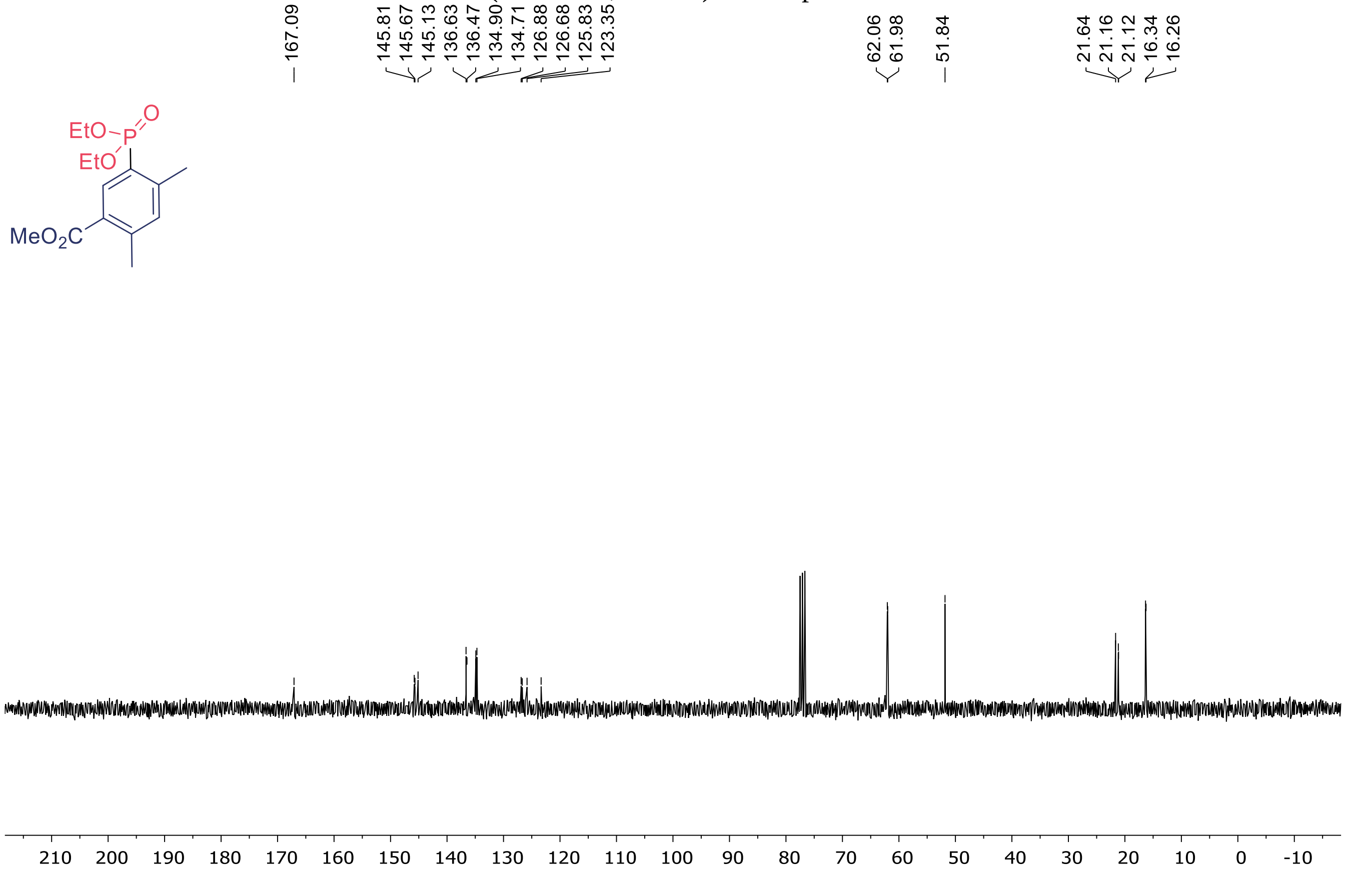

^{13}C NMR (75 MHz, $CDCl_3$) of compound **10**.

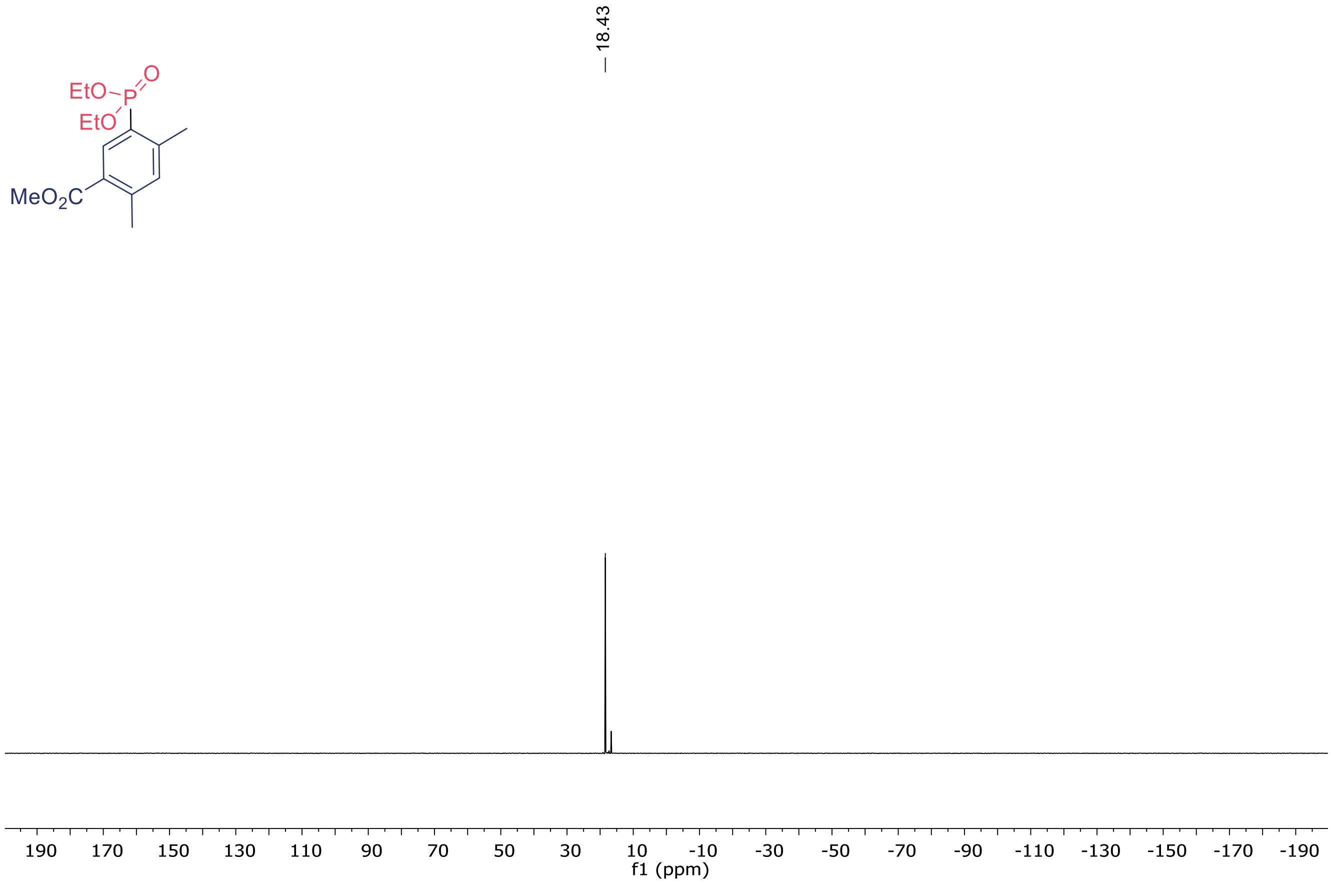

^{31}P NMR (122 MHz, $CDCl_3$) of compound **10**.

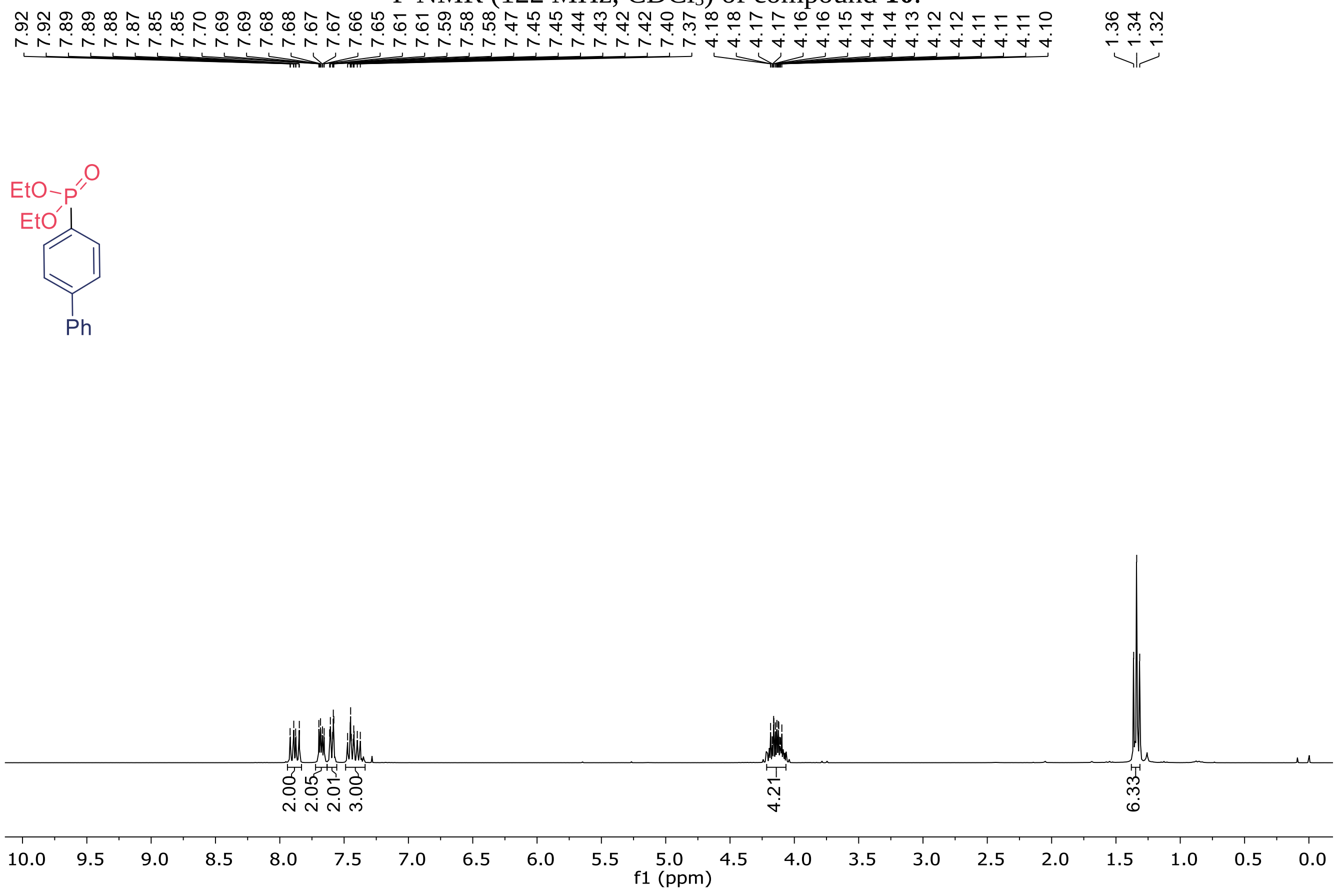

^{1}H NMR (300 MHz, $CDCl_3$) of compound **11**.

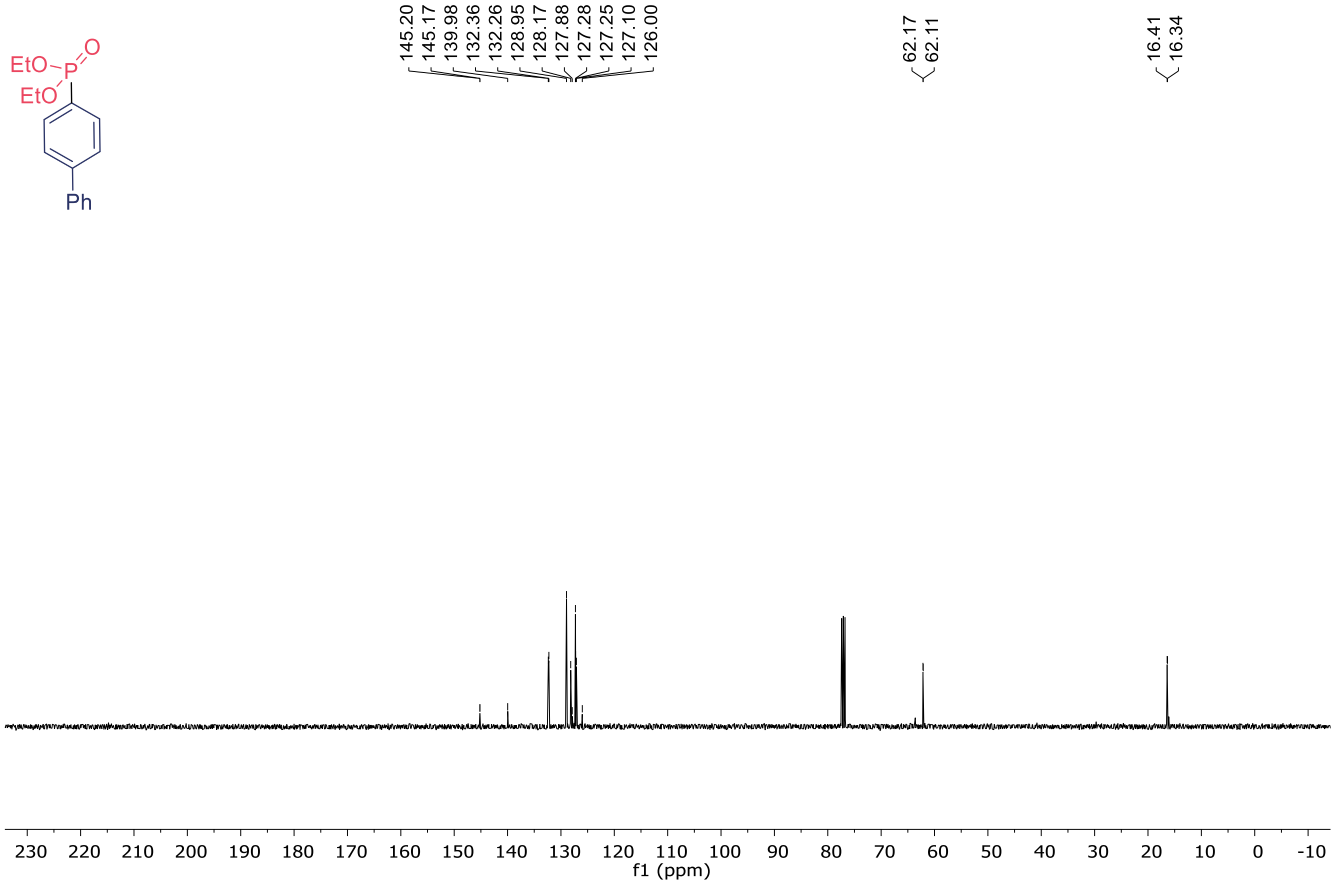

^{13}C NMR (75 MHz, $CDCl_3$) of compound **11**.

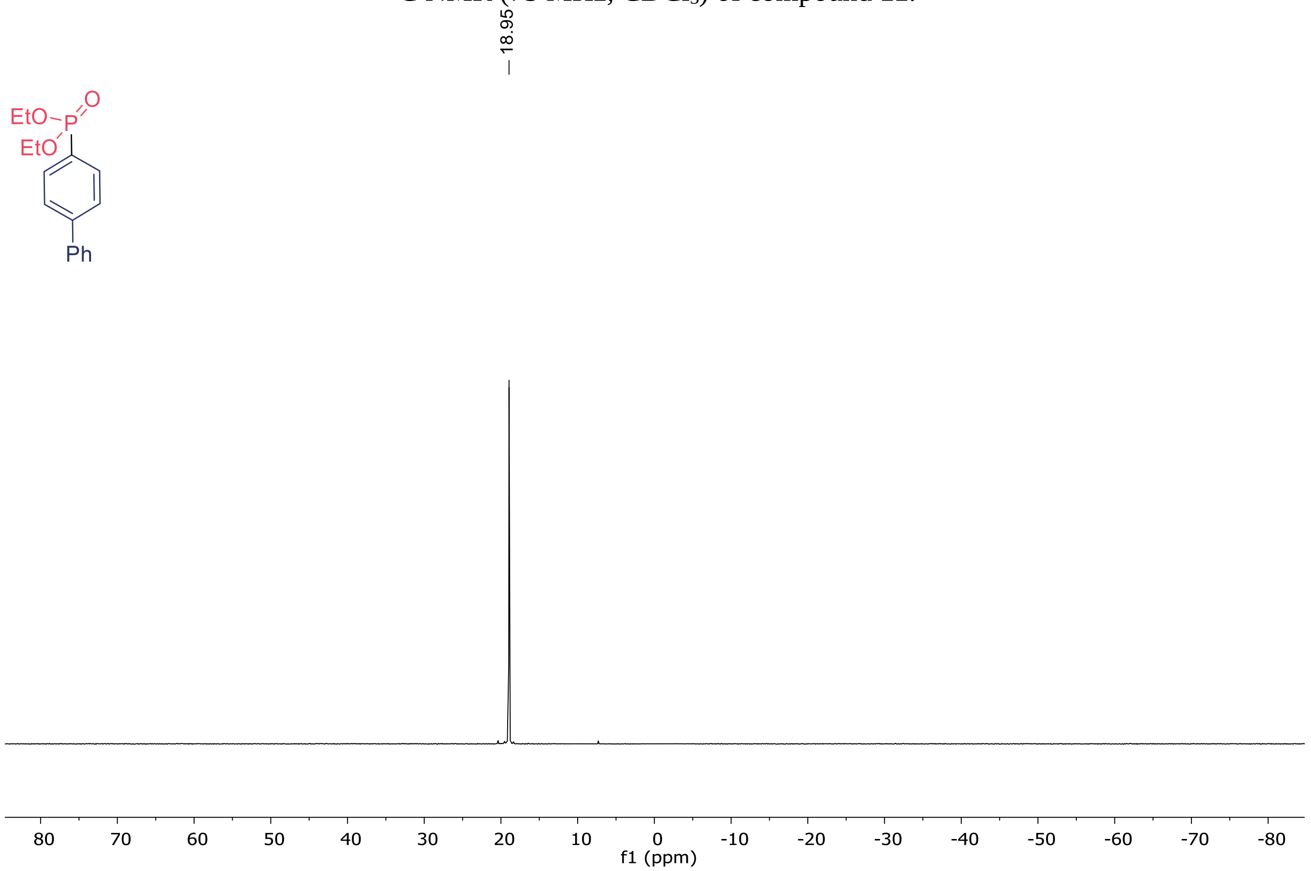

^{31}P NMR (122 MHz, $CDCl_3$) of compound **11**.

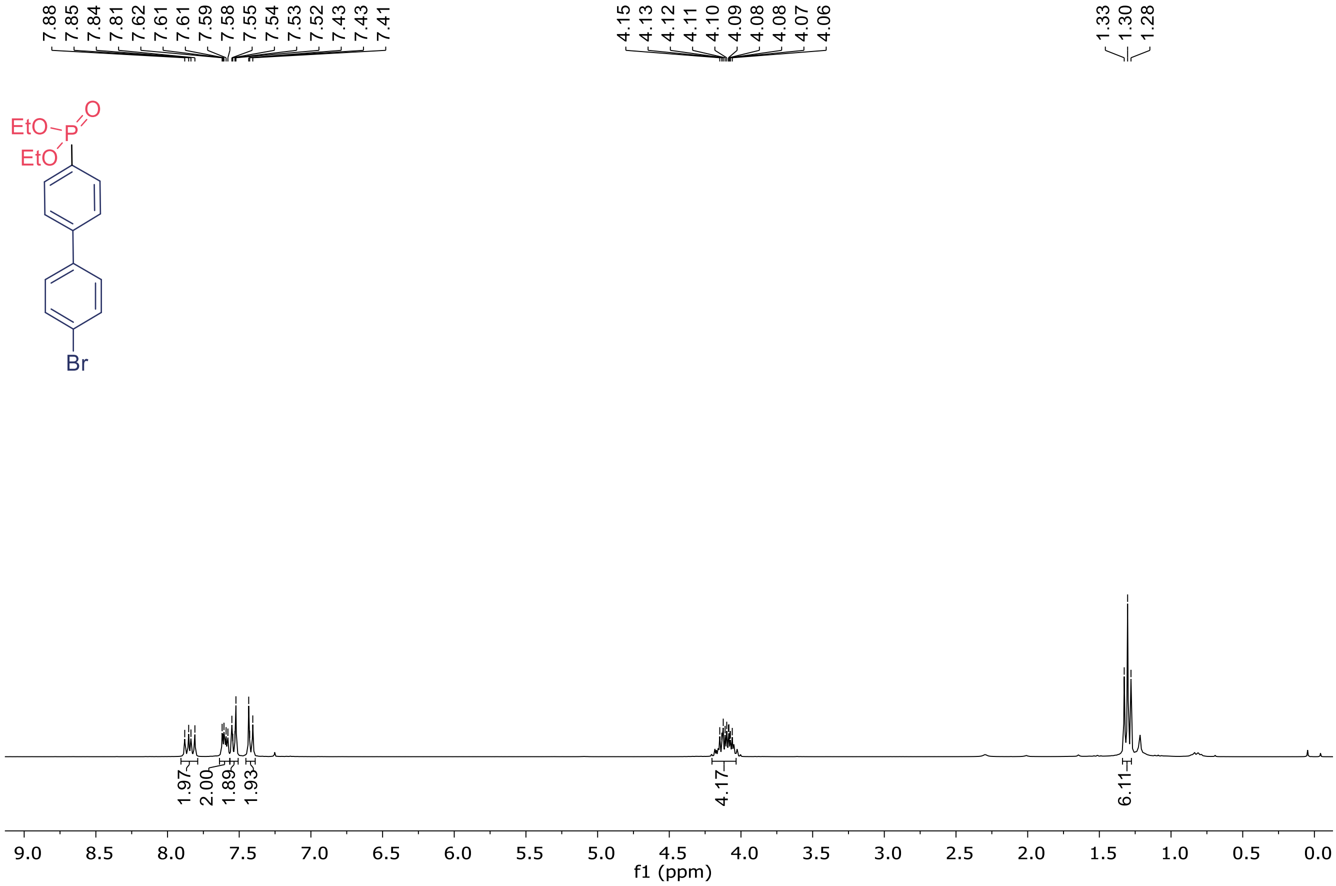

^{1}H NMR (300 MHz, $CDCl_3$) of compound **12**.

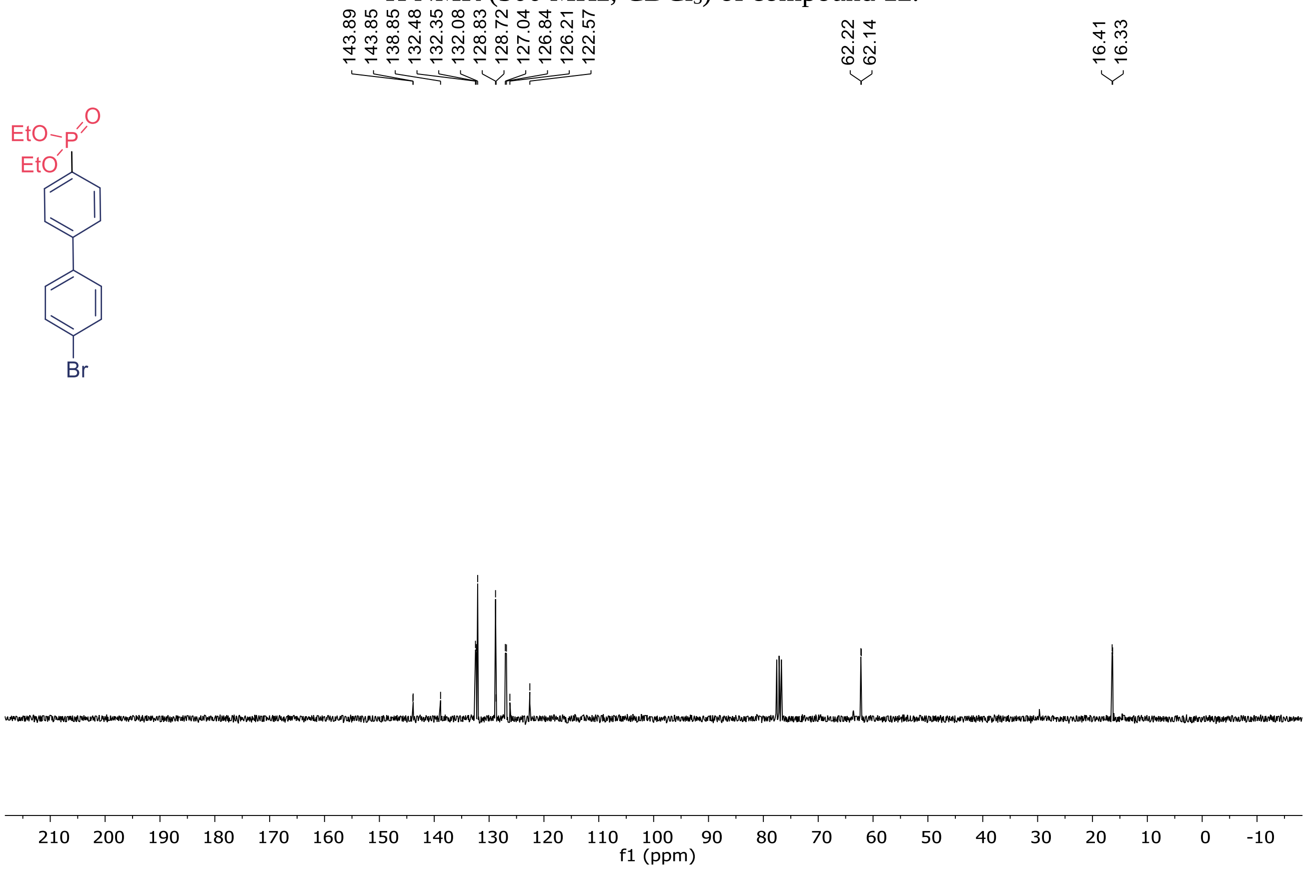

^{13}C NMR (75 MHz, $CDCl_3$) of compound **12**.

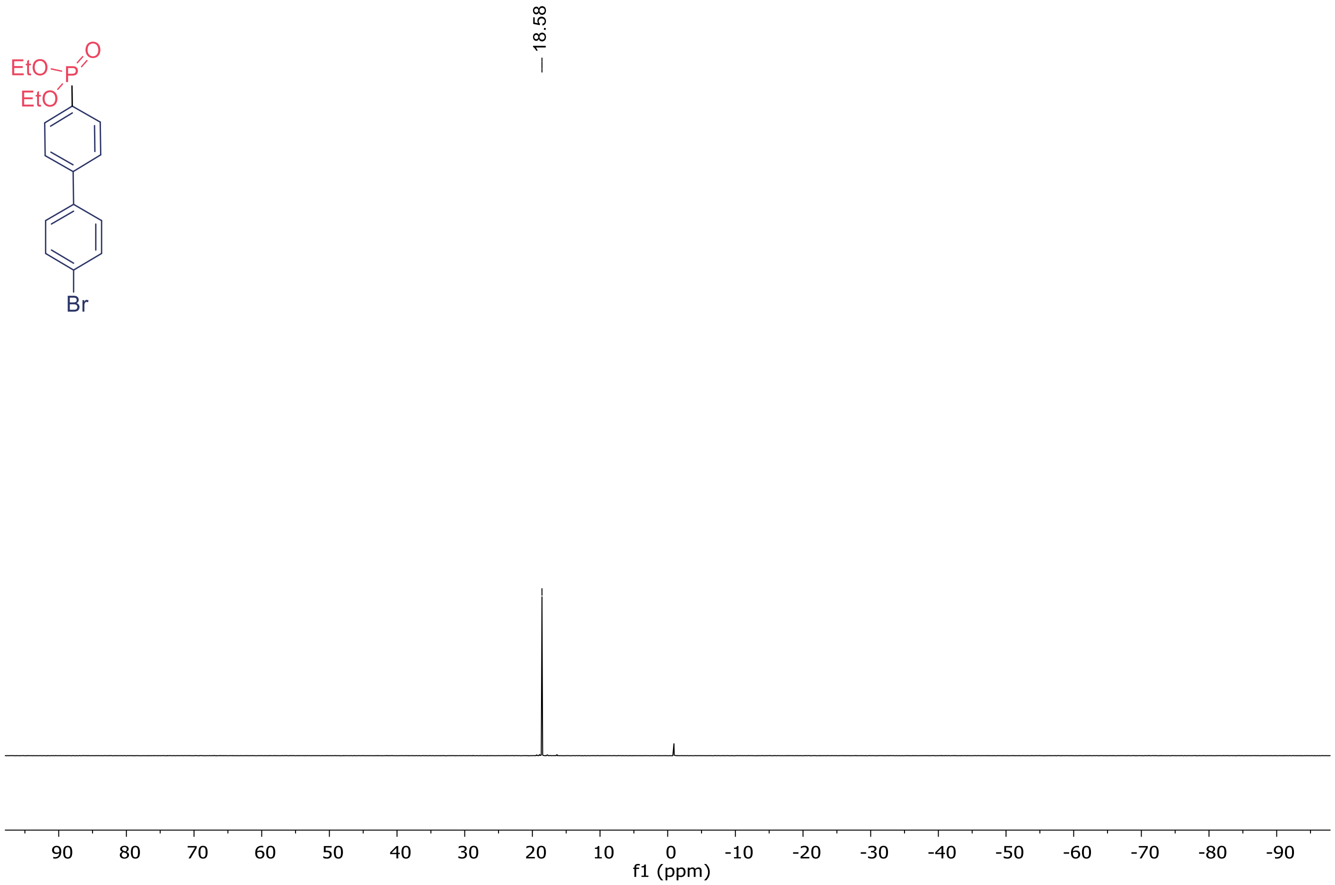

^{31}P NMR (122 MHz, $CDCl_3$) of compound **12**.

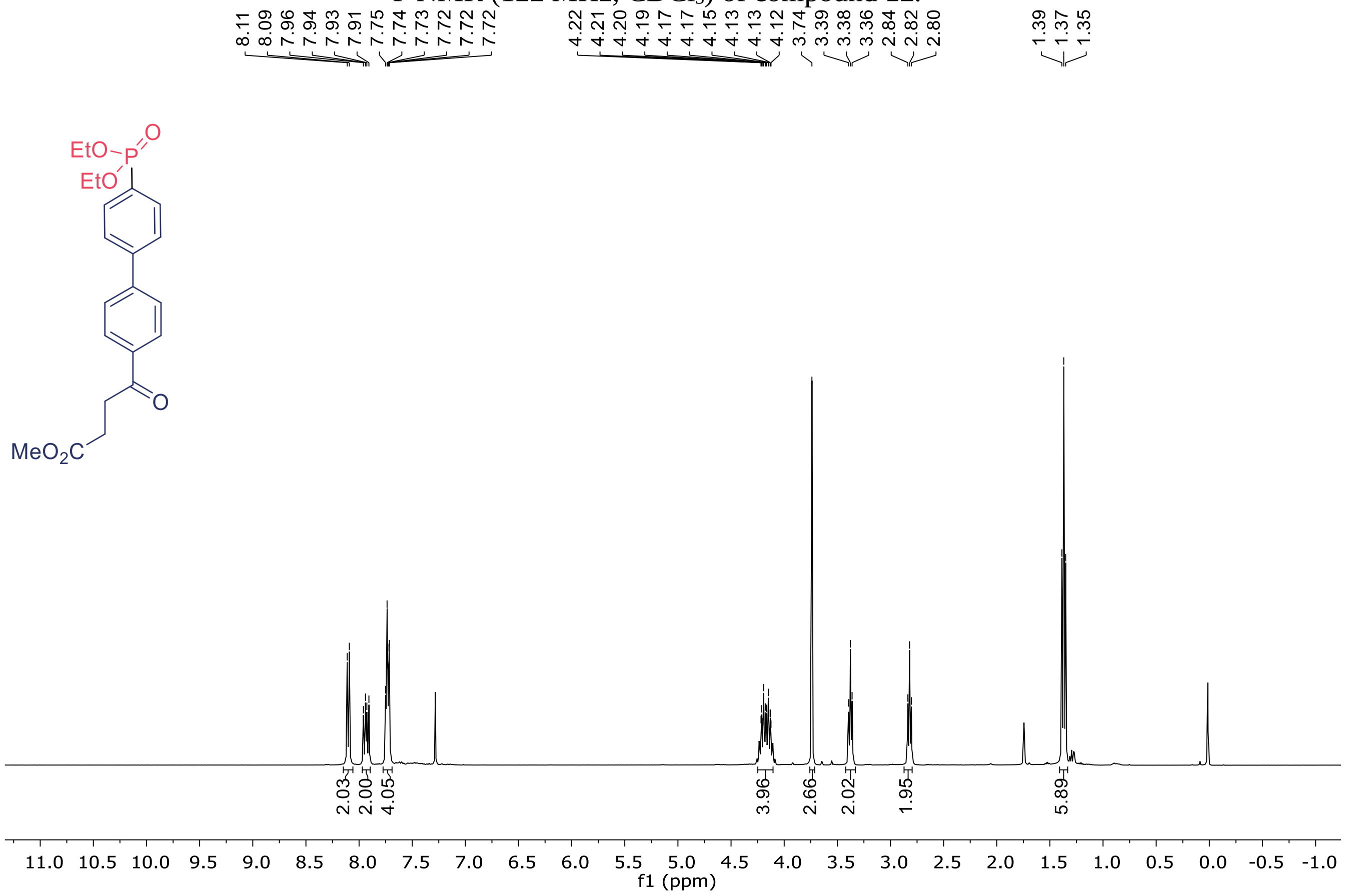

^{1}H NMR (400 MHz, $CDCl_3$) of compound **13**.

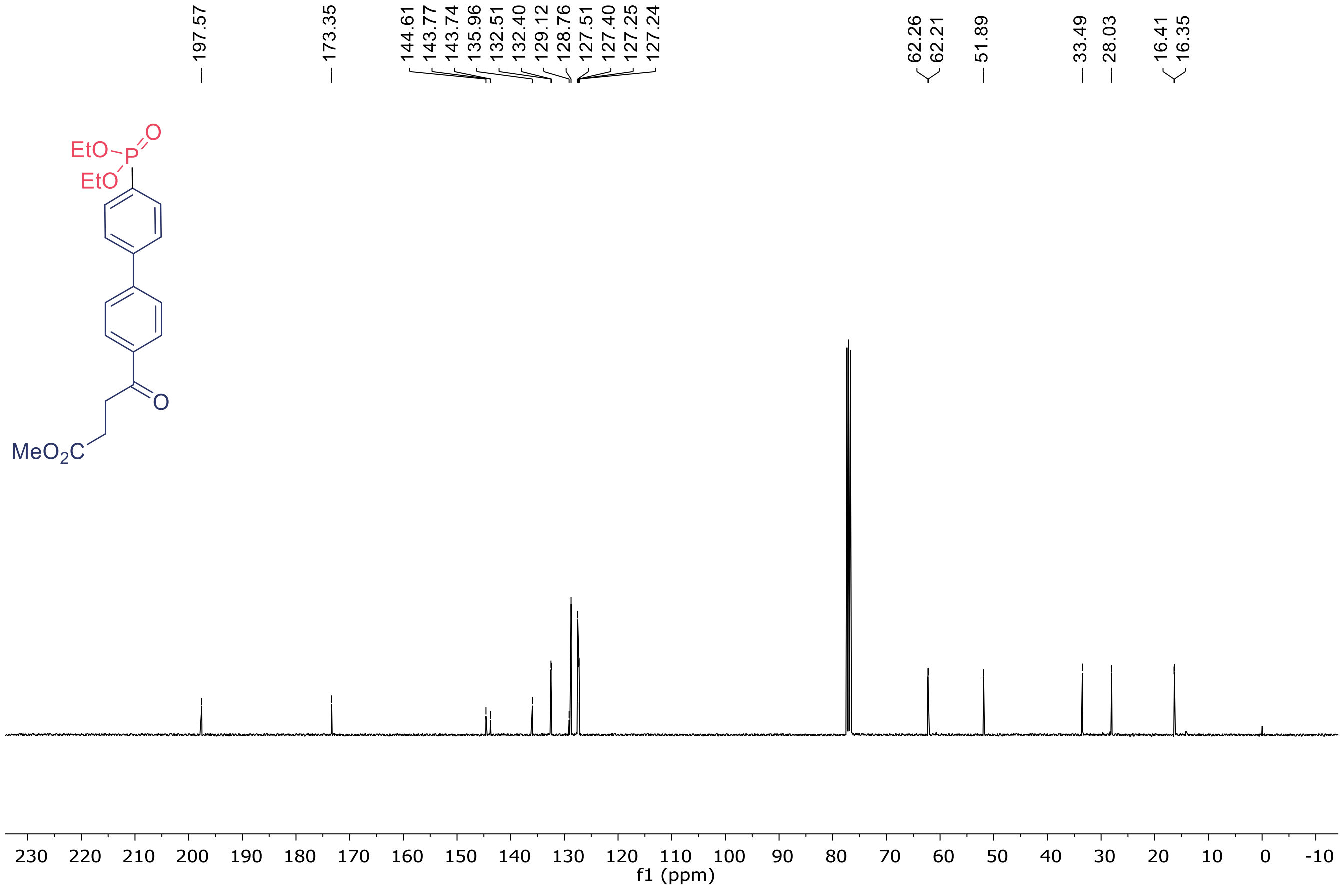

^{13}C NMR (101 MHz, $CDCl_3$) of compound **13**.

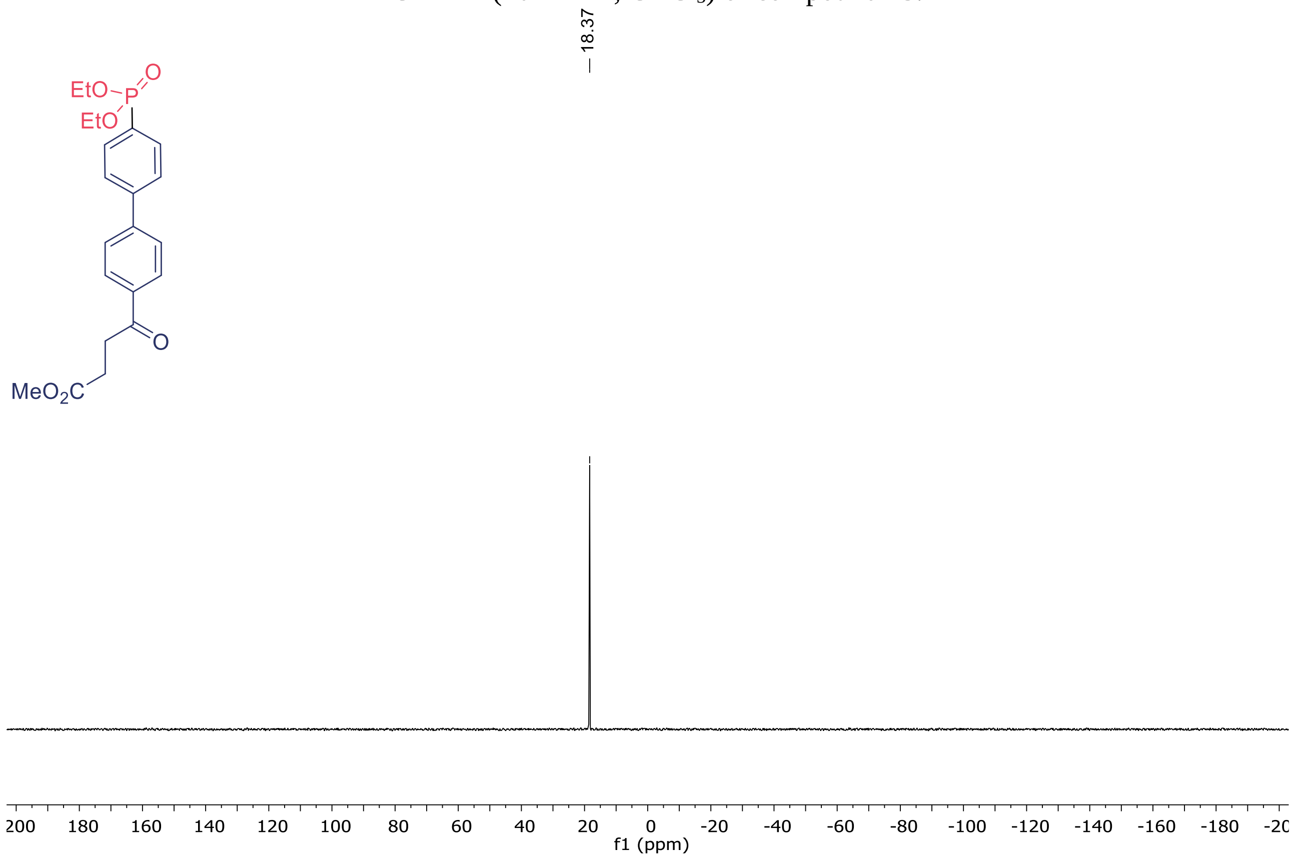

^{31}P NMR (162 MHz, $CDCl_3$) of compound **13**.

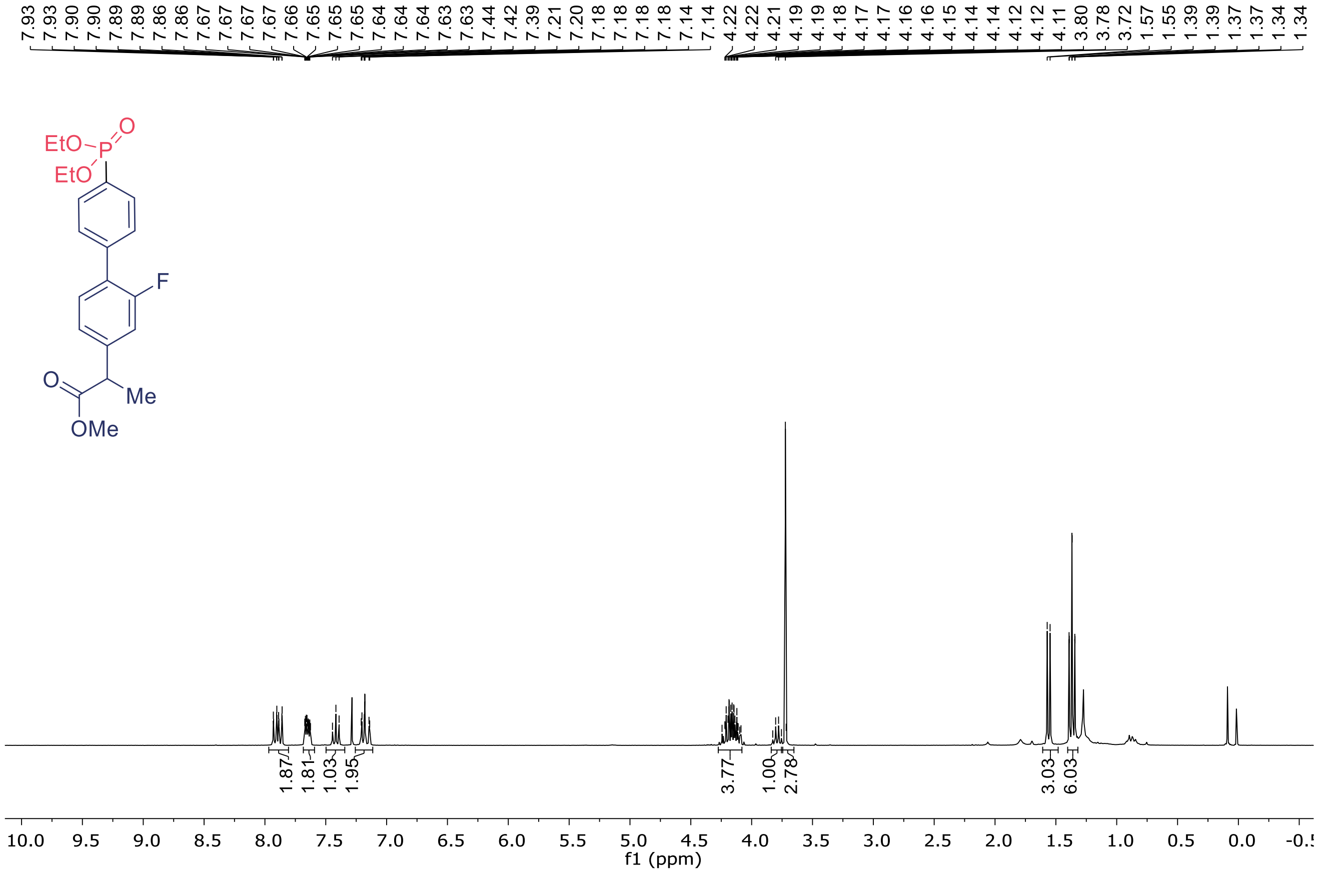

^{1}H NMR (300 MHz, $CDCl_3$) of compound **14**.

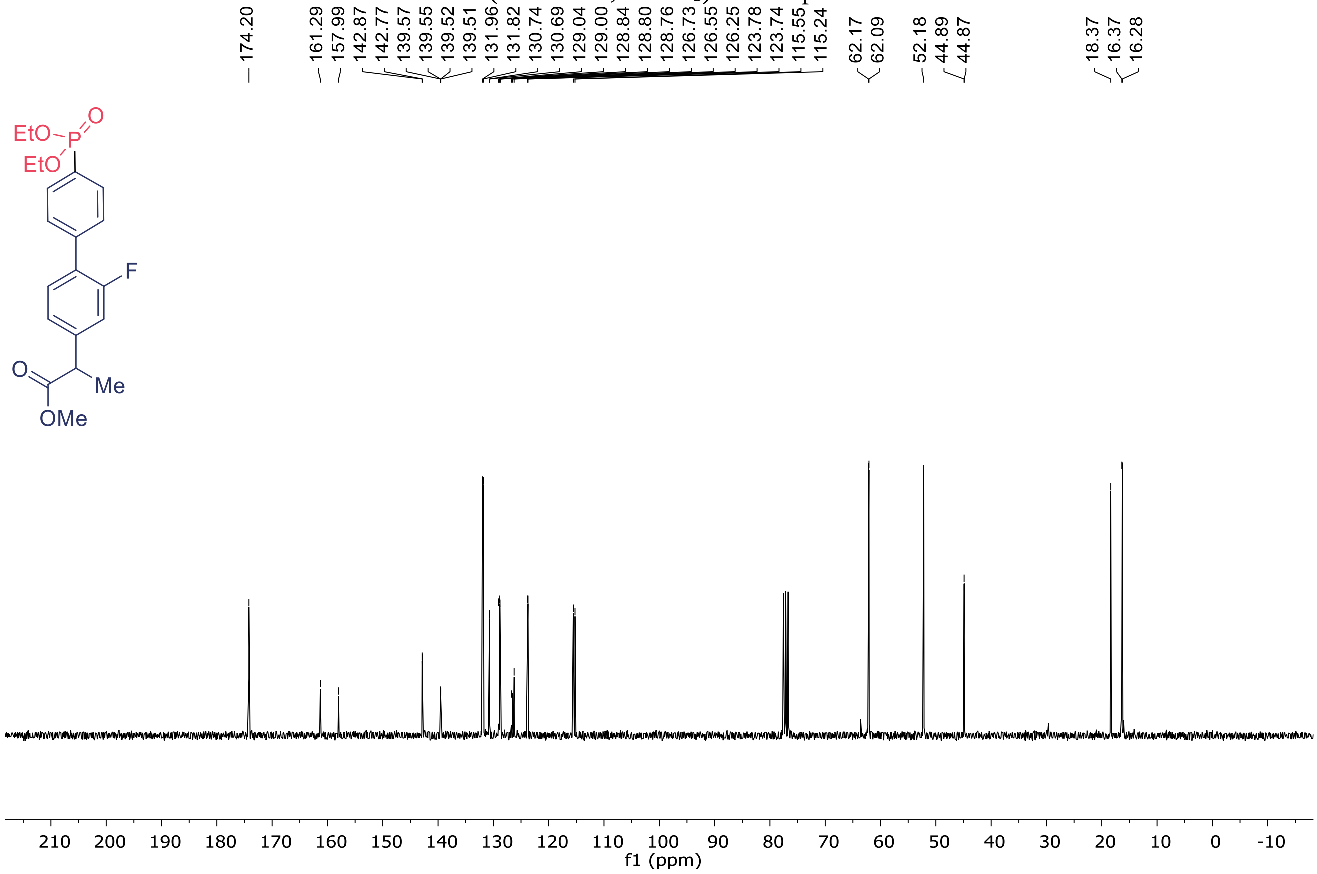

^{13}C NMR (75 MHz, $CDCl_3$) of compound **14**.

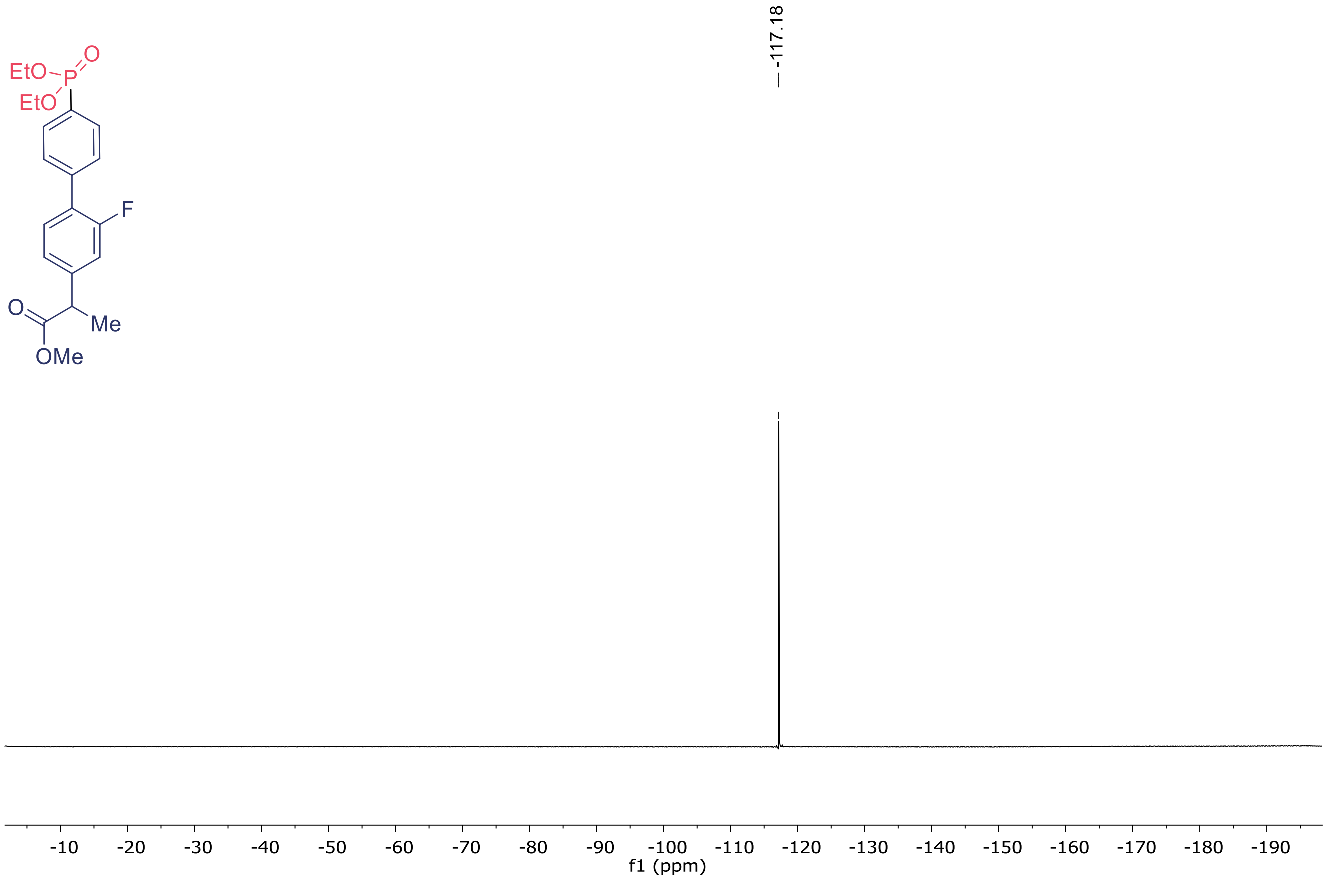

^{19}F NMR (282 MHz, $CDCl_3$) of compound **14**.

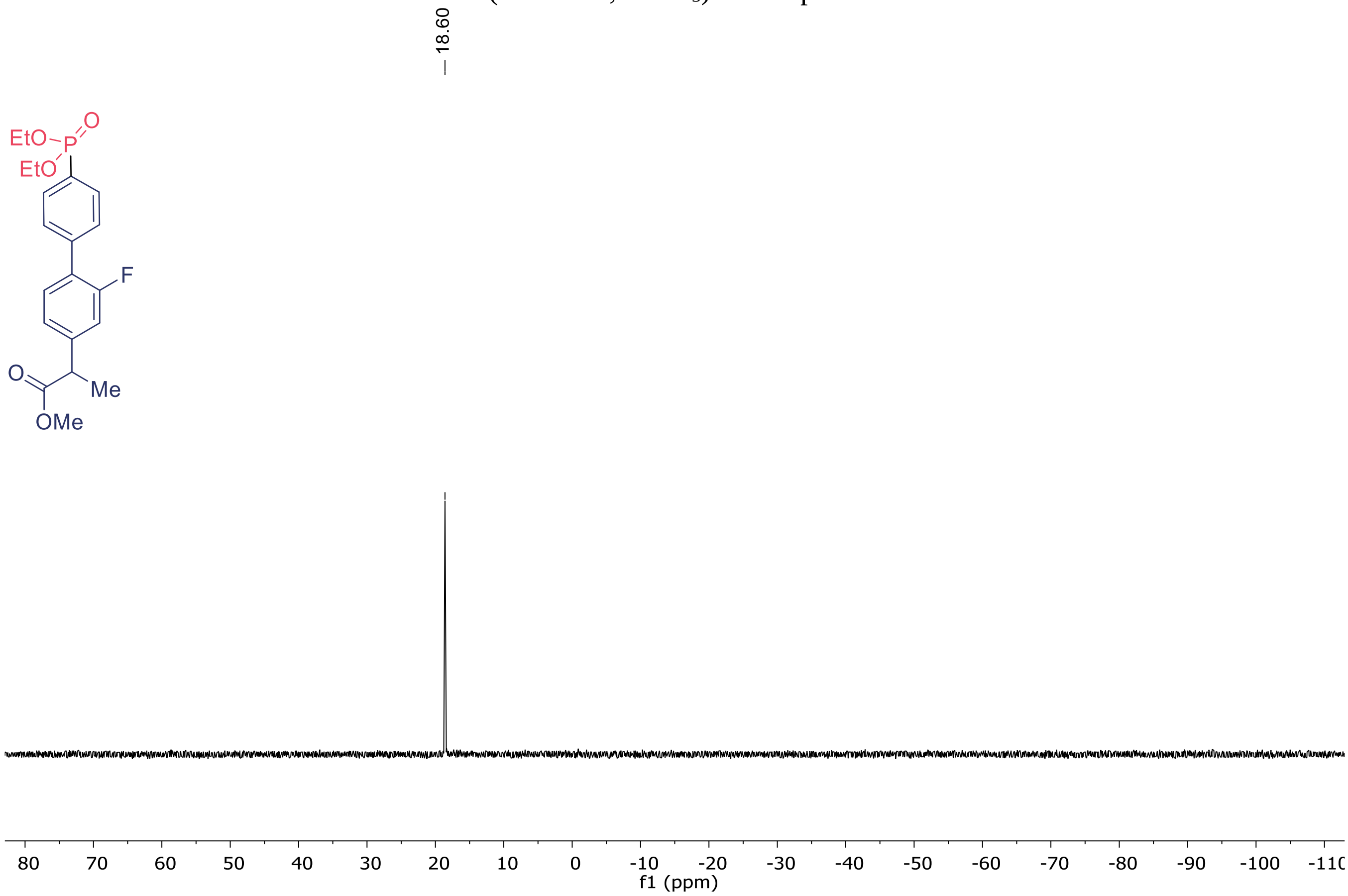

^{31}P NMR (122 MHz, $CDCl_3$) of compound **14**.

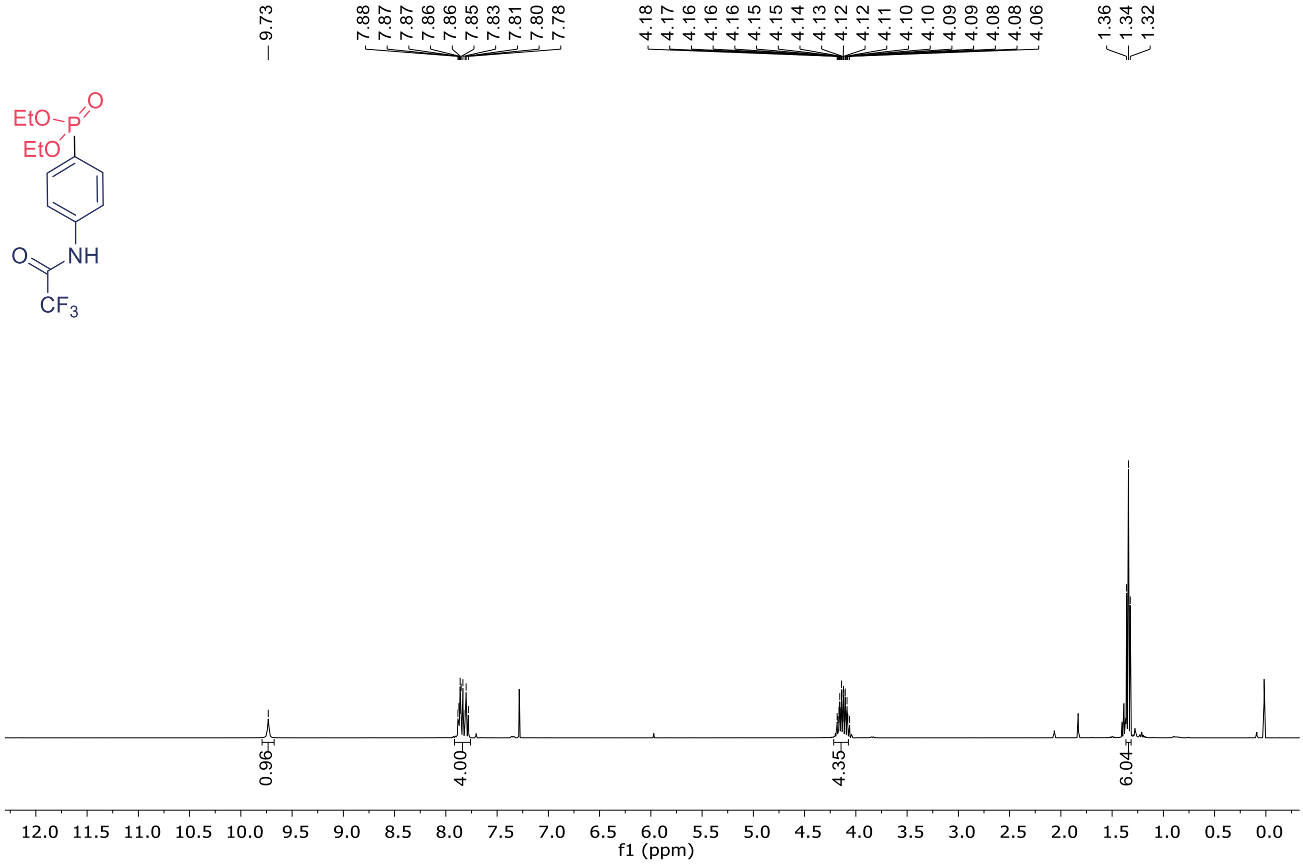

^{1}H NMR (400 MHz, $CDCl_3$) of compound **15**.

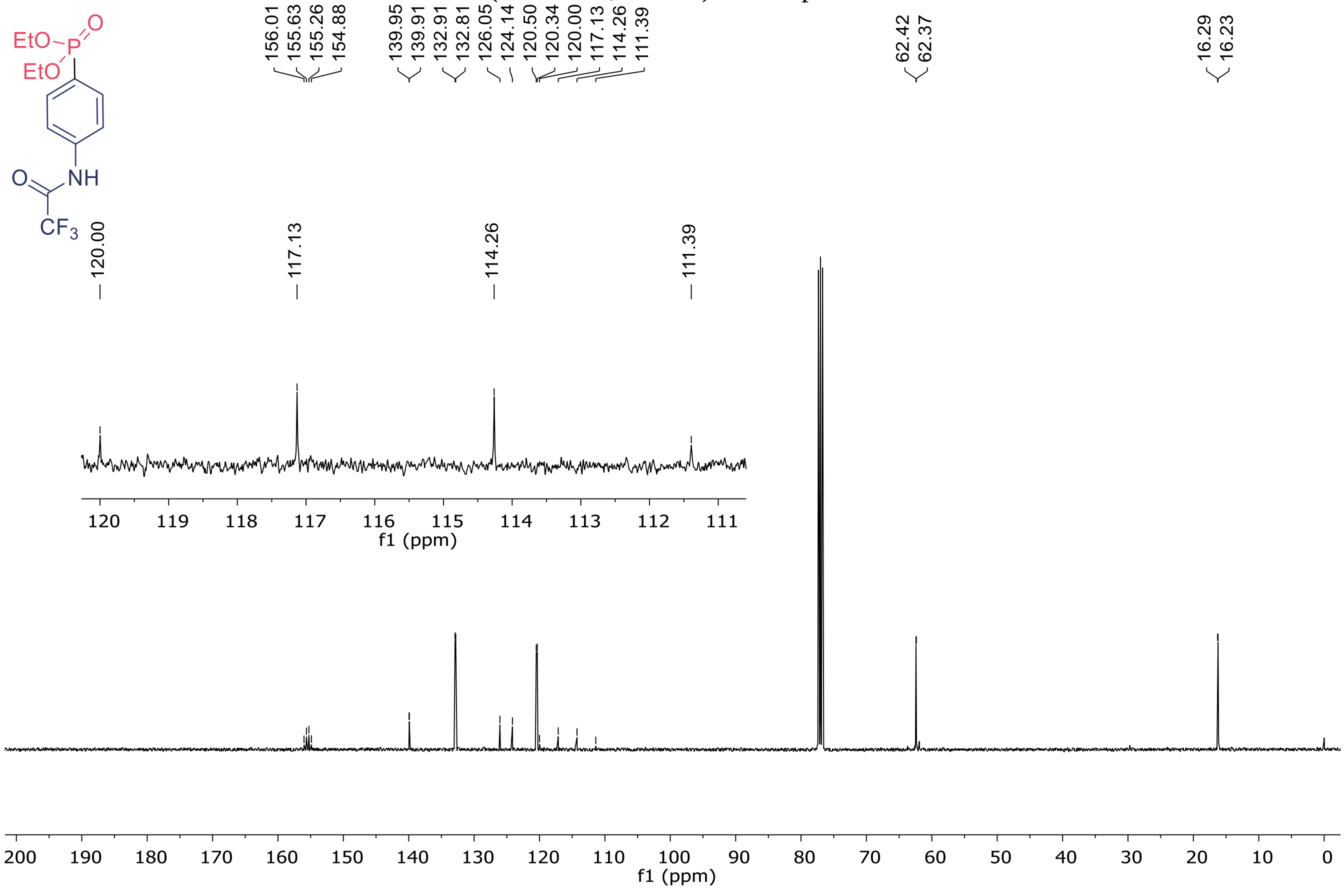

^{13}C NMR (101 MHz, $CDCl_3$) of compound **15**.

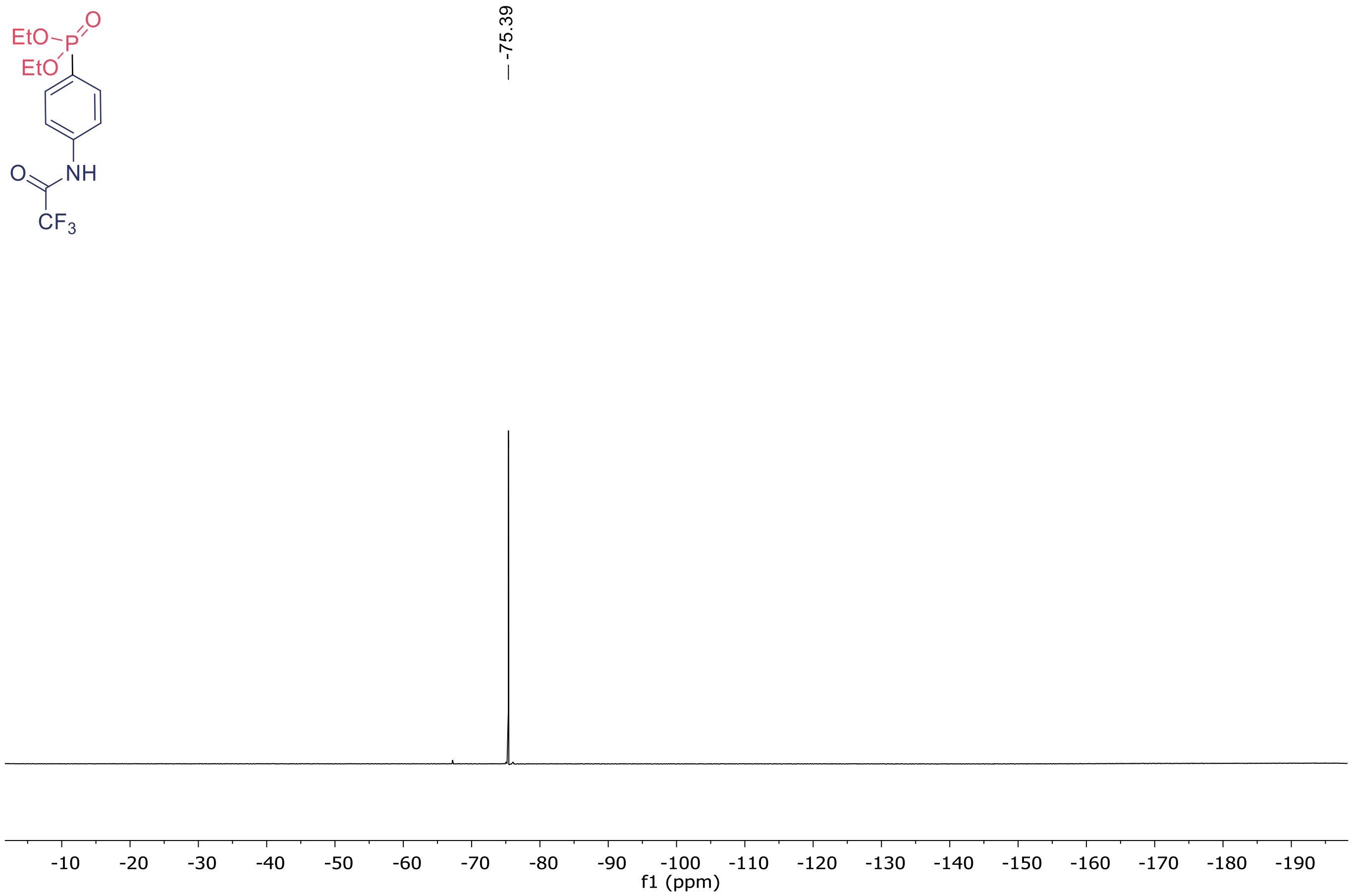

^{19}F NMR (376 MHz, $CDCl_3$) of compound **15**.

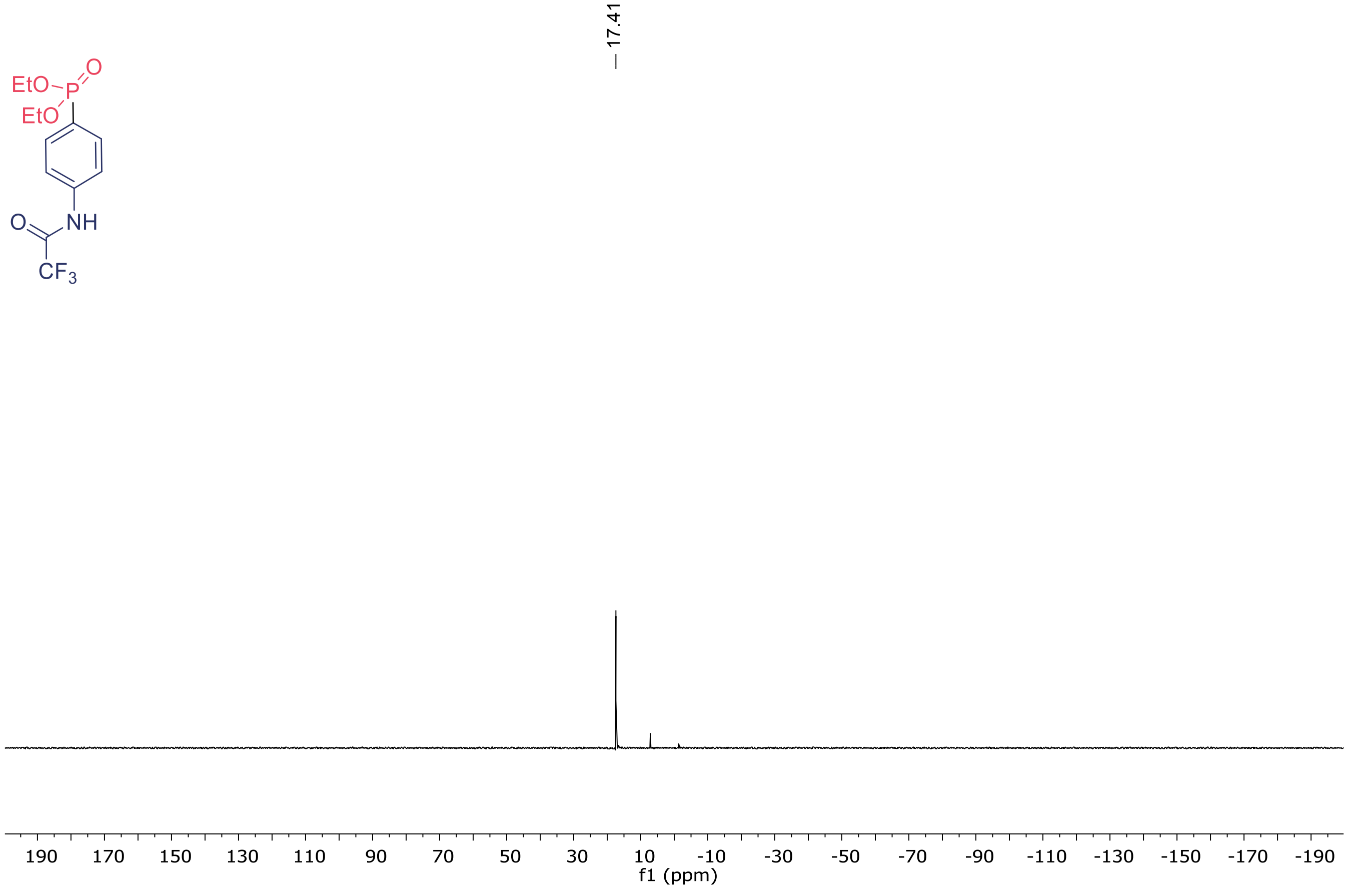

^{31}P NMR (162 MHz, $CDCl_3$) of compound **15**.

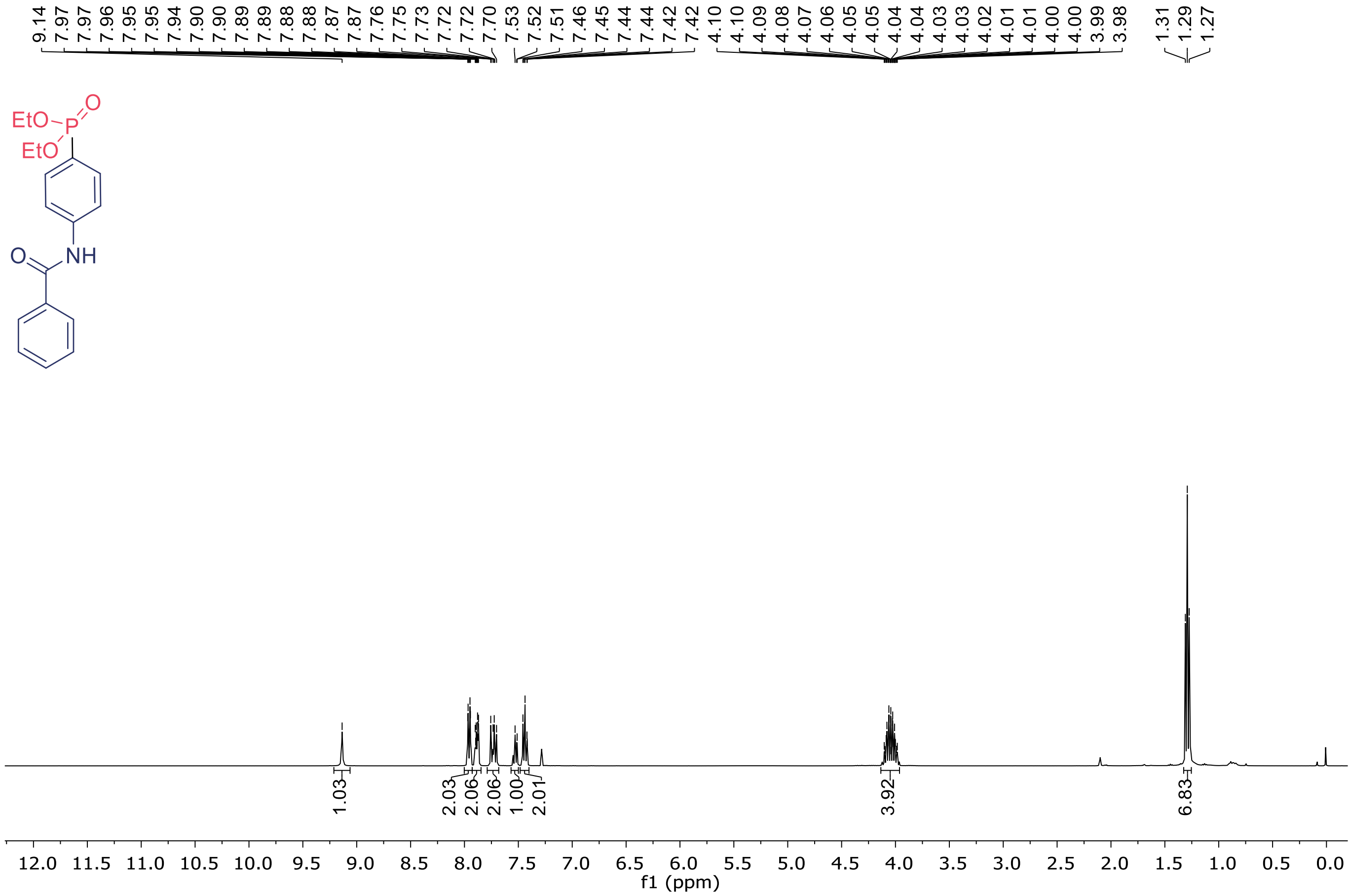

^{1}H NMR (400 MHz, $CDCl_3$) of compound **16**.

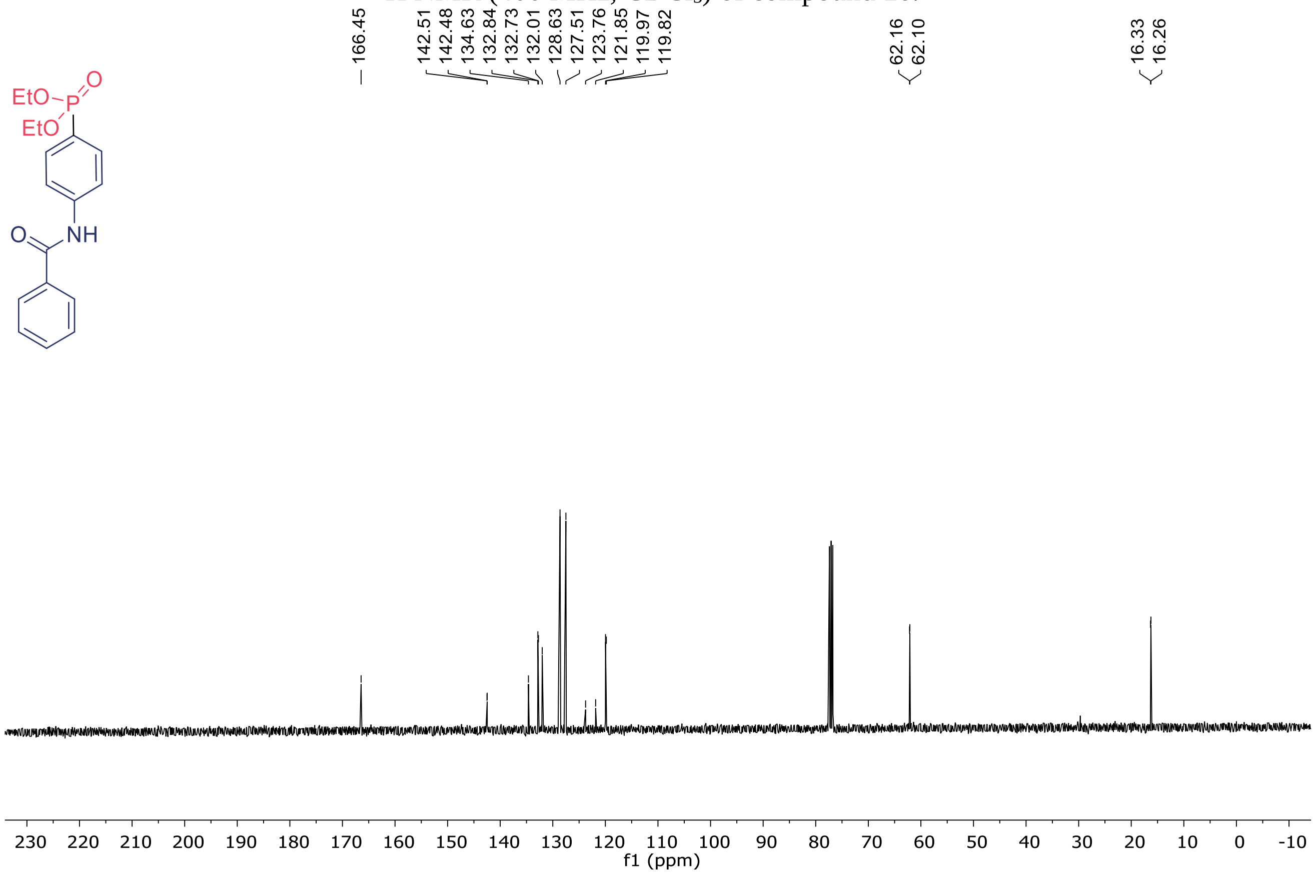

^{13}C NMR (101 MHz, $CDCl_3$) of compound **16**.

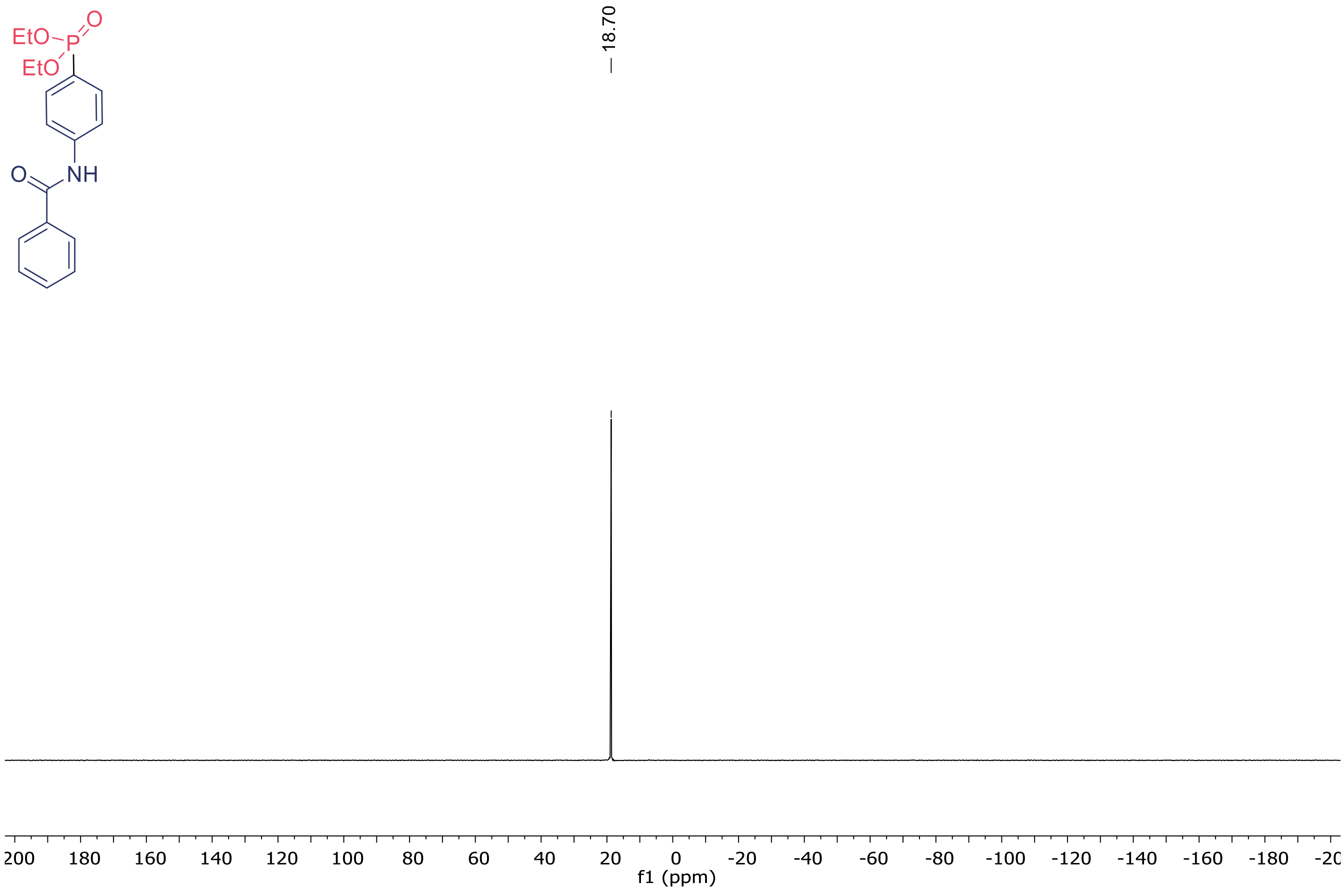

^{31}P NMR (162 MHz, $CDCl_3$) of compound **16**.

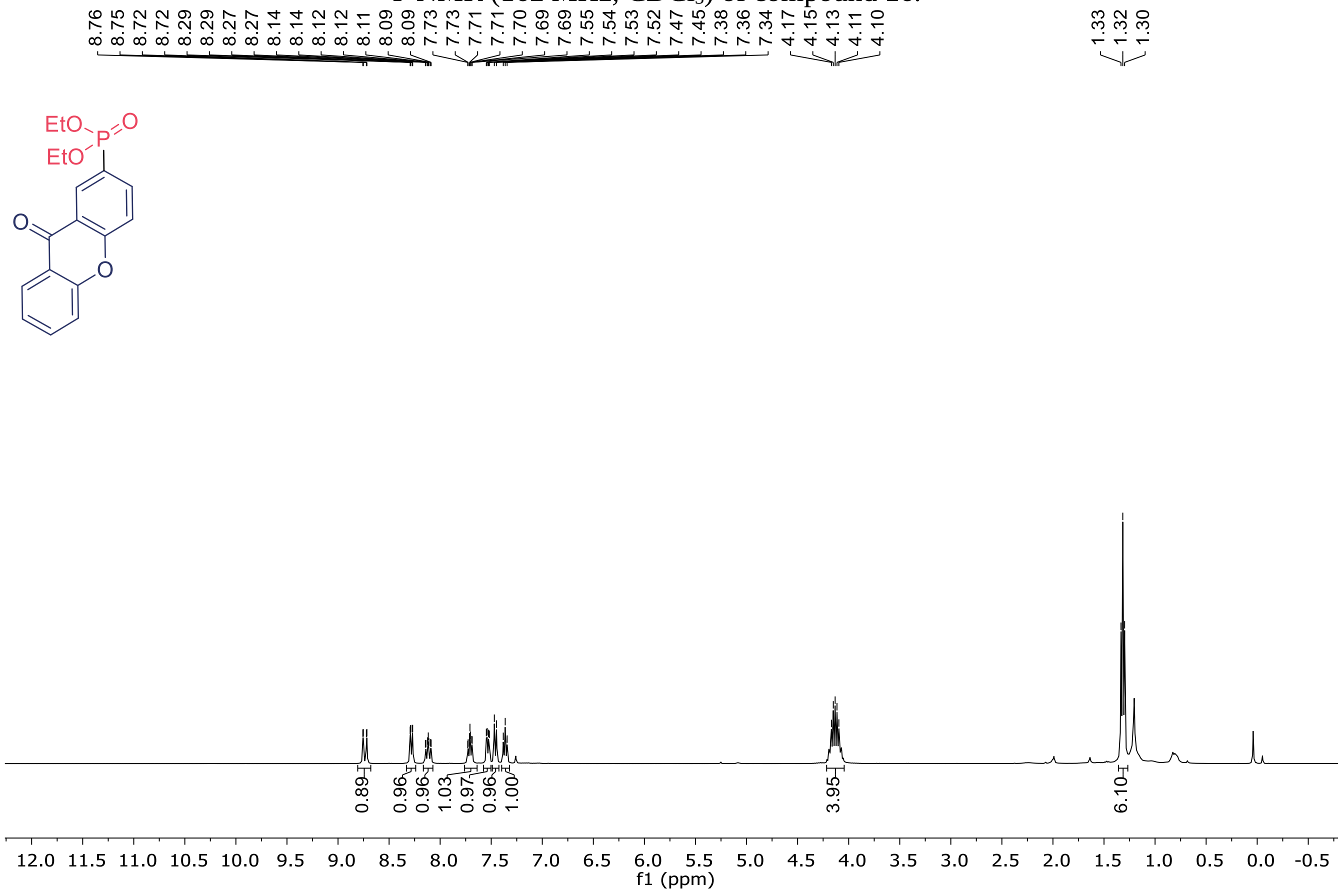

^{1}H NMR (400 MHz, $CDCl_3$) of compound **17**.

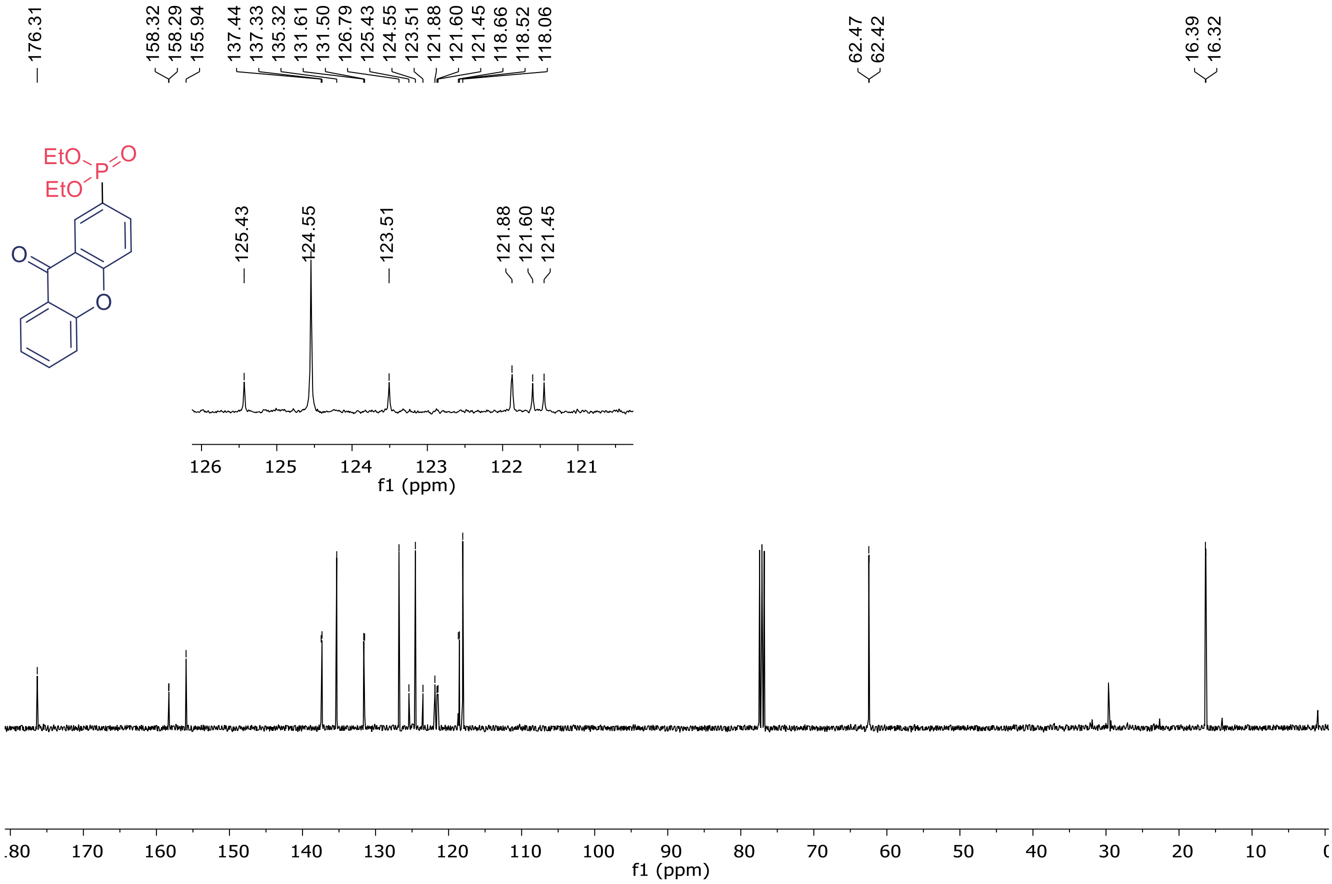

^{13}C NMR (101 MHz, $CDCl_3$) of compound **17**.

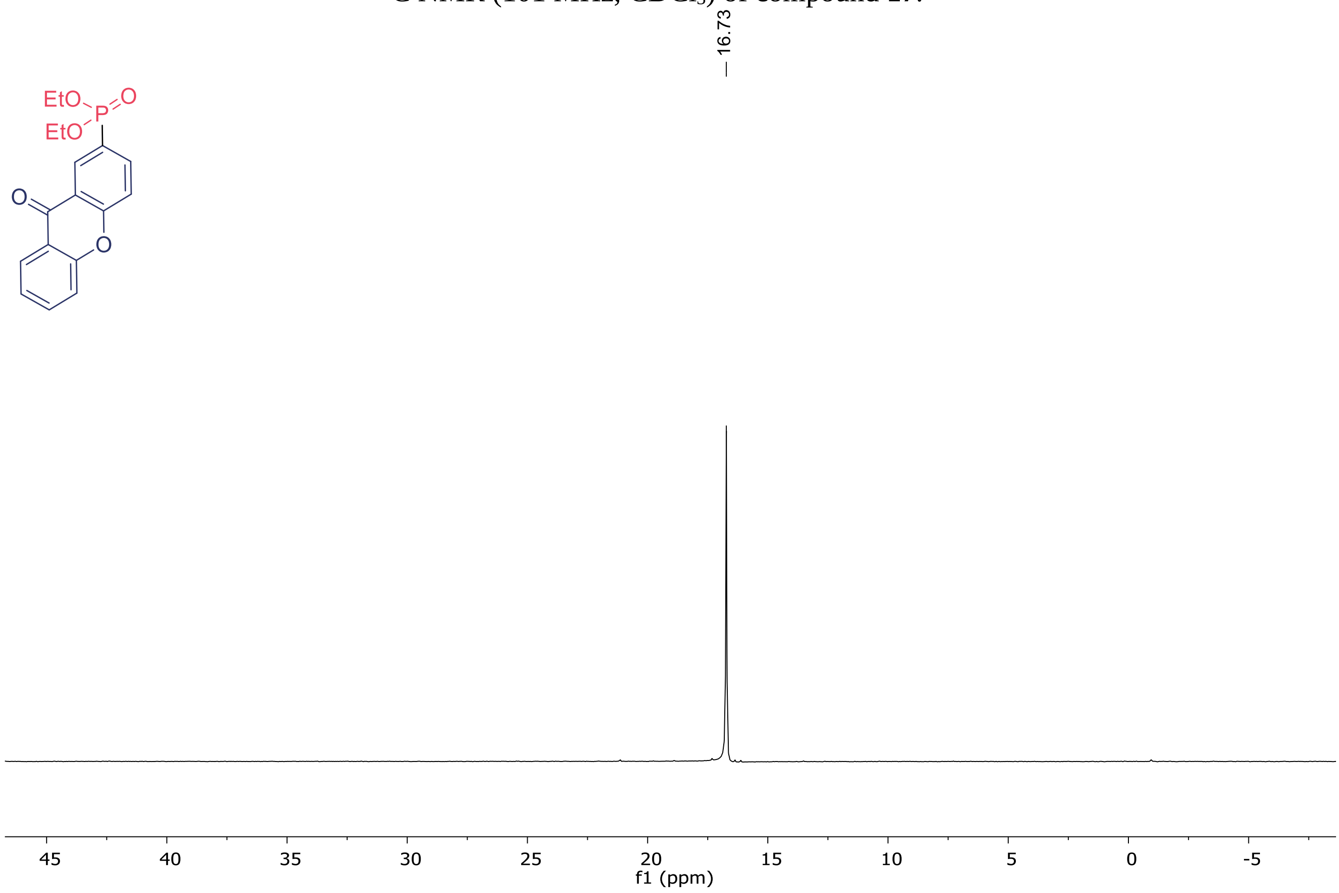

^{31}P NMR (162 MHz, $CDCl_3$) of compound **17**.

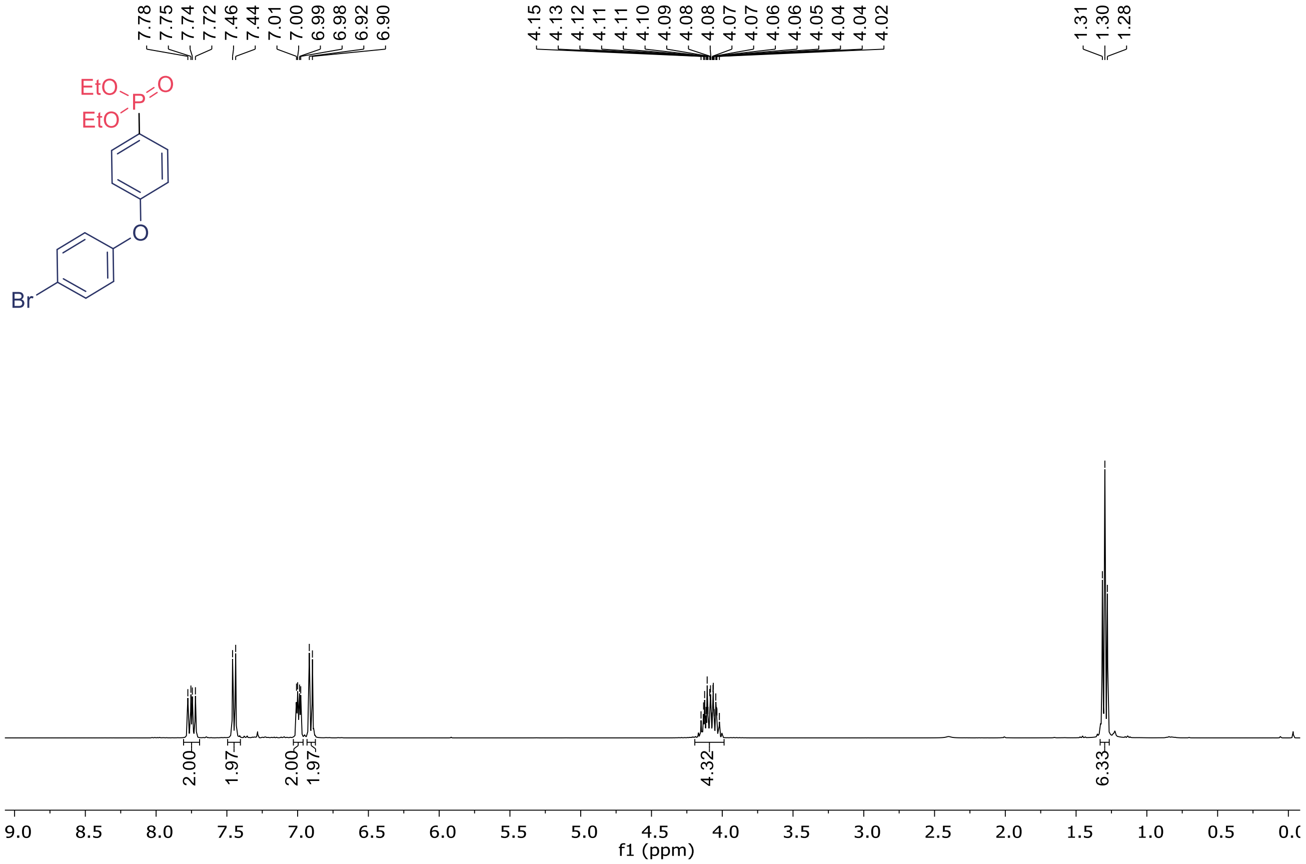

^{1}H NMR (400 MHz, $CDCl_3$) of compound **18**.

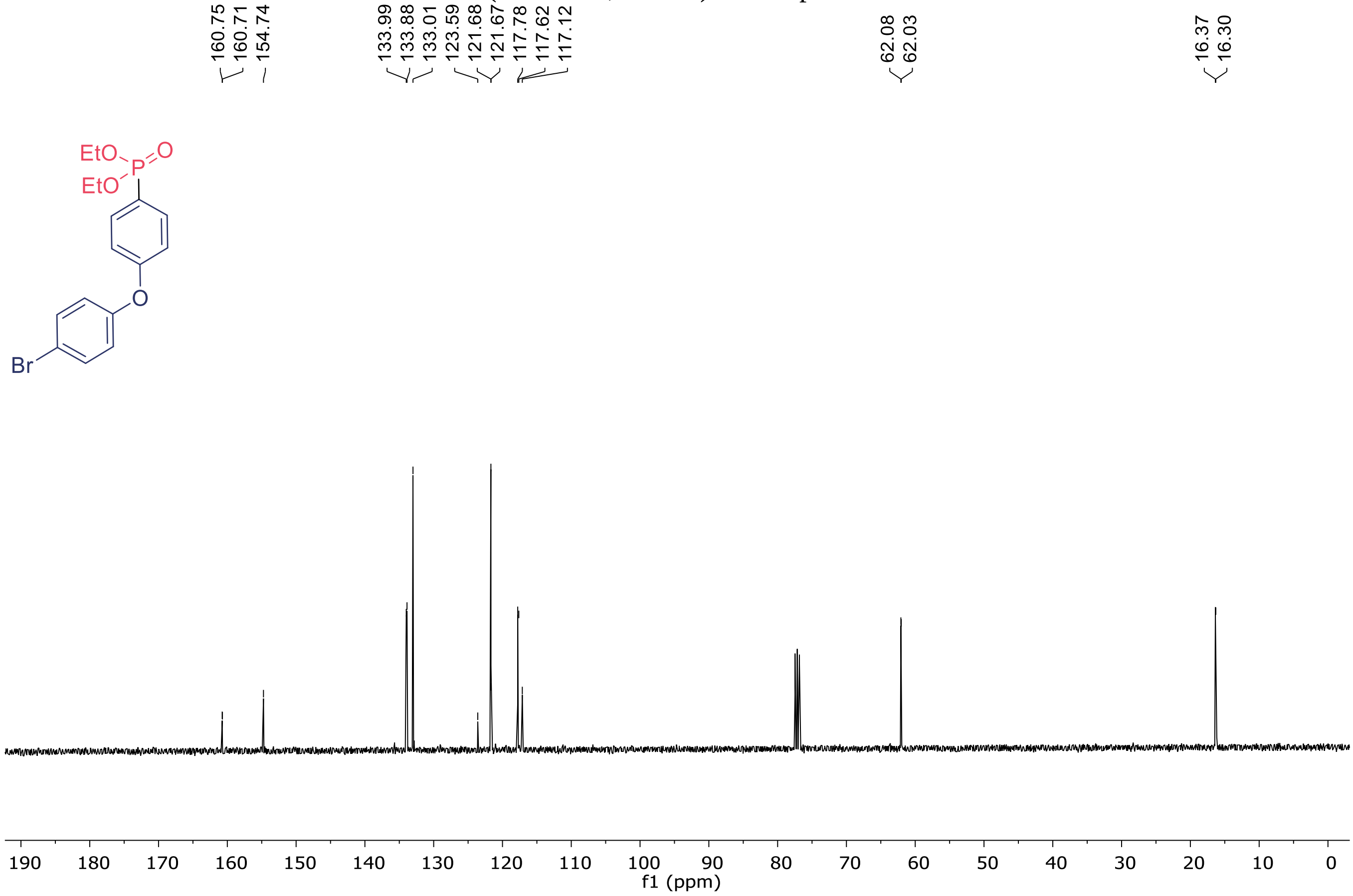

^{13}C NMR (101 MHz, $CDCl_3$) of compound **18**.

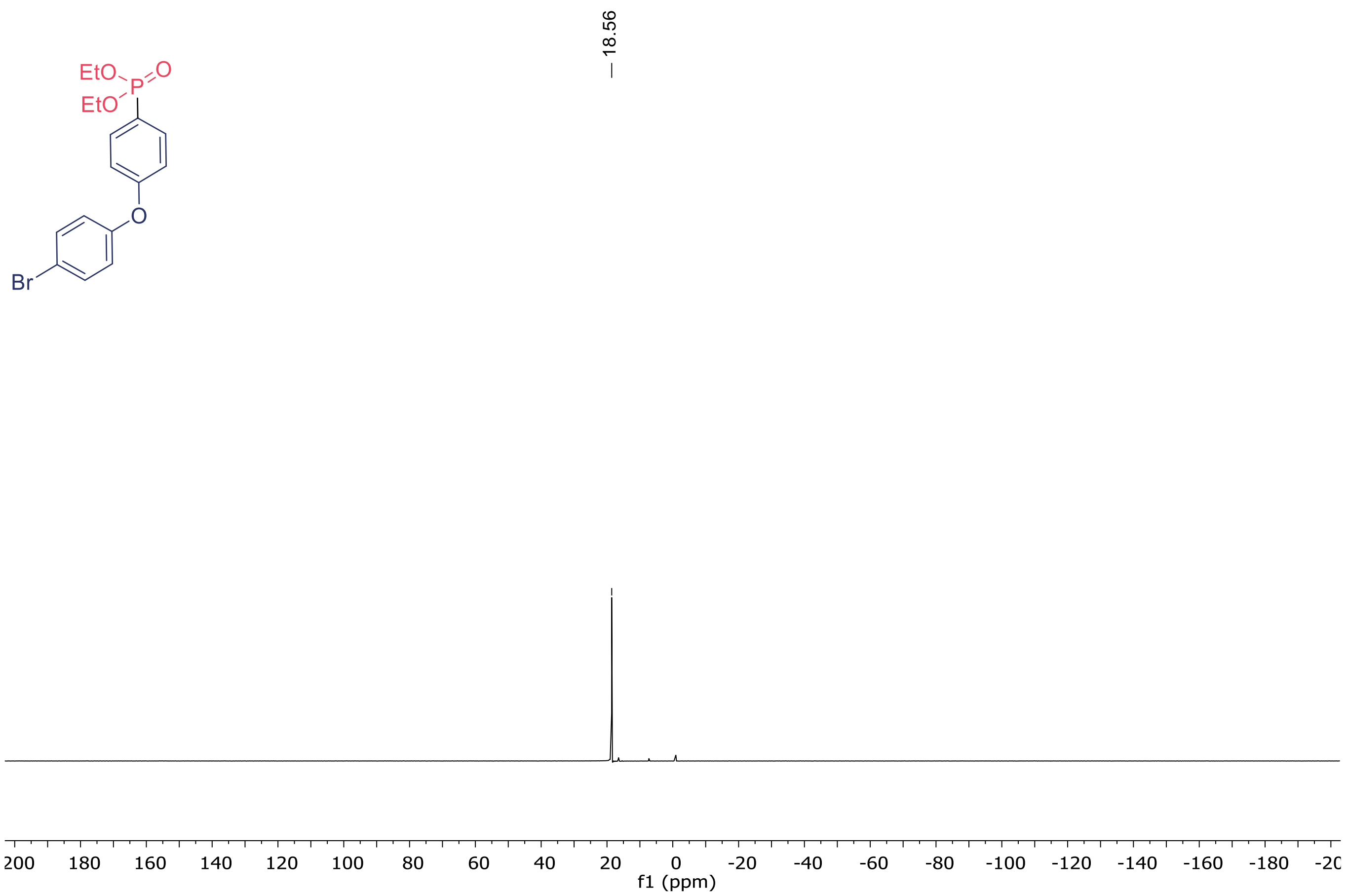

^{31}P NMR (162 MHz, $CDCl_3$) of compound **18**.

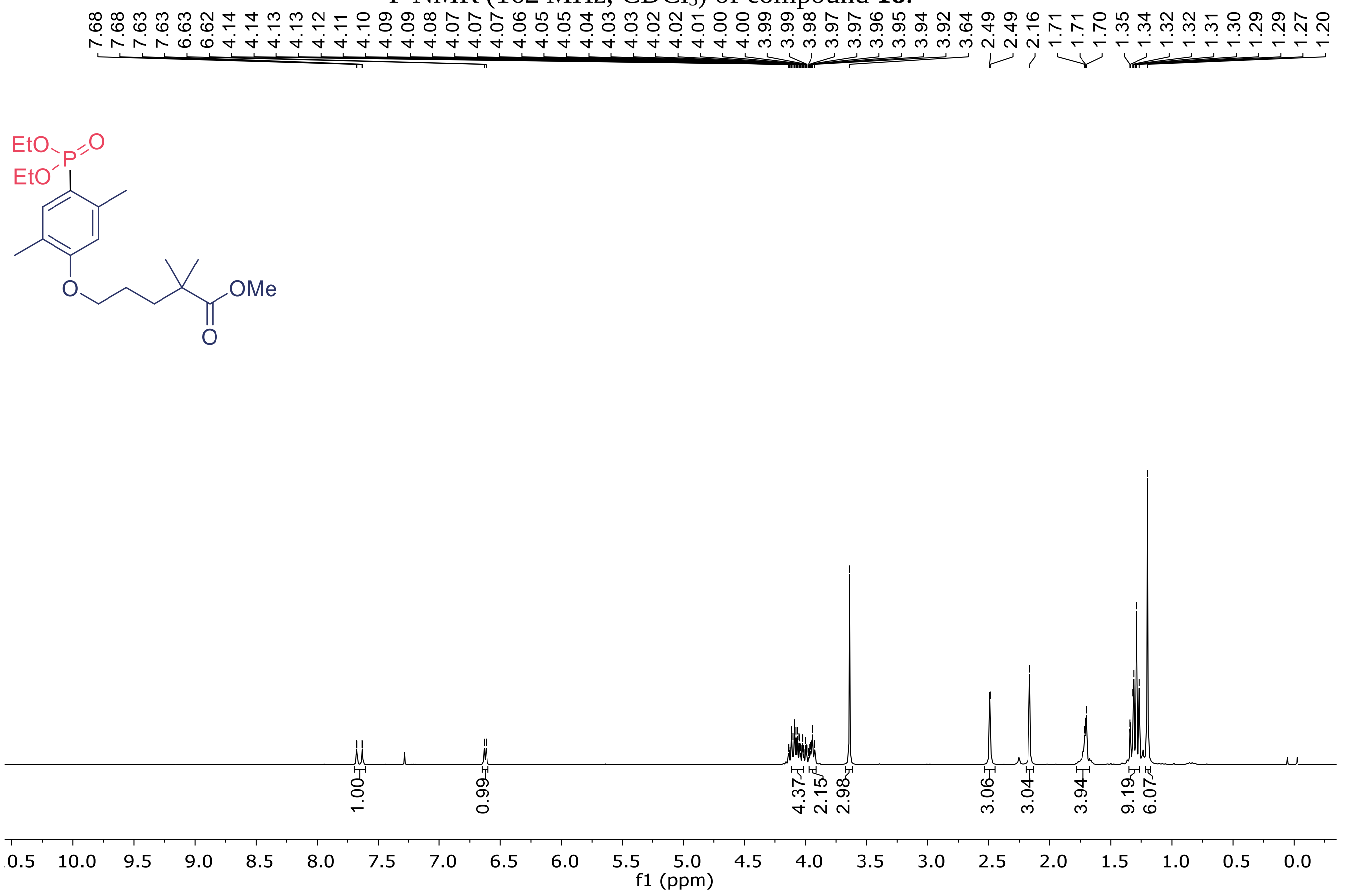

^1H NMR (300 MHz, $CDCl_3$) of compound **19**.

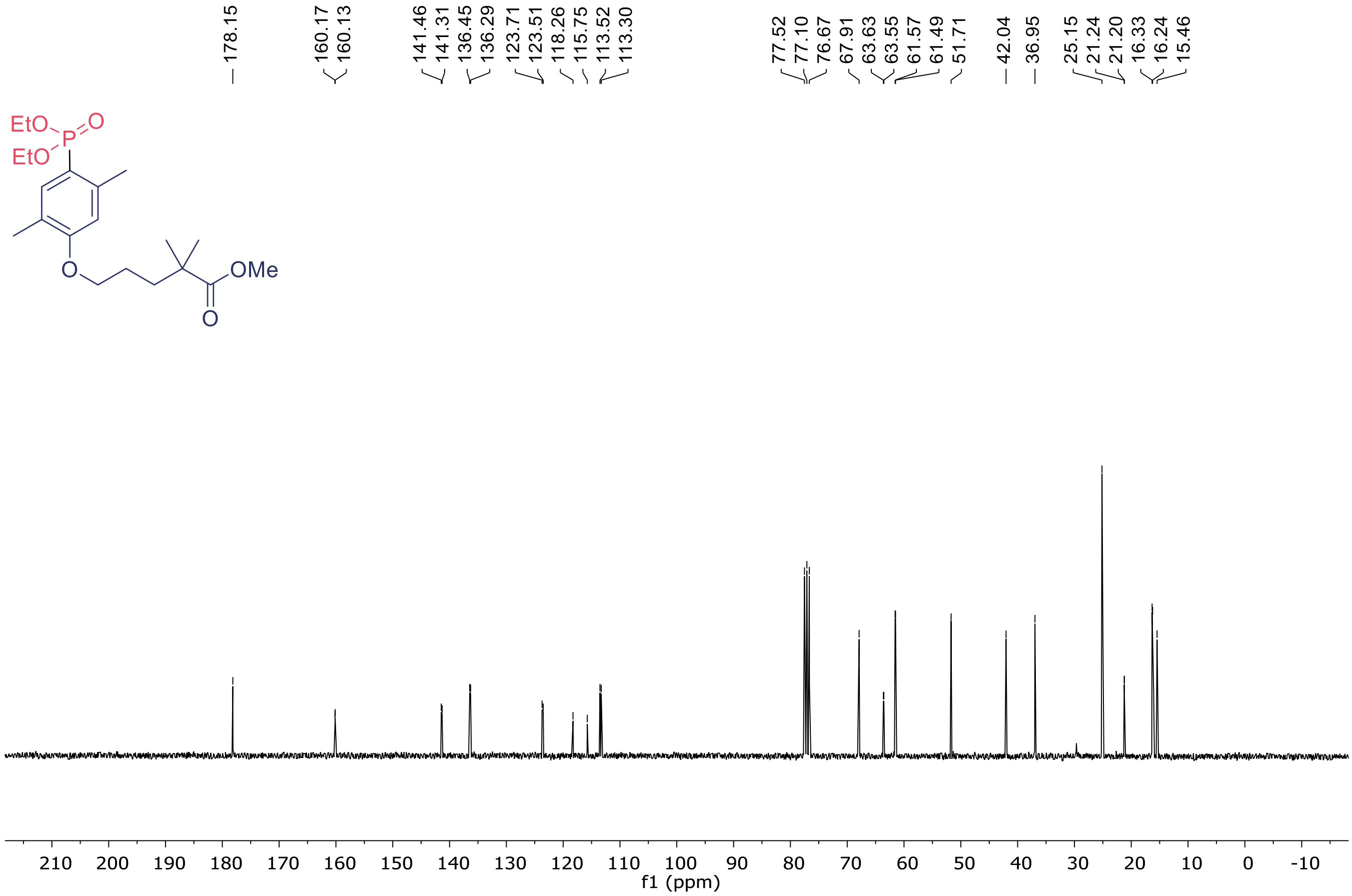

^{13}C NMR (75 MHz, $CDCl_3$) of compound **19**.

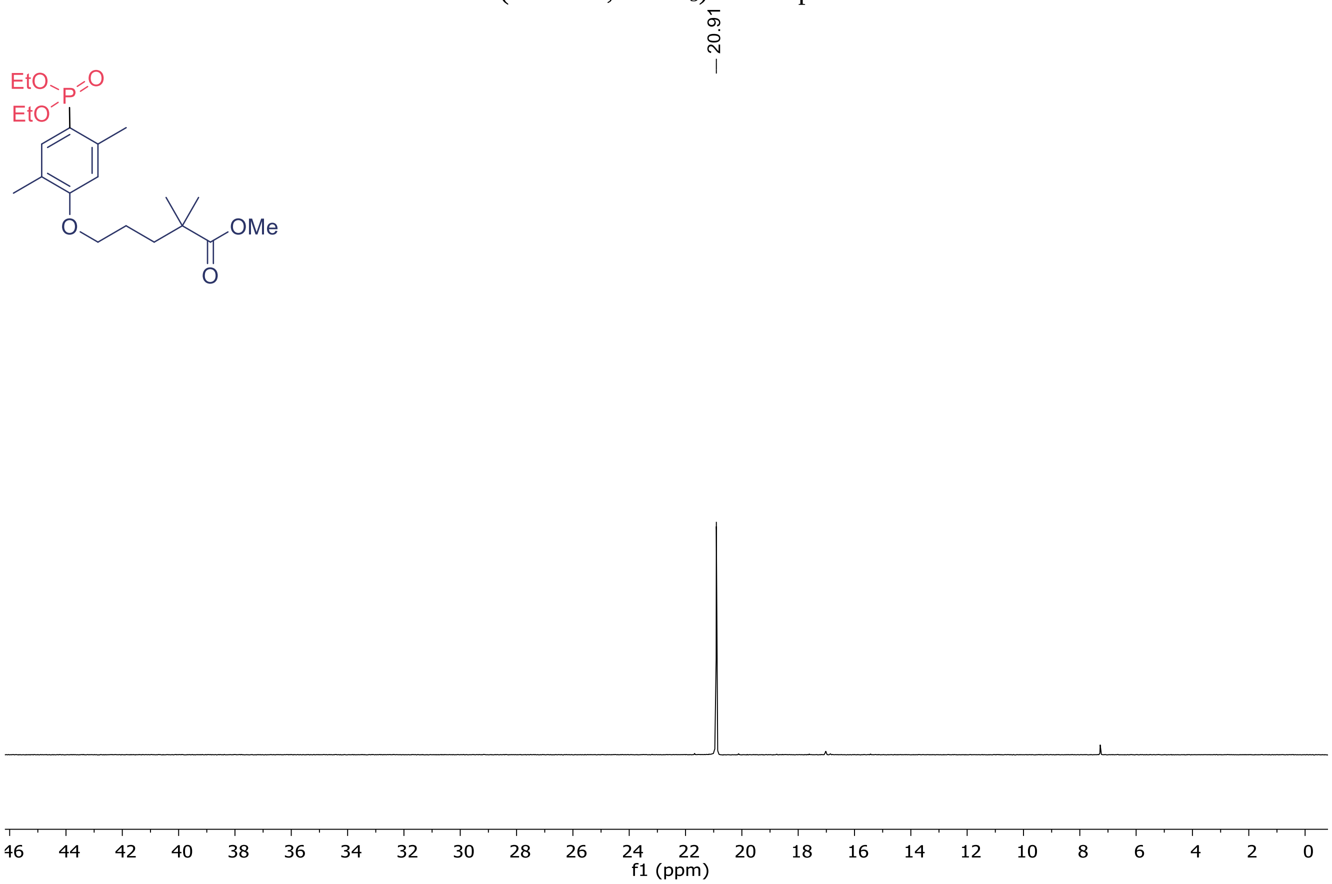

^{31}P NMR (122 MHz, $CDCl_3$) of compound **19**.

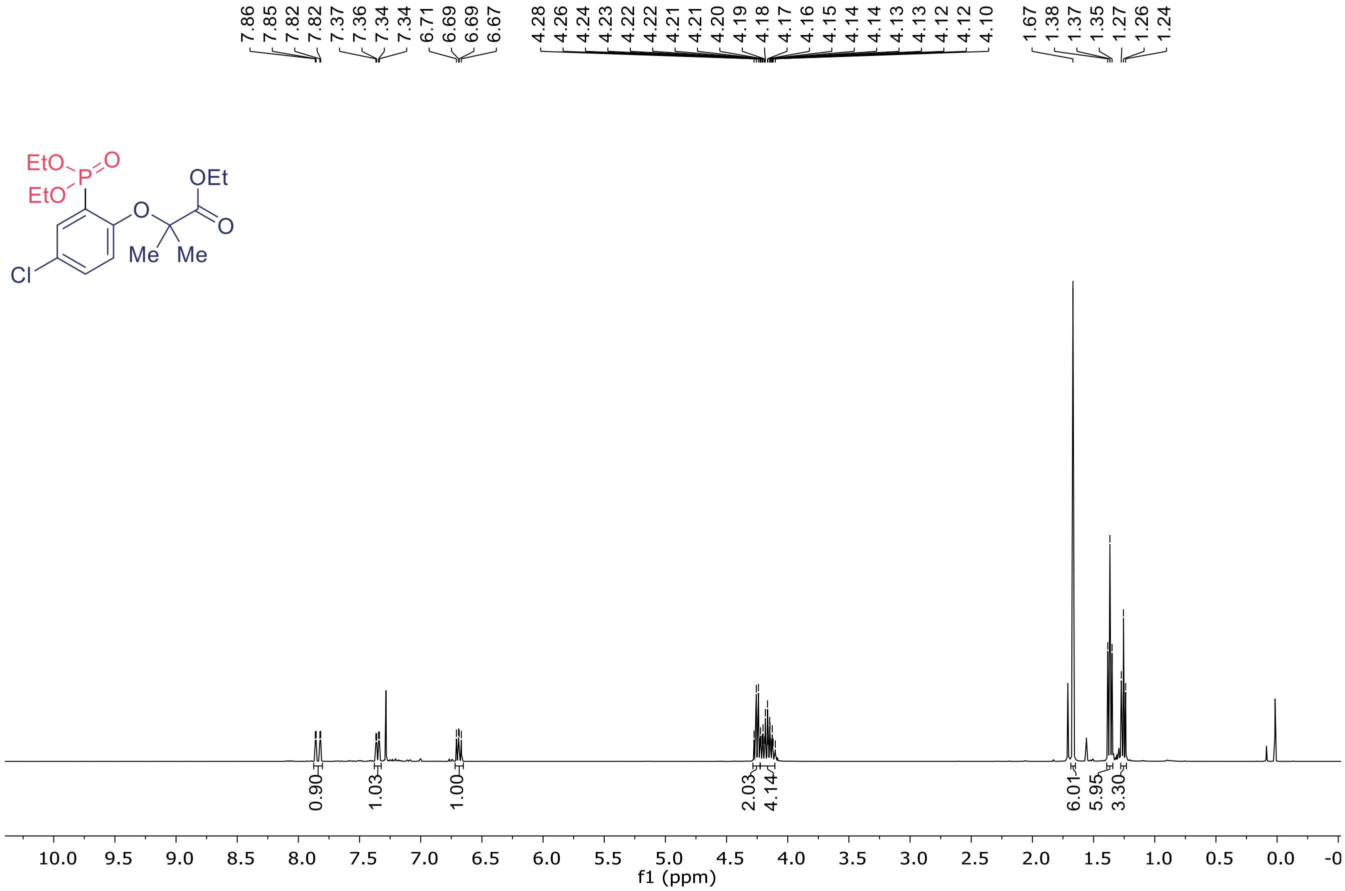

^{1}H NMR (400 MHz, $CDCl_3$) of compound **20**.

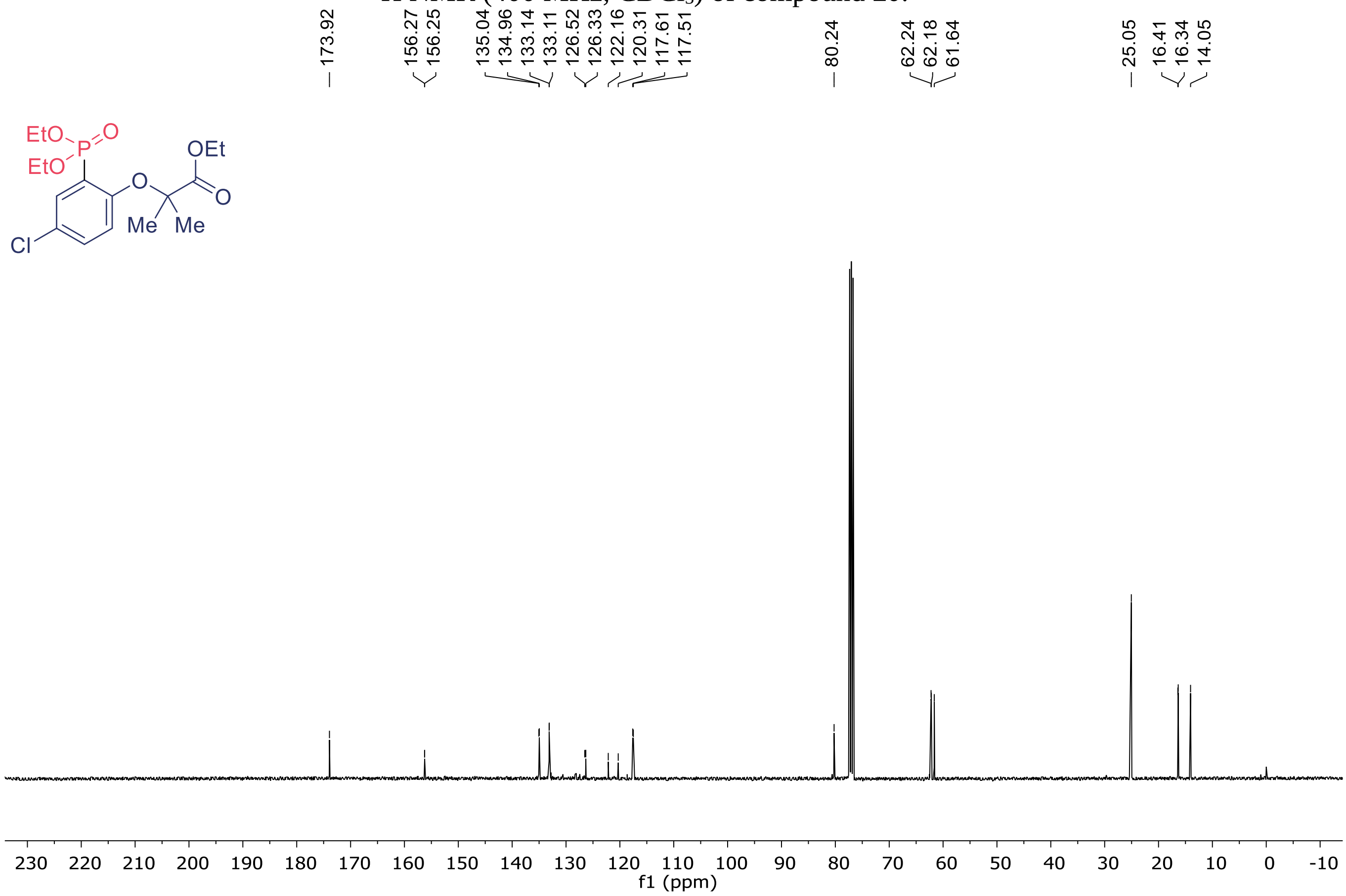

^{13}C NMR (101 MHz, $CDCl_3$) of compound **20**.

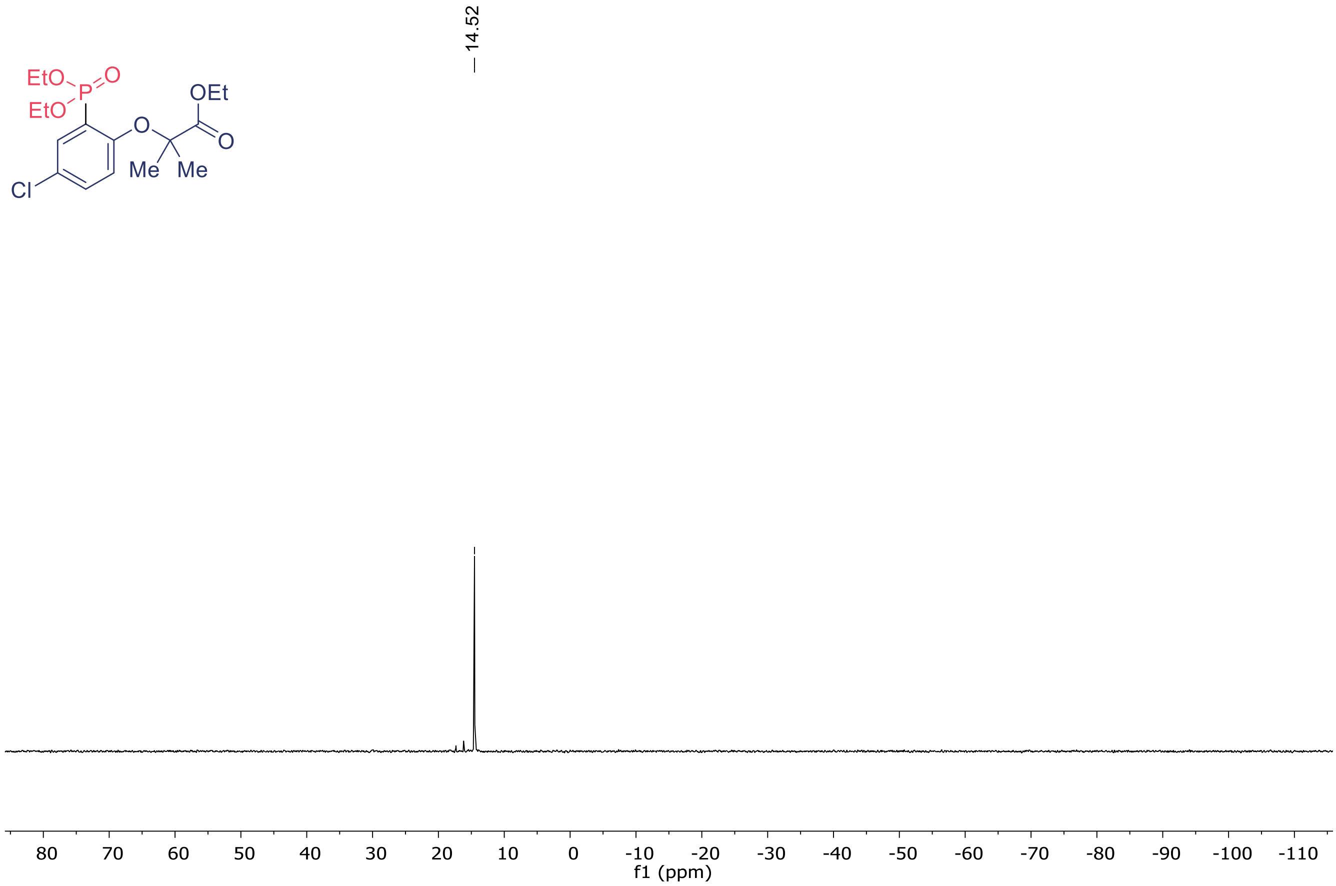

^{31}P NMR (162 MHz, CDCl$_3$) of compound **20**.

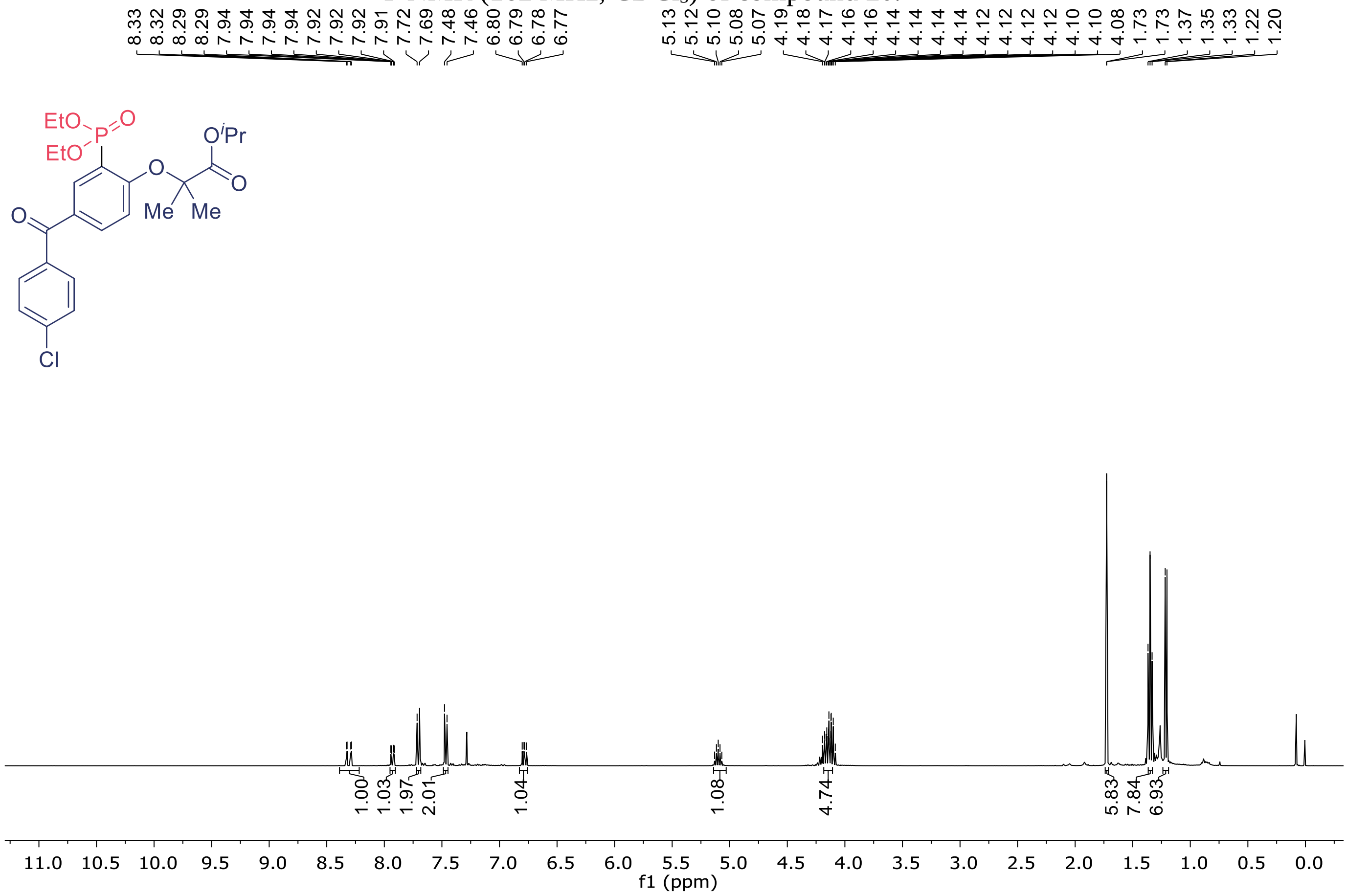

^{1}H NMR (400 MHz, CDCl$_3$) of compound **21**.

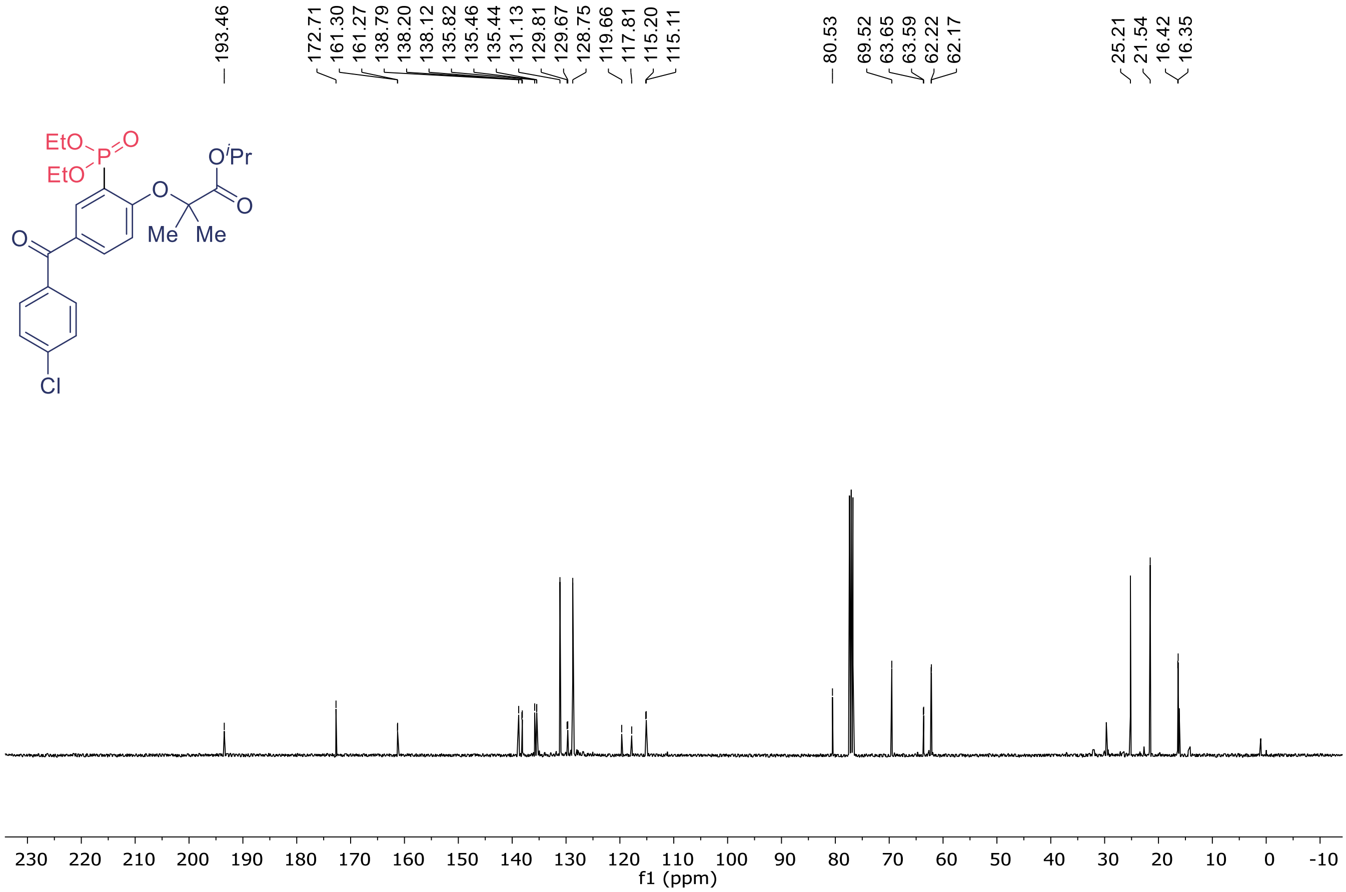

^{13}C NMR (101 MHz, $CDCl_3$) of compound **21**.

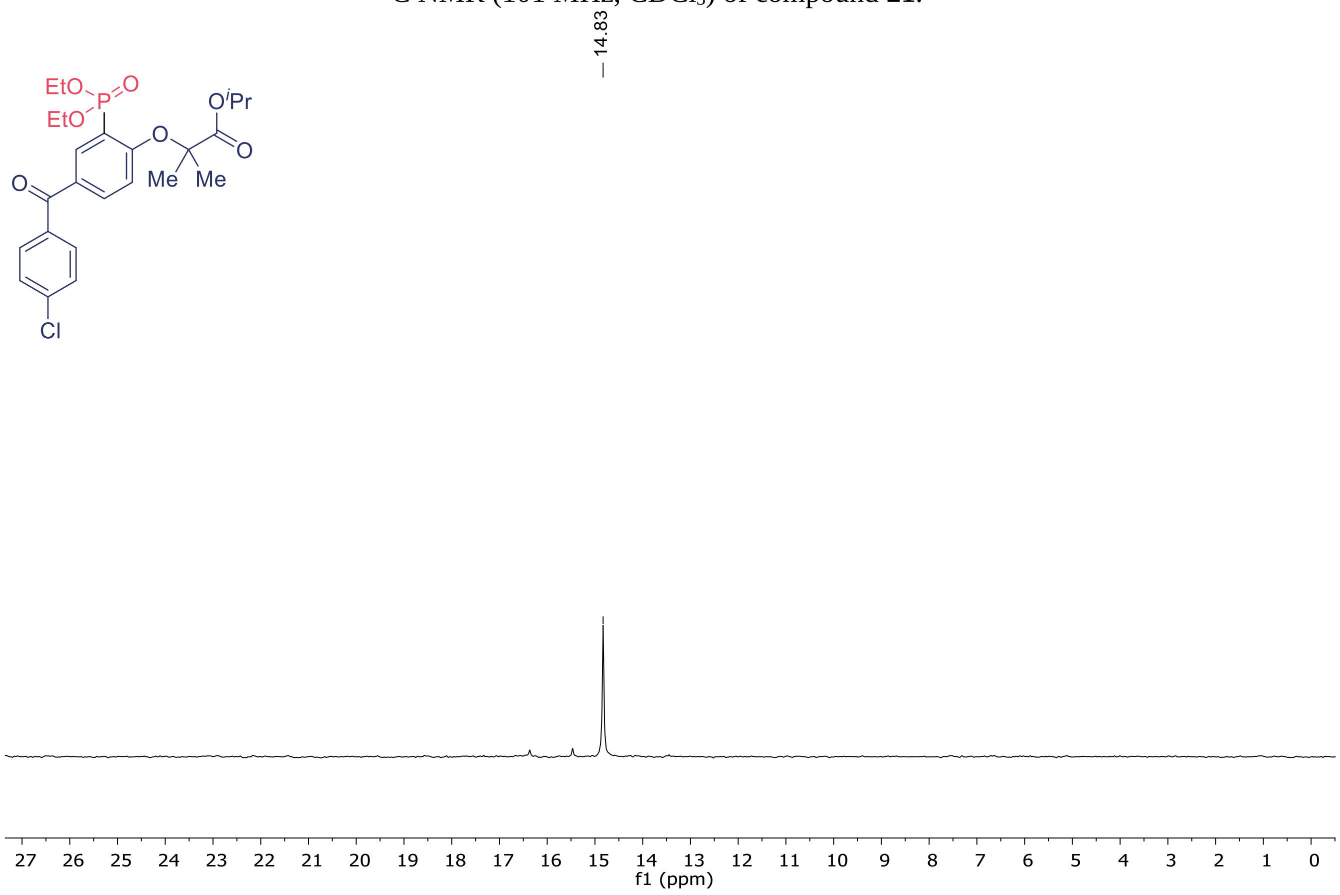

^{31}P NMR (162 MHz, $CDCl_3$) of compound **21**.

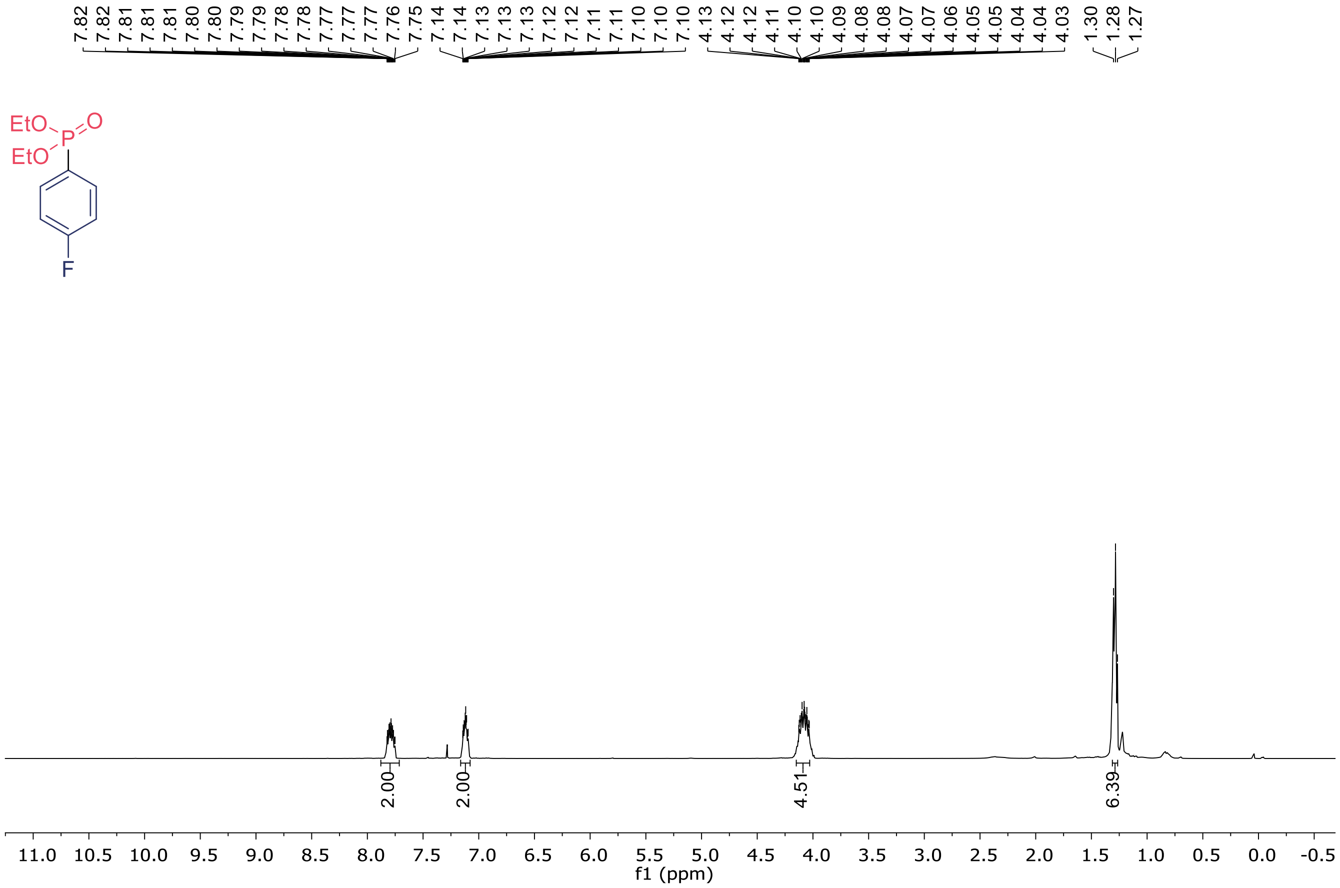

^{1}H NMR (400 MHz, $CDCl_3$) of compound **22**.

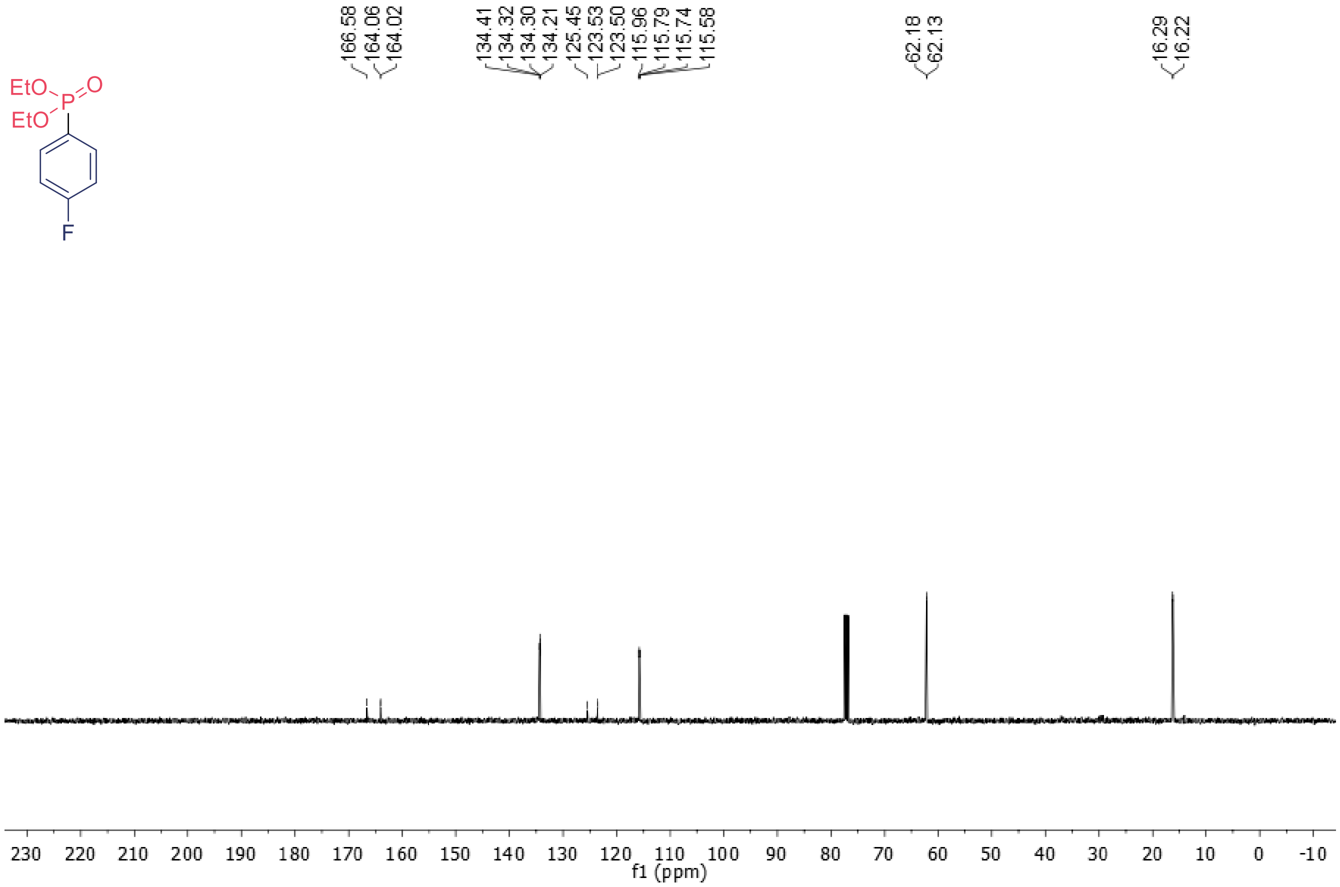

^{13}C NMR (101 MHz, $CDCl_3$) of compound **22**.

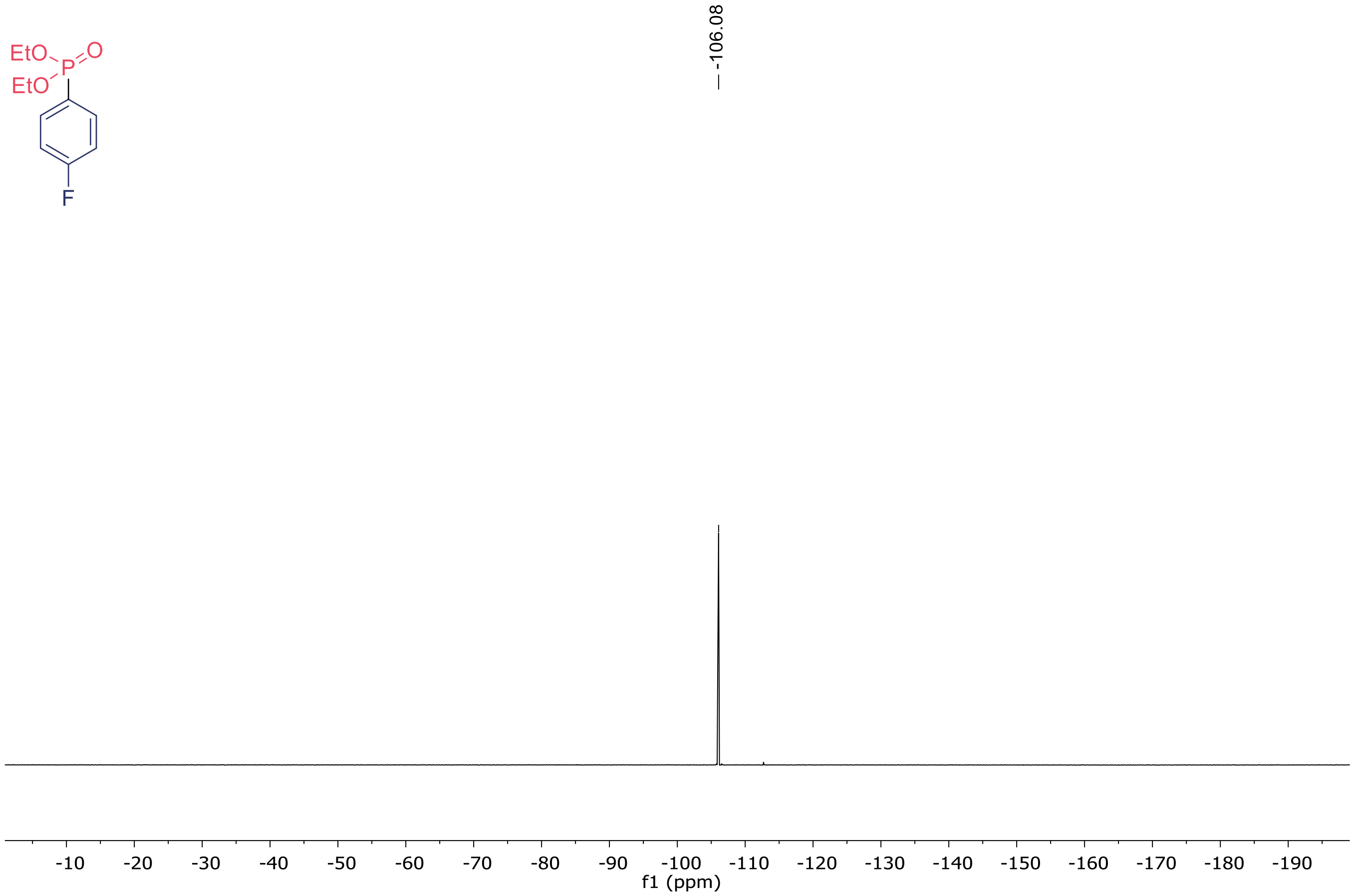

^{19}F NMR (376 MHz, $CDCl_3$) of compound **22**.

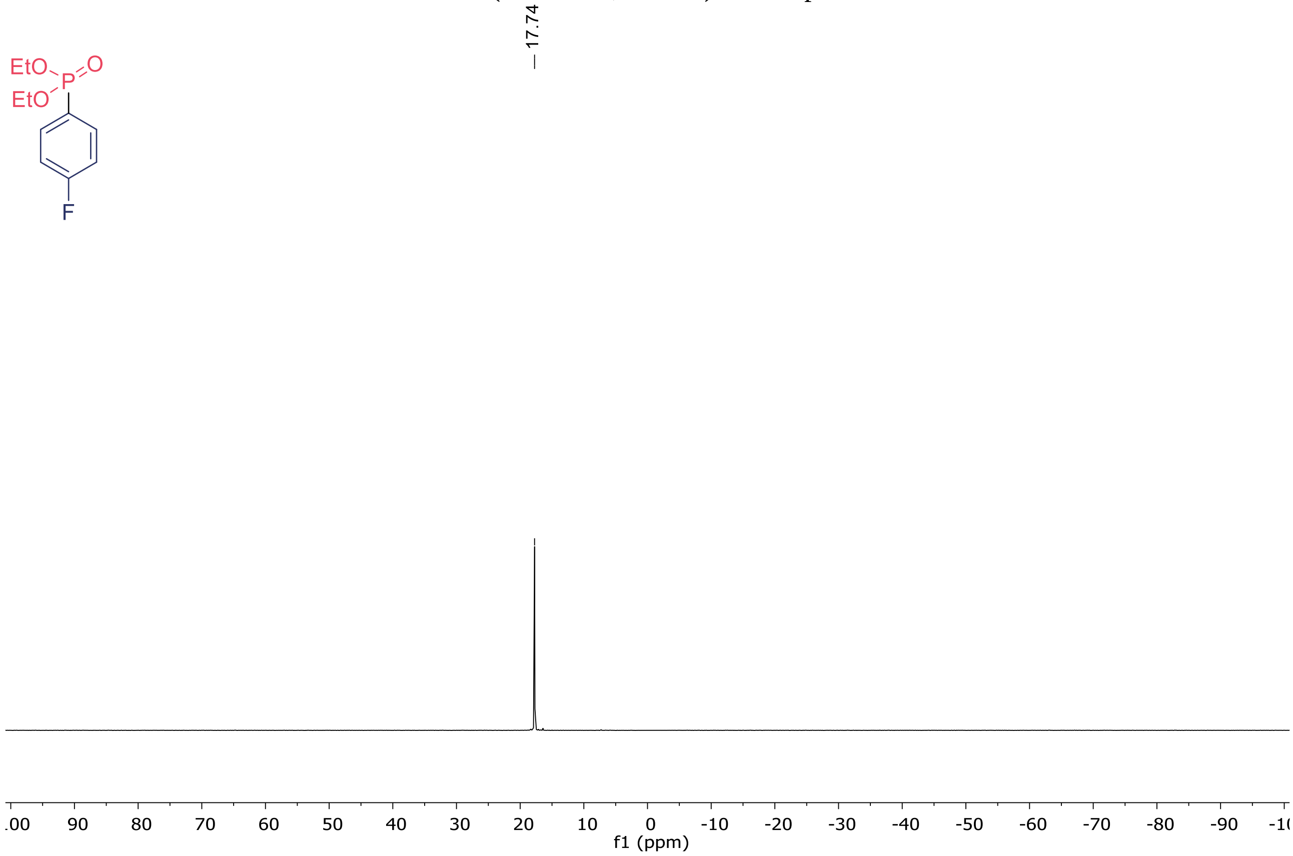

^{31}P NMR (162 MHz, $CDCl_3$) of compound **22**.

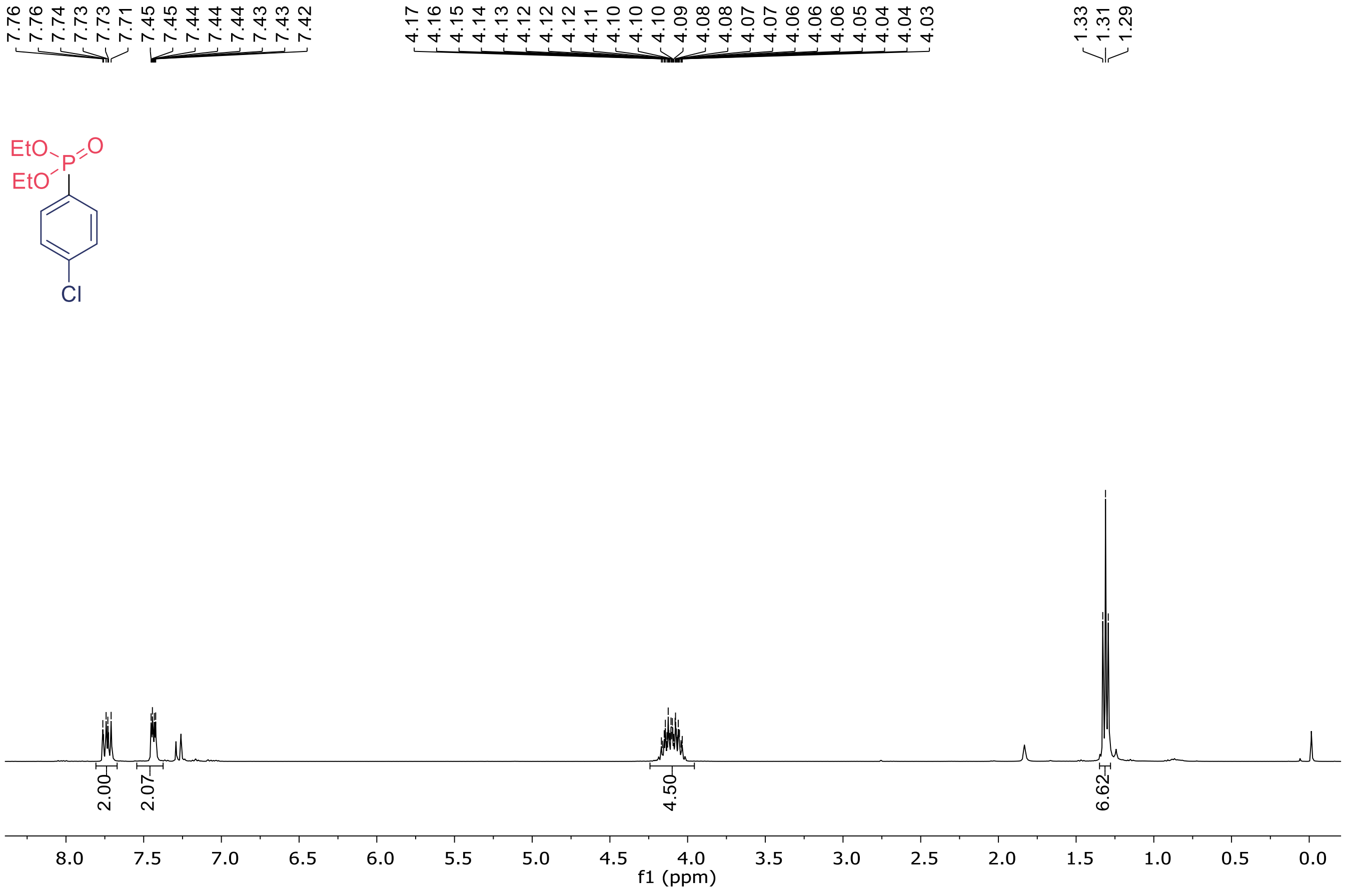

^{1}H NMR (400 MHz, $CDCl_3$) of compound **23**.

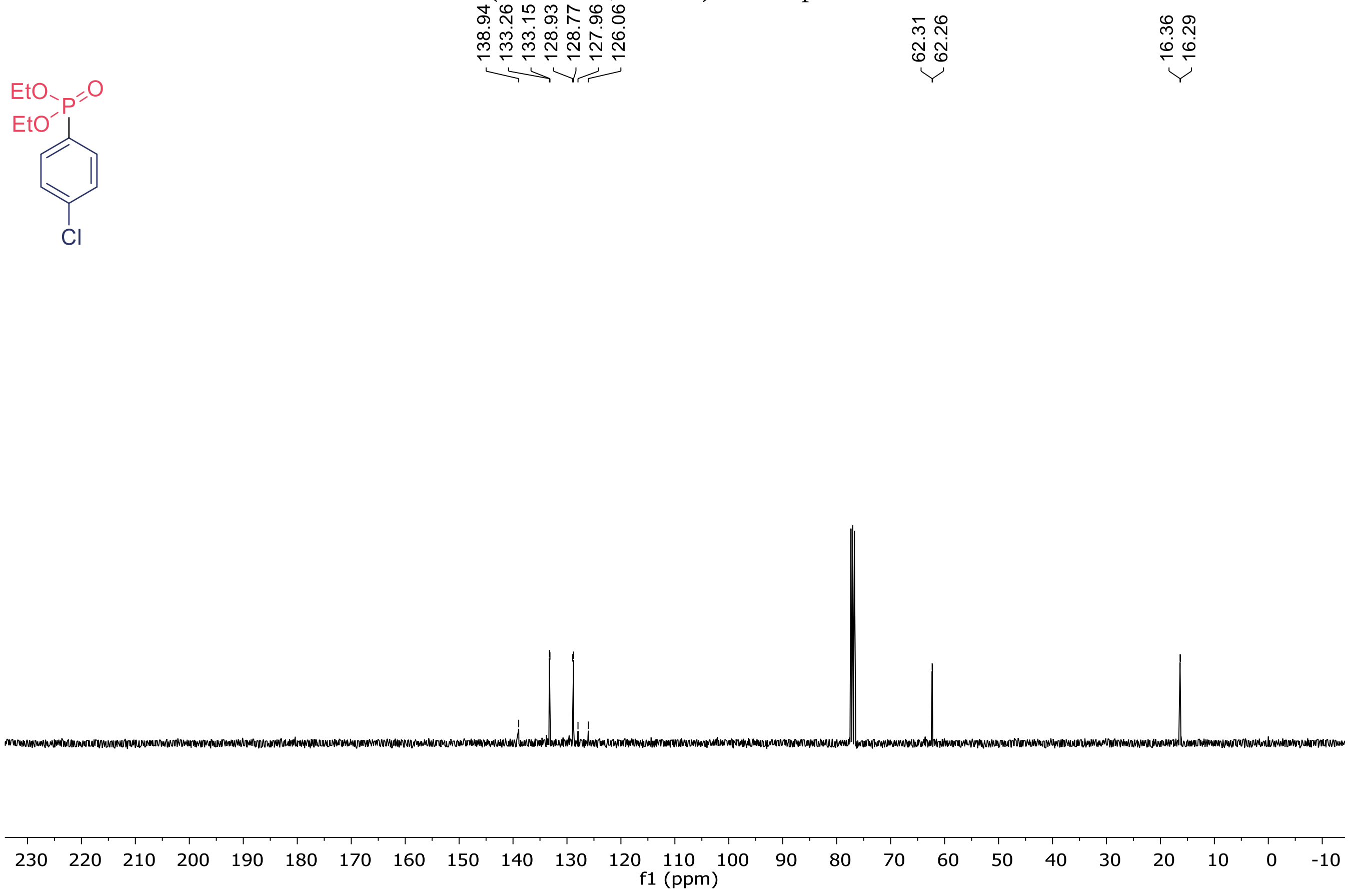

^{13}C NMR (101 MHz, $CDCl_3$) of compound **23**.

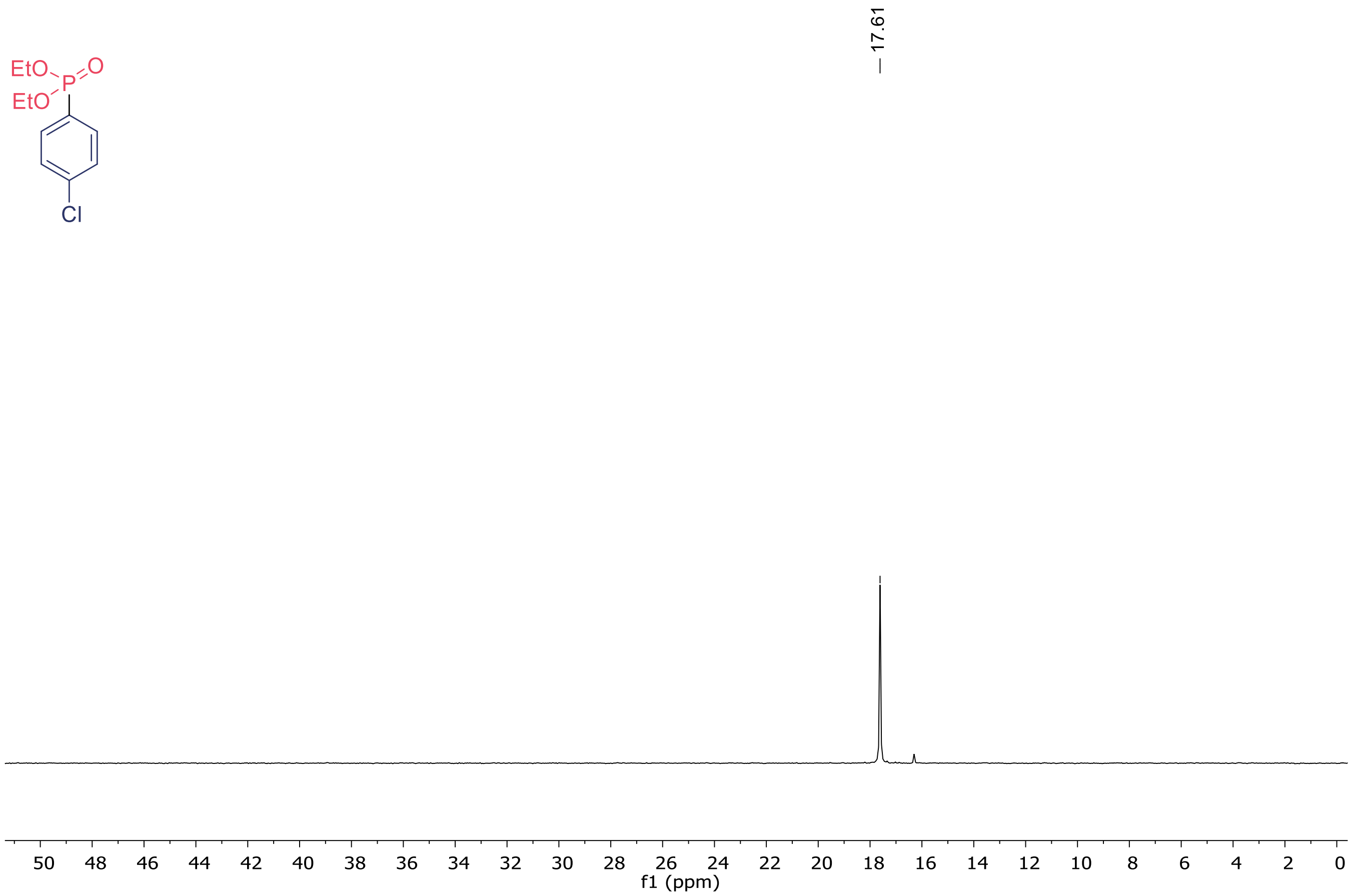

^{31}P NMR (162 MHz, $CDCl_3$) of compound **23**.

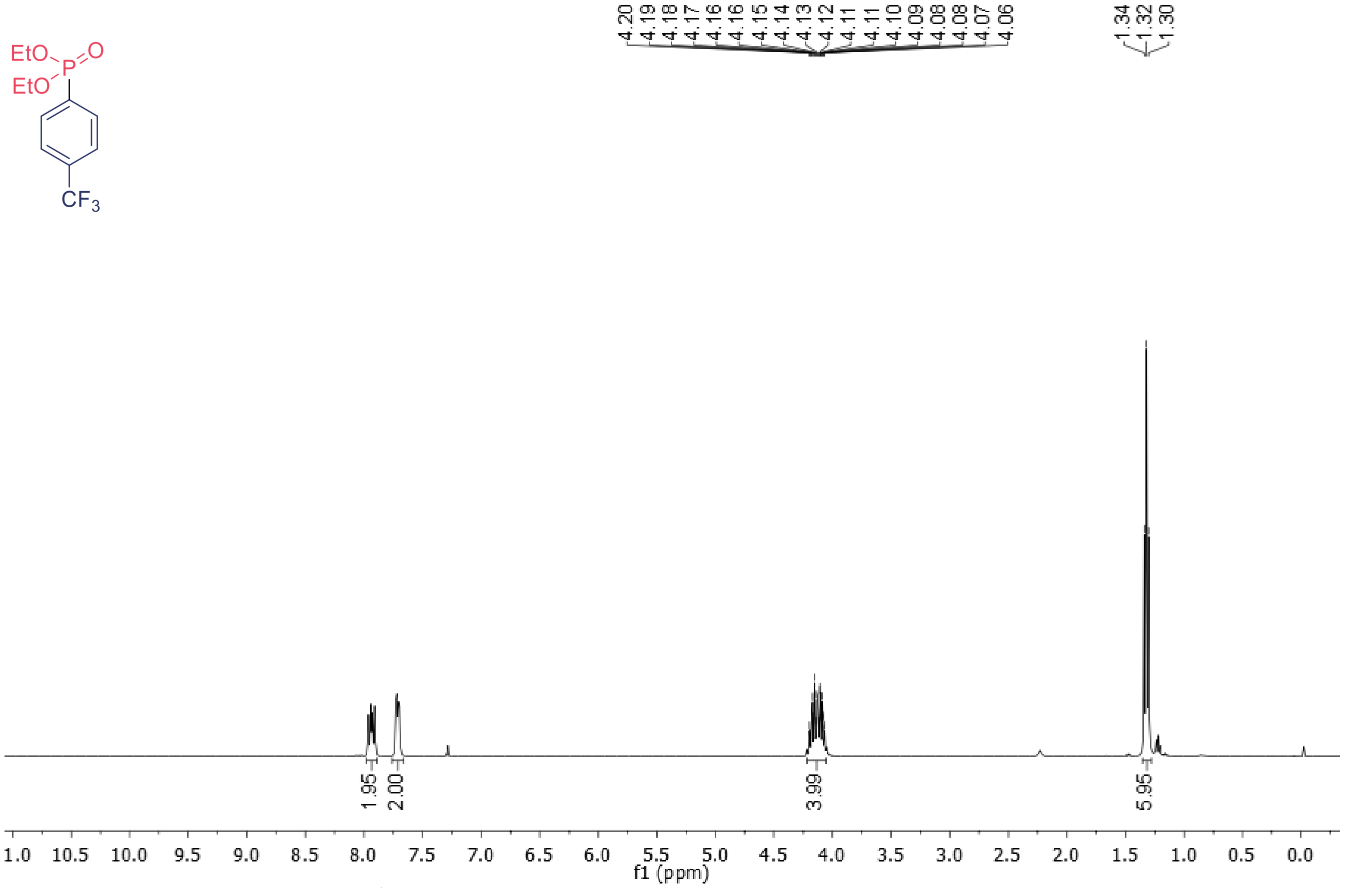

^{1}H NMR (400 MHz, $CDCl_3$) of compound **24**.

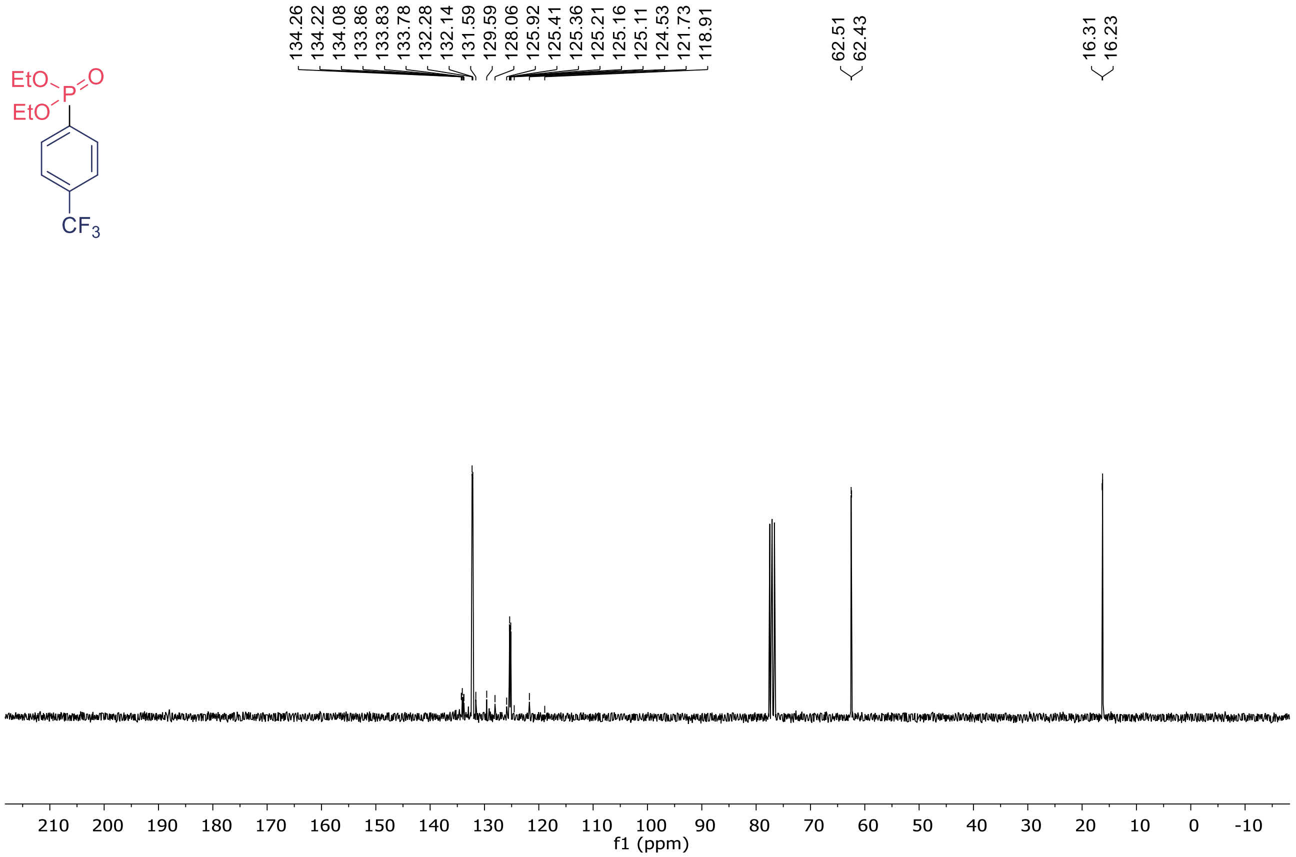

^{13}C NMR (101 MHz, $CDCl_3$) of compound **24**.

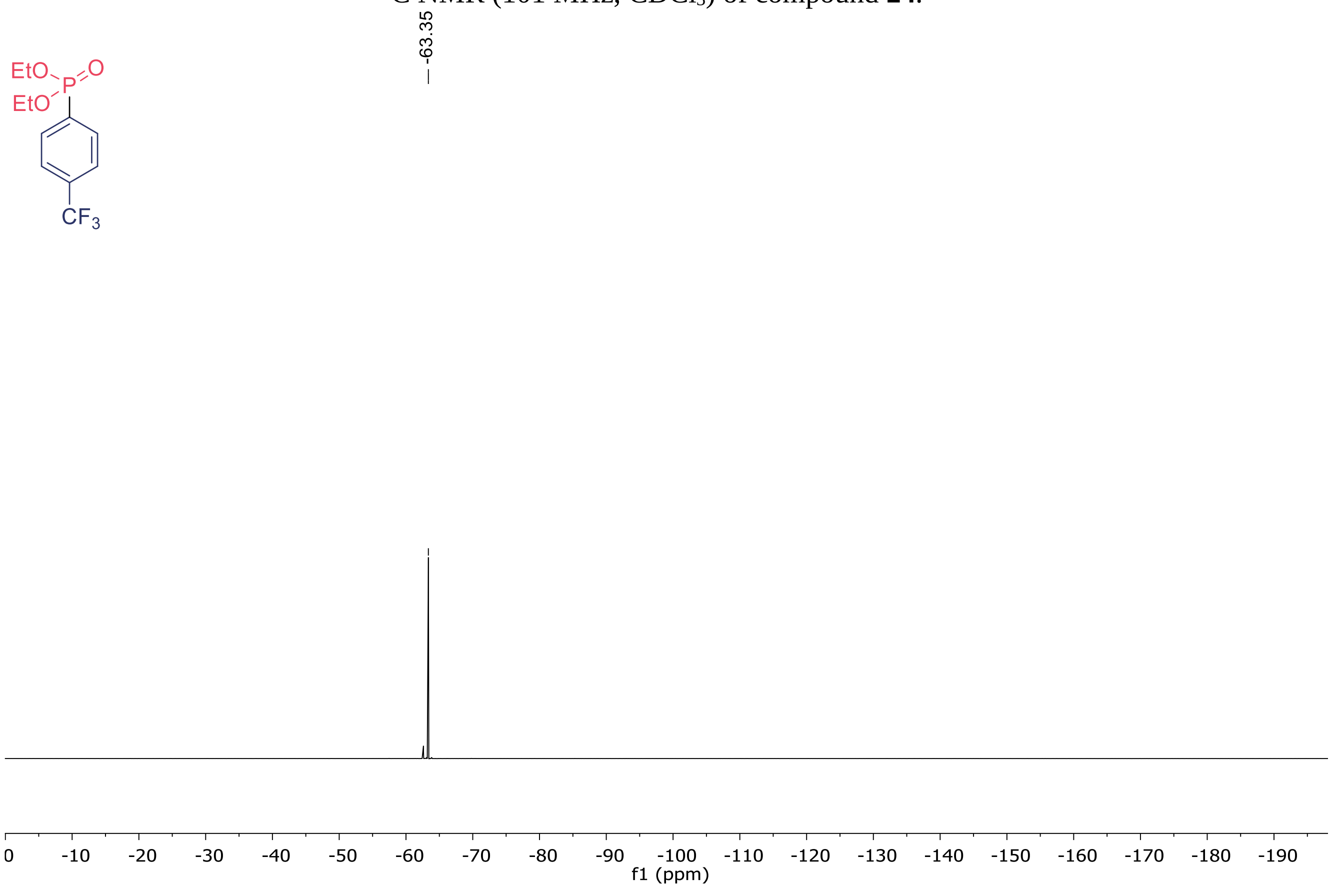

^{19}F NMR (376 MHz, $CDCl_3$) of compound **24**.

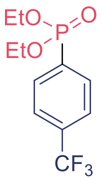

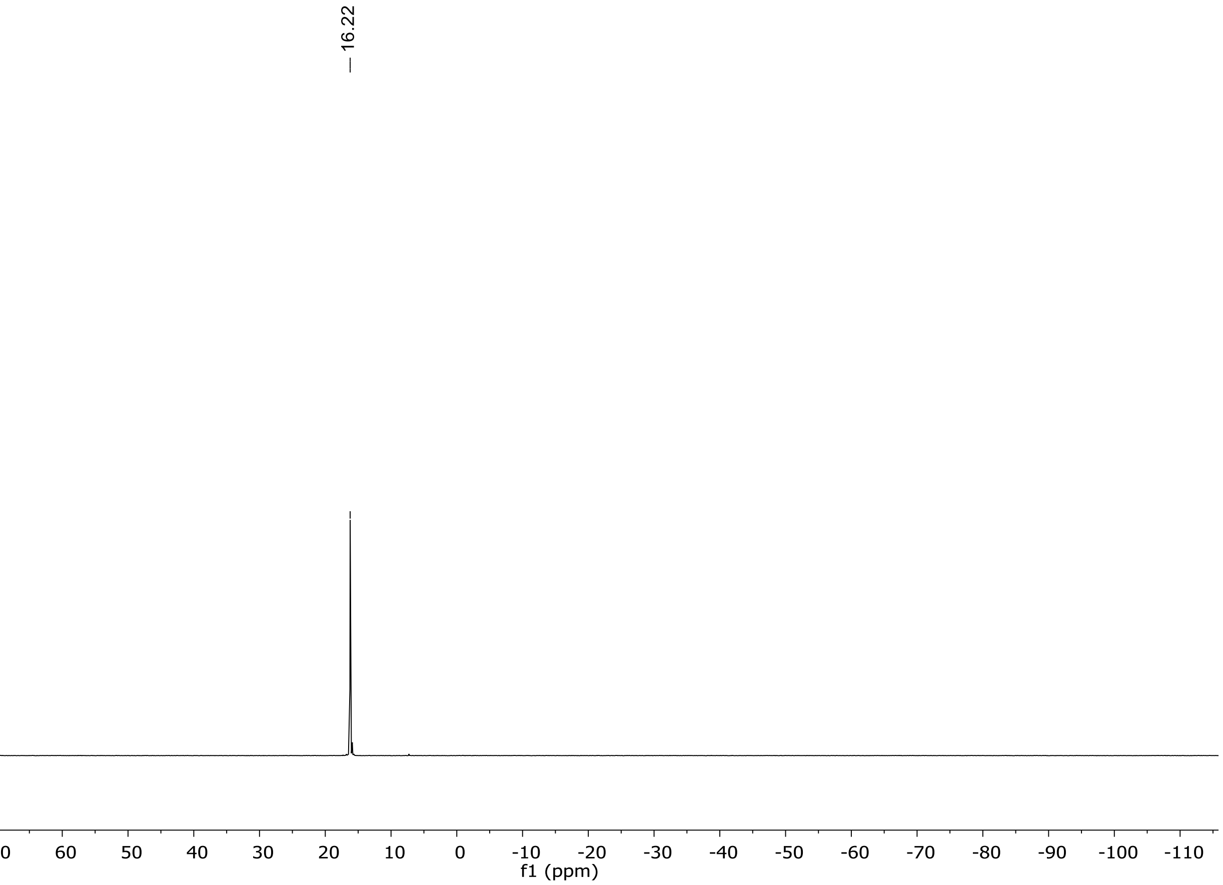

^{31}P NMR (162 MHz, $CDCl_3$) of compound **24**.

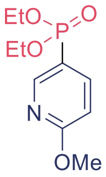

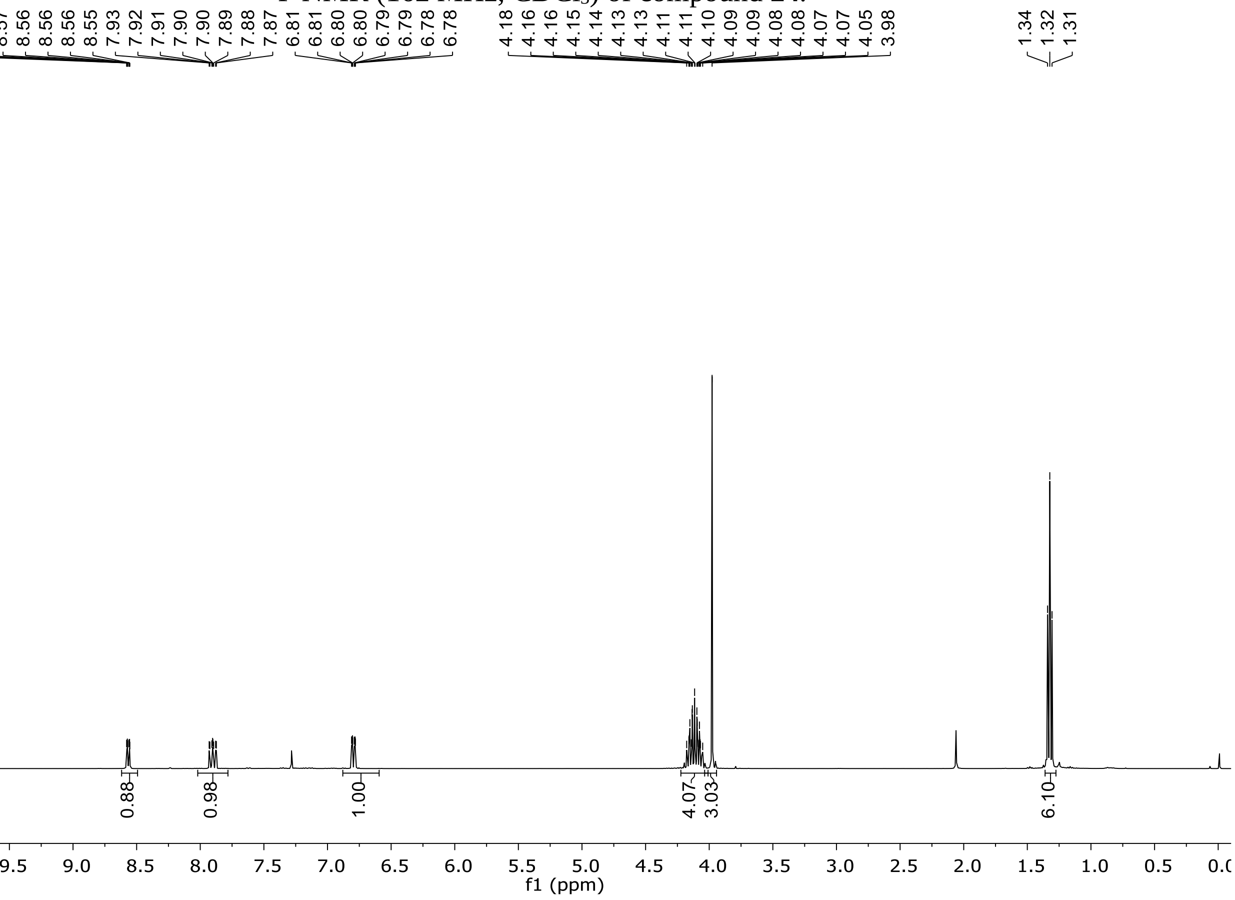

^{1}H NMR (400 MHz, $CDCl_3$) of compound **25**.

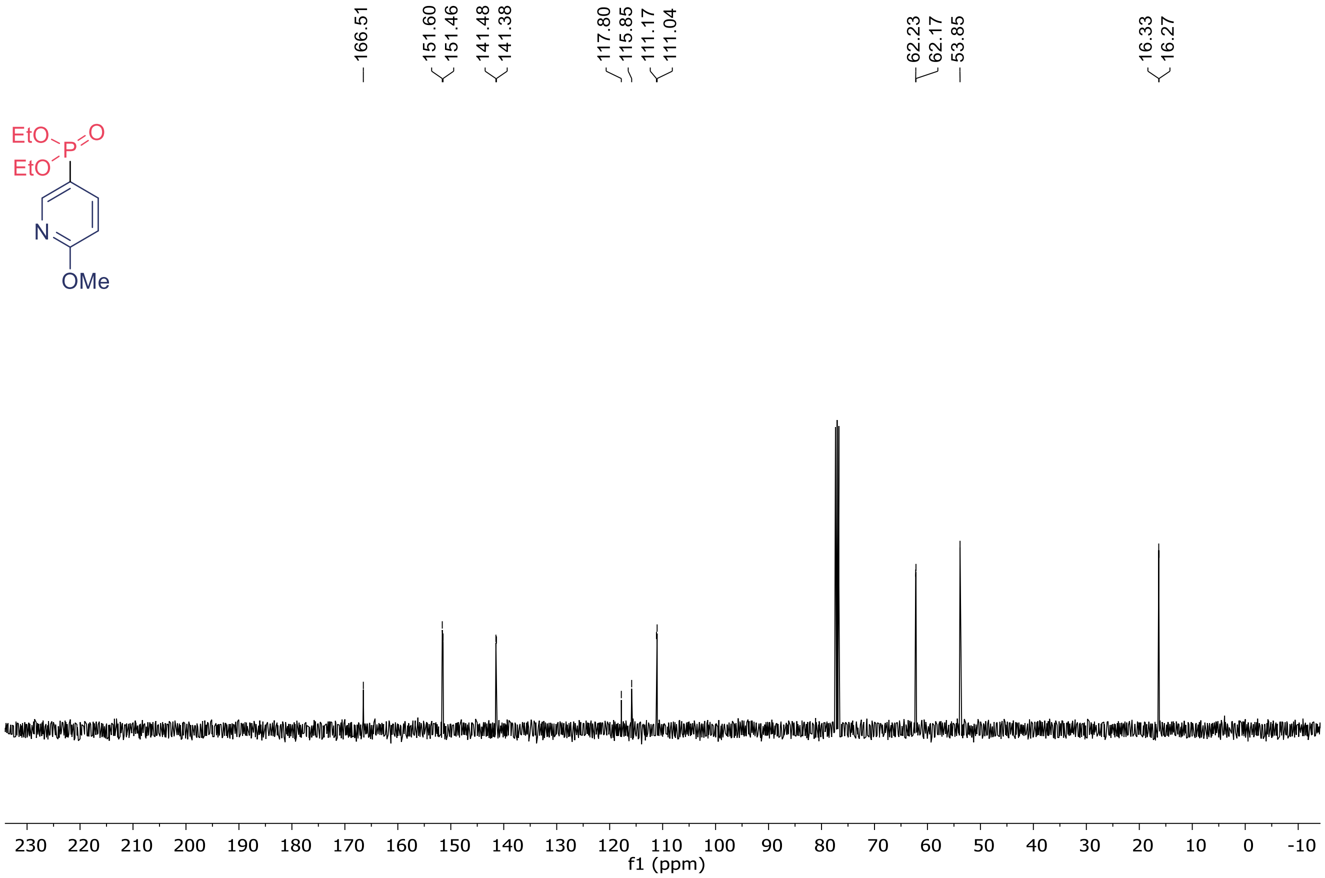

^{13}C NMR (101 MHz, $CDCl_3$) of compound **25**.

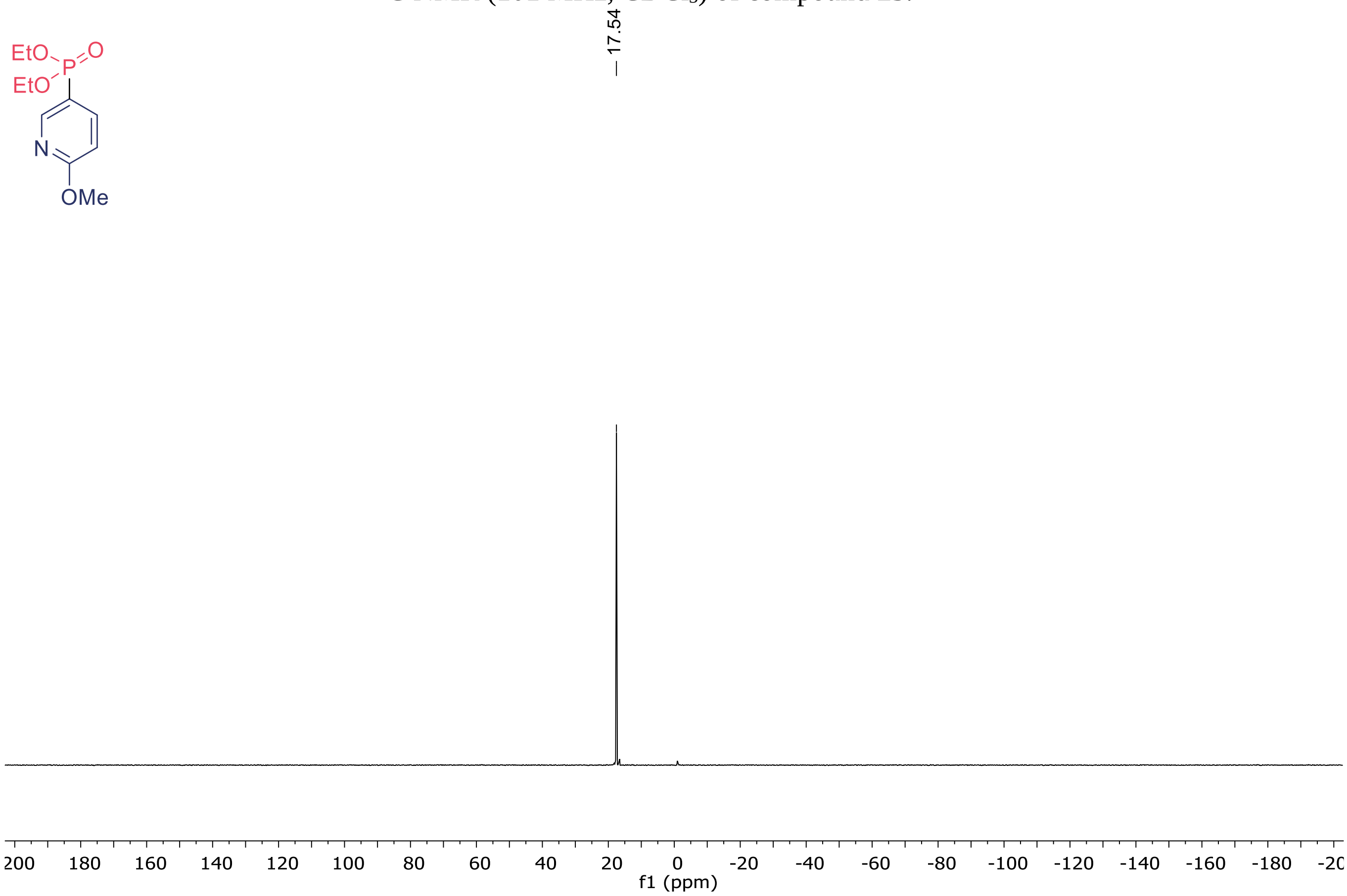

^{31}P NMR (162 MHz, $CDCl_3$) of compound **25**.

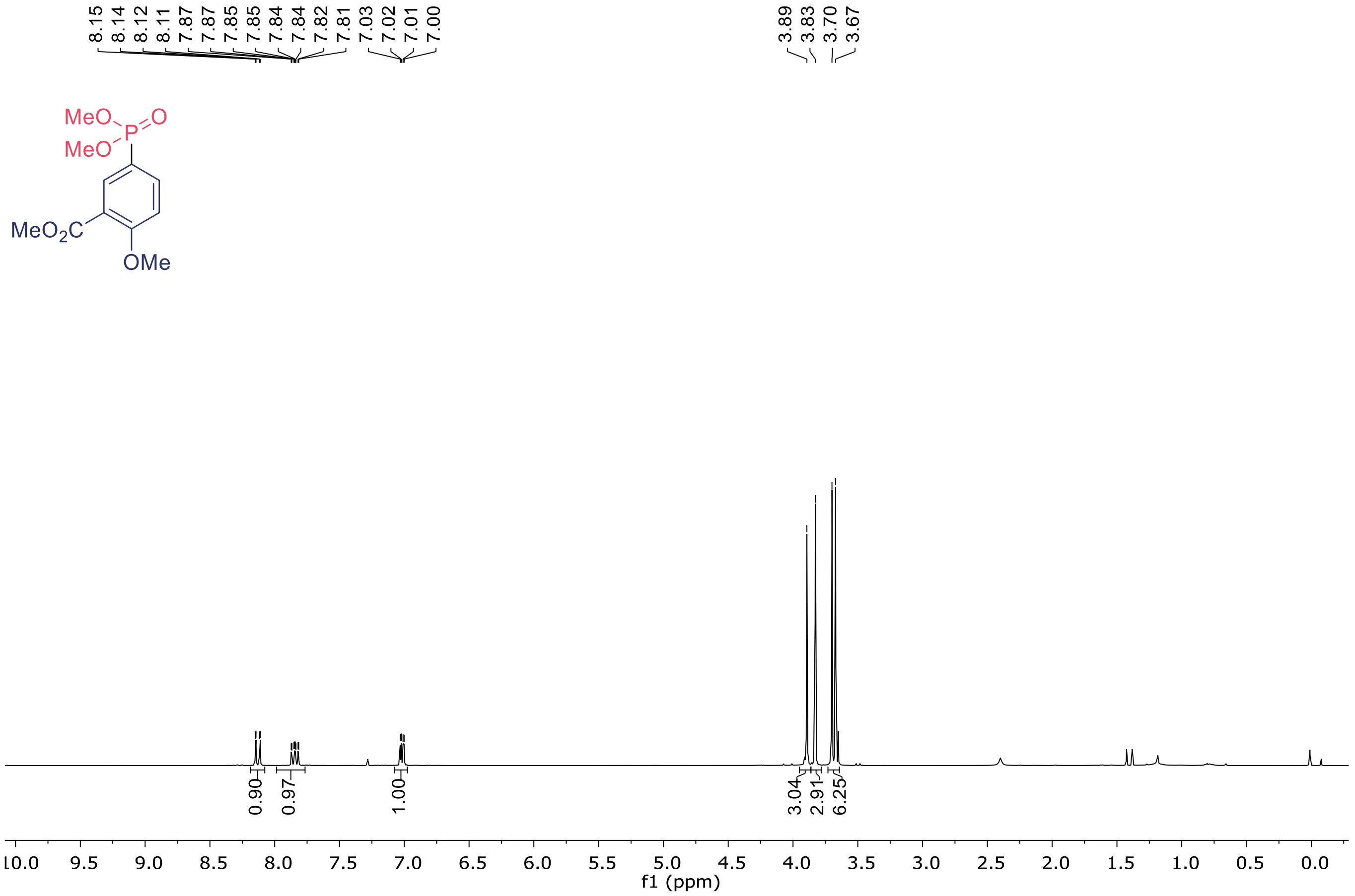

^{1}H NMR (400 MHz, $CDCl_3$) of compound **26**.

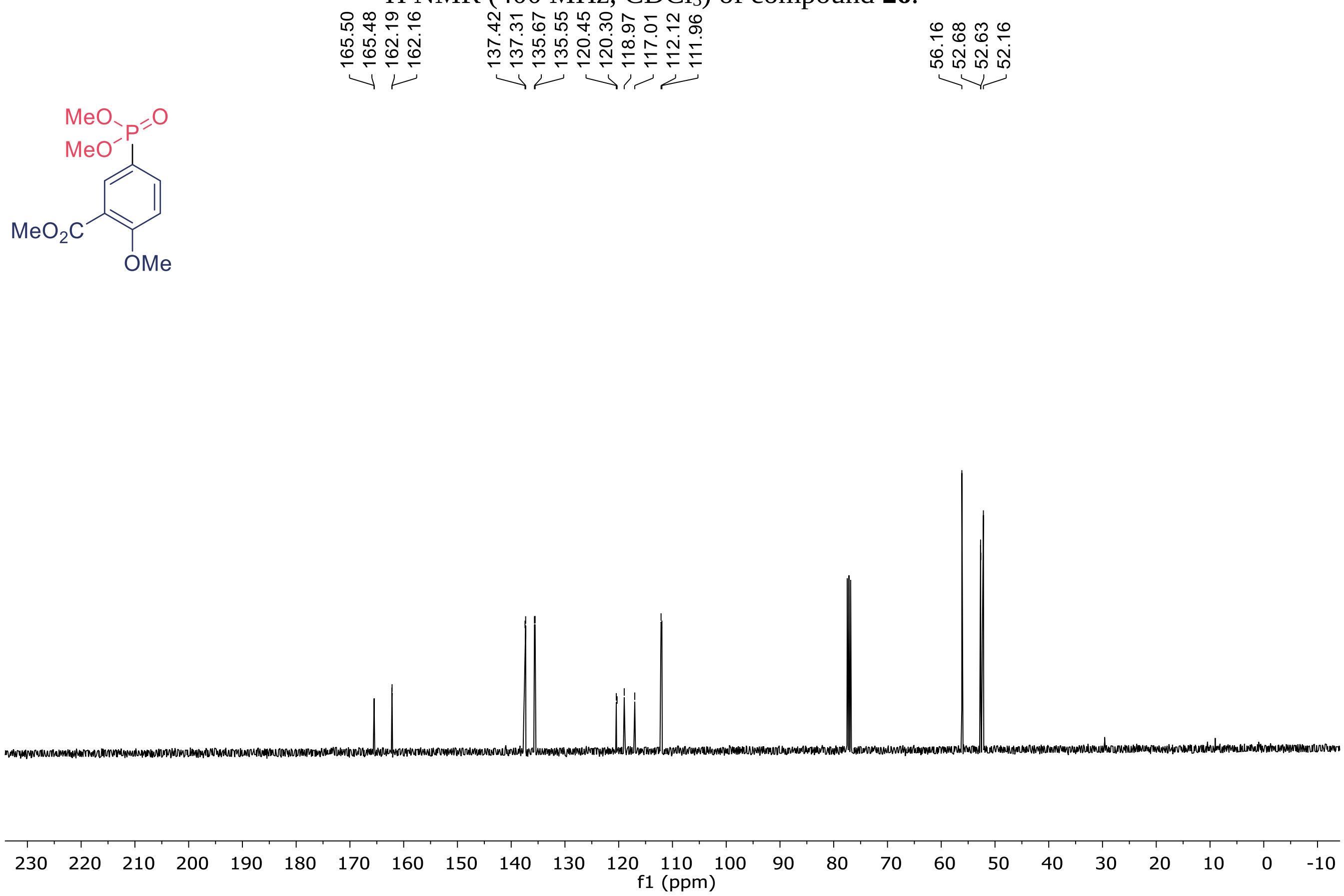

^{13}C NMR (101 MHz, $CDCl_3$) of compound **26**.

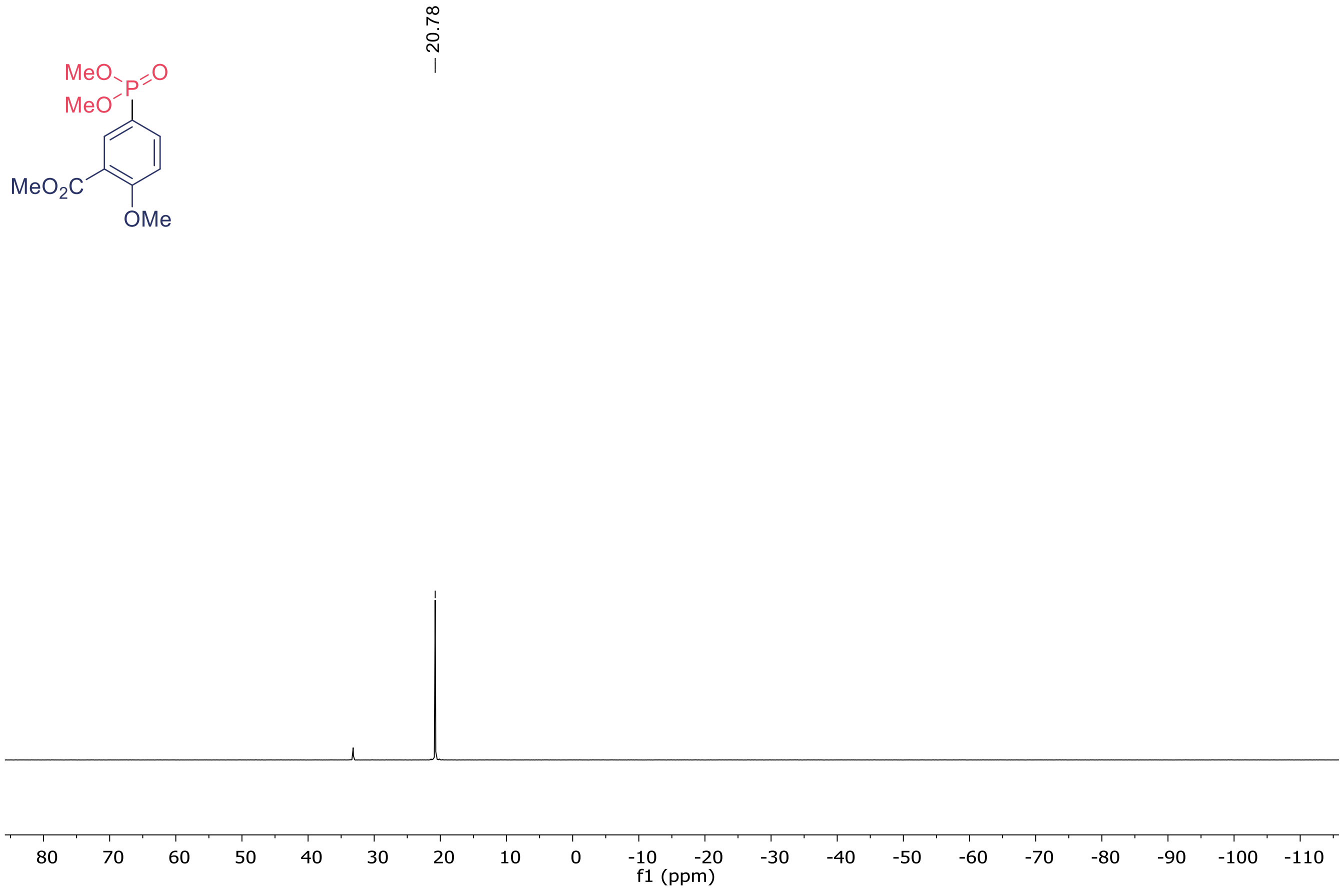

^{31}P NMR (162 MHz, $CDCl_3$) of compound **26**.

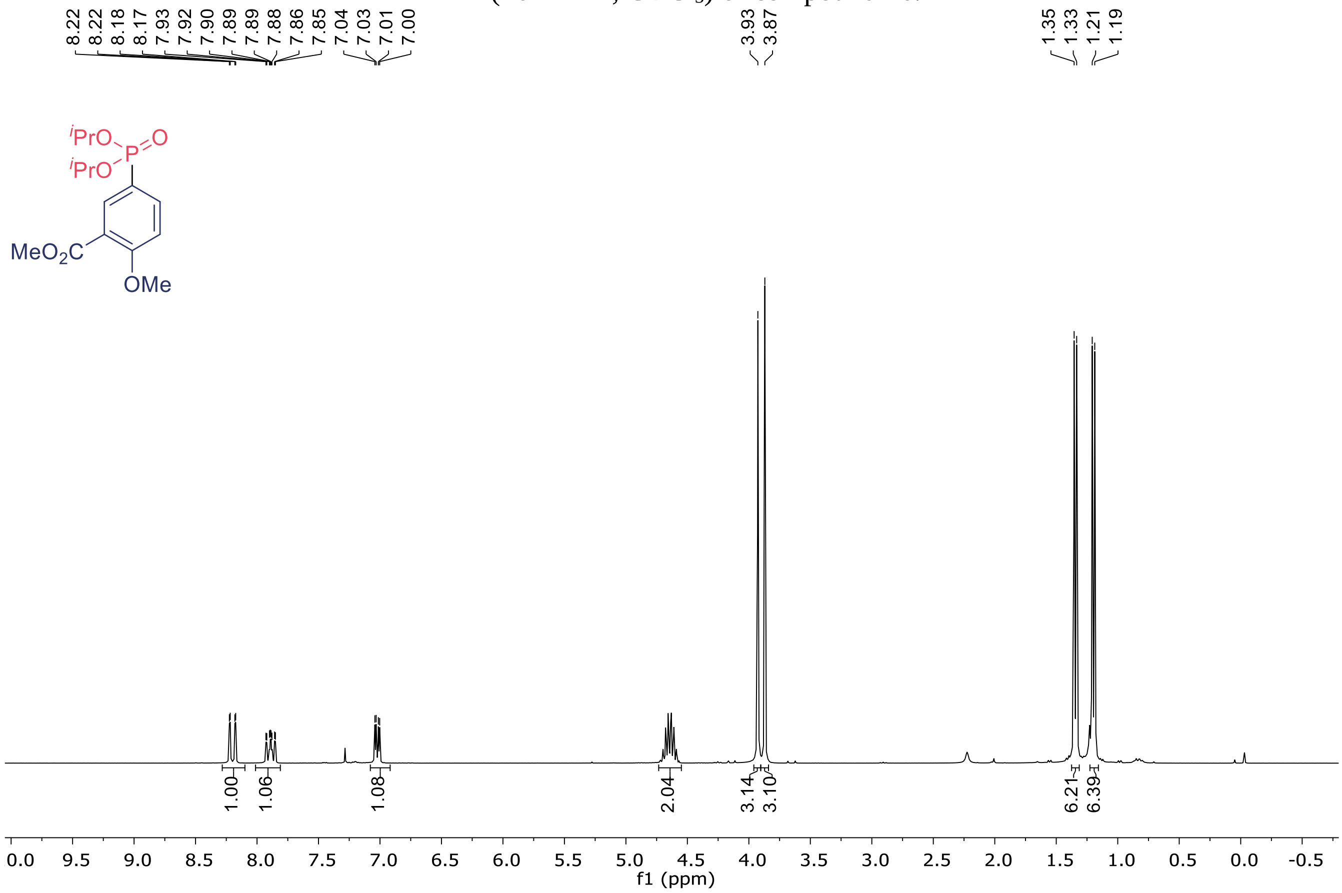

^{1}H NMR (300 MHz, $CDCl_3$) of compound **27**.

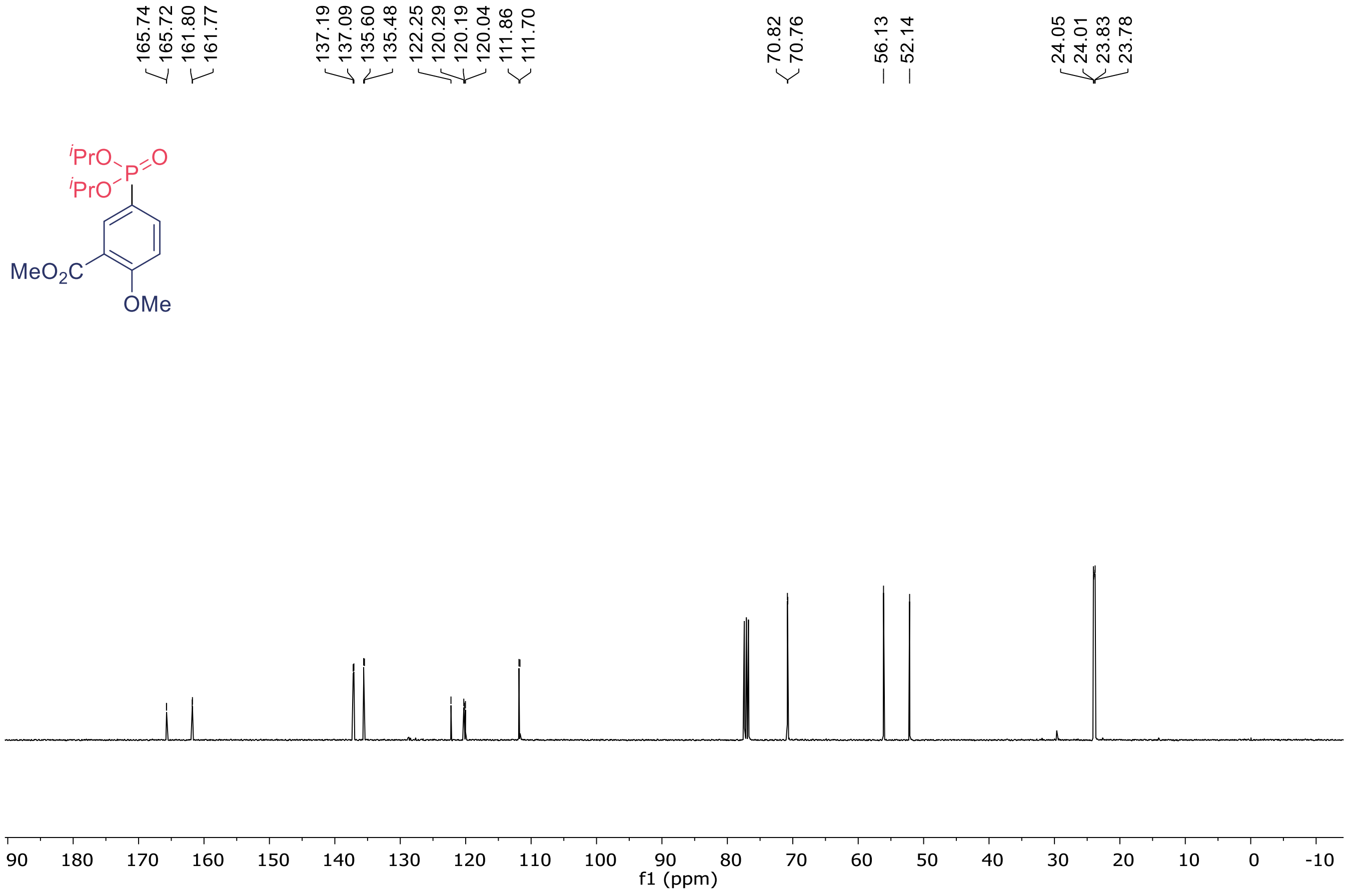

^{13}C NMR (75 MHz, $CDCl_3$) of compound **27**.

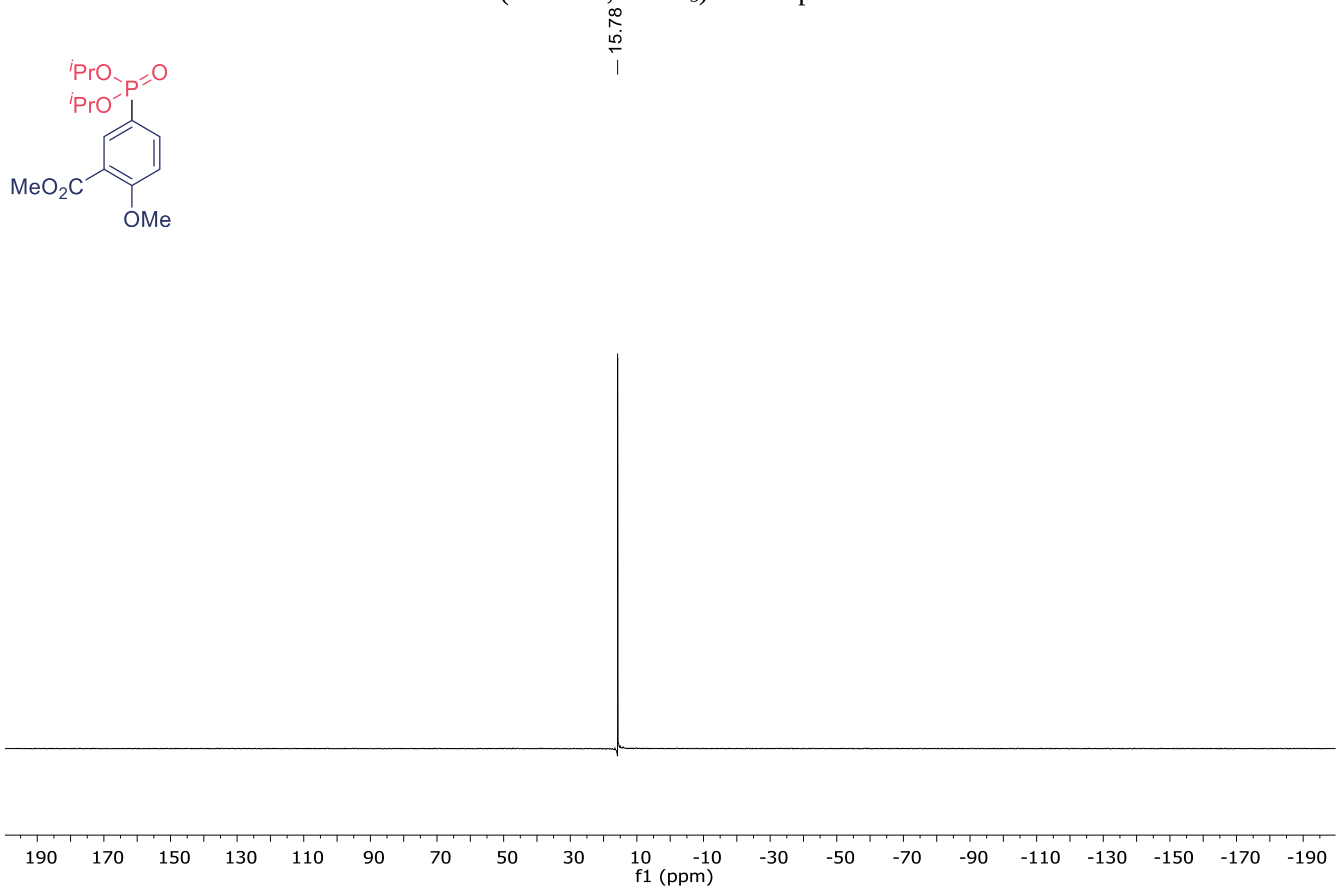

^{31}P NMR (122 MHz, $CDCl_3$) of compound **27**.

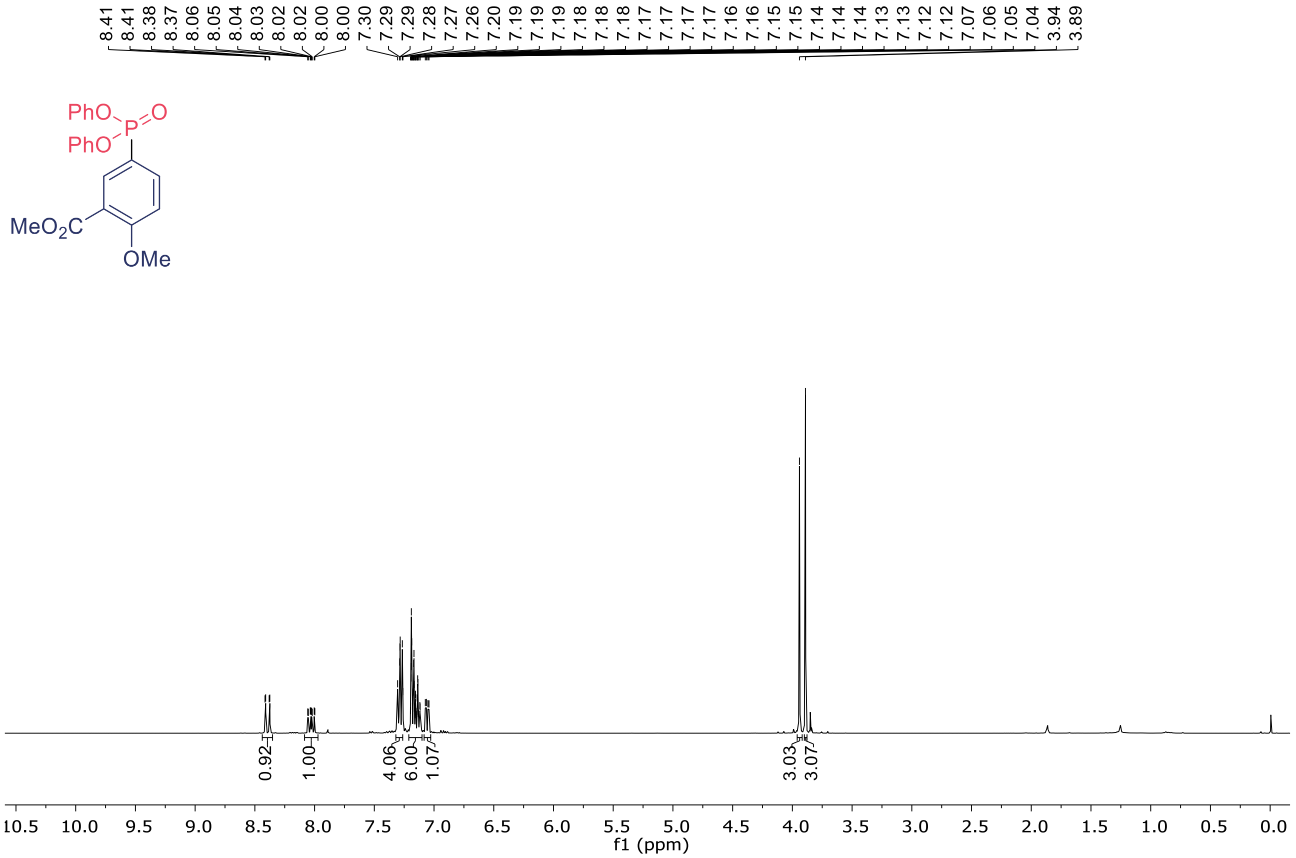

^{1}H NMR (400 MHz, $CDCl_3$) of compound **28**.

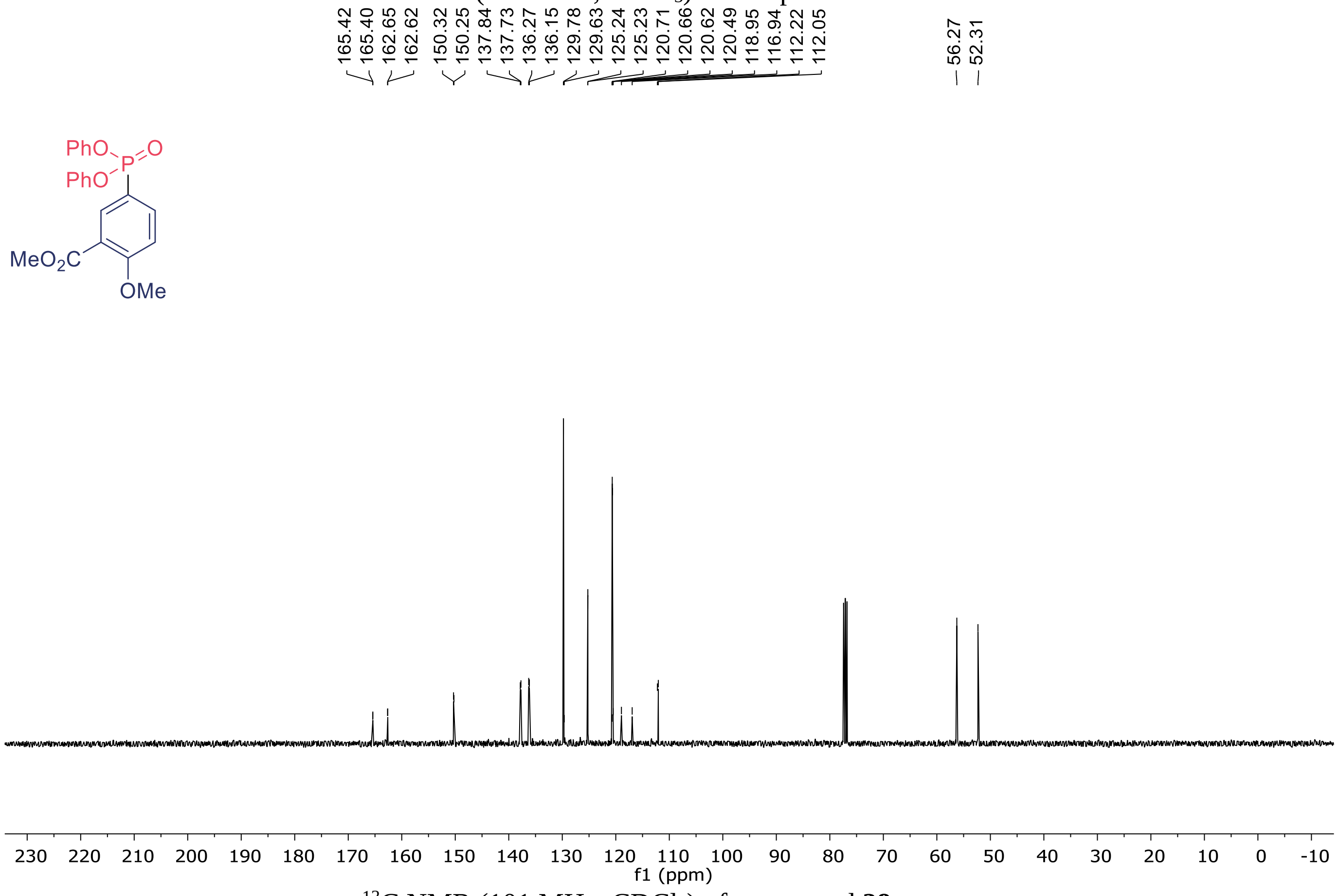

^{13}C NMR (101 MHz, $CDCl_3$) of compound **28**.

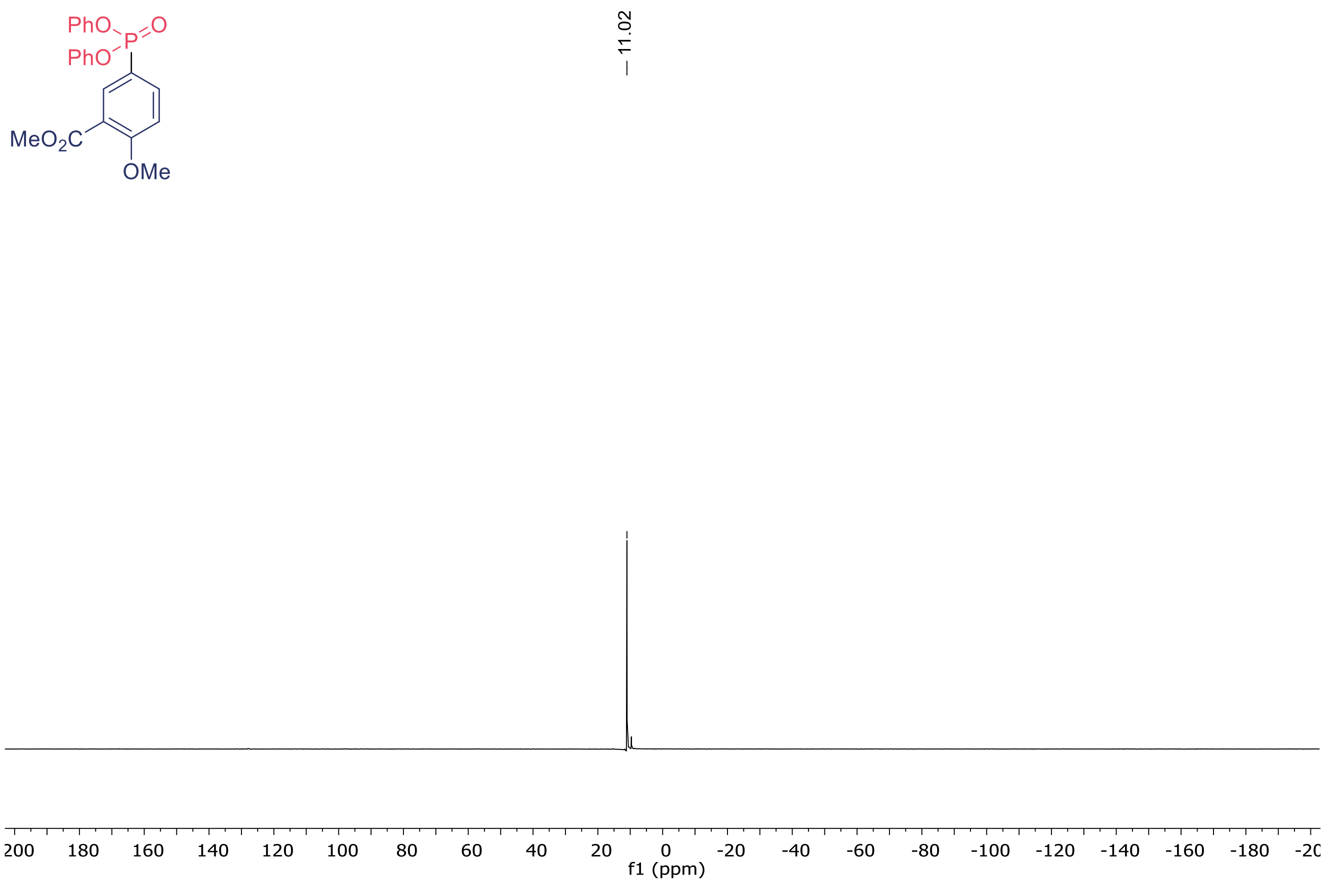

^{31}P NMR (162 MHz, $CDCl_3$) of compound **28**.
